# Identification of gene family members and a key structural variation reveal important roles of OVATE genes in regulating tea (*Camellia sinensis*) leaf development

**DOI:** 10.3389/fpls.2022.1008408

**Published:** 2022-09-23

**Authors:** Yanlin An, Xiaobo Xia, Tingting Jing, Feng Zhang

**Affiliations:** ^1^Department of Food Science and Engineering, Moutai Institute, Renhuai, China; ^2^CIMMYT-JAAS Joint Center for Wheat Diseases/Key Laboratory of Germplasm Innovation in Downstream of Huaihe River (Nanjing) Ministry of Agriculture and Rural Affairs, Nanjing, China; ^3^State Key Laboratory of Tea Plant Biology and Utilization, Anhui Agricultural University, Hefei, China

**Keywords:** OVATE gene family, tea tree, leaf development, gene expression, structural variation

## Abstract

OVATE genes are a new class of transcriptional repressors with important regulatory roles in plant growth and development. Through genome-wide analysis of the OVATE gene family of tea plants, 26 and 13 family members were identified in cultivated and ancient tea plants, respectively. Syntenic results showed that OVATE gene family in cultivated tea plants may have experienced a special expansion event. Based on phylogenetic tree analysis, all OVATE genes were divided into four groups, and the third group had the largest number, reaching 16. Transcriptome data from different organs and populations indicated that many OVATE family members were highly expressed in young shoots and leaves, and their expression levels gradually decreased as tea leaves developed. Finally, the expression trends of the six key candidate genes were verified by RT-qPCR, which were consistent with the transcriptome results, indicating that the ovate gene family plays an important role in regulating the process of tea leaf development. In addition, we identified a key structural variation with a length of 184 bp, and the population genotyping showed that it was closely related to the area of tea leaves. Our research provides an important clue for further exploring the function of ovate gene family in tea plants and the development mechanism of tea leaves.

## Introduction

As one of the most important vegetative organs and receptors of higher plants, leaves participate in photosynthesis, respiration, and transpiration of plants, and provide nutrients and power for plant growth and mineral absorption. At the same time, it can also respond to changes in the external environment and play a role in the signal transmission process of plants against various biotic and abiotic stresses (Kierzkowski et al., 2019; Xiong et al., 2021). For many grain and cash crops, leaf shape can also affect crop yield and mechanized harvesting efficiency (Wang et al., 2021). Tea [*Camellia sinensis* (L.) O. Kuntze] has become one of the three most popular non-alcoholic beverages because of its rich taste, pleasant aroma and high health value (Liu et al., 2020). For tea plants, leaves are not only vegetative organs, but also the main economic harvest. As the two most widely cultivated tea varieties, *Camellia sinensis* var. *assamica* (CSA, also known as large leaf species) and the *Camellia sinensis* var. *sinensis* (CSS, also known as small leaf species) are different in quality, morphology and stress resistance, among which the most significant difference is that CSA (mainly arbor and small arbor) have larger leaf area than CSS varieties (mainly shrub), while the latter has stronger adaptability to low temperature and drought. However, the key mutation sites or regulatory factors causing these differences have not been successfully identified (Li et al., 2020).

Leaf development is closely related to factors such as mineral nutrition, hormones, light, temperature, and transcriptional regulation of genes. Many studies have shown that the OVATE (*OFPs*) gene family is not only important in regulating plant responses to drought and salt stress, but also indispensable in regulating plant fruit shape and leaf size (Guan et al., 2021). OVATE family proteins are a new type of plant-specific transcription regulator, which can mediate plant growth and development, such as the formation of secondary cell wall, the growth of ovule and vascular bundle, and the signal transduction of brassinolide (Yang et al., 2016). OVATE protein contains a C-terminal domain of about 70 amino acids, which is called DUF623 domain. It is found that this domain is unique to plants, and protein containing this conserved domain is named OVATE family protein. The first OVATE gene was identified as a major quantitative trait locus controlling tomato fruit shape, which as a plant growth inhibitory protein also negatively regulates tomato leaf and flower growth and development (Liu et al., 2002). Studies in *Arabidopsis* suggest that OFPs play a role in regulating plant growth such as cotyledon development and floret shape (Wang et al., 2007; Zhou et al., 2021); In banana, *MaOFP1* interacts with banana *MuMADS1* protein to control fruit development (Liu et al., 2015), while ovate protein in pepper changes fruit shape by inhibiting *CaGA20ox1* expression (Tsaballa et al., 2011). In addition, recent studies have found that the ectopic overexpression of *PpOFP1* in peach trees can lead to oval leaves and siliques of *Arabidopsis thaliana*, and a 1.7 Mb chromosome inversion located downstream of it may activate the transcription of *PpOFP1*, thus inhibiting the fruit from elongating at the early stage of development to form a flat fruit (Zhou et al., 2021).

Protein interaction analysis confirmed that OFPs had close functional relationship with many other basic regulatory factors of plant development. As shown by Hackbusch et al. (2005), the interaction between *AtOFP1* and *BLH1* (*BLH1* is a member of TALE family protein) leads to the relocation of *BLH1* from nucleus to cytoplasm; Li et al. (2011) found that *AtOFP1* and *AtOFP4* can combine with the multi-protein transcription regulatory complex containing *BLH6* and *KNAT7* proteins to regulate the formation of secondary cell wall.

Plant hormones such as auxin, brassinosteroids, and gibberellins play crucial roles in plant growth and development, and OFPs have been shown to play a role in plant hormone pathways (Pagnussat et al., 2007). For example, Wang et al. (2007) found that *AtOFP1* plays a role in the gibberellin signaling pathway by regulating the expression of *AtGA20ox1*, a gene encoding a key enzyme in gibberellin biosynthesis; Over-expression of *OsOFP2* showed that it could change the shape of rice leaves and seeds by down-regulating the expression level of *OsGA20ox7*, and inhibit the level of GA (Liu et al., 2014; Schmitz et al., 2015). At the same time, preliminary research shows that OVATE gene family can also play a role in plant stress regulation. The results of drought and freezing injury showed that over-expressed *OsOFP6* could slow down the water loss rate of rice, reduce the accumulation level of H_2_O_2_ in plants, and reduce the relative electrical conductivity (Ma et al., 2017). Wax is closely related to plant drought resistance. Tang et al. (2018) revealed that *AtOFP8* can participate in the regulation of epidermal wax synthesis in *Arabidopsis thaliana*.

In present study, we comprehensively identified the members of OVATE gene family in tea plants, and revealed the close relationship between candidate family members and tea leaf development through population transcriptome and RT-qPCR analysis. At the same time, based on high-throughput capillary electrophoresis, a key structural mutation related to leaf size was identified. This study will provide a global perspective on the evolution, expansion, and functional characteristics of the OVATE gene family in tea plants, and indicate important clues for further studies on its involvement in tea plant development.

## Results

### Genome-wide identification of the OVATE gene family

**Figure 1:**
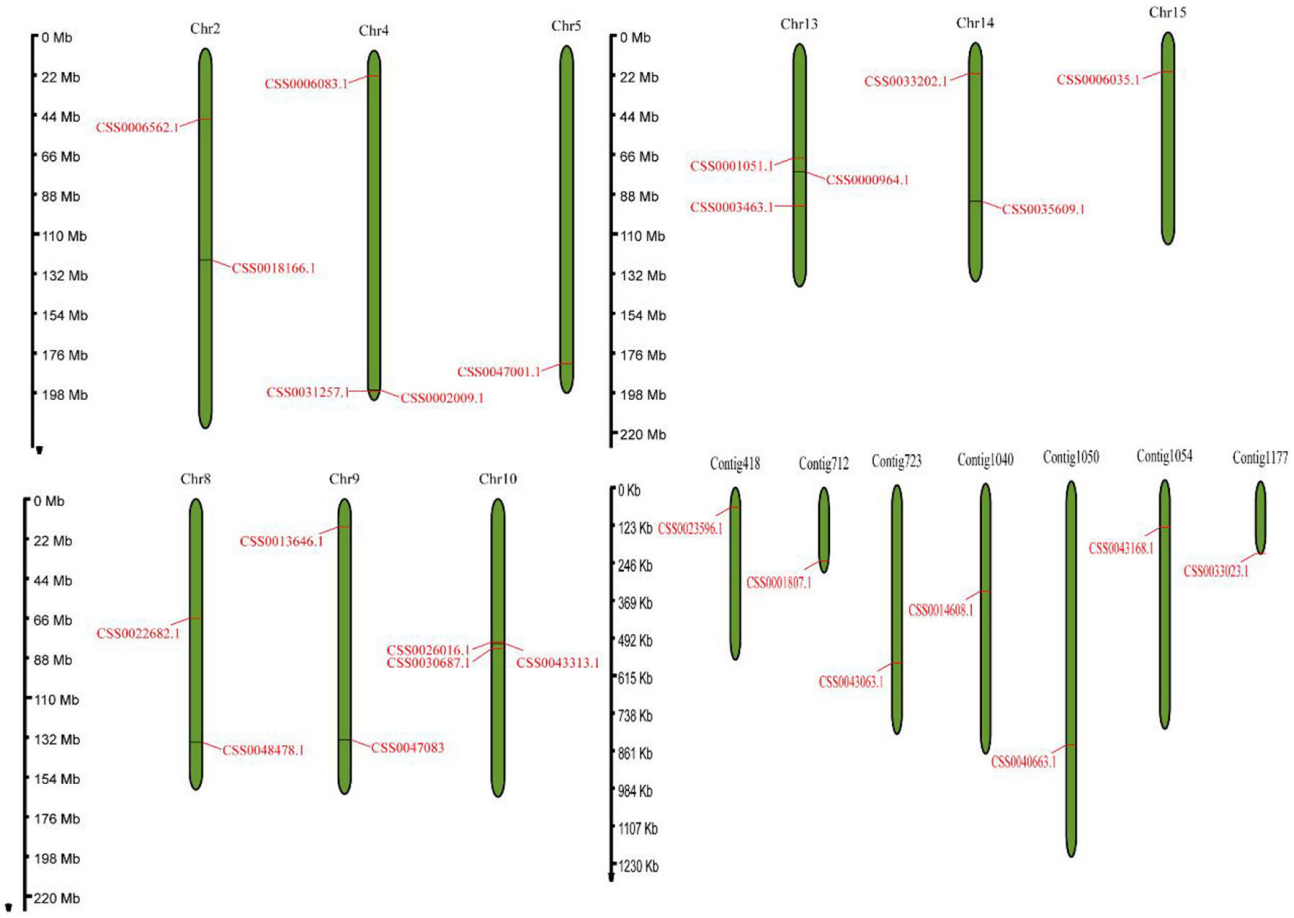
Genome location of the OVATE genes. The distribution of OVATEs on chromosome and contig with a scale bar was displayed in megabase (Mb).

Based on the protein sequence of “ShuChaZao” reference genome, (Xia et al., 2020), the genome-wide identification of OVATE gene family was carried out by hmmsearch software. A total of 26 OVATE genes were identified, as shown in Figure 1, these genes were distributed on 9 chromosomes and 7 contigs. All Contig have only one OVATE gene, and the chromosomes contain one to three genes. Chromosomes 4, 10, and 13 all contain three ovate genes, while chromosomes 2, 8, 9, and 14 each contain two ovate genes. In particular, three genes on chromosome 10 form a co-expressed gene cluster. Further prediction analysis showed that the molecular weight (MW) and isoelectric point (pI) of 26 ovate-encoded protein ranged from 11.2 kDa to 47.2 kDa (with an average of 28.3 kDa) and 4.61 to 9.99 (with an average of 7.6), respectively (Supplementary Table S1). In addition, we also performed the identification of the OVATE gene family on the ancient tea plant genome to explore its differences from cultivated tea plants. Interestingly, 13 OVATE family members were identified in ancient tea plants (Supplementary Table S1, average MW and pI were 29.3 kDa and 8.1, respectively), which may suggest that the OVATE gene has undergone expansion in cultivated tea plants.

### Phylogenetic and conserved protein motifs analysis of the OVATE gene family

**Figure 2:**
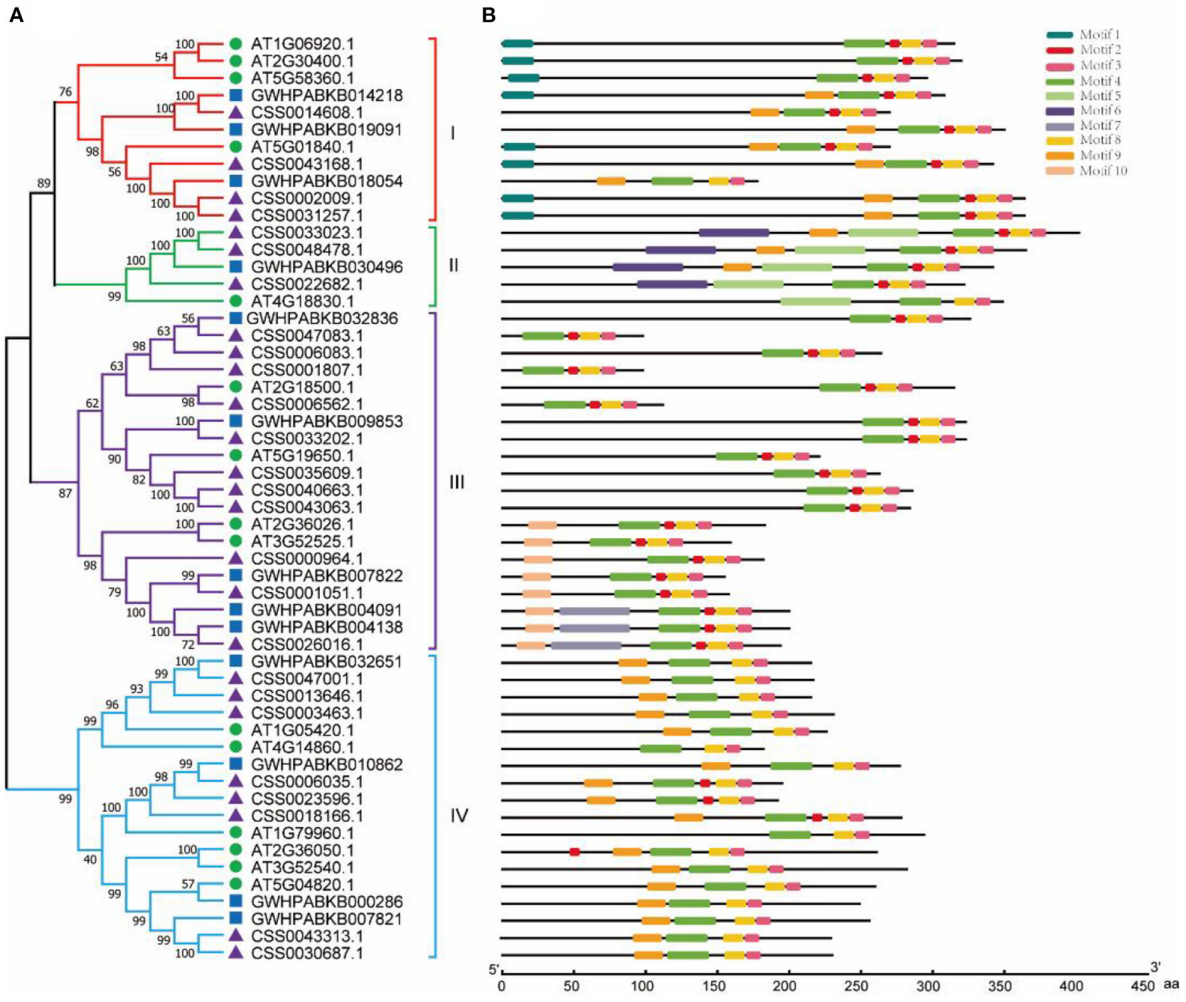
Phylogenetic and structural analysis of OVATE proteins. **(A)** The phylogenetic tree of OVATE proteins from *Arabidopsis thaliana* and tea plant constructed using the neighbor-joining method. Green circle, blue square and purple triangle represent *Arabidopsis thaliana*, ancient tea tree (“DASZ”) and cultivated tea tree (“ShuChaZao”) respectively. **(B)** Protein motifs of OVATE.

According to the amino acid sequence, we constructed a phylogenetic tree by aligning OVATE gene from CSS (*CsOVATE*) with 13 OVATE gene from ancient tea tree (*CaOVATE*) and 15 from *Arabidopsis thaliana*. As shown in Figure 2A, all protein sequences are classified into 4 subfamilies. Among them, class I contained 4 *CsOVATEs* and 3 *CaOVATEs;* class II contained 3 *CsOVATEs* and 1 *CaOVATE*; class III contained 11 *CsOVATEs* and 5 *CaOVATEs*; and class IV contained 8 *CsOVATEs* and 4 *CaOVATEs*. These results indicate that OVATE gene family may have expanded in cultivated tea plants. Based on sequence features of OVATE proteins, 10 conserved motifs were predicted using MEME (https://meme-suite.org/meme/). Among them, motif 3, motif 4, and motif 8 are highly conserved and are recognized in all protein sequences, while motif 1 and motif 6 are only recognized in the group I and group II, respectively (Figure 2B). In addition, structural analysis showed that most OVATE genes in tea plants were single exon genes (Supplementary Figure S1).

### Analysis of cis-regulatory element distribution in OVATE promoters

**Figure 3:**
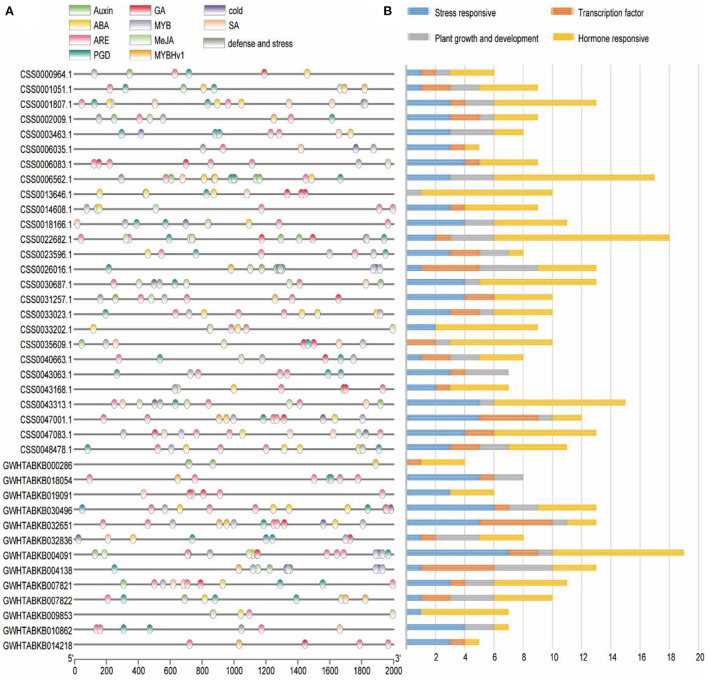
Analysis of cis-regulatory element distribution in the OVATE promoters. **(A)** The cis-elements in the OVATE were marked by different colors. **(B)** The number of cis-elements numbers in four categories.

In order to explore the regulatory network of OVATE gene in the process of tea plant growth and development, the sequence of 2 kb 5′-upstream region was extracted to identify cis-acting elements (Figure 3A). According to previous studies (Huang et al., 2022), all cis-acting elements are classified into four types including stress response, plant growth and development, hormone response and transcription factor (Figure 3B). In the stress response category, 15 LTRs (low temperature responsiveness), 85 AREs (anaerobic inducible elements) and 9 TC-rich repeats were found. There are 9 types of cis-acting elements in the hormone-responsive category, of which the four most are ABRE (50), TCA-element (31), TGACG-motif (28), and CGTCA-motif (28). In addition, there are 16 TGA-elements, 12 P-boxes, 12 AuxREs, 4 GARE-motifs, and 1 TGA-box. In the category of plant growth and development, 11 types of cis-acting elements were identified, and the first four types with the largest number were CAT-box (12), GCN4-motif (11), O2-site (10), and AT-rich sequence (9). The transcription factor category includes three types of cis-acting elements, MBS, MRE and MBSI, whose numbers are 26, 12, and 1, respectively. The detailed information is shown in Supplementary Table S1.

### Synteny analysis for OVATEs in tea plant

**Figure 4:**
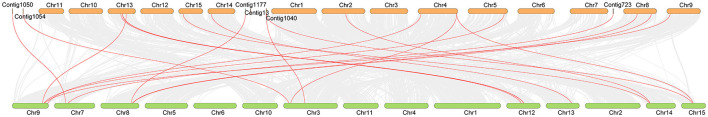
The syntenic relationship of OVATE gene between cultivated tea tree and ancient tea tree. The red lines highlight the syntenic OVATE gene pairs. Orange and green represent “ShuChaZao” and “DASZ” genomes, respectively.

Many studies have proved that there are obviously different evolutionary characteristics between cultivated tea plants and ancient tea plants. To further investigate the collinearity of the OVATE gene family in cultivated and ancient tea plants, we constructed syntenic maps between the two genomes by TBtools (Figure 4). A total of 20 gene pairs were identified between the two genomes, in which two *CsOVATE* gene family members were found to have syntenic relationships with two *CaOVATE* gene family members, respectively. Notably, no syntenic gene pairs were found for the eight *CsOVATE* genes (Supplementary Table S3).

### Expression profile analysis of OVATE genes in various tissues and validation by RT-qPCR

**Figure 5:**
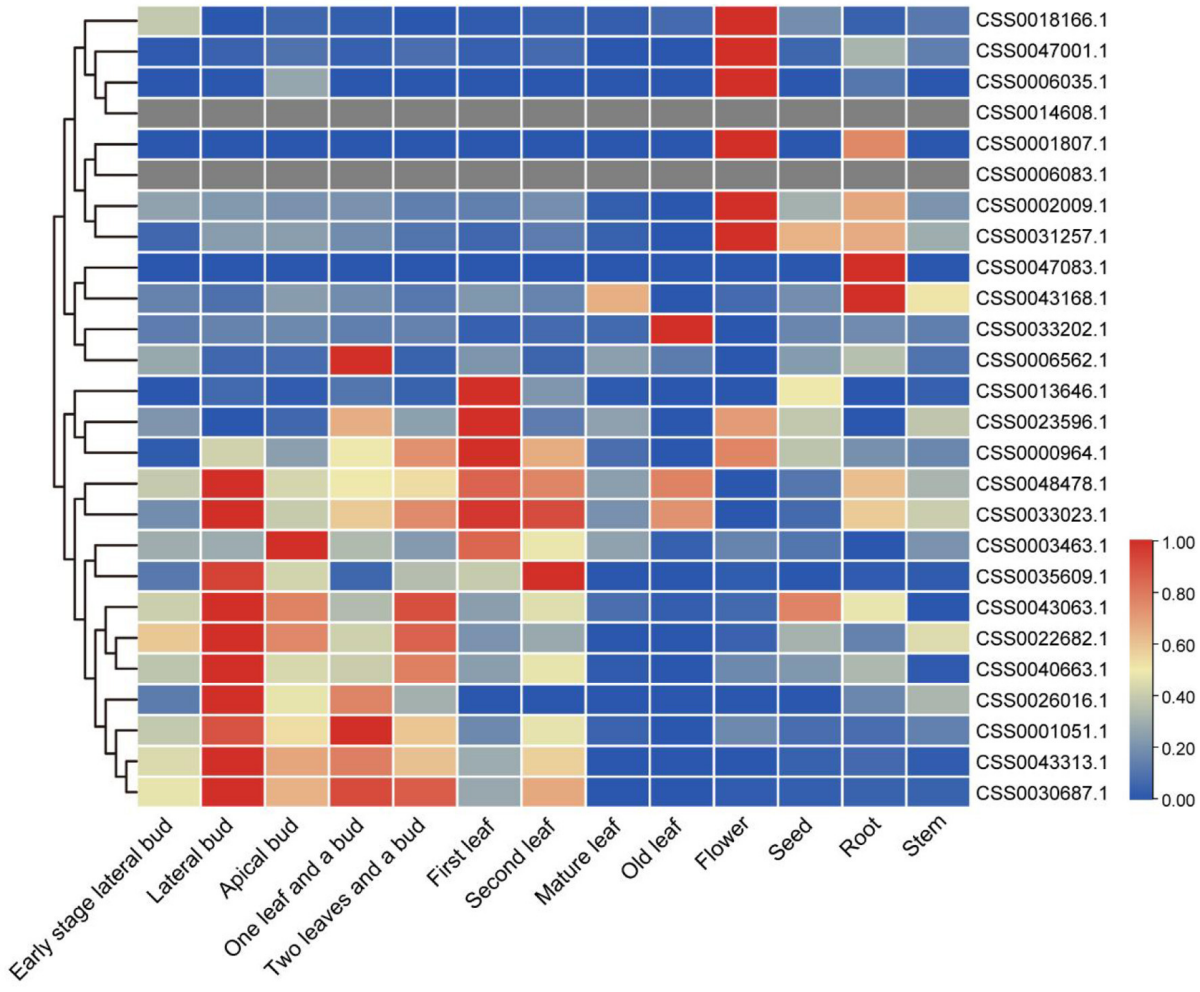
Expression analysis of OVATE genes in different tea plant tissues. The red color indicates high expression and the blue color indicates low expression.

Transcriptome analysis of different tea tissues provides support for exploring the expression level of OVATE gene from a global perspective. The analysis found that 26 OVATE family genes showed diverse expression trends in tea plants. As shown in Figure 5, six genes including CSS0018166.1, CSS0047001.1, CSS0006035.1, CSS0001807.1, CSS0002009.1, and CSS0031257.1 are highly expressed in flowers, while CSS0047083.1 and CSS0043168.1 are highly expressed in roots. In addition, 15 genes with high expression in buds and leaves were identified.

**Figure 6:**
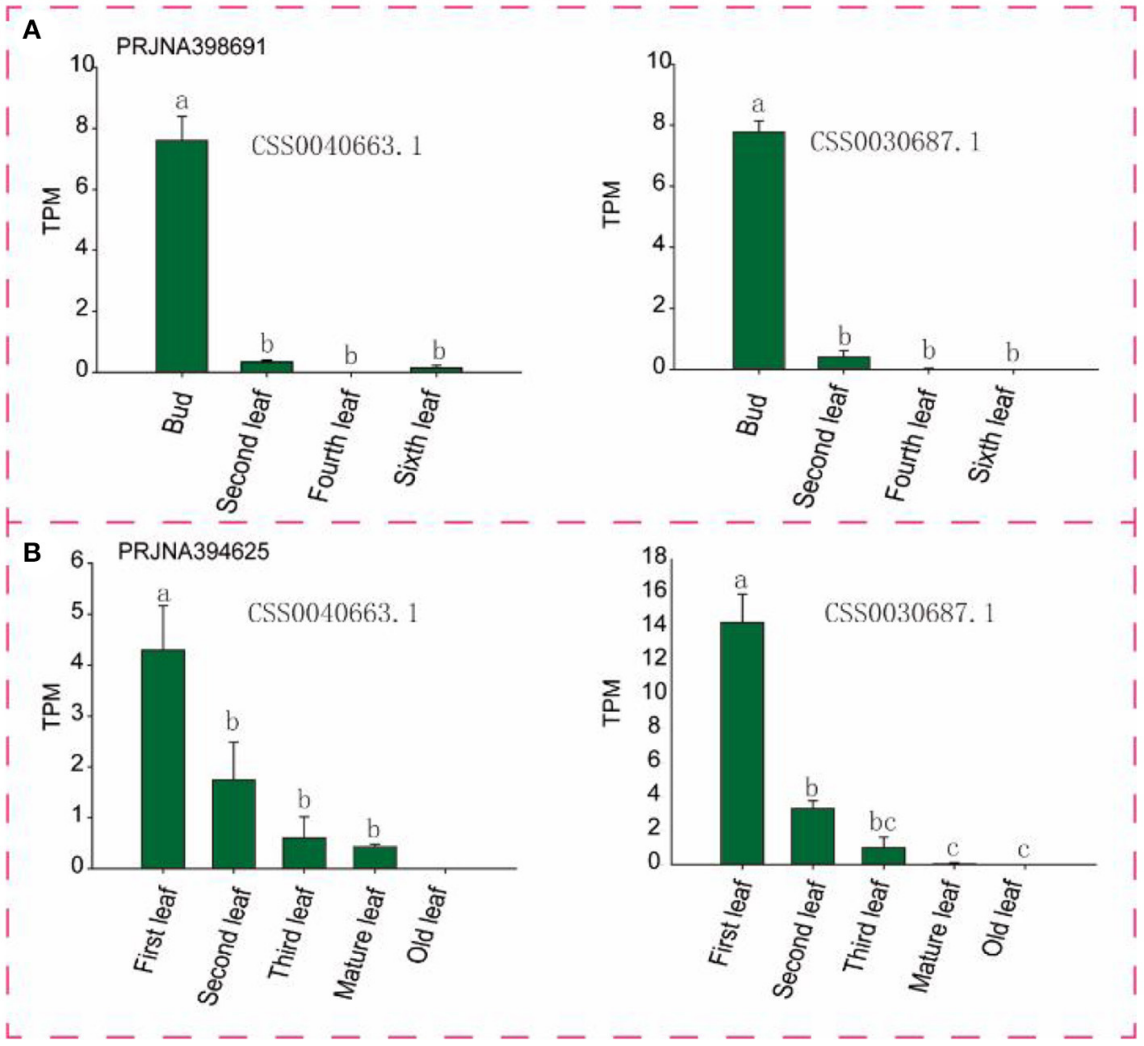
Expression levels of two candidate genes in different transcriptome and different tissues. **(A,B)** Represent different transcriptome data sources, respectively. TPM, Transcripts Per Kilobase of exon model per Million mapped reads. Different letters indicate significant differences (P < 0.05).

To identify candidate genes closely related to tea leaves, we performed expression level analysis on 63 transcriptome data. For example, as shown in Figure 6 and Supplementary Figure S2, with the development of leaves, the expression levels of CSS0040663.1 and CSS0030687.1 in multiple transcriptome data show a downward trend, and almost no expression is found in mature leaves. Finally, six genes (CSS0026016.1, CSS0043313.1, CSS0048478.1, CSS0006562.1, CSS0040663.1, and CSS0030687.1) were screened out as candidate genes for regulating the development of tea leaves (Supplementary Table S4). Interestingly, no syntenic gene pairs were found for the four candidate genes in the ancient tea plant genome.

**Figure 7:**
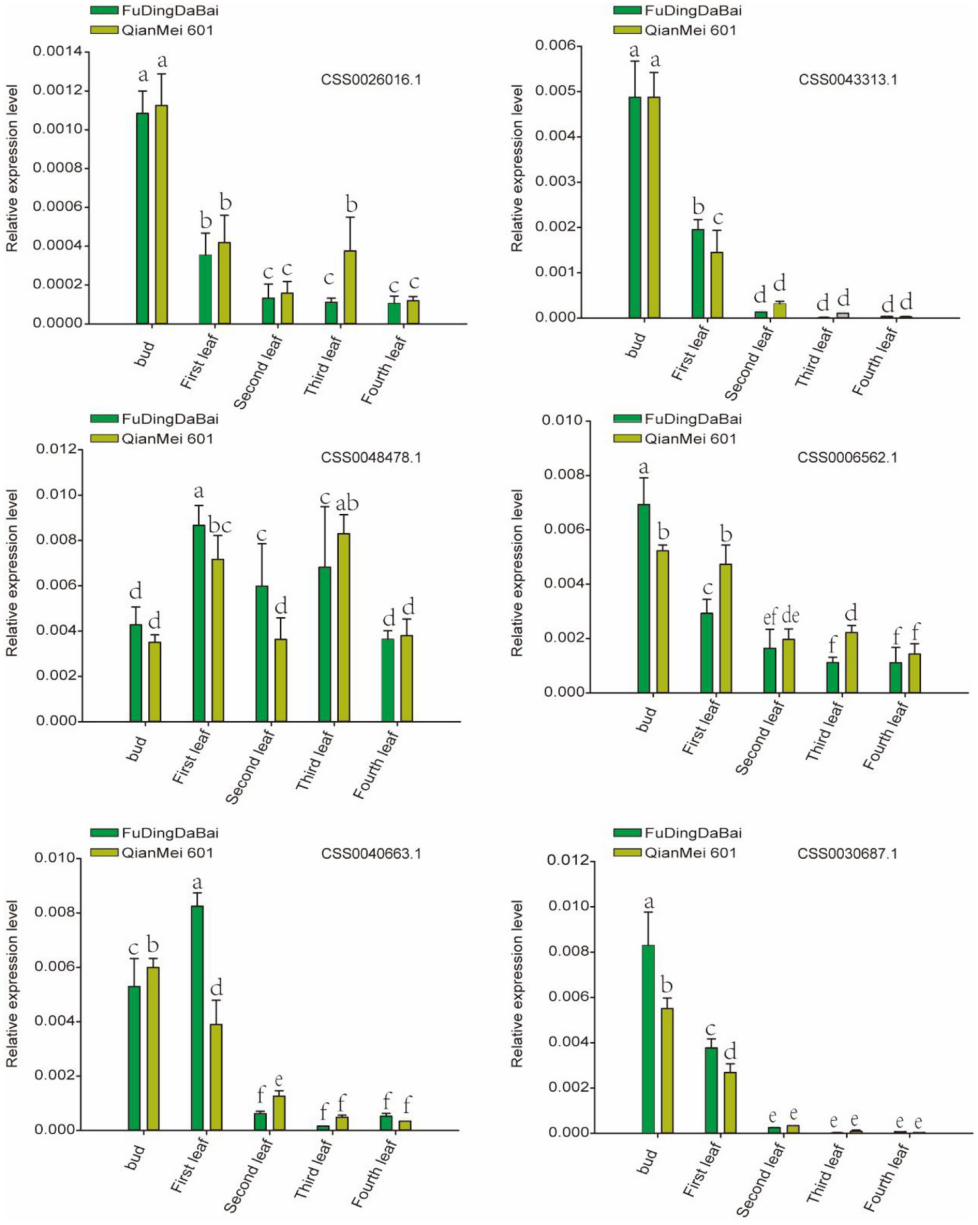
RT-qPCR verified the expression levels of six candidate genes in different tissues of “QianMei 601” and “FuDingDaBai”. Different letters indicate significant differences (P < 0.05).

In order to further verify the expression level of six candidate genes, five tissues (bud, the first, second, third, and fourth leaves) of two tea varieties were collected for RT-qPCR. As shown in Figure 7, four (CSS0026016.1, CSS0043313.1, CSS0006562.1, CSS0040663.1, and CSS0030687.1) of the six candidate genes were significantly highly expressed in buds and first leaves, their expression levels decreased to the lowest in the fourth leaf with the development of leaves. More interestingly, the expression levels of four genes (CSS0043313.1, CSS0006562.1, CSS0040663.1, and CSS0030687.1) were also significantly different in buds and leaves of “FuDingDaBai” and “QianMei 601”. The highly consistent RT-qPCR and transcriptome expression levels indicated that the OVATE gene family was closely related to tea development.

### Comparison of genotype differences between large leaf and small leaf tea plants

**Figure 8:**
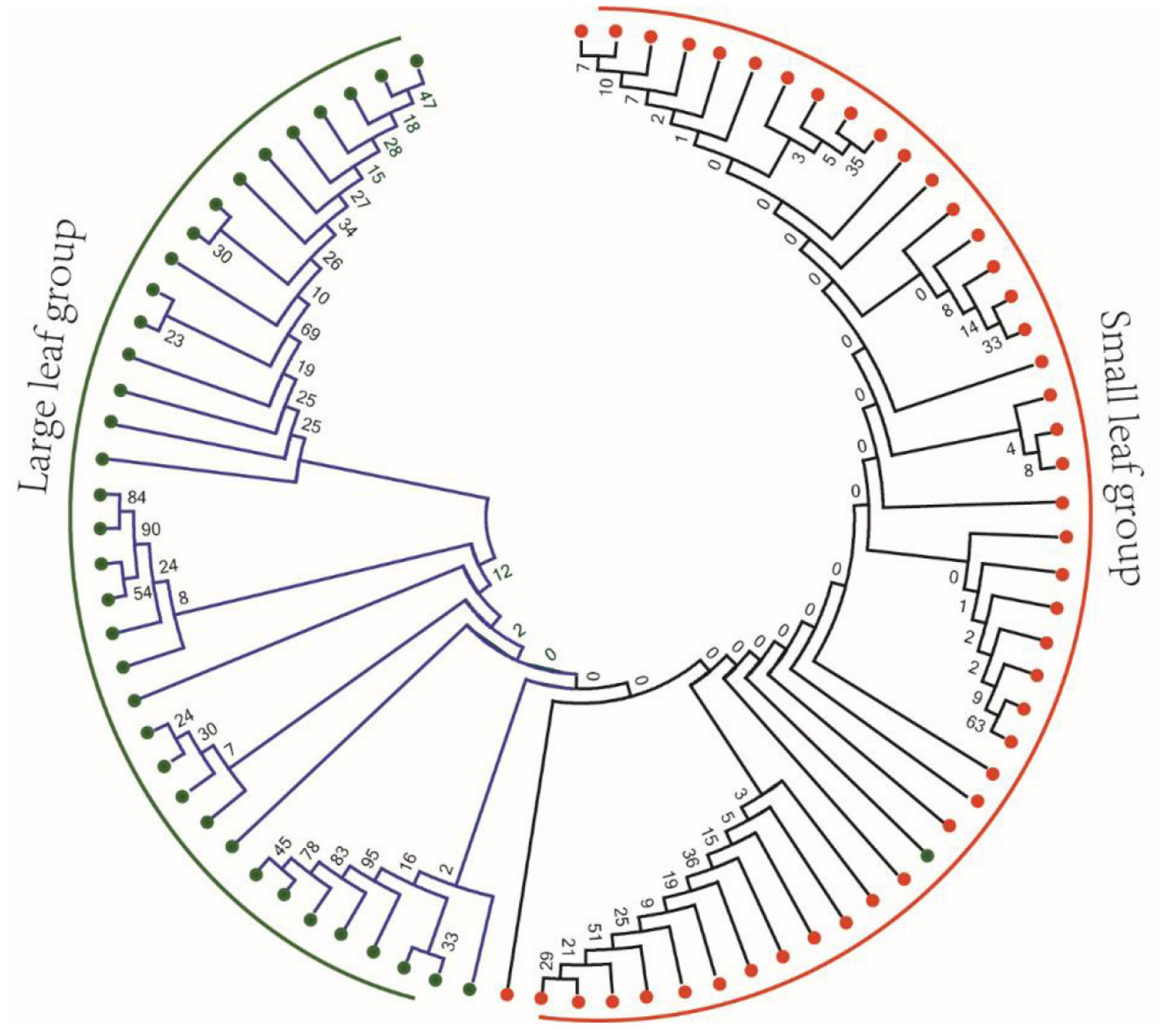
Phylogenetic analysis of 82 tea plant samples based on 69 resequenced SNP loci of OVATE family.

The difference of leaf phenotype may be caused by the difference of genotype. Therefore, we selected 82 previously published tea sequencing data to identify the mutations (Zhang et al., 2021). In the mRNA sequences of 26 OVATE genes, a total of 69 high-quality SNP mutation sites were identified. To observe whether the genotypes of these mutations have obvious population differences in large leaf and small leaf tea plants, an NJ phylogenetic tree based on 69 SNP loci was constructed. As shown in Figure 8, except for one large leaf tea variety which was classified into small leaf group, the remaining tea varieties showed good classification results. Considering the sequencing error, genotyping error and genotype loss, it can be considered that these mutation sites have obviously different allele types in large leaf and small leaf tea plants.

### Identification of a key SV mutation related to leaf development

**Figure 9:**
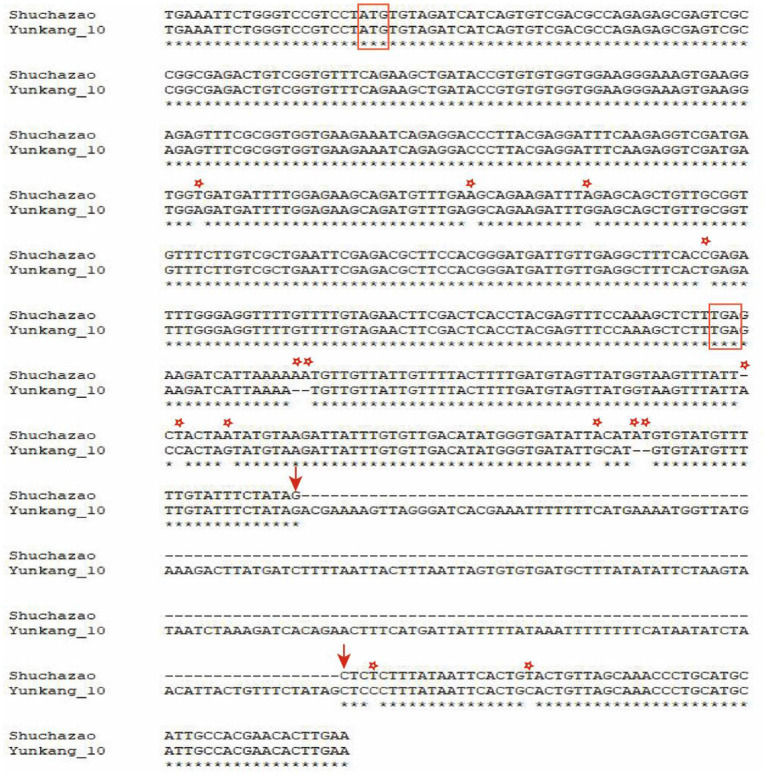
Visualize SV mutation sites by double sequence alignment. The red box represents the start codon and stop codon. Arrows indicate where SV variation begins and ends. The asterisk represents SNP and small Indel mutations.

Along with the successful publication of multiple tea plant genomes, many Pacbio sequencing data were also released at the same time (Wang et al., 2020; Zhang et al., 2021). Based on these Pacbio data, a structural variation of 184 bp in length was identified and sequenced at 135 bp downstream of the candidate gene CSS0006562.1 (Figure 9). In addition, some SNPs and small Indel mutations were identified by TA clone sequencing. Studies in peach trees have proved that structural variation downstream of OVATE gene can regulate fruit characters and leaf development (Guan et al., 2021; Zhou et al., 2021). Combined with transcriptome data and RT-qPCR, we speculate that this structural variation is also related to the leaf size of tea plants.

**Figure 10:**
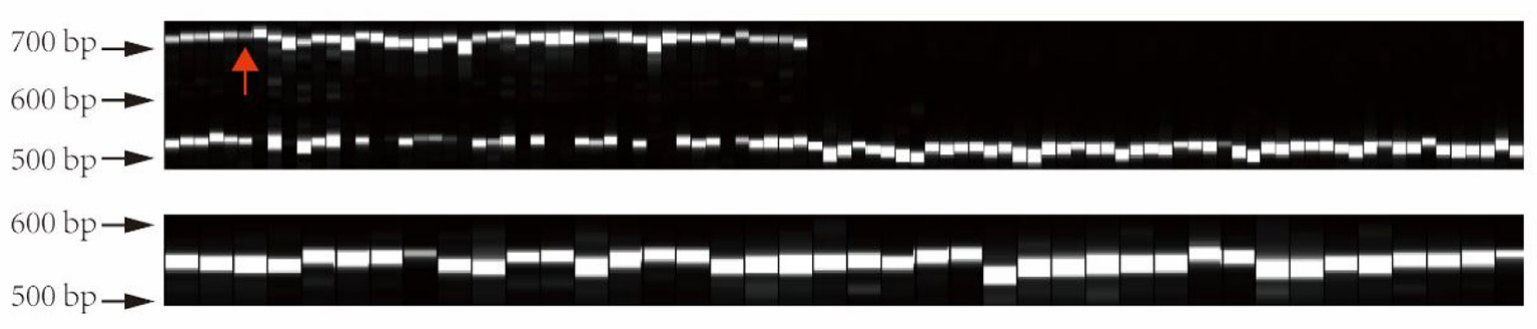
Validation of structural variant genotypes in different tea plants by capillary electrophoresis. All large-leaf tea plants have a larger length allele. The red arrow indicates the “FoShou” variety.

In order to verify the genotype of this structural variation in different tea varieties, we extracted 133 DNA samples from tea varieties, including 44 large leaf samples and 89 small leaf samples. Subsequently, the primers were designed to PCR amplify this structural variation in the above materials. PCR products were used for capillary electrophoresis analysis. The electrophoresis results are shown in Figure 10. The CSA variants all have a longer allele (about 741–746 bp), while the CSS all show a homozygous genotype (about 539–549 bp). Interestingly, as a CSS variety, “FoShou” (indicated by red arrow) has a larger leaf area than many CSA varieties, and it also has a longer-length allele (Figure 10).

## Discussion

Researchers have conducted in-depth studies on the genetic development mechanism of many plant leaves. For example, Zeng et al. (2022) found that the complex formed by *CiKN1* and *CiKN6* proteins can combine with the promoter of miR164a gene to inhibit its expression level, thus regulating the development of citrus leaves. The research results in poplar showed that *PpnGRF5-1* inhibited the expression of *PpnCKX1*, which led to the accumulation of cytokinin, and then promoted the proliferation of leaves and formed the meristematic potential of cells (Wu et al., 2021). However, there are still few studies on the leaf development mechanism of different tea germplasm. Based on the F1 population obtained from the cross of “LongJing 43” and “BaiHaoZao”, Tan et al. (2016) constructed a genetic map containing 483 SSR markers. QTL mapping was carried out for the size of mature leaves of tea trees, but limited by the quality of genetic map, no major QTL loci regulating tea development were found. Subsequently, An et al. (2021) constructed a high-density genetic map based on 96 F1 generation materials generated from the cross of “JinXuan” and “YunCha 1” through whole-genome resequencing and map integration technology. Twenty-five potential QTL loci that may be associated with leaf area were identified on chromosome 2. In addition, independent genome-wide association analysis also identified significant associated loci on chromosome 2. However, due to factors such as genome-wide linkage disequilibrium, further screening and functional verification of the mapping results are required.

Many studies have shown that OVATE gene family members are closely related to plant growth and development (Wang et al., 2007, 2011, 2016). For example, over-expression of *SlOFP20* in tomatoes can not only change the structure of plants, but also enhance the accumulation of chlorophyll and delay the senescence of leaves (Zhou et al., 2019). In this study, we identified 26 members of the OVATE gene family in the genome of the tea plant “ShuChaZao”, which is more abundant than that in *Arabidopsis* (19) but less than that in rice (31) (Yang et al., 2018). These genes are unevenly distributed on 9 chromosomes and 7 contigs (Figure 1). Compared with cultivated tea plants, ancient tea plants contain only 13 gene family members, which is roughly equivalent to the number of OVATE members in peach genome (15) (Li et al., 2019). Different from the reports in citrus (Wu et al., 2022), phylogenetic tree analysis showed that OVATE genes in tea plants were divided into four categories, of which the third group contains the most family members (Figure 2A). A total of 10 motifs were identified by sequence analysis, among which motif 3, motif 4, and motif 8 were highly conserved among different family members. However, the number of motifs contained in the same category is also different. For example, the number of motifs contained in the second category of family members ranges from 4 to 6. In addition to a large number of light-responsive elements identified in the promoter sequence, many cis-acting elements related to hormone response, stress response and growth and development were also found. This result further proves that OVATE gene family may play an important role in regulating the growth and development of tea plants (Hackbusch et al., 2005; Chen et al., 2021; Yu et al., 2021).

Tandem repeats, fragments and whole-genome duplications are important drivers of genome expansion and evolution, while also increasing the genetic diversity of plants (Yu et al., 2005). For example, analysis in citrus shows that the expansion of OVATE gene family is mainly driven by fragment duplication and tandem repeats (Wu et al., 2022). Comparative genome analysis showed that 20 OVATE gene pairs were identified between the genomes of cultivated tea plants and ancient tea plants. Interestingly, the number of OVATE family members in the genome of cultivated tea trees is twice that of ancient tea trees, which suggests that cultivated tea trees may have experienced unique expansion event (Zhang Q.-J. et al., 2020; Zhang W. et al., 2020). In particular, no gene pairs were found for the 8 *CsOVATE* genes in ancient tea plants, and transcriptome analysis showed that the expression levels of these genes were significantly correlated with the development of tea leaves.

In order to more accurately identify members of the OVATE gene family associated with tea leaf development, we performed population transcriptome analysis. The results showed that many members of the OVATE gene family of tea plant showed significantly high expression in response to biotic and abiotic stresses and in the young shoots and leaves of tea plant. Finally, 6 candidate genes were identified. The highly consistent results of RT-qPCR and population transcriptome showed that the expression levels of 5 candidate genes (CSS0026016.1, CSS0043313.1, CSS0006562.1, CSS0030687.1, and CSS0040663.1) decreased from bud to fourth leaf as tea developed. In particular, three genes located on chromosome 10 (including CSS0026016.1, CSS0030687.1, CSS0043313.1) formed a gene cluster that presented co-expression signatures in many transcriptome data sources (http://liushang.top:8025/ or http://47.106.184.91:8025/).

Obviously, similar to the research reports in other plants, our research proves that ovate gene may be involved in regulating the development of tea leaves (Tang et al., 2018; Yang et al., 2018; Zhou et al., 2019). However, it remains unknown whether it contributes to the different leaf phenotypes of large-leaf and small-leaf tea plants. A large number of studies have proved that SNP or structural variation in the genome may cause changes in plant traits and even lead to individual death. For example, SNP changes in the first exon of the *ZmTE1* gene on maize chromosome 3 lead to dwarf maize and increased leaves (Wang et al., 2022); A SNP significantly associated with spring bud flowering time was identified in tea plant by genome-wide association analysis (Wang et al., 2019). SNP-based phylogenetic tree analysis found that the ovate gene family had significant genotypic differences between large-leaf and small-leaf tea cultivars. In the exon region of CSS0006562 gene, we identified four SNPs (including one non-synonymous mutation: GTG>GAG). More importantly, a 184 bp structural variation closely related to leaf area was discovered. Sequencing results showed that this structural variation was located 135 bp downstream of the CSS0006562.1 gene (Figure 9). The genotyping analysis of 133 tea samples showed that, different from the small-leaf species, the large-leaf species all have a longer allele, which may lead to the difference in leaf traits between the two (Figure 10). These results comprehensively show that the OVATE gene family plays an important role in the growth and development of tea plants.

## Conclusion

In this study, we systematically analyzed the members of OVATE gene family in tea plants for the first time. Twenty-six and thirteen family members were identified in cultivated tea trees and ancient tea trees, respectively, and these genes were classified into four subfamilies. We not only investigated motifs and cis-acting elements, but also verified the expression signature of candidate OVATE genes at different developmental stages in tea leaves by transcriptome and RT-qPCR. At the same time, a structural variation closely related to tea leaf area was identified. In a word, our results prove that OVATE gene family plays an important role in the development of tea leaves, and identify a key structural variation related to leaf area. However, its specific regulation mechanism is worth further exploring in the future.

## Materials and methods

### Identification of the OVATE family in tea plant

First, we downloaded the whole genome protein sequence of the “ShuChaZao” (http://tpdb.shengxin.ren/index.html), “DASZ” (NCBI, PRJNA595851) and Arabidopsis (https://www.arabidopsis.org/). The Hidden Markov Model (HMM) profile of the OVATE family (PF04844.16) was retrieved from the Protein Family Database (Pfam) (http://pfam.sanger.ac.uk/) website. Hmmsearch software was used to identify OVATE gene in genome (hmmsearch –cut_tc –domtblout output hmm.model Genome.pep.fa), and the sequence with *e*-value < 1e^−20^ was retainer. Subsequently, all candidate OVATE proteins were examined by SMARAT (http://smart.embl-heidelberg.de/) for the presence of OFP domains (Huang et al., 2022). The MW and *pI* values of OVATE family proteins were predicted using the ExPasy website (https://www.expasy.org/). Cis-acting elements were predicted by PlantCARE online software (https://bioinformatics.psb.ugent.be/webtools/plantcare/html/).

### Phylogenetic analysis and motif identification

All protein sequences were aligned by ClustalW program built in MEGA 7, and then NJ phylogenetic tree was constructed based on poisson model with a bootstrap value of 1,000. And all protein sequence motif predictions were performed by MEME (http://meme-suite.org/) (Lu et al., 2021).

### Chromosomal distribution, gene duplication, and synteny analysis

After obtaining the location information of OVATE gene family based on gff file of “ShuChaZao” genome, MG2C (http://mg2c.iask.in/mg2c_v2.1/) was used to visualize the distribution of these genes on chromosomes. Gene duplication and synteny analysis were completed and visualized by using MCSCANX (Multiple Collinearities Scan Toolkit) built in TBtools. In addition, the heat maps of transcriptome expression levels in different tissues were also visualized by TBtools (Chen et al., 2020).

### RNA extraction and RT-qPCR validation

To verify the expression level of candidate OVATE genes in tea plants, the buds, first, second, third, and fourth leaves of “QianMei 601” and “FuDingDaBai” were collected from the Tea Tree Resource Garden of Guizhou Academy of Agricultural Sciences. All samples were quickly frozen in liquid nitrogen immediately after picking and stored in a refrigerator at −80°C until total RNA was extracted. Total RNA purification kit (Norgen Biotek Corporation, Canada) was used to extract total RNA from all samples according to the protocol provided by the manufacturer. Then, the qualified total RNA was purified and reverse transcribed into cNDA using Prime Script RT Master Mix (Takara, Japan). The GAPDH gene was selected as an internal control, and the relative gene expression values were calculated by the 2^−Δ*ΔCt*^ method (Li et al., 2020). The detailed RT-qPCR amplification procedure and parameters refer to previous studies (Zhao et al., 2018). Each sample contains two biological replicates and eight technical replicates. Primer information is listed in Supplementary Table S5.

### Identification of SNPs and structural variations

In order to identify SNP and structural variation, the original sequencing data were obtained from GSA database (PRJCA003090) and NCBI database (PRJNA595851). Pacbio sequencing data was aligned to the “ShuChaZao” genome using minimap2 (Li, 2018), and then the structural variation was identified by cuteSV software (Jiang et al., 2020). The alignment between Illumina resequencing data and reference genome and SNP identification were carried out by bwa and GATK software respectively (Zhang et al., 2021). The conversion of all data formats was done using samtools software. The NJ phylogenetic tree is constructed by MEGA, and the bootstrap value is 1,000.

### DNA extraction and validation of SV variation in different tea varieties

A total of 133 tea samples were collected from the Tea Resource Garden of Guizhou Academy of Agricultural Sciences. All the young leaves of the samples were picked and quickly frozen in liquid nitrogen, and then stored in a −80°C freezer until DNA extraction. The total DNA was extracted according to the previous research of Liu et al. (2018). Qualified DNA was diluted to 50–60 ng/μL for the key SV variant amplification. Detailed PCR amplification conditions were carried out with reference to previous studies (Liu et al., 2017). The sequence of specific primer pairs used for amplification is as follows: TGAAATTCTGGGTCCGTCCTA (forward primer); TTCAAGTGTTCGTGGCAATGC (reverse primer). The 96-capillary automated DNA fragment analyzer (fragment analyzer 96, advanced analytical technologies, Inc., Ames, ia) was used for electrophoresis separation of PCR amplified fragments (Liu et al., 2018). The results of capillary electrophoresis were visualized by PROSize™ 2.0 software (Liu et al., 2019).

## Data availability statement

The original contributions presented in the study are included in the article/Supplementary material, further inquiries can be directed to the corresponding author/s.

## Author contributions

FZ and TJ conceived and designed the research project. YA and XX performed sample collection, DNA and RNA extraction, and capillary electrophoresis analysis. YA undertook gene family identification, transcriptome analysis, and RT-qPCR validation. All authors have read and agreed to the published version of the manuscript.

## Funding

This research was funded by Zunyi Technology and Big Data Bureau, Moutai Institute Joint Science and Technology Research and Development Project (ZSKHHZ [2022] No. 166), the National Natural Science Foundation of China (32160441), and the Special Food Resources Utilization Talent Base of Moutai Institute.

## Conflict of interest

The authors declare that the research was conducted in the absence o any commercial or financial relationships that could be construed as a potential conflict of interest.

## Publisher's note

All claims expressed in this article are solely those of the authors and do not necessarily represent those of their affiliated organizations, or those of the publisher, the editors and the reviewers. Any product that may be evaluated in this article, or claim that may be made by its manufacturer, is not guaranteed or endorsed by the publisher.

## Supplementary material

Supplementary Table S1 Details of the pl, MW and subcellular localization of members of the ovate gene family in the genomes of cultivated and ancient tea plants.

Supplementary Table S2 Detailed information on cis-acting elements identified in the genomes of cultivated and ancient tea plants.

Supplementary Table S3 Gene pair information of the OVATE gene family in “ShuChaZao” and “DASZ” genomes.

Supplementary Table S4 Expression levels of six candidate genes in four different transcriptome sources (TPM).

Supplementary Table S5 Quantitative primer sequences used in this study.

Supplementary Figure S1 Gene structure of OVATE gene family.

Supplementary Figure S2 Expression levels of two candidate genes in different transcriptome and different tissues. **(A,B)** Represent different transcriptome data sources, respectively. TPM, Transcripts Per Kilobase of exon model per Million mapped reads.
