# Supplementary figures and images for: Identification of gene family members and a key structural variation reveal important roles of OVATE genes in regulating tea (Camellia sinensis) leaf development
Source: Front Plant Sci. 2022 Sep 23;13:1008408. doi: 10.3389/fpls.2022.1008408 (PMC9539550; doi:10.3389/fpls.2022.1008408)

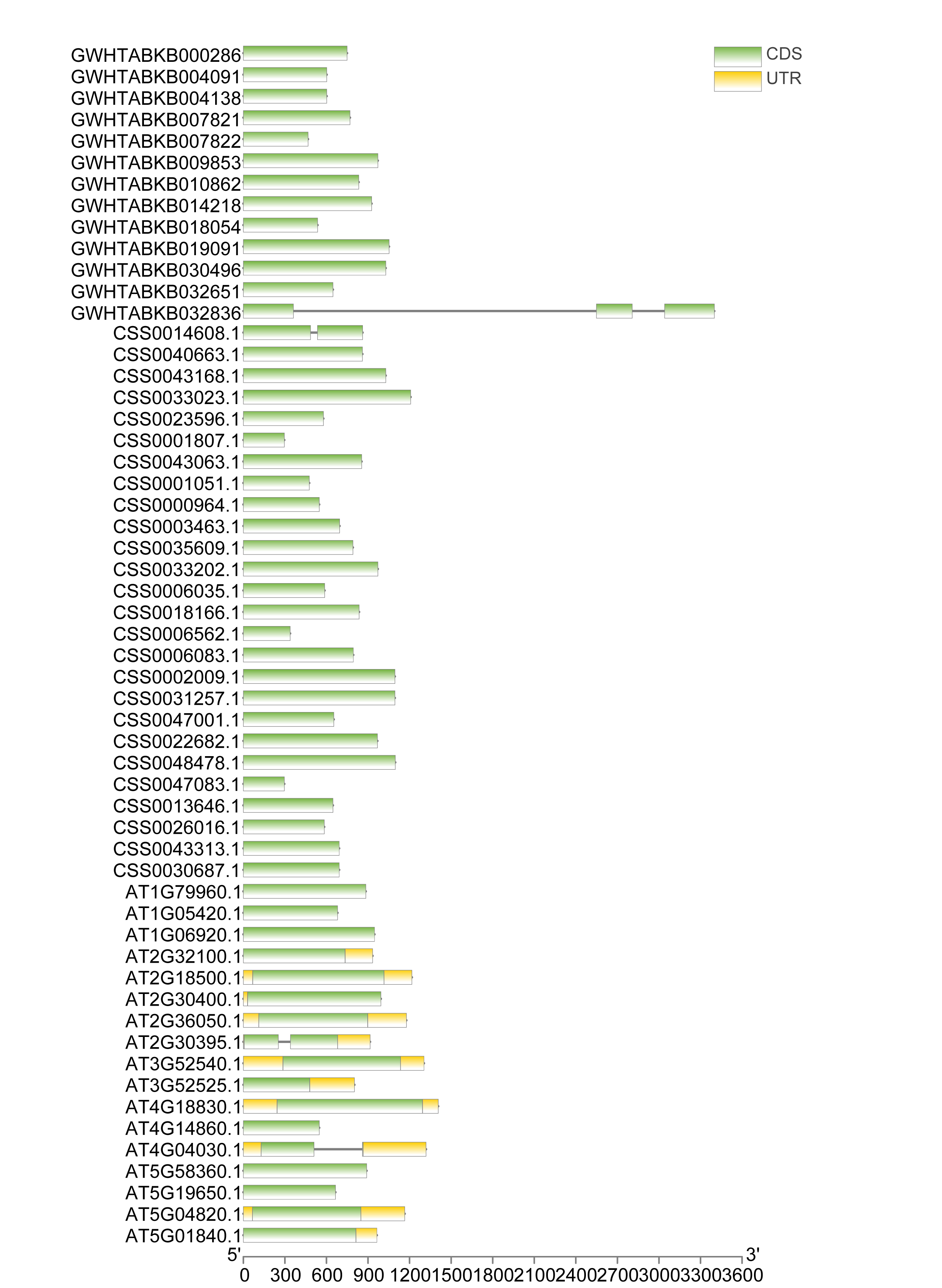

Supplement: Supplementary Figure S1 — Gene structure of OVATE gene family. [file Image_1.TIF]

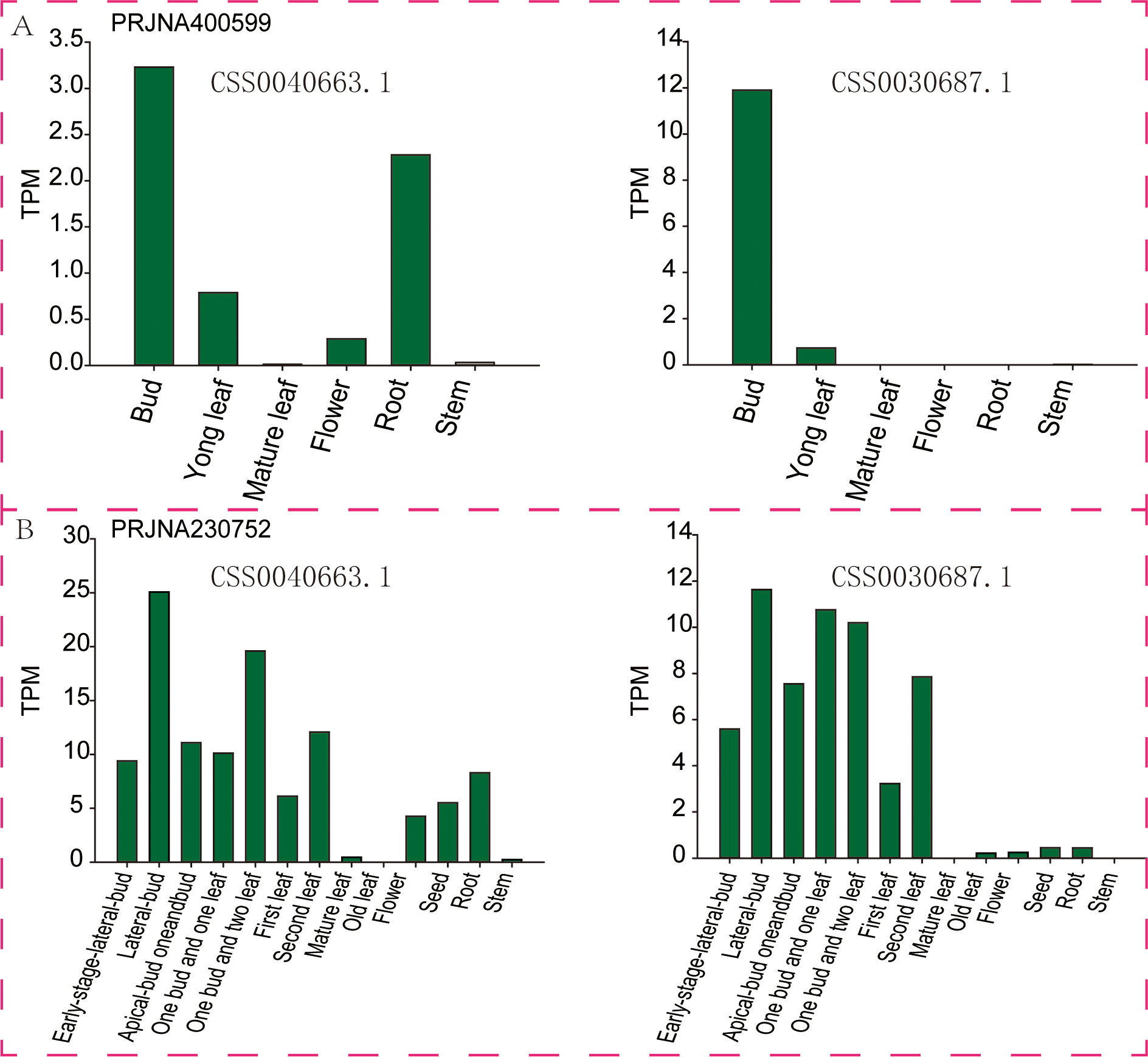

Supplement: Supplementary Figure S2 — Expression levels of two candidate genes in different transcriptome and different tissues. (A,B) Represent different transcriptome data sources, respectively. TPM, Transcripts Per Kilobase of exon model per Million mapped reads. [file Image_2.TIF]
