# Supplementary material for: Identification of gene family members and a key structural variation reveal important roles of OVATE genes in regulating tea (Camellia sinensis) leaf development
Source: Front Plant Sci. 2022 Sep 23;13:1008408. doi: 10.3389/fpls.2022.1008408 (PMC9539550; doi:10.3389/fpls.2022.1008408)
Supplement: Supplementary file 8 [file Data_Sheet_1.PDF]

Fragment Analyzer Run Summary:

Analysis Mode: DNA

Copyright 2015 Advanced Analytical Technologies, Inc.

Gel Image

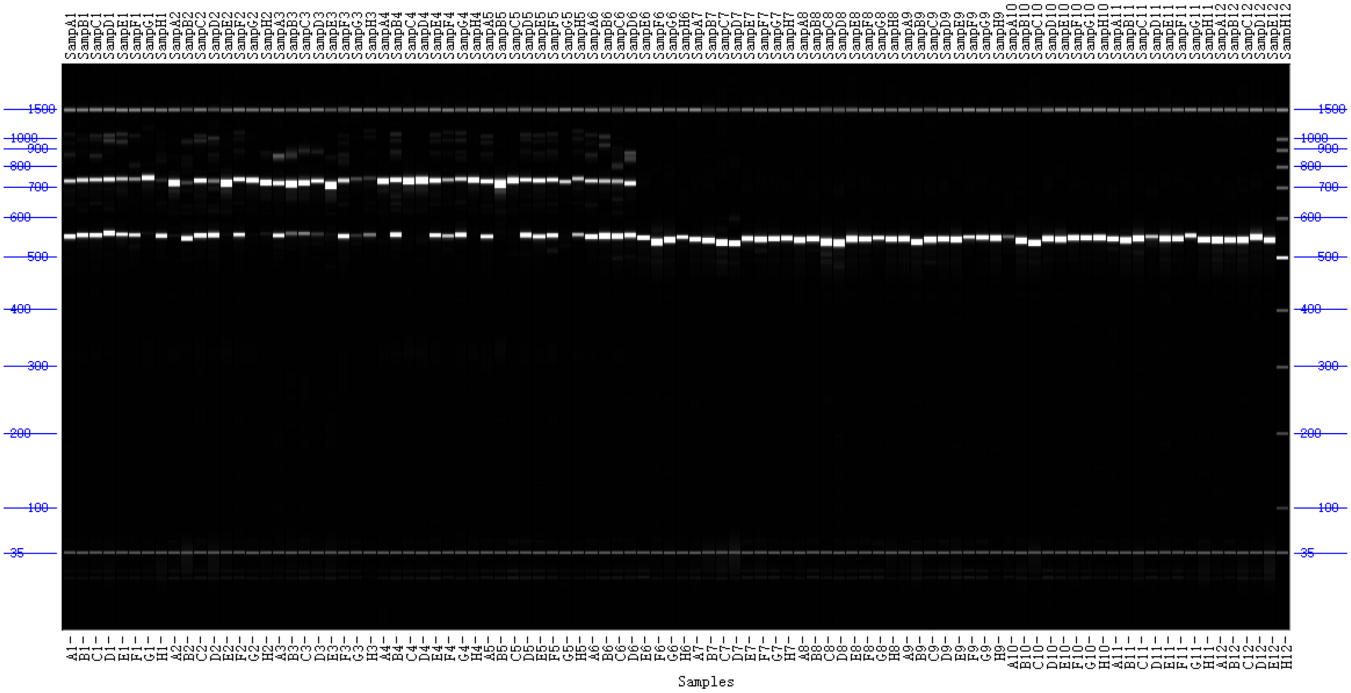

Copyright 2015 Advanced Analytical Technologies, Inc.

Sample: SampA1
Well Location: A1

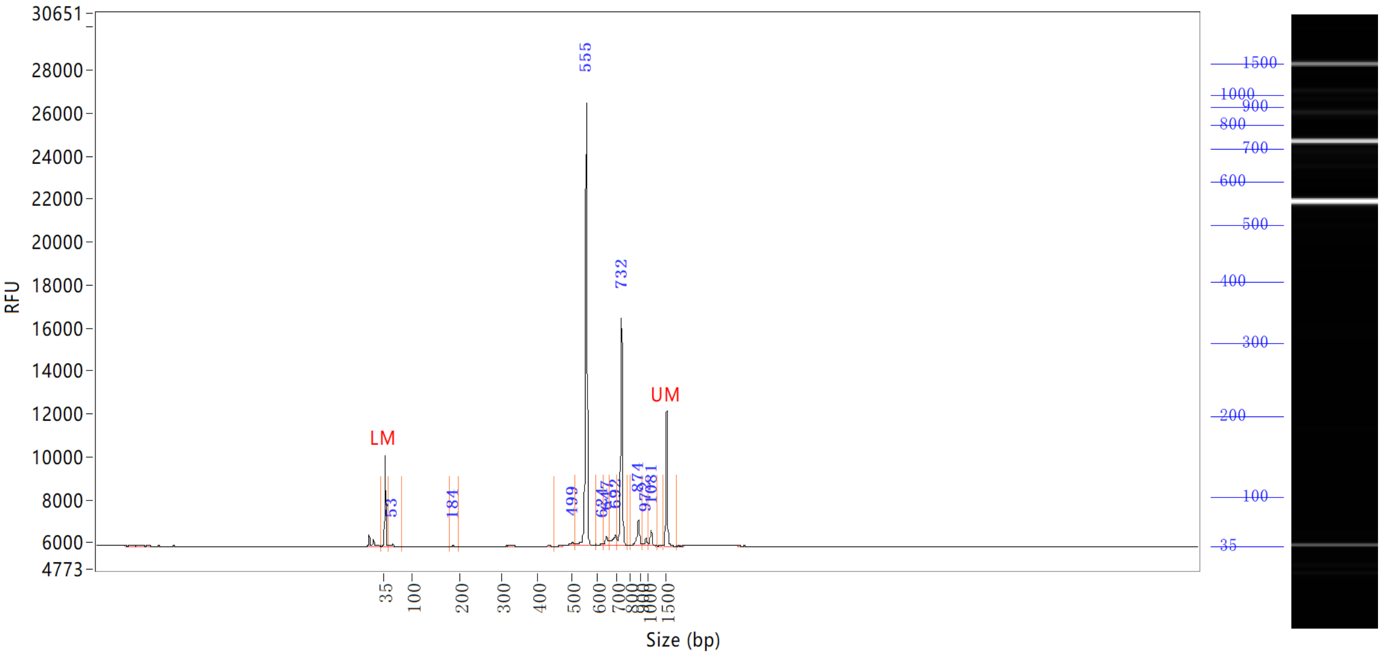

Peak	Size (bp)	Conc. (ng/uL)	From (bp)	To (bp)	Avg. Size (bp)	CV%	RFU	Corr. Peak Area
1	35 (LM)	0.4539	26	43	35	4.75	4240	19.590
2	53	0.4104	43	76	52	10.87	113	1.476
3	184	0.1382	177	198	185	2.51	52	0.497
4	499	0.5965	447	511	488	2.89	132	2.145
5	555	24.1756	511	597	553	1.02	20629	86.946
6	624	0.1533	597	630	620	1.14	82	0.551
7	647	0.8413	630	664	648	1.30	425	3.026
8	692	1.3523	664	706	686	1.60	505	4.864
9	732	11.7228	706	776	731	0.96	10621	42.160
10	874	1.9481	806	926	870	2.03	1234	7.006
11	972	0.4853	926	1000	965	1.81	343	1.745
12	1081	0.9045	1000	1267	1068	3.07	738	3.253
13	1500 (UM)	0.5000	1407	1770	1494	1.77	6326	21.579

TIC:	42.7283	ng/uL
TIM:	126.1964	nmole/L
Total Conc.:	43.5447	ng/uL

```
Sample Peak Width (sec): 5    Sample Min Peak Height: 50    Sample Baseline V to V?: Y    Sample Baseline V to V pts: 3
Sample Filter: Binomial       # of Pts for Filter: 3        Sample Start Region (min): 0  Sample End Region (min): 75
Marker Peak Width (sec): 5    Marker Min Peak Height: 500   Marker Baseline V to V?: Y    Marker Baseline V to V pts: 3
Lower Marker Selection: First Peak > 500 RFU                Upper Marker Selection: Last Peak > 500 RFU
Ladder Size (bp)35, 100, 200, 300, 400, 500, 600, 700, 800, 900, 1000, 1500
Quantification Using: Upper Marker         Final Concentration (ng/uL): 0.5000         Dilution Factor: 12.0
```

 Copyright 2015 Advanced Analytical Technologies, Inc.

Sample: SampB1
Well Location: B1

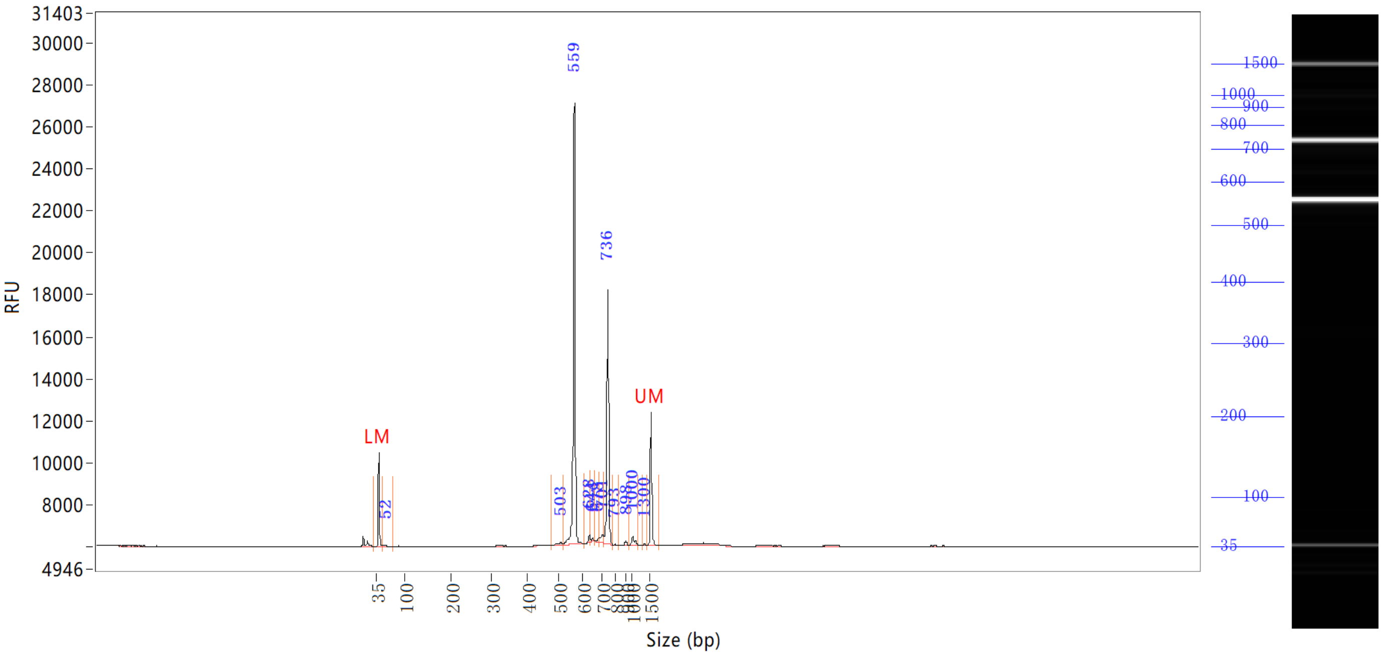

Peak	Size (bp)	Conc. (ng/uL)	From (bp)	To (bp)	Avg. Size (bp)	CV%	RFU	Corr. Peak Area
1	35 (LM)	0.4896	25	45	35	5.65	4451	21.451
2	52	0.3686	45	69	53	11.18	84	1.346
3	503	0.3658	477	516	497	2.00	129	1.336
4	559	25.7206	516	599	557	1.14	21038	93.911
5	628	0.4003	599	637	626	0.81	325	1.462
6	644	0.1977	637	658	644	0.63	184	0.722
7	678	0.2622	658	682	675	0.80	193	0.957
8	701	0.7952	682	710	697	1.10	398	2.903
9	736	13.8626	710	778	735	1.04	12124	50.615
10	793	0.0546	778	832	792	1.20	51	0.199
11	898	0.4016	832	943	892	2.42	221	1.467
12	1000	0.8199	943	1147	1013	3.85	446	2.994
13	1300	0.0944	1260	1414	1325	3.07	51	0.345
14	1500 (UM)	0.5000	1414	1724	1494	1.78	6374	21.907

TIC:	43.3435	ng/uL
TIM:	126.0113	nmole/L
Total Conc.:	44.3987	ng/uL

Sample Peak Width (sec): 5   Sample Min Peak Height: 50   Sample Baseline V to V?: Y   Sample Baseline V to V pts: 3
Sample Filter: Binomial   # of Pts for Filter: 3   Sample Start Region (min): 0   Sample End Region (min): 75
Marker Peak Width (sec): 5   Marker Min Peak Height: 500   Marker Baseline V to V?: Y   Marker Baseline V to V pts: 3
Lower Marker Selection: First Peak > 500 RFU   Upper Marker Selection: Last Peak > 500 RFU
Ladder Size (bp)35, 100, 200, 300, 400, 500, 600, 700, 800, 900, 1000, 1500
Quantification Using: Upper Marker   Final Concentration (ng/uL): 0.5000   Dilution Factor: 12.0

 Copyright 2015 Advanced Analytical Technologies, Inc.

Sample: SampC1
Well Location: C1

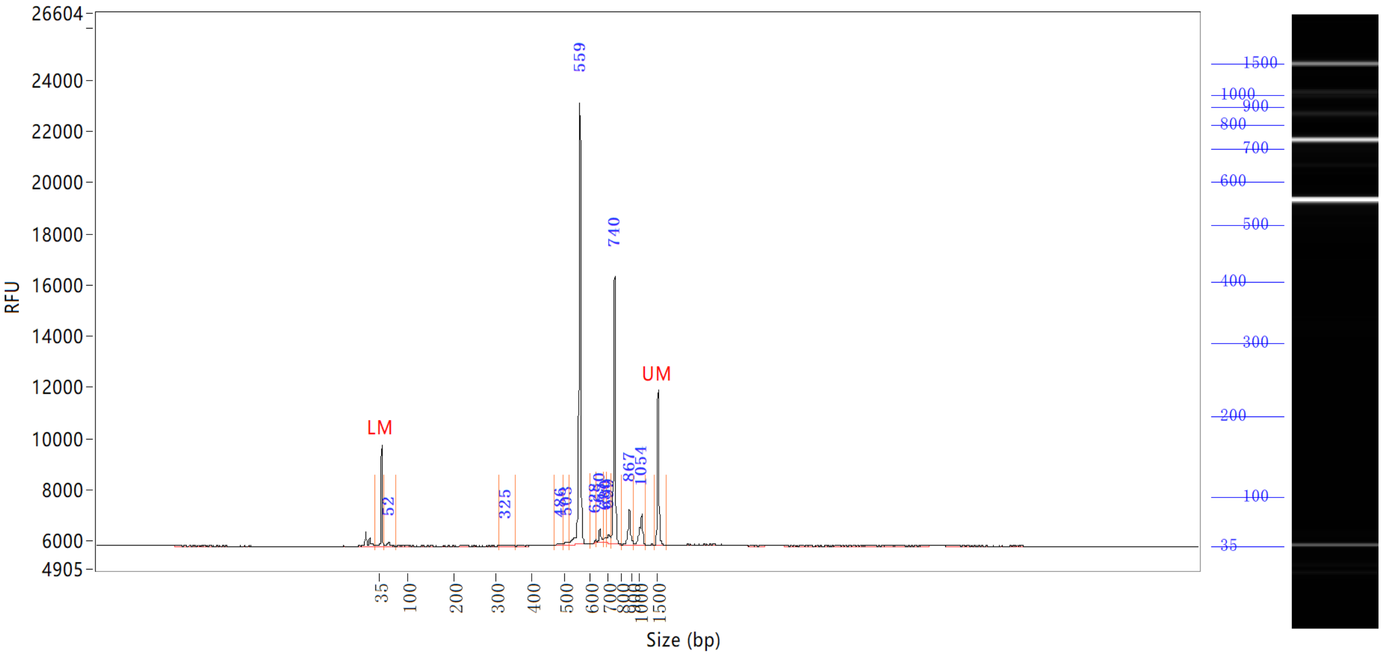

Peak	Size (bp)	Conc. (ng/uL)	From (bp)	To (bp)	Avg. Size (bp)	CV%	RFU	Corr. Peak Area
1	35 (LM)	0.4533	24	44	35	5.76	3938	18.998
2	52	0.4415	44	71	51	9.14	127	1.542
3	325	0.3169	308	352	326	3.19	51	1.107
4	486	0.2282	466	493	481	1.52	54	0.797
5	503	0.3518	493	518	506	1.36	118	1.228
6	559	21.9510	518	600	556	1.18	17257	76.658
7	628	0.0858	600	634	626	0.77	93	0.300
8	650	0.7818	634	671	653	1.24	514	2.730
9	680	0.3218	671	686	679	0.67	208	1.124
10	699	0.6683	686	712	698	1.03	322	2.334
11	740	12.1931	712	800	737	1.05	10453	42.581
12	867	2.1798	800	919	864	1.95	1394	7.612
13	1054	2.3271	919	1181	1021	3.82	1236	8.127
14	1500 (UM)	0.5000	1427	1750	1498	1.85	6055	20.953

TIC:	41.8471	ng/uL
TIM:	122.3565	nmole/L
Total Conc.:	42.7164	ng/uL

Sample Peak Width (sec): 5 Sample Min Peak Height: 50 Sample Baseline V to V?: Y Sample Baseline V to V pts: 3
Sample Filter: Binomial # of Pts for Filter: 3 Sample Start Region (min): 0 Sample End Region (min): 75
Marker Peak Width (sec): 5 Marker Min Peak Height: 500 Marker Baseline V to V?: Y Marker Baseline V to V pts: 3
Lower Marker Selection: First Peak > 500 RFU Upper Marker Selection: Last Peak > 500 RFU
Ladder Size (bp)35, 100, 200, 300, 400, 500, 600, 700, 800, 900, 1000, 1500
Quantification Using: Upper Marker Final Concentration (ng/uL): 0.5000 Dilution Factor: 12.0

 Copyright 2015 Advanced Analytical Technologies, Inc.

Sample: SampD1
Well Location: D1

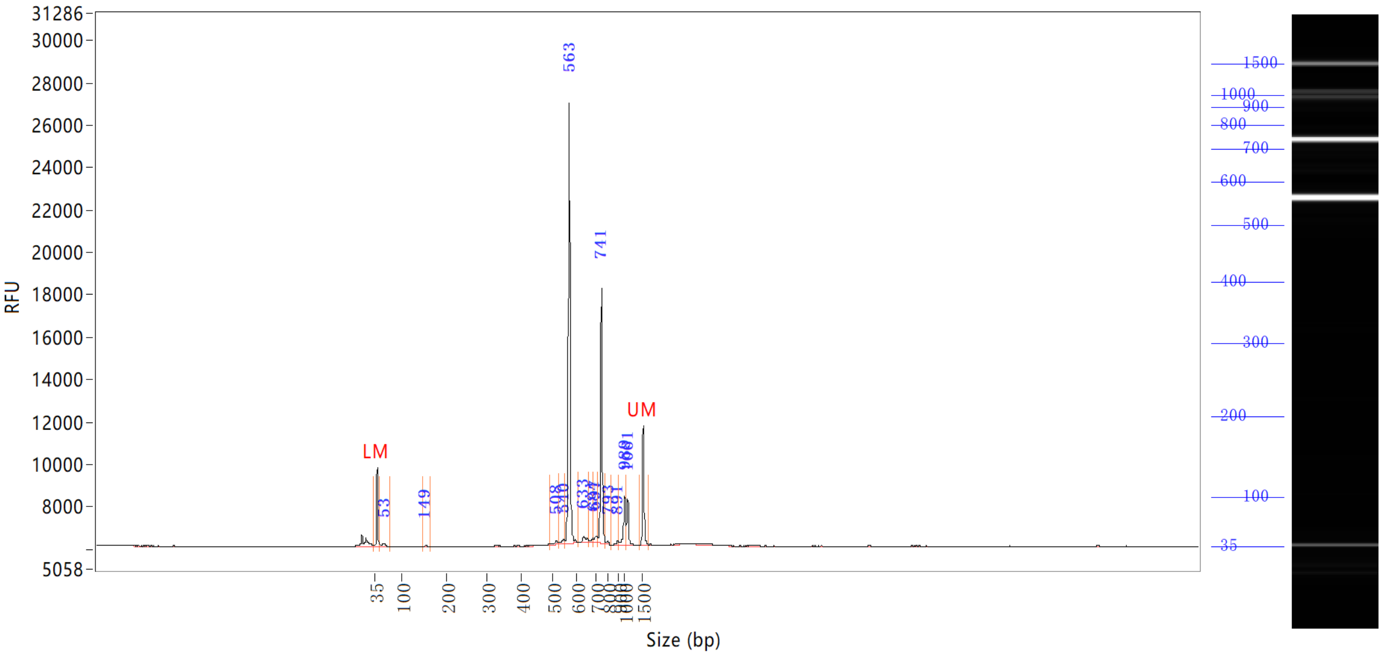

Peak	Size (bp)	Conc. (ng/uL)	From (bp)	To (bp)	Avg. Size (bp)	CV%	RFU	Corr. Peak Area
1	35 (LM)	0.4545	30	45	35	5.89	3720	18.239
2	53	0.4871	45	67	51	8.61	124	1.629
3	149	0.1107	145	160	150	1.86	60	0.370
4	508	0.4746	486	521	504	1.82	142	1.587
5	540	0.5021	521	545	535	1.23	193	1.679
6	563	27.7356	545	604	563	0.89	20812	92.750
7	633	0.8661	604	663	639	1.78	328	2.896
8	684	0.4357	663	689	678	1.05	202	1.457
9	697	0.6346	689	714	700	1.03	310	2.122
10	741	15.1072	714	776	739	1.07	12028	50.520
11	793	0.2266	776	822	789	0.88	154	0.758
12	891	0.3759	822	912	884	1.99	192	1.257
13	989	3.3476	912	1014	980	2.04	2306	11.195
14	1061	2.8230	1014	1407	1062	4.41	2198	9.440
15	1500 (UM)	0.5000	1407	1665	1501	1.83	5665	20.064

TIC:	53.1267	ng/uL
TIM:	150.6146	nmole/L
Total Conc.:	54.1305	ng/uL

```
Sample Peak Width (sec): 5     Sample Min Peak Height: 50    Sample Baseline V to V?: Y     Sample Baseline V to V pts: 3
Sample Filter: Binomial        # of Pts for Filter: 3        Sample Start Region (min): 0  Sample End Region (min): 75
Marker Peak Width (sec): 5     Marker Min Peak Height: 500   Marker Baseline V to V?: Y     Marker Baseline V to V pts: 3
Lower Marker Selection: First Peak > 500 RFU                 Upper Marker Selection: Last Peak > 500 RFU
Ladder Size (bp)35, 100, 200, 300, 400, 500, 600, 700, 800, 900, 1000, 1500
Quantification Using: Upper Marker          Final Concentration (ng/uL): 0.5000          Dilution Factor: 12.0
```

 Copyright 2015 Advanced Analytical Technologies, Inc.

Sample: SampE1
Well Location: E1

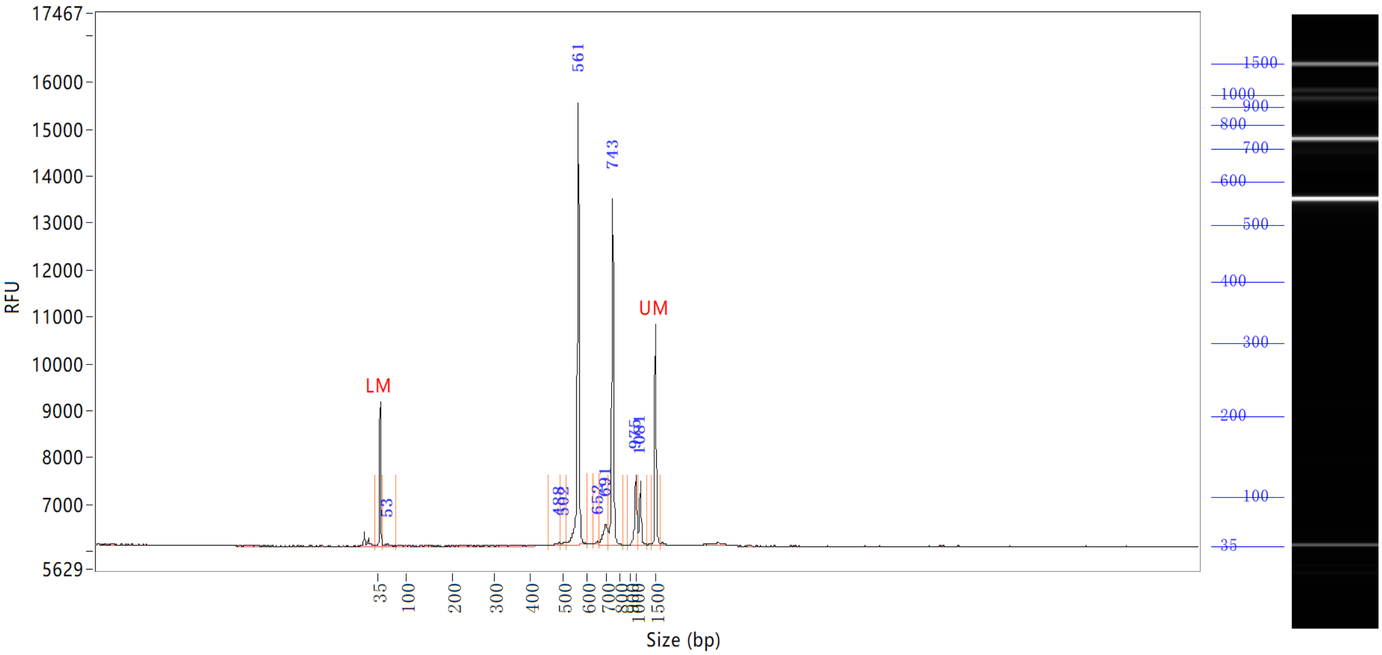

Peak	Size (bp)	Conc. (ng/uL)	From (bp)	To (bp)	Avg. Size (bp)	CV%	RFU	Corr. Peak Area
1	35 (LM)	0.4557	24	43	35	5.26	3083	14.859
2	53	0.2983	43	73	52	10.71	53	0.811
3	488	0.2514	454	493	480	1.96	53	0.683
4	502	0.2183	493	511	502	0.99	69	0.593
5	561	16.0534	511	600	558	1.45	9420	43.624
6	652	0.1825	631	659	647	1.14	66	0.496
7	691	1.8673	659	716	691	1.87	449	5.074
8	743	11.5470	716	827	741	1.27	7388	31.378
9	975	2.6529	876	1021	971	2.25	1483	7.209
10	1081	2.1007	1021	1254	1076	2.75	1359	5.709
11	1500 (UM)	0.5000	1380	1638	1492	1.77	4718	16.305

TIC:	35.1719	ng/uL
TIM:	96.5862	nmole/L
Total Conc.:	36.2856	ng/uL

```
Sample Peak Width (sec): 5    Sample Min Peak Height: 50    Sample Baseline V to V?: Y    Sample Baseline V to V pts: 3
Sample Filter: Binomial       # of Pts for Filter: 3        Sample Start Region (min): 0  Sample End Region (min): 75
Marker Peak Width (sec): 5    Marker Min Peak Height: 500   Marker Baseline V to V?: Y    Marker Baseline V to V pts: 3
Lower Marker Selection: First Peak > 500 RFU                Upper Marker Selection: Last Peak > 500 RFU
Ladder Size (bp)35, 100, 200, 300, 400, 500, 600, 700, 800, 900, 1000, 1500
Quantification Using: Upper Marker          Final Concentration (ng/uL): 0.5000          Dilution Factor: 12.0
```

 Copyright 2015 Advanced Analytical Technologies, Inc.

Sample: SampF1
Well Location: F1

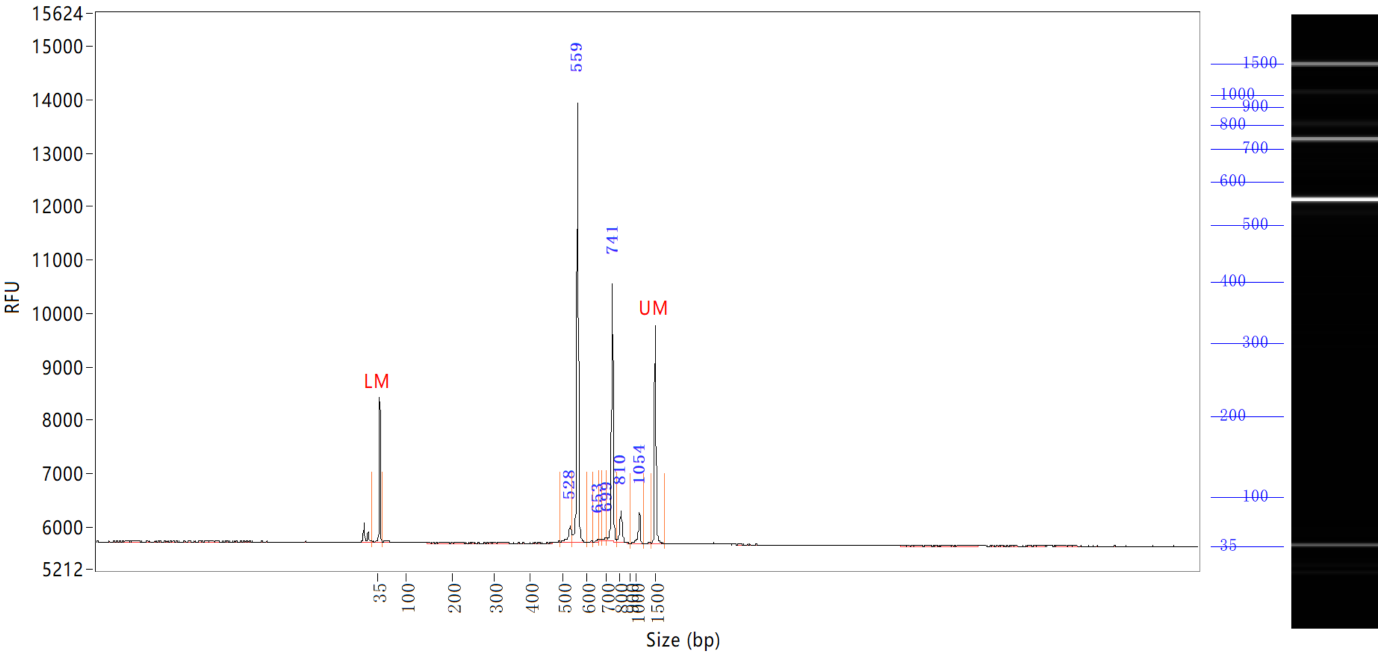

Peak	Size (bp)	Conc. (ng/uL)	From (bp)	To (bp)	Avg. Size (bp)	CV%	RFU	Corr. Peak Area
1	35 (LM)	0.4510	20	42	34	5.11	2714	12.710
2	528	1.3413	490	541	524	2.30	315	3.150
3	559	14.1980	541	602	558	0.86	8252	33.343
4	653	0.1305	630	663	652	1.01	54	0.307
5	699	0.1978	678	706	692	1.16	52	0.465
6	741	7.8795	706	776	740	1.02	4826	18.504
7	810	1.4067	776	905	809	1.82	577	3.303
8	1054	1.1417	905	1201	1037	3.72	597	2.681
9	1500 (UM)	0.5000	1387	1757	1494	2.07	4093	14.091

TIC:	26.2956	ng/uL
TIM:	69.0816	nmole/L
Total Conc.:	28.0757	ng/uL

```
Sample Peak Width (sec): 5     Sample Min Peak Height: 50    Sample Baseline V to V?: Y    Sample Baseline V to V pts: 3
Sample Filter: Binomial        # of Pts for Filter: 3        Sample Start Region (min): 0  Sample End Region (min): 75
Marker Peak Width (sec): 5     Marker Min Peak Height: 500   Marker Baseline V to V?: Y    Marker Baseline V to V pts: 3
Lower Marker Selection: First Peak > 500 RFU                 Upper Marker Selection: Last Peak > 500 RFU
Ladder Size (bp)35, 100, 200, 300, 400, 500, 600, 700, 800, 900, 1000, 1500
Quantification Using: Upper Marker          Final Concentration (ng/uL): 0.5000          Dilution Factor: 12.0
```

 Copyright 2015 Advanced Analytical Technologies, Inc.

Sample: SampG1
Well Location: G1

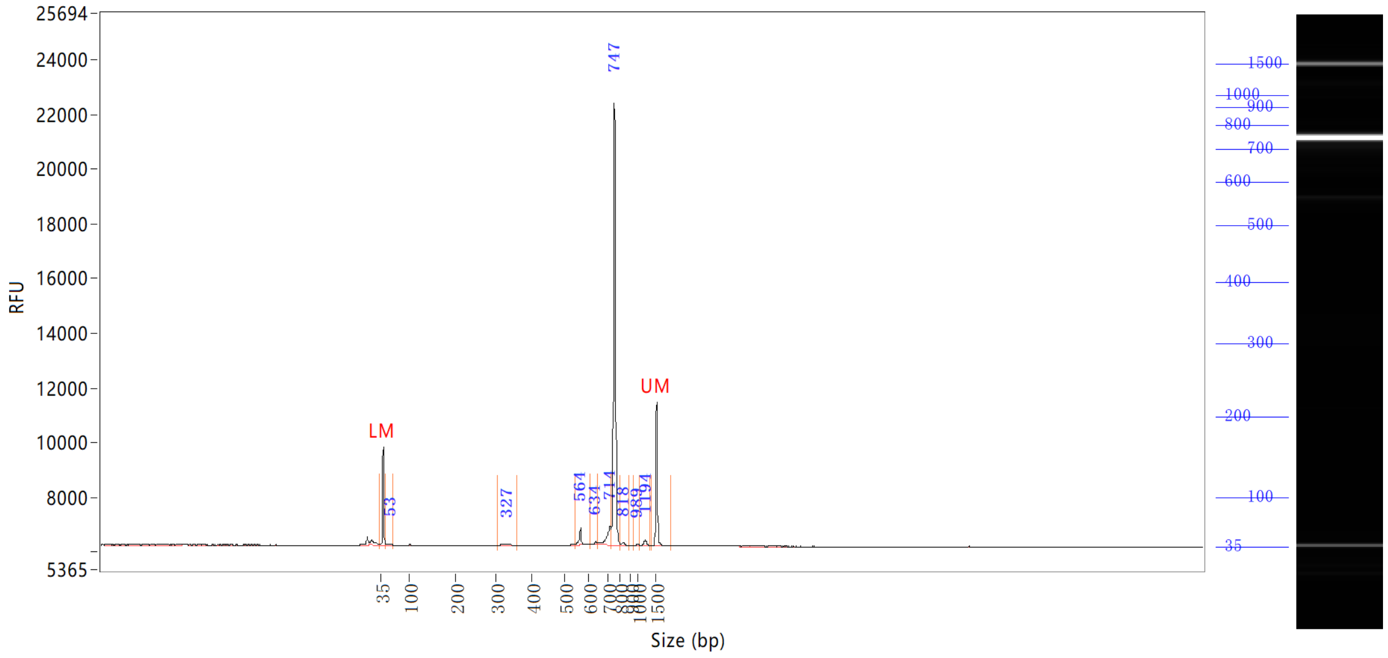

Peak	Size (bp)	Conc. (ng/uL)	From (bp)	To (bp)	Avg. Size (bp)	CV%	RFU	Corr. Peak Area
1	35 (LM)	0.4643	29	44	35	4.85	3570	16.818
2	53	0.2316	44	59	49	6.68	57	0.699
3	327	0.5256	301	356	325	4.16	51	1.587
4	564	1.1182	543	611	563	1.63	620	3.375
5	634	0.2801	611	650	634	1.45	108	0.845
6	714	2.2673	650	723	699	2.52	674	6.844
7	747	24.3035	723	796	746	1.17	16183	73.359
8	818	0.2234	796	886	819	2.04	111	0.674
9	989	0.1250	926	1048	990	2.45	70	0.377
10	1194	0.5048	1048	1314	1177	4.37	215	1.524
11	1500 (UM)	0.5000	1354	1908	1501	2.77	5254	18.111

TIC:	29.5795	ng/uL
TIM:	74.7151	nmole/L
Total Conc.:	30.6973	ng/uL

```
Sample Peak Width (sec): 5    Sample Min Peak Height: 50    Sample Baseline V to V?: Y    Sample Baseline V to V pts: 3
Sample Filter: Binomial       # of Pts for Filter: 3        Sample Start Region (min): 0  Sample End Region (min): 75
Marker Peak Width (sec): 5    Marker Min Peak Height: 500   Marker Baseline V to V?: Y    Marker Baseline V to V pts: 3
Lower Marker Selection: First Peak > 500 RFU                Upper Marker Selection: Last Peak > 500 RFU
Ladder Size (bp)35, 100, 200, 300, 400, 500, 600, 700, 800, 900, 1000, 1500
Quantification Using: Upper Marker          Final Concentration (ng/uL): 0.5000          Dilution Factor: 12.0
```

 Copyright 2015 Advanced Analytical Technologies, Inc.

Sample: SampH1
Well Location: H1

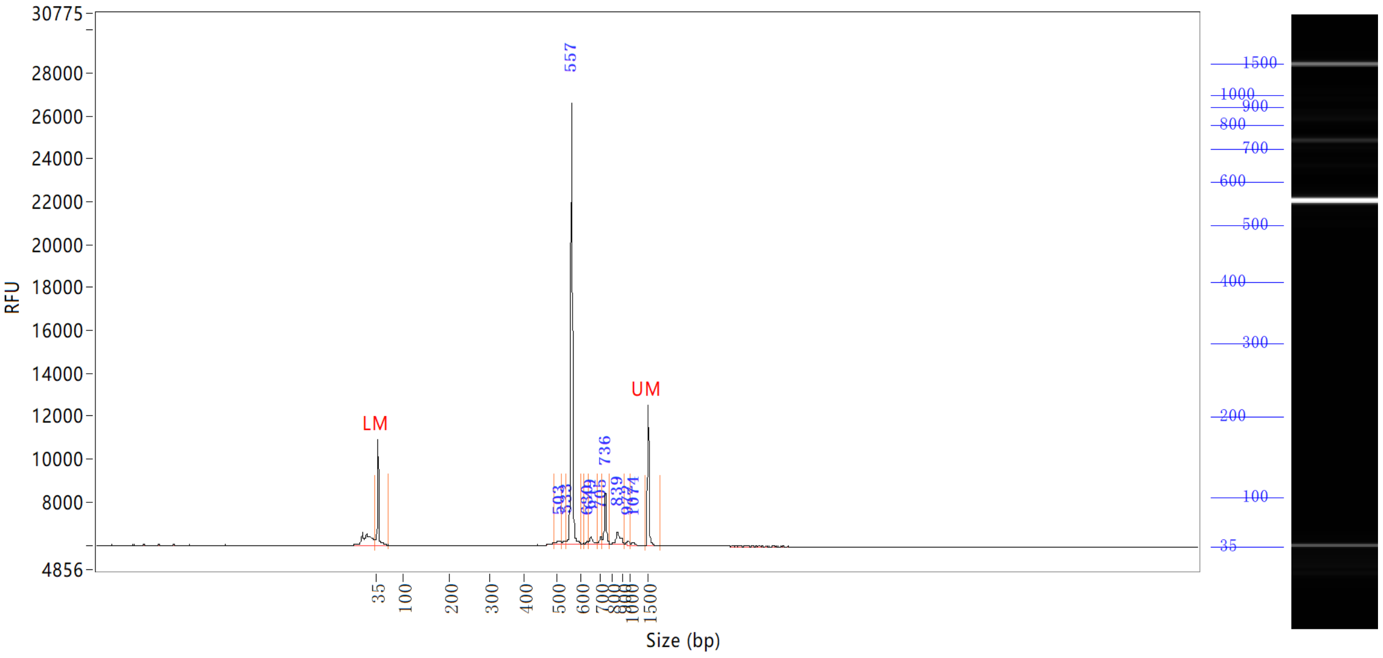

Peak	Size (bp)	Conc. (ng/uL)	From (bp)	To (bp)	Avg. Size (bp)	CV%	RFU	Corr. Peak Area
1	35 (LM)	0.5680	31	64	36	10.30	4960	26.240
2	503	0.3344	488	517	503	1.39	138	1.288
3	533	0.3722	517	538	528	1.13	154	1.433
4	557	23.7051	538	599	556	0.93	20580	91.258
5	630	0.1468	615	637	629	0.73	130	0.565
6	649	0.6894	637	685	654	1.84	366	2.654
7	705	0.5732	685	716	701	1.10	373	2.207
8	736	2.7544	716	776	736	1.20	2382	10.604
9	839	1.6282	776	912	848	3.08	592	6.268
10	972	0.3445	912	1008	961	2.43	197	1.326
11	1074	0.2635	1008	1414	1108	7.63	132	1.015
12	1500 (UM)	0.5000	1427	1862	1495	2.15	6576	23.098

TIC:	30.8118	ng/uL
TIM:	86.1795	nmole/L
Total Conc.:	31.6273	ng/uL

```
Sample Peak Width (sec): 5     Sample Min Peak Height: 50    Sample Baseline V to V?: Y     Sample Baseline V to V pts: 3
Sample Filter: Binomial        # of Pts for Filter: 3        Sample Start Region (min): 0   Sample End Region (min): 75
Marker Peak Width (sec): 5     Marker Min Peak Height: 500   Marker Baseline V to V?: Y     Marker Baseline V to V pts: 3
Lower Marker Selection: First Peak > 500 RFU                 Upper Marker Selection: Last Peak > 500 RFU
Ladder Size (bp)35, 100, 200, 300, 400, 500, 600, 700, 800, 900, 1000, 1500
Quantification Using: Upper Marker          Final Concentration (ng/uL): 0.5000          Dilution Factor: 12.0
```

 Copyright 2015 Advanced Analytical Technologies, Inc.

Sample: SampA2
Well Location: A2

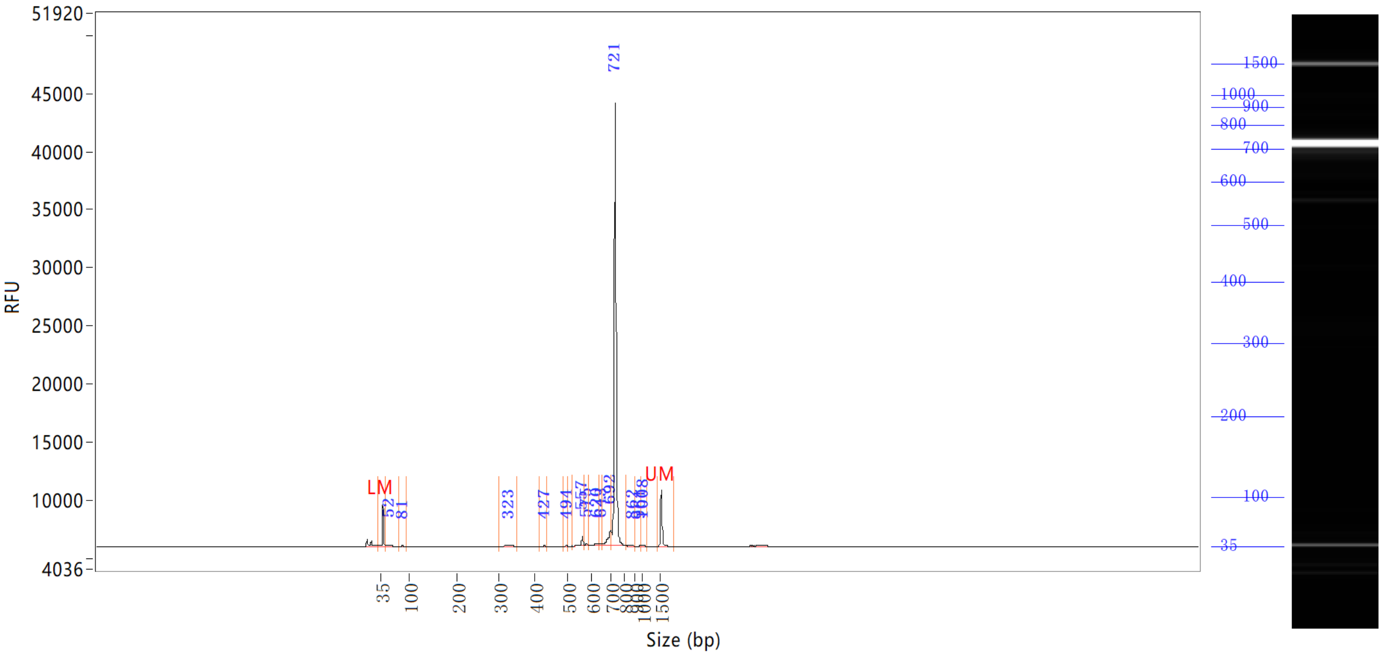

Peak	Size (bp)	Conc. (ng/uL)	From (bp)	To (bp)	Avg. Size (bp)	CV%	RFU	Corr. Peak Area
1	35 (LM)	0.5467	27	44	35	6.89	3641	18.634
2	52	0.8974	44	73	51	10.56	171	2.549
3	81	0.1469	73	91	81	3.72	63	0.417
4	323	0.7311	301	351	324	3.07	96	2.077
5	427	0.2039	413	435	425	1.14	79	0.579
6	494	0.0749	488	499	493	0.39	63	0.213
7	557	1.1197	521	567	555	0.97	799	3.180
8	575	0.2328	567	584	573	0.59	145	0.661
9	620	0.6249	584	637	620	1.65	192	1.775
10	643	0.2976	637	652	644	0.72	147	0.845
11	692	4.0732	652	699	683	1.67	1352	11.570
12	721	62.3455	699	818	722	1.37	38212	177.092
13	862	0.1895	818	895	855	1.79	73	0.538
14	961	0.1294	895	979	953	1.86	65	0.367
15	1008	0.1696	979	1114	1009	2.44	88	0.482
16	1500 (UM)	0.5000	1407	1869	1501	2.64	4842	17.043

TIC:	71.2363	ng/uL
TIM:	195.7515	nmole/L
Total Conc.:	71.9391	ng/uL

Sample Peak Width (sec): 5   Sample Min Peak Height: 50   Sample Baseline V to V?: Y   Sample Baseline V to V pts: 3
Sample Filter: Binomial   # of Pts for Filter: 3   Sample Start Region (min): 0   Sample End Region (min): 75
Marker Peak Width (sec): 5   Marker Min Peak Height: 500   Marker Baseline V to V?: Y   Marker Baseline V to V pts: 3
Lower Marker Selection: First Peak > 500 RFU   Upper Marker Selection: Last Peak > 500 RFU
Ladder Size (bp)35, 100, 200, 300, 400, 500, 600, 700, 800, 900, 1000, 1500
Quantification Using: Upper Marker   Final Concentration (ng/uL): 0.5000   Dilution Factor: 12.0

 Copyright 2015 Advanced Analytical Technologies, Inc.

Sample: SampB2
Well Location: B2

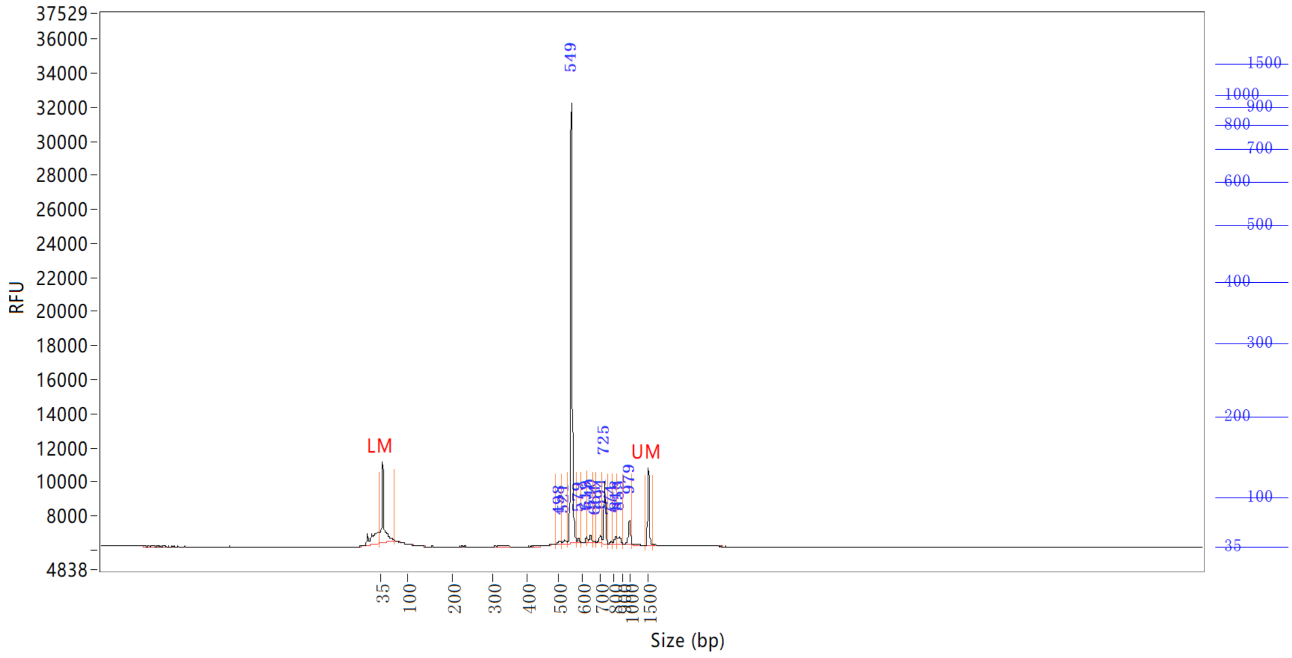

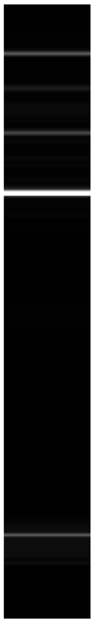

Peak	Size (bp)	Conc. (ng/uL)	From (bp)	To (bp)	Avg. Size (bp)	CV%	RFU	Corr. Peak Area
1	35 (LM)	1.2162	29	65	38	17.75	4723	39.996
2	498	0.5149	489	512	500	1.02	178	1.411
3	521	0.5898	512	533	523	1.06	197	1.616
4	549	40.4488	533	574	550	0.81	25920	110.853
5	579	0.5300	574	595	581	0.80	250	1.452
6	619	0.4970	595	626	617	0.82	323	1.362
7	640	0.7285	626	654	638	0.88	385	1.997
8	664	0.1060	654	675	662	0.65	64	0.291
9	694	0.9298	675	705	692	0.92	425	2.548
10	725	5.7170	705	756	724	1.04	3631	15.668
11	774	0.4141	756	789	775	1.02	225	1.135
12	818	1.3258	789	836	814	1.61	474	3.633
13	853	1.1519	836	893	855	1.45	472	3.157
14	979	2.6828	893	1054	970	2.20	1408	7.353
15	1500 (UM)	0.5000	1407	1651	1502	1.93	4603	16.443

TIC:	55.6366	ng/uL
TIM:	155.0784	nmole/L
Total Conc.:	56.9064	ng/uL

```
Sample Peak Width (sec): 5    Sample Min Peak Height: 50    Sample Baseline V to V?: Y    Sample Baseline V to V pts: 3
Sample Filter: Binomial       # of Pts for Filter: 3        Sample Start Region (min): 0  Sample End Region (min): 75
Marker Peak Width (sec): 5    Marker Min Peak Height: 500   Marker Baseline V to V?: Y    Marker Baseline V to V pts: 3
Lower Marker Selection: First Peak > 500 RFU                Upper Marker Selection: Last Peak > 500 RFU
Ladder Size (bp)35, 100, 200, 300, 400, 500, 600, 700, 800, 900, 1000, 1500
Quantification Using: Upper Marker          Final Concentration (ng/uL): 0.5000          Dilution Factor: 12.0
```

 Copyright 2015 Advanced Analytical Technologies, Inc.

Sample: SampC2
Well Location: C2

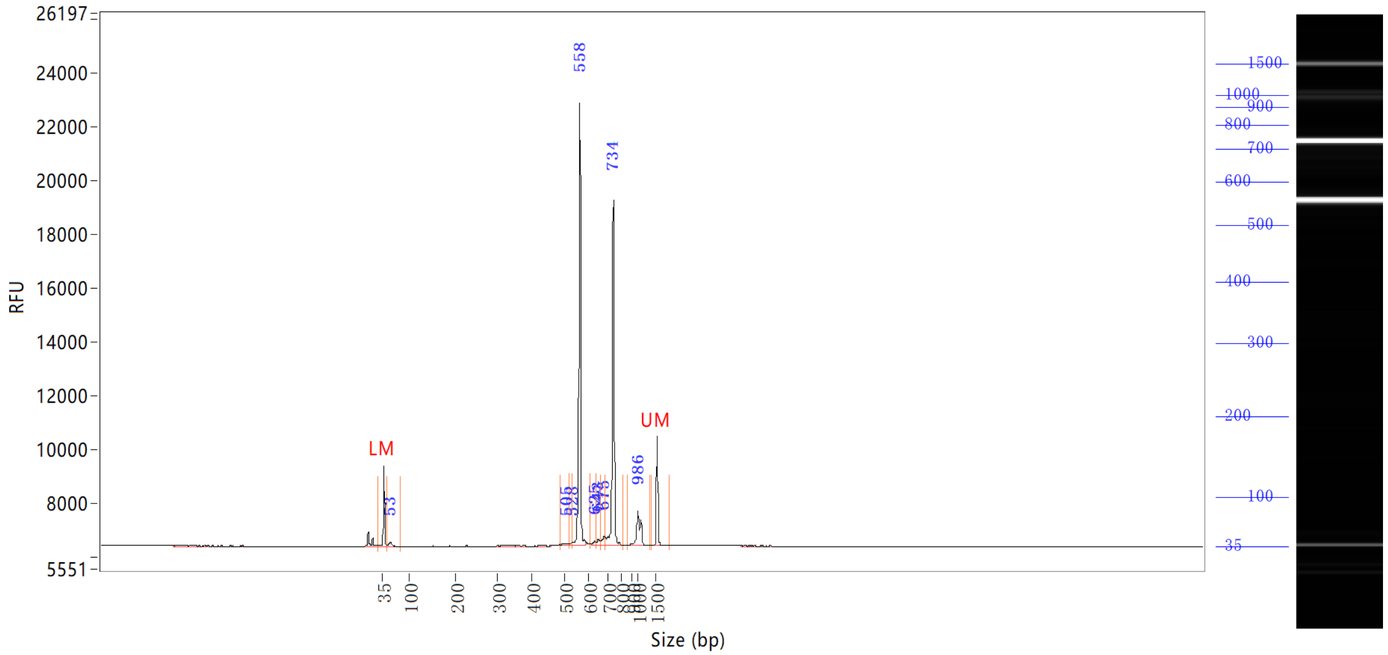

Peak	Size (bp)	Conc. (ng/uL)	From (bp)	To (bp)	Avg. Size (bp)	CV%	RFU	Corr. Peak Area
1	35 (LM)	0.5137	22	45	35	6.31	2961	14.497
2	53	0.7161	45	75	52	9.66	155	1.684
3	505	0.2688	485	515	500	1.51	60	0.632
4	528	0.2551	515	532	526	0.90	102	0.600
5	558	29.9991	532	601	557	0.94	16429	70.555
6	625	0.3197	601	633	623	1.07	131	0.752
7	643	0.6619	633	659	646	1.16	224	1.557
8	675	1.1411	659	687	674	1.14	320	2.684
9	734	23.9223	687	810	731	1.54	12837	56.263
10	986	4.4374	865	1300	1002	4.63	1281	10.436
11	1500 (UM)	0.5000	1354	1836	1501	2.40	4067	14.111

TIC:	61.7214	ng/uL
TIM:	179.3365	nmole/L
Total Conc.:	63.4607	ng/uL

Sample Peak Width (sec): 5 Sample Min Peak Height: 50 Sample Baseline V to V?: Y Sample Baseline V to V pts: 3
Sample Filter: Binomial # of Pts for Filter: 3 Sample Start Region (min): 0 Sample End Region (min): 75
Marker Peak Width (sec): 5 Marker Min Peak Height: 500 Marker Baseline V to V?: Y Marker Baseline V to V pts: 3
Lower Marker Selection: First Peak > 500 RFU Upper Marker Selection: Last Peak > 500 RFU
Ladder Size (bp)35, 100, 200, 300, 400, 500, 600, 700, 800, 900, 1000, 1500
Quantification Using: Upper Marker Final Concentration (ng/uL): 0.5000 Dilution Factor: 12.0

 Copyright 2015 Advanced Analytical Technologies, Inc.

Sample: SampD2
Well Location: D2

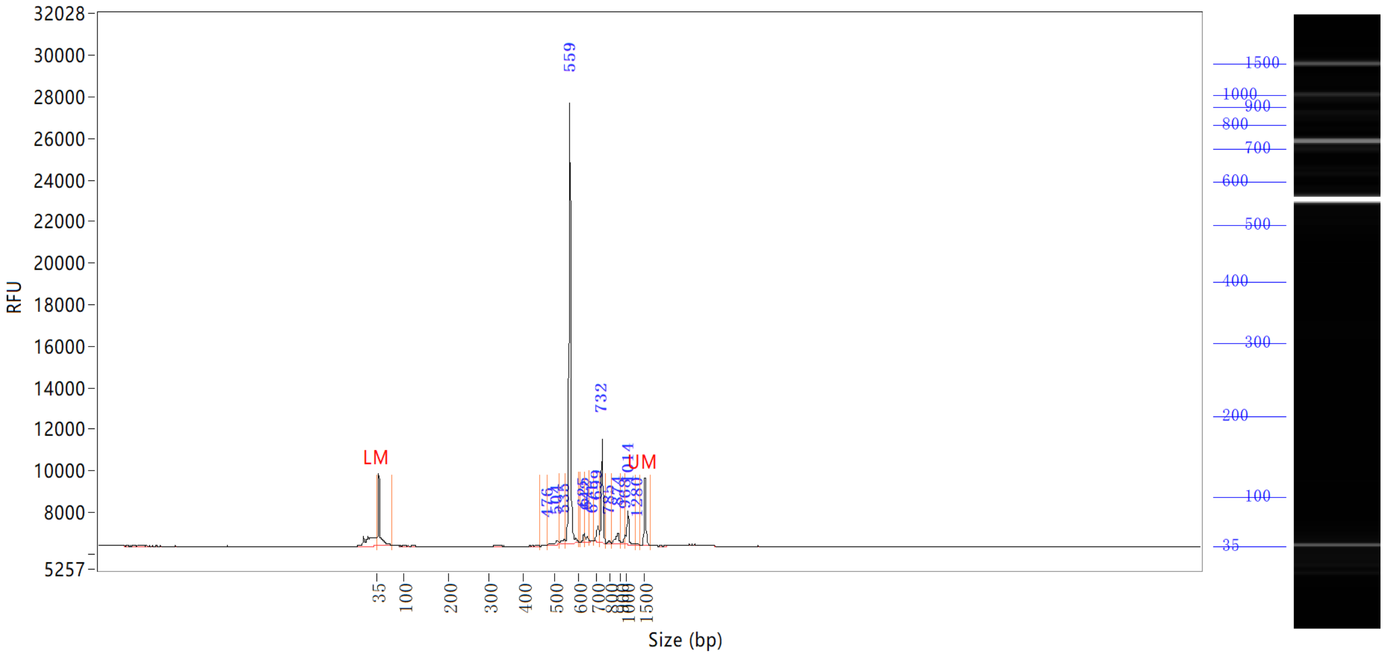

Peak	Size (bp)	Conc. (ng/uL)	From (bp)	To (bp)	Avg. Size (bp)	CV%	RFU	Corr. Peak Area
1	35 (LM)	0.9991	31	70	38	17.59	3454	24.223
2	476	0.1727	450	477	469	1.50	51	0.349
3	504	1.1445	477	517	499	2.19	180	2.312
4	535	0.9572	517	540	529	1.27	196	1.934
5	559	46.6671	540	598	558	0.97	21249	94.283
6	625	0.9675	610	634	624	0.83	424	1.955
7	642	0.7269	634	659	643	0.97	284	1.469
8	671	0.1723	659	681	668	0.76	62	0.348
9	699	2.1078	681	712	699	0.97	747	4.259
10	732	10.4430	712	765	731	1.04	4971	21.098
11	785	0.3543	765	806	786	1.03	149	0.716
12	874	2.2547	806	905	859	2.39	545	4.555
13	968	1.0970	905	979	955	1.85	474	2.216
14	1014	3.7070	979	1247	1025	5.23	1648	7.489
15	1280	0.1926	1247	1380	1289	2.18	72	0.389
16	1500 (UM)	0.5000	1380	1665	1502	1.83	3281	12.122

TIC:	70.9645	ng/uL
TIM:	191.4447	nmole/L
Total Conc.:	72.4574	ng/uL

Sample Peak Width (sec): 5 Sample Min Peak Height: 50 Sample Baseline V to V?: Y Sample Baseline V to V pts: 3
Sample Filter: Binomial # of Pts for Filter: 3 Sample Start Region (min): 0 Sample End Region (min): 75
Marker Peak Width (sec): 5 Marker Min Peak Height: 500 Marker Baseline V to V?: Y Marker Baseline V to V pts: 3
Lower Marker Selection: First Peak > 500 RFU Upper Marker Selection: Last Peak > 500 RFU
Ladder Size (bp)35, 100, 200, 300, 400, 500, 600, 700, 800, 900, 1000, 1500
Quantification Using: Upper Marker Final Concentration (ng/uL): 0.5000 Dilution Factor: 12.0

 Copyright 2015 Advanced Analytical Technologies, Inc.

Sample: SampE2
Well Location: E2

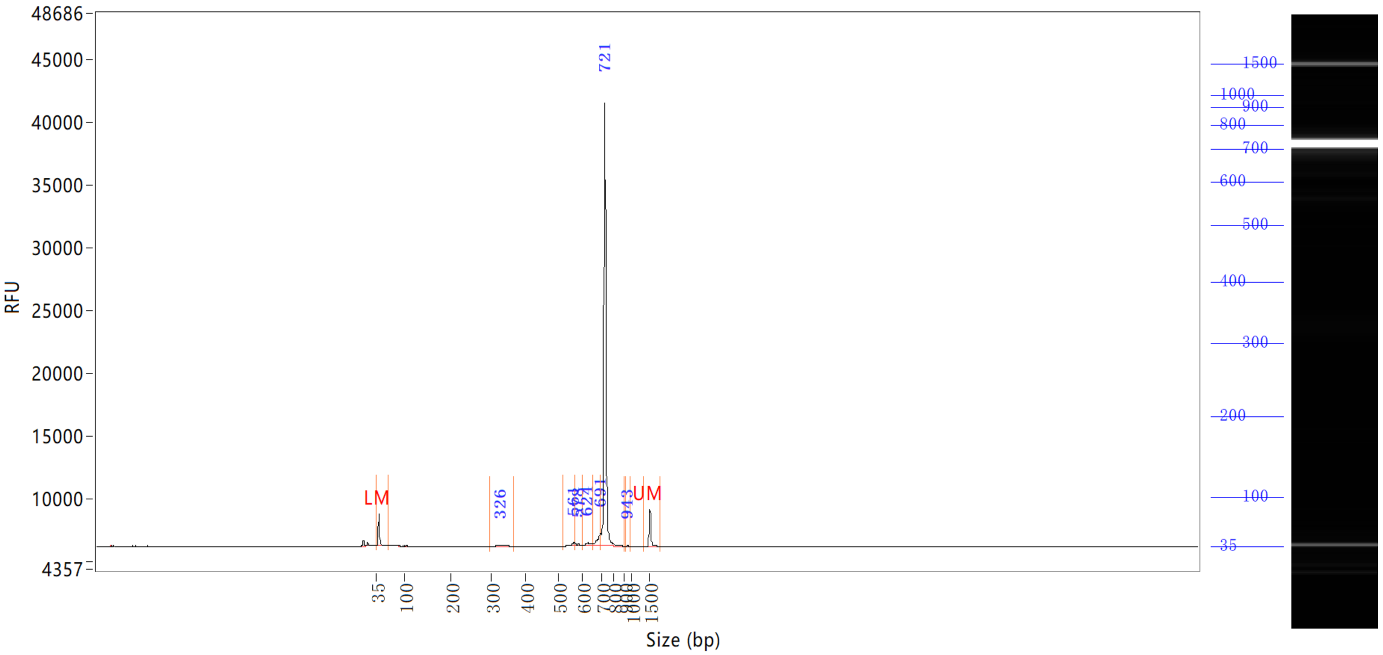

Peak	Size (bp)	Conc. (ng/uL)	From (bp)	To (bp)	Avg. Size (bp)	CV%	RFU	Corr. Peak Area
1	35 (LM)	0.6482	31	60	37	13.13	2557	14.690
2	326	1.3590	295	361	327	3.72	102	2.566
3	561	0.9310	516	571	556	1.57	257	1.758
4	578	0.3264	571	597	579	0.91	106	0.616
5	624	1.1184	597	652	629	1.94	206	2.112
6	691	4.7504	652	697	682	1.67	994	8.971
7	721	88.9609	697	888	722	1.71	35368	168.005
8	943	0.1626	912	982	944	1.83	60	0.307
9	1500 (UM)	0.5000	1347	1776	1501	3.03	2992	11.331

TIC:	97.6088	ng/uL
TIM:	227.9756	nmole/L
Total Conc.:	98.9808	ng/uL

```
Sample Peak Width (sec): 5    Sample Min Peak Height: 50    Sample Baseline V to V?: Y    Sample Baseline V to V pts: 3
Sample Filter: Binomial       # of Pts for Filter: 3        Sample Start Region (min): 0  Sample End Region (min): 75
Marker Peak Width (sec): 5    Marker Min Peak Height: 500   Marker Baseline V to V?: Y    Marker Baseline V to V pts: 3
Lower Marker Selection: First Peak > 500 RFU                Upper Marker Selection: Last Peak > 500 RFU
Ladder Size (bp)35, 100, 200, 300, 400, 500, 600, 700, 800, 900, 1000, 1500
Quantification Using: Upper Marker          Final Concentration (ng/uL): 0.5000          Dilution Factor: 12.0
```

 Copyright 2015 Advanced Analytical Technologies, Inc.

Sample: SampF2
Well Location: F2

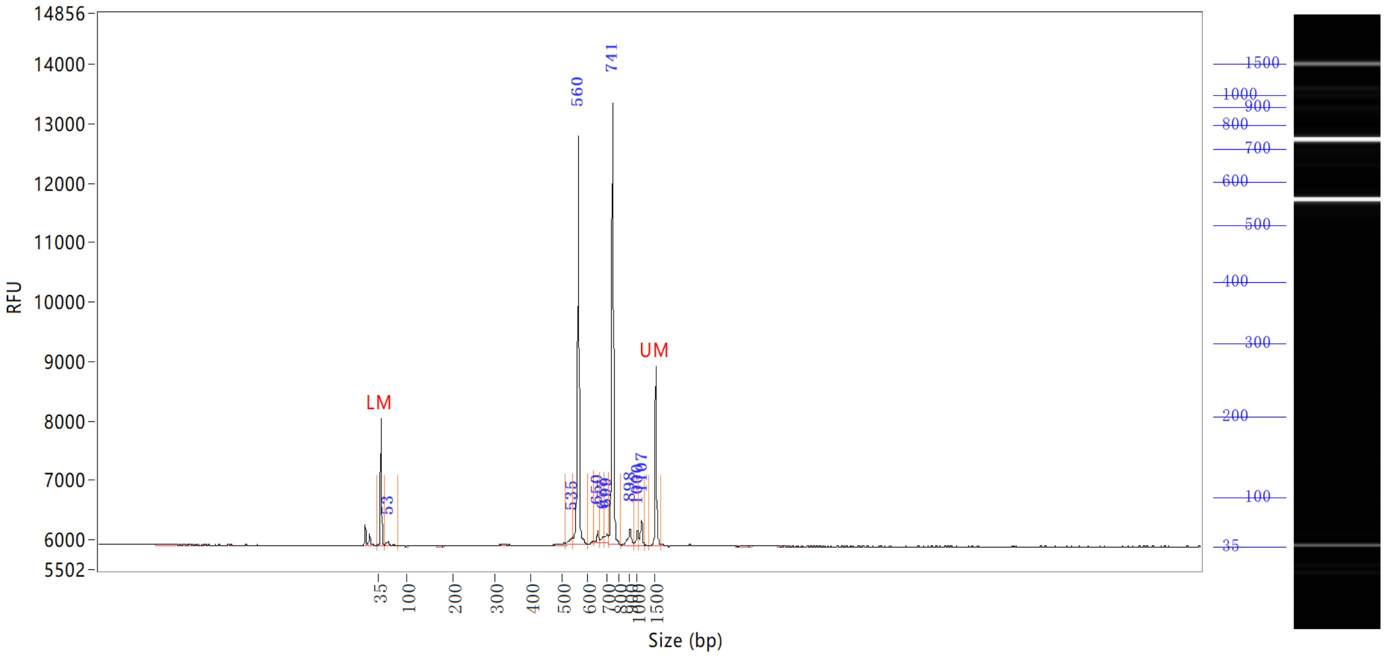

Peak	Size (bp)	Conc. (ng/uL)	From (bp)	To (bp)	Avg. Size (bp)	CV%	RFU	Corr. Peak Area
1	35 (LM)	0.4855	28	44	35	4.80	2123	10.400
2	53	0.5514	44	79	55	13.27	64	0.984
3	535	0.7536	508	540	527	1.69	127	1.345
4	560	16.7340	540	601	558	0.98	6867	29.872
5	650	0.4035	635	662	650	0.78	182	0.720
6	677	0.4881	662	690	678	1.13	109	0.871
7	699	0.5095	690	710	699	0.84	151	0.910
8	741	17.6842	710	808	739	1.23	7426	31.568
9	898	1.1678	808	947	886	2.68	276	2.085
10	1000	0.7153	947	1048	999	2.26	248	1.277
11	1107	1.0821	1048	1194	1101	2.41	433	1.932
12	1500 (UM)	0.5000	1334	1671	1498	1.99	3021	10.711

TIC:	40.0896	ng/uL
TIM:	115.8872	nmole/L
Total Conc.:	42.2284	ng/uL

Sample Peak Width (sec): 5 Sample Min Peak Height: 50 Sample Baseline V to V?: Y Sample Baseline V to V pts: 3
Sample Filter: Binomial # of Pts for Filter: 3 Sample Start Region (min): 0 Sample End Region (min): 75
Marker Peak Width (sec): 5 Marker Min Peak Height: 500 Marker Baseline V to V?: Y Marker Baseline V to V pts: 3
Lower Marker Selection: First Peak > 500 RFU Upper Marker Selection: Last Peak > 500 RFU
Ladder Size (bp)35, 100, 200, 300, 400, 500, 600, 700, 800, 900, 1000, 1500
Quantification Using: Upper Marker Final Concentration (ng/uL): 0.5000 Dilution Factor: 12.0

 Copyright 2015 Advanced Analytical Technologies, Inc.

Sample: SampG2
Well Location: G2

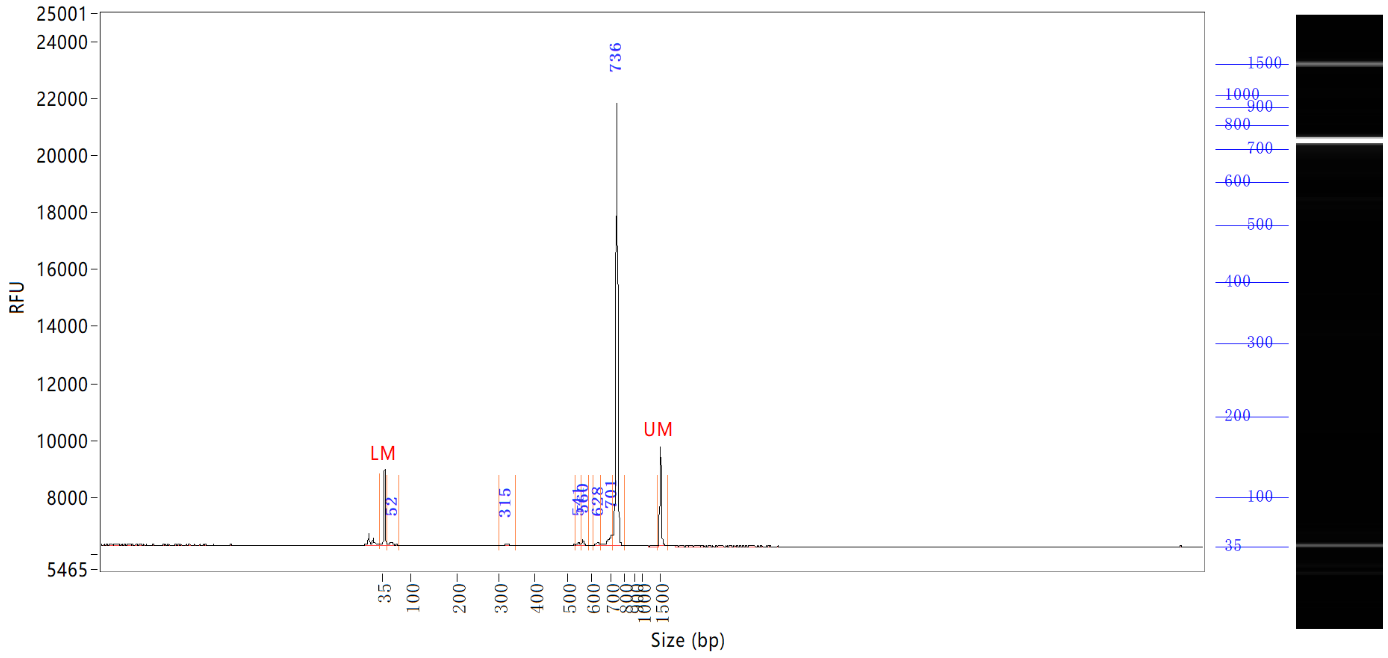

Peak	Size (bp)	Conc. (ng/uL)	From (bp)	To (bp)	Avg. Size (bp)	CV%	RFU	Corr. Peak Area
1	35 (LM)	0.5772	25	44	34	6.40	2679	13.663
2	52	0.6623	44	72	51	10.68	90	1.307
3	315	0.4968	299	344	319	2.92	51	0.980
4	541	0.3916	529	553	542	1.14	94	0.772
5	560	0.4617	553	586	561	1.09	189	0.911
6	628	0.4264	604	644	627	1.57	91	0.841
7	701	1.9628	644	708	687	2.25	365	3.872
8	736	34.5584	708	798	735	1.08	15574	68.169
9	1500 (UM)	0.5000	1420	1724	1496	1.84	3482	11.835

TIC:	38.9600	ng/uL
TIM:	109.5806	nmole/L
Total Conc.:	40.7400	ng/uL

```
Sample Peak Width (sec): 5     Sample Min Peak Height: 50    Sample Baseline V to V?: Y    Sample Baseline V to V pts: 3
Sample Filter: Binomial        # of Pts for Filter: 3        Sample Start Region (min): 0  Sample End Region (min): 75
Marker Peak Width (sec): 5     Marker Min Peak Height: 500   Marker Baseline V to V?: Y    Marker Baseline V to V pts: 3
Lower Marker Selection: First Peak > 500 RFU                 Upper Marker Selection: Last Peak > 500 RFU
Ladder Size (bp)35, 100, 200, 300, 400, 500, 600, 700, 800, 900, 1000, 1500
Quantification Using: Upper Marker          Final Concentration (ng/uL): 0.5000          Dilution Factor: 12.0
```

 Copyright 2015 Advanced Analytical Technologies, Inc.

Sample: SampH2
Well Location: H2

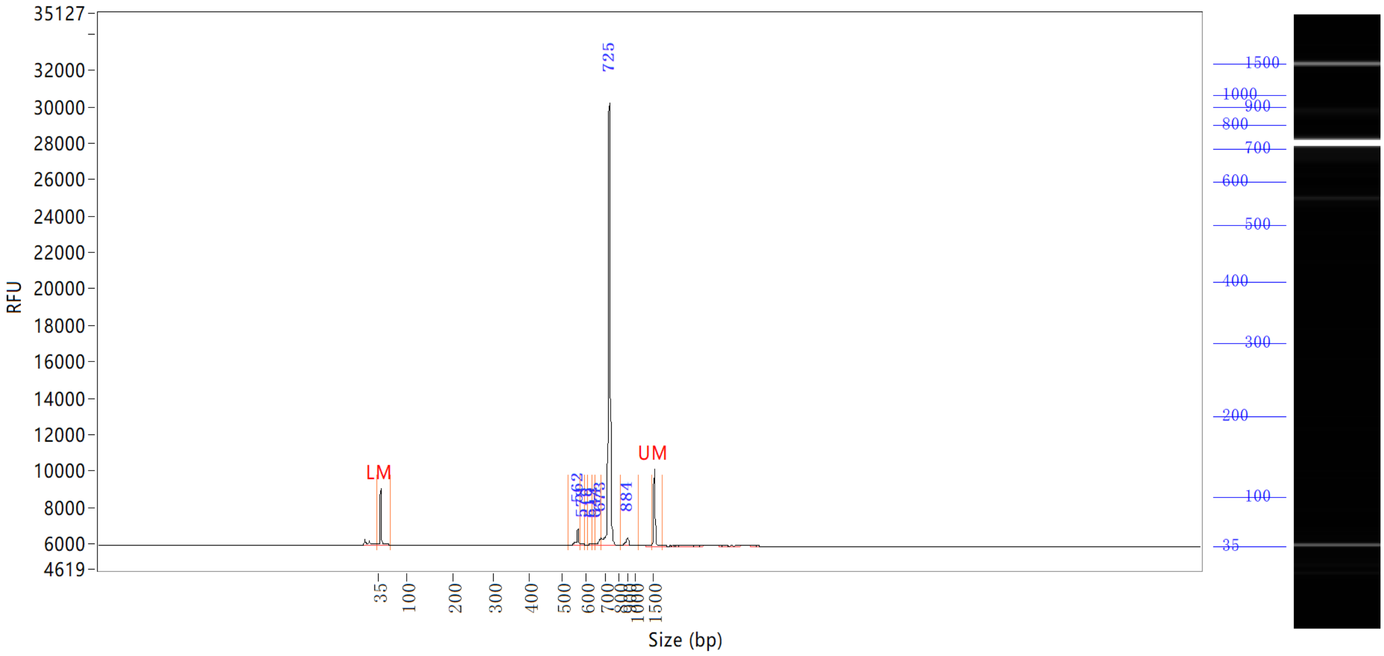

Peak	Size (bp)	Conc. (ng/uL)	From (bp)	To (bp)	Avg. Size (bp)	CV%	RFU	Corr. Peak Area
1	35 (LM)	0.5986	29	62	36	12.19	3121	16.735
2	562	1.9275	526	572	557	1.45	855	4.491
3	578	0.2676	572	593	579	0.84	89	0.623
4	619	0.1889	607	633	620	0.98	76	0.440
5	644	0.1304	633	649	641	0.76	55	0.304
6	673	1.3202	649	682	670	1.22	383	3.076
7	725	47.9659	682	813	723	1.37	24318	111.749
8	884	1.3601	813	1074	876	2.46	453	3.169
9	1500 (UM)	0.5000	1433	1737	1497	1.87	4199	13.979

TIC:	53.1606	ng/uL
TIM:	122.2747	nmole/L
Total Conc.:	54.9462	ng/uL

```
Sample Peak Width (sec): 5    Sample Min Peak Height: 50    Sample Baseline V to V?: Y    Sample Baseline V to V pts: 3
Sample Filter: Binomial       # of Pts for Filter: 3        Sample Start Region (min): 0  Sample End Region (min): 75
Marker Peak Width (sec): 5    Marker Min Peak Height: 500   Marker Baseline V to V?: Y    Marker Baseline V to V pts: 3
Lower Marker Selection: First Peak > 500 RFU                Upper Marker Selection: Last Peak > 500 RFU
Ladder Size (bp)35, 100, 200, 300, 400, 500, 600, 700, 800, 900, 1000, 1500
Quantification Using: Upper Marker         Final Concentration (ng/uL): 0.5000          Dilution Factor: 12.0
```

 Copyright 2015 Advanced Analytical Technologies, Inc.

Sample: SampA3
Well Location: A3

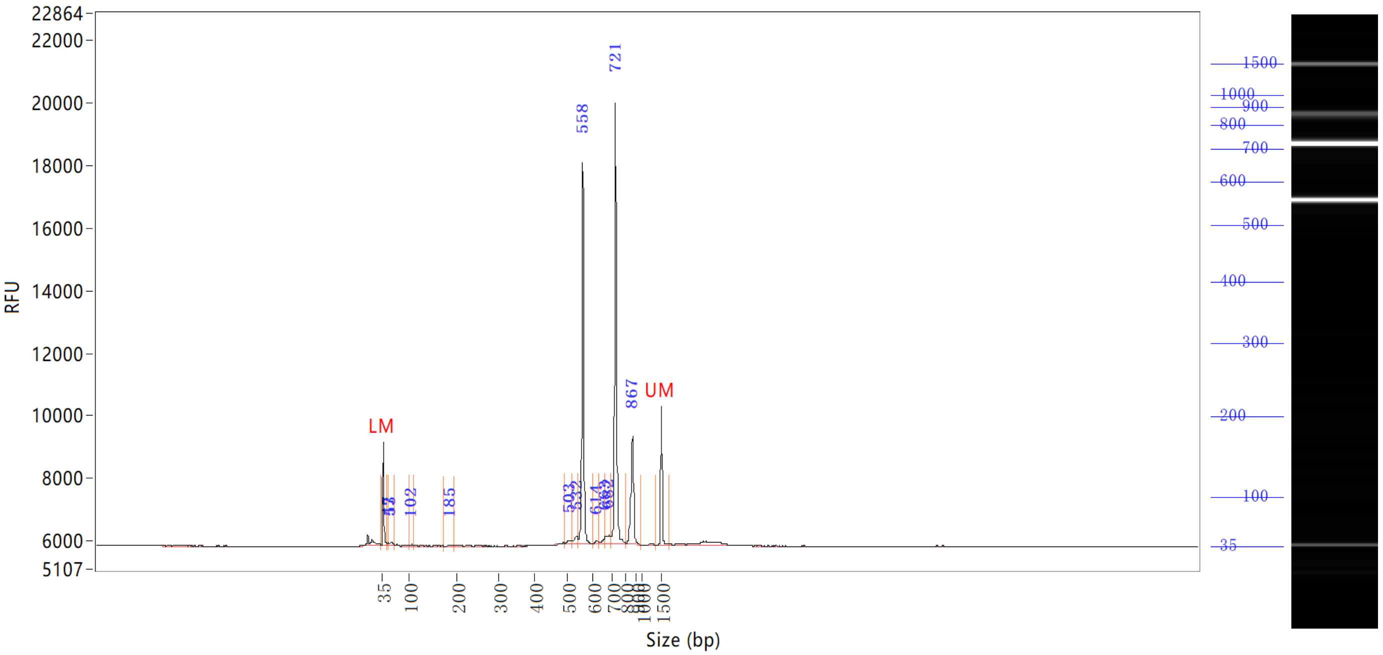

Peak	Size (bp)	Conc. (ng/uL)	From (bp)	To (bp)	Avg. Size (bp)	CV%	RFU	Corr. Peak Area
1	35 (LM)	0.5367	30	44	35	5.45	3296	15.653
2	47	0.2351	44	49	47	3.29	79	0.571
3	53	0.4637	49	62	54	5.85	86	1.127
4	102	0.2489	96	108	102	2.99	66	0.605
5	185	0.3275	170	194	184	2.77	57	0.796
6	503	0.4962	490	519	506	1.41	113	1.206
7	532	0.8277	519	540	530	1.06	247	2.011
8	558	21.2459	540	601	557	0.85	12150	51.633
9	614	0.2653	601	630	617	1.09	89	0.645
10	663	0.5553	630	664	653	1.51	226	1.350
11	682	1.2238	664	695	680	1.34	267	2.974
12	721	26.0054	695	806	722	1.52	14103	63.200
13	867	8.6072	806	972	860	1.71	3462	20.918
14	1500 (UM)	0.5000	1354	1737	1494	2.04	4447	14.582

TIC:	60.5019	ng/uL
TIM:	176.9642	nmole/L
Total Conc.:	62.2188	ng/uL

```
Sample Peak Width (sec): 5    Sample Min Peak Height: 50    Sample Baseline V to V?: Y    Sample Baseline V to V pts: 3
Sample Filter: Binomial       # of Pts for Filter: 3        Sample Start Region (min): 0  Sample End Region (min): 75
Marker Peak Width (sec): 5    Marker Min Peak Height: 500   Marker Baseline V to V?: Y    Marker Baseline V to V pts: 3
Lower Marker Selection: First Peak > 500 RFU                Upper Marker Selection: Last Peak > 500 RFU
Ladder Size (bp)35, 100, 200, 300, 400, 500, 600, 700, 800, 900, 1000, 1500
Quantification Using: Upper Marker          Final Concentration (ng/uL): 0.5000          Dilution Factor: 12.0
```

 Copyright 2015 Advanced Analytical Technologies, Inc.

Sample: SampB3
Well Location: B3

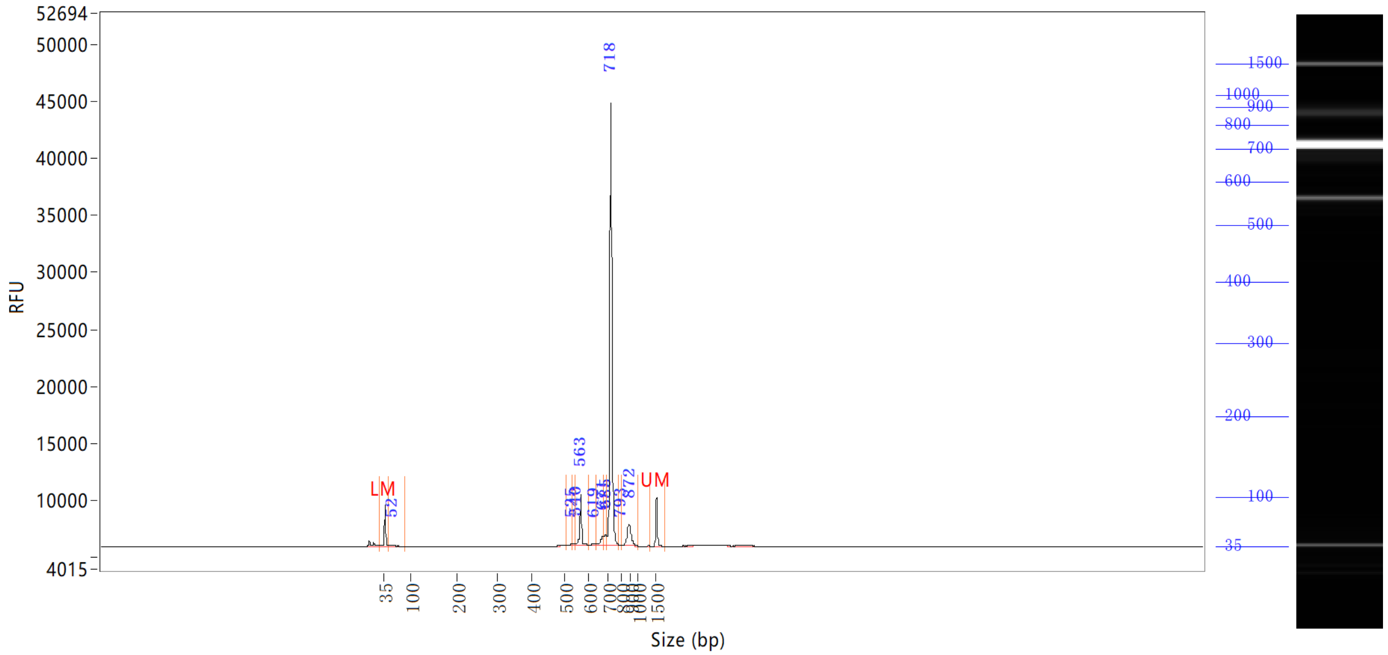

Peak	Size (bp)	Conc. (ng/uL)	From (bp)	To (bp)	Avg. Size (bp)	CV%	RFU	Corr. Peak Area
1	35 (LM)	0.6822	24	45	35	9.09	3619	19.870
2	52	1.3528	45	83	56	16.27	153	3.283
3	525	0.3805	502	530	523	1.15	157	0.924
4	540	0.4526	530	543	537	0.71	195	1.099
5	563	8.3952	543	596	561	1.06	4501	20.376
6	619	0.3274	596	634	618	1.38	108	0.795
7	671	2.4881	634	677	664	1.41	772	6.039
8	685	2.1715	677	694	685	0.73	836	5.271
9	718	77.6502	694	782	717	1.31	38773	188.467
10	793	0.1196	782	808	790	0.69	64	0.290
11	872	7.2180	808	996	875	2.72	1900	17.519
12	1500 (UM)	0.5000	1347	1744	1497	1.99	4264	14.563

TIC:	100.5558	ng/uL
TIM:	271.1132	nmole/L
Total Conc.:	102.1971	ng/uL

```
Sample Peak Width (sec): 5    Sample Min Peak Height: 50    Sample Baseline V to V?: Y    Sample Baseline V to V pts: 3
Sample Filter: Binomial       # of Pts for Filter: 3        Sample Start Region (min): 0  Sample End Region (min): 75
Marker Peak Width (sec): 5    Marker Min Peak Height: 500   Marker Baseline V to V?: Y    Marker Baseline V to V pts: 3
Lower Marker Selection: First Peak > 500 RFU                Upper Marker Selection: Last Peak > 500 RFU
Ladder Size (bp)35, 100, 200, 300, 400, 500, 600, 700, 800, 900, 1000, 1500
Quantification Using: Upper Marker          Final Concentration (ng/uL): 0.5000          Dilution Factor: 12.0
```

 Copyright 2015 Advanced Analytical Technologies, Inc.

Sample: SampC3
Well Location: C3

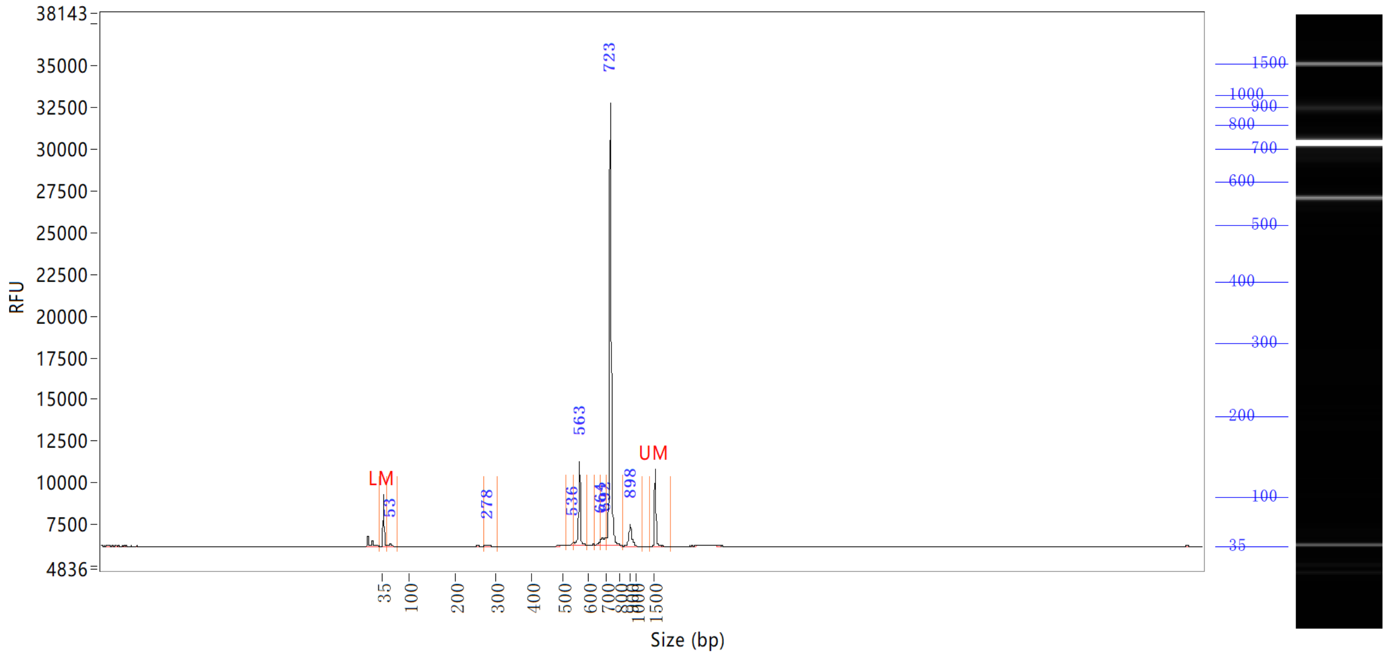

Peak	Size (bp)	Conc. (ng/uL)	From (bp)	To (bp)	Avg. Size (bp)	CV%	RFU	Corr. Peak Area
1	35 (LM)	0.4883	29	44	35	5.76	3127	15.456
2	53	0.9681	44	71	52	10.08	144	2.553
3	278	0.3604	268	304	280	2.74	52	0.951
4	536	0.5327	507	542	532	1.65	178	1.405
5	563	8.0268	542	597	561	1.04	4974	21.172
6	664	0.6097	635	666	657	1.13	361	1.608
7	692	2.0923	666	699	683	1.45	479	5.519
8	723	43.9969	699	827	723	1.45	26571	116.050
9	898	3.4627	827	1147	902	2.96	1287	9.134
10	1500 (UM)	0.5000	1354	1921	1499	3.08	4631	15.826

TIC:	60.0497	ng/uL
TIM:	170.8456	nmole/L
Total Conc.:	61.7462	ng/uL

```
Sample Peak Width (sec): 5     Sample Min Peak Height: 50    Sample Baseline V to V?: Y    Sample Baseline V to V pts: 3
Sample Filter: Binomial        # of Pts for Filter: 3        Sample Start Region (min): 0  Sample End Region (min): 75
Marker Peak Width (sec): 5     Marker Min Peak Height: 500   Marker Baseline V to V?: Y    Marker Baseline V to V pts: 3
Lower Marker Selection: First Peak > 500 RFU                 Upper Marker Selection: Last Peak > 500 RFU
Ladder Size (bp)35, 100, 200, 300, 400, 500, 600, 700, 800, 900, 1000, 1500
Quantification Using: Upper Marker          Final Concentration (ng/uL): 0.5000          Dilution Factor: 12.0
```

 Copyright 2015 Advanced Analytical Technologies, Inc.

Sample: SampD3
Well Location: D3

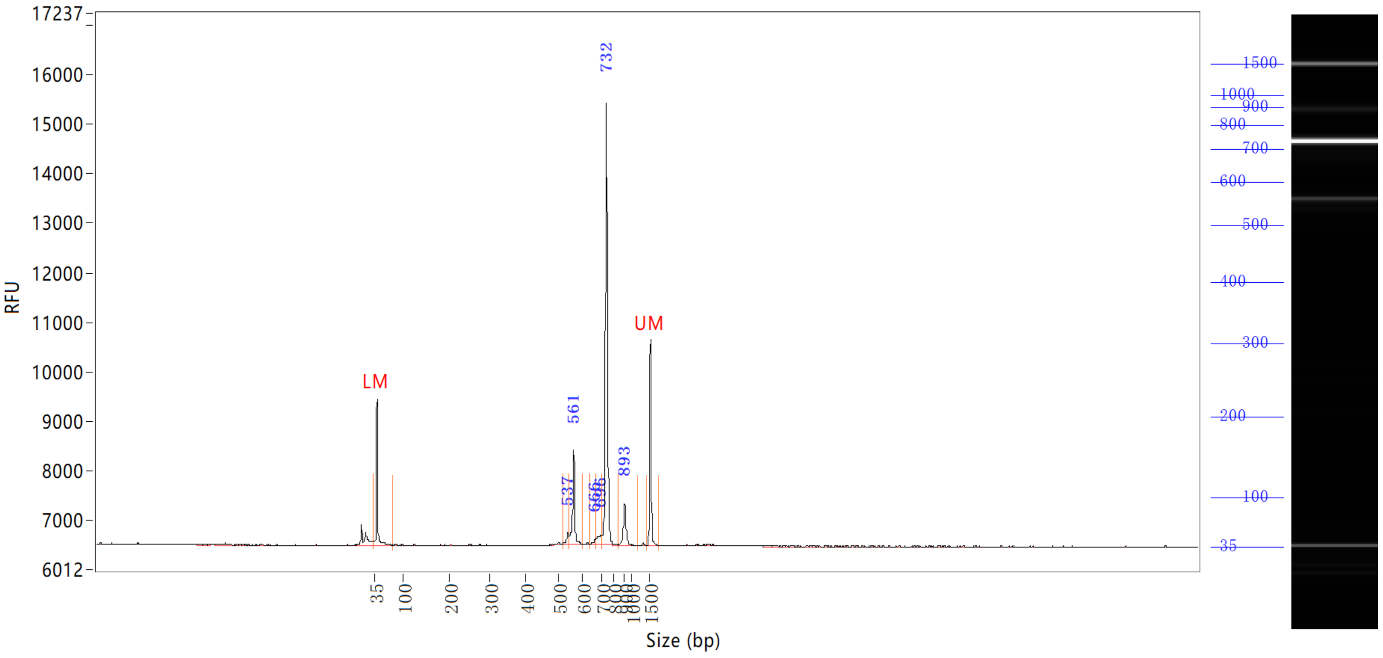

Peak	Size (bp)	Conc. (ng/uL)	From (bp)	To (bp)	Avg. Size (bp)	CV%	RFU	Corr. Peak Area
1	35 (LM)	0.5755	30	73	36	14.58	2939	15.970
2	537	0.6418	523	543	536	0.91	236	1.484
3	561	4.1606	543	601	560	1.26	1897	9.622
4	666	0.2818	638	668	659	1.18	136	0.652
5	696	0.9694	668	703	687	1.46	206	2.242
6	732	17.9695	703	829	732	1.42	8928	41.555
7	893	2.2460	829	1161	896	3.07	850	5.194
8	1500 (UM)	0.5000	1414	1744	1499	2.01	4159	13.875

TIC:	26.2692	ng/uL
TIM:	61.7500	nmole/L
Total Conc.:	27.9864	ng/uL

```
Sample Peak Width (sec): 5    Sample Min Peak Height: 50    Sample Baseline V to V?: Y    Sample Baseline V to V pts: 3
Sample Filter: Binomial       # of Pts for Filter: 3        Sample Start Region (min): 0  Sample End Region (min): 75
Marker Peak Width (sec): 5    Marker Min Peak Height: 500   Marker Baseline V to V?: Y    Marker Baseline V to V pts: 3
Lower Marker Selection: First Peak > 500 RFU                Upper Marker Selection: Last Peak > 500 RFU
Ladder Size (bp)35, 100, 200, 300, 400, 500, 600, 700, 800, 900, 1000, 1500
Quantification Using: Upper Marker          Final Concentration (ng/uL): 0.5000          Dilution Factor: 12.0
```

 Copyright 2015 Advanced Analytical Technologies, Inc.

Sample: SampE3
Well Location: E3

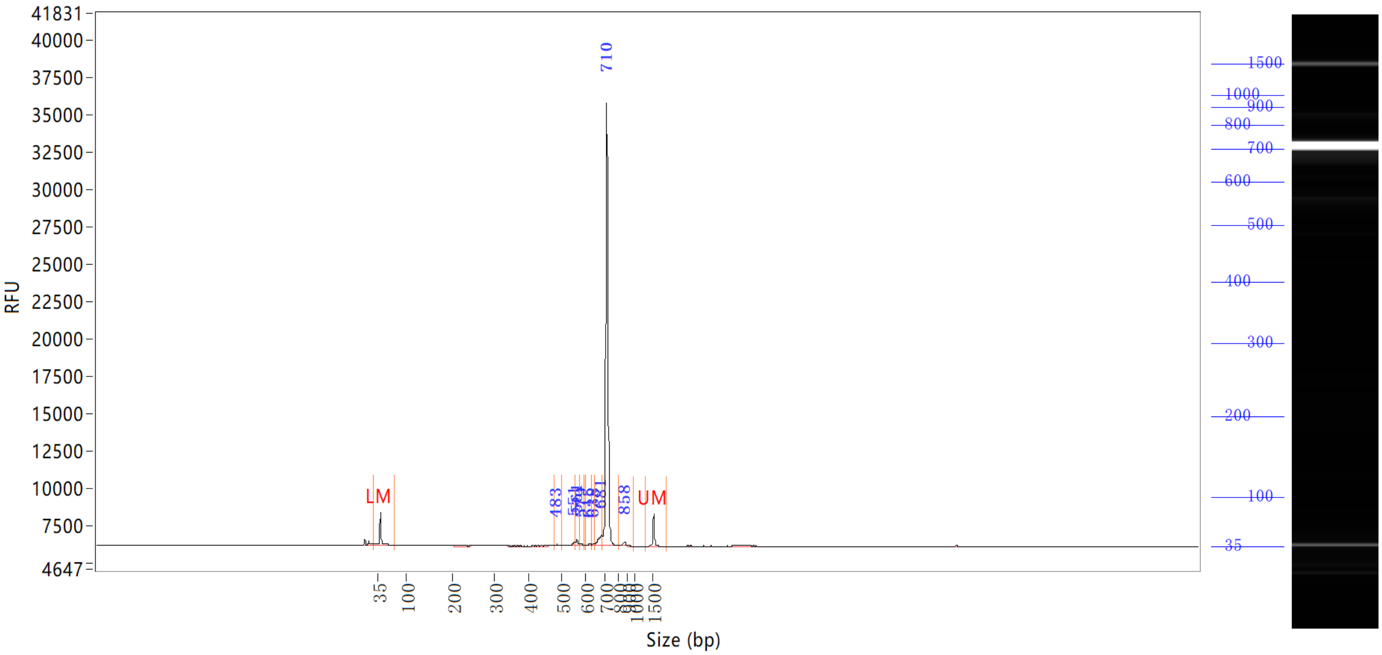

Peak	Size (bp)	Conc. (ng/uL)	From (bp)	To (bp)	Avg. Size (bp)	CV%	RFU	Corr. Peak Area
1	35 (LM)	0.9970	20	72	36	20.77	2181	15.834
2	483	0.4645	475	502	485	1.07	122	0.615
3	551	1.3142	502	554	542	2.05	215	1.739
4	561	1.4796	554	575	561	0.95	384	1.958
5	579	0.3536	575	591	579	0.55	98	0.468
6	615	0.4138	602	628	615	0.92	105	0.548
7	638	0.2397	628	643	636	0.70	67	0.317
8	681	5.6046	643	687	671	1.64	685	7.417
9	710	110.8871	687	806	710	1.29	29643	146.751
10	858	1.3486	806	968	854	2.35	292	1.785
11	1500 (UM)	0.5000	1280	1869	1498	3.04	2180	7.941

TIC:	122.1056	ng/uL
TIM:	286.0165	nmole/L
Total Conc.:	124.7575	ng/uL

Sample Peak Width (sec): 5 Sample Min Peak Height: 50 Sample Baseline V to V?: Y Sample Baseline V to V pts: 3
Sample Filter: Binomial # of Pts for Filter: 3 Sample Start Region (min): 0 Sample End Region (min): 75
Marker Peak Width (sec): 5 Marker Min Peak Height: 500 Marker Baseline V to V?: Y Marker Baseline V to V pts: 3
Lower Marker Selection: First Peak > 500 RFU Upper Marker Selection: Last Peak > 500 RFU
Ladder Size (bp)35, 100, 200, 300, 400, 500, 600, 700, 800, 900, 1000, 1500
Quantification Using: Upper Marker Final Concentration (ng/uL): 0.5000 Dilution Factor: 12.0

 Copyright 2015 Advanced Analytical Technologies, Inc.

Sample: SampF3
Well Location: F3

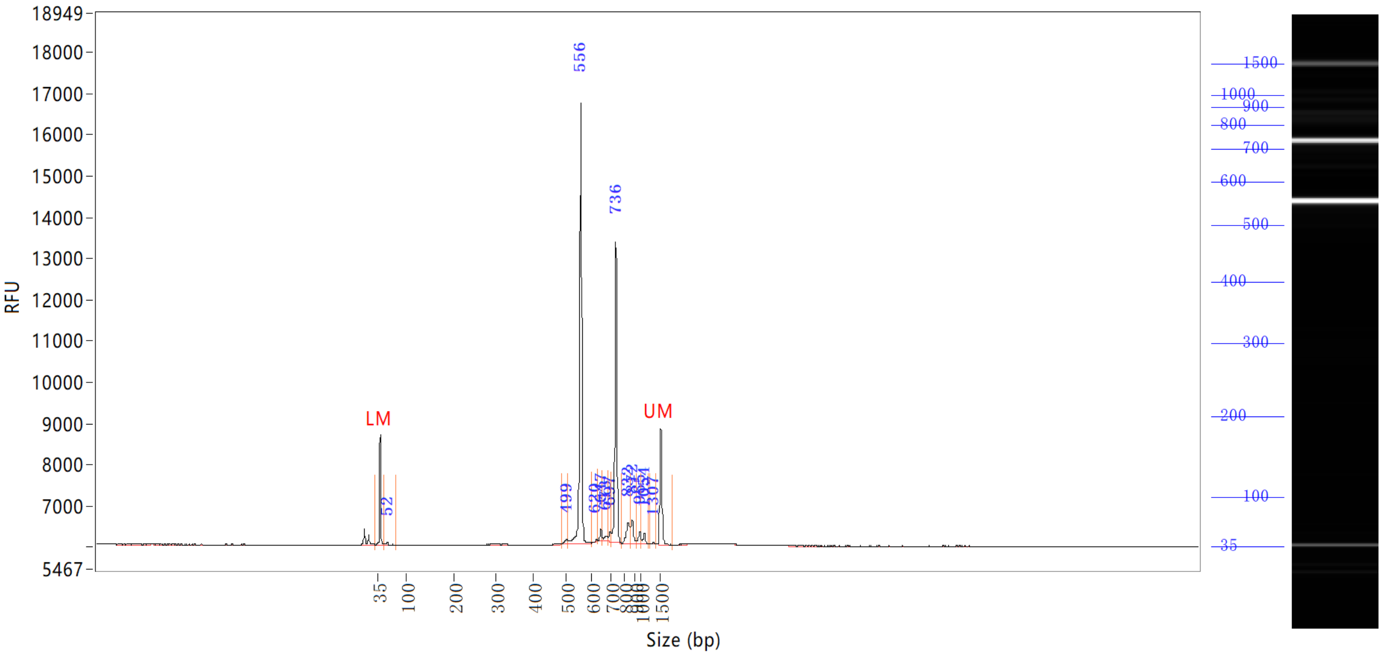

Peak	Size (bp)	Conc. (ng/uL)	From (bp)	To (bp)	Avg. Size (bp)	CV%	RFU	Corr. Peak Area
1	35 (LM)	0.5344	24	45	35	5.83	2692	12.981
2	52	0.3781	45	72	53	11.04	62	0.765
3	499	0.5148	488	508	499	1.01	122	1.042
4	556	25.5709	508	599	553	1.35	10704	51.760
5	620	0.0964	599	631	620	1.02	56	0.195
6	647	0.6465	631	659	646	0.84	275	1.309
7	673	0.4632	659	684	672	0.99	129	0.938
8	697	0.7874	684	706	696	0.89	246	1.594
9	736	16.7729	706	782	733	1.11	7275	33.952
10	832	2.0274	782	855	829	2.00	488	4.104
11	872	1.6833	855	922	874	1.60	579	3.407
12	965	0.8338	922	1000	961	1.86	312	1.688
13	1054	0.6198	1000	1167	1049	2.75	256	1.255
14	1307	0.1518	1234	1400	1313	2.89	52	0.307
15	1500 (UM)	0.5000	1400	1842	1500	2.26	2821	12.145

TIC:	50.5464	ng/uL
TIM:	141.8228	nmole/L
Total Conc.:	52.2817	ng/uL

```
Sample Peak Width (sec): 5    Sample Min Peak Height: 50    Sample Baseline V to V?: Y    Sample Baseline V to V pts: 3
Sample Filter: Binomial       # of Pts for Filter: 3        Sample Start Region (min): 0  Sample End Region (min): 75
Marker Peak Width (sec): 5    Marker Min Peak Height: 500   Marker Baseline V to V?: Y    Marker Baseline V to V pts: 3
Lower Marker Selection: First Peak > 500 RFU                Upper Marker Selection: Last Peak > 500 RFU
Ladder Size (bp)35, 100, 200, 300, 400, 500, 600, 700, 800, 900, 1000, 1500
Quantification Using: Upper Marker          Final Concentration (ng/uL): 0.5000          Dilution Factor: 12.0
```

 Copyright 2015 Advanced Analytical Technologies, Inc.

Sample: SampG3
Well Location: G3

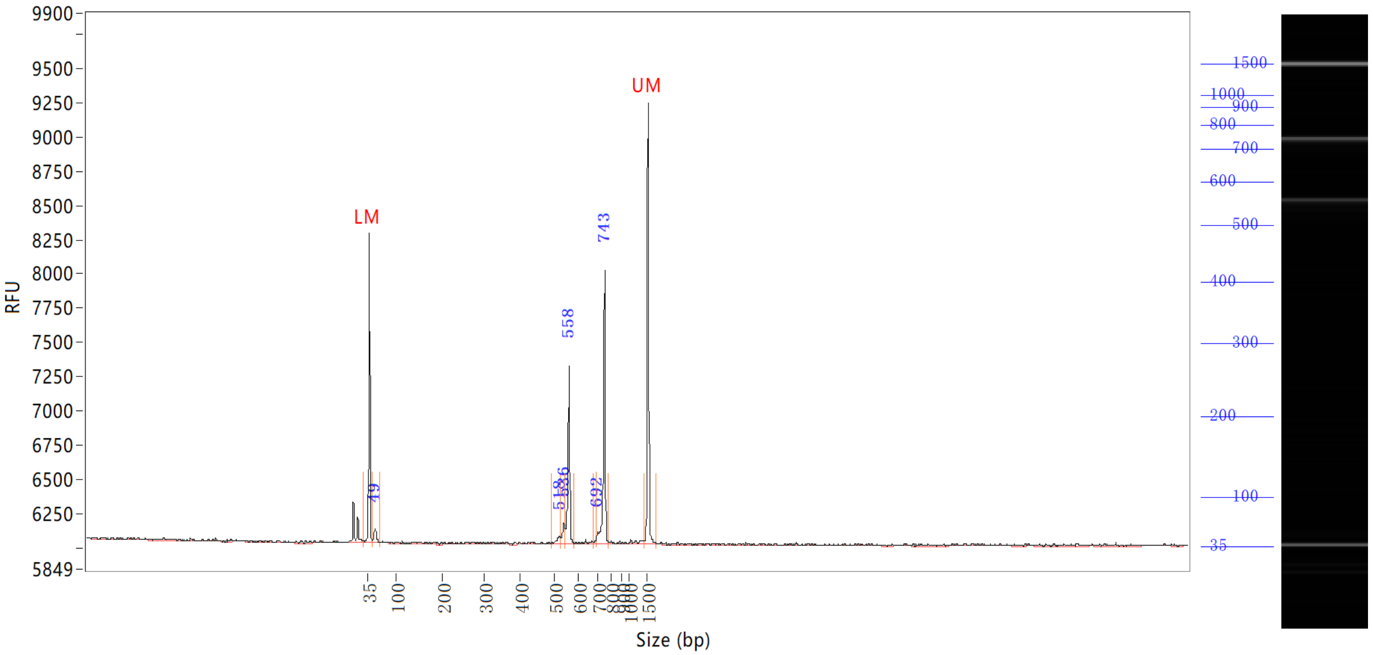

Peak	Size (bp)	Conc. (ng/uL)	From (bp)	To (bp)	Avg. Size (bp)	CV%	RFU	Corr. Peak Area
1	35 (LM)	0.5144	24	43	35	5.09	2257	10.899
2	49	0.6123	43	63	50	6.82	98	1.081
3	518	0.2888	494	526	514	1.51	56	0.510
4	536	0.5293	526	542	535	0.83	151	0.935
5	558	3.1936	542	579	555	0.85	1291	5.639
6	692	0.1333	675	694	687	0.79	65	0.235
7	743	4.9374	694	774	737	1.58	1984	8.718
8	1500 (UM)	0.5000	1414	1730	1494	1.80	3217	10.594

TIC:	9.6946	ng/uL
TIM:	43.4228	nmole/L
Total Conc.:	12.0594	ng/uL

```
Sample Peak Width (sec): 5     Sample Min Peak Height: 50    Sample Baseline V to V?: Y    Sample Baseline V to V pts: 3
Sample Filter: Binomial        # of Pts for Filter: 3        Sample Start Region (min): 0  Sample End Region (min): 75
Marker Peak Width (sec): 5     Marker Min Peak Height: 500   Marker Baseline V to V?: Y    Marker Baseline V to V pts: 3
Lower Marker Selection: First Peak > 500 RFU                 Upper Marker Selection: Last Peak > 500 RFU
Ladder Size (bp)35, 100, 200, 300, 400, 500, 600, 700, 800, 900, 1000, 1500
Quantification Using: Upper Marker          Final Concentration (ng/uL): 0.5000          Dilution Factor: 12.0
```

 Copyright 2015 Advanced Analytical Technologies, Inc.

Sample: SampH3
Well Location: H3

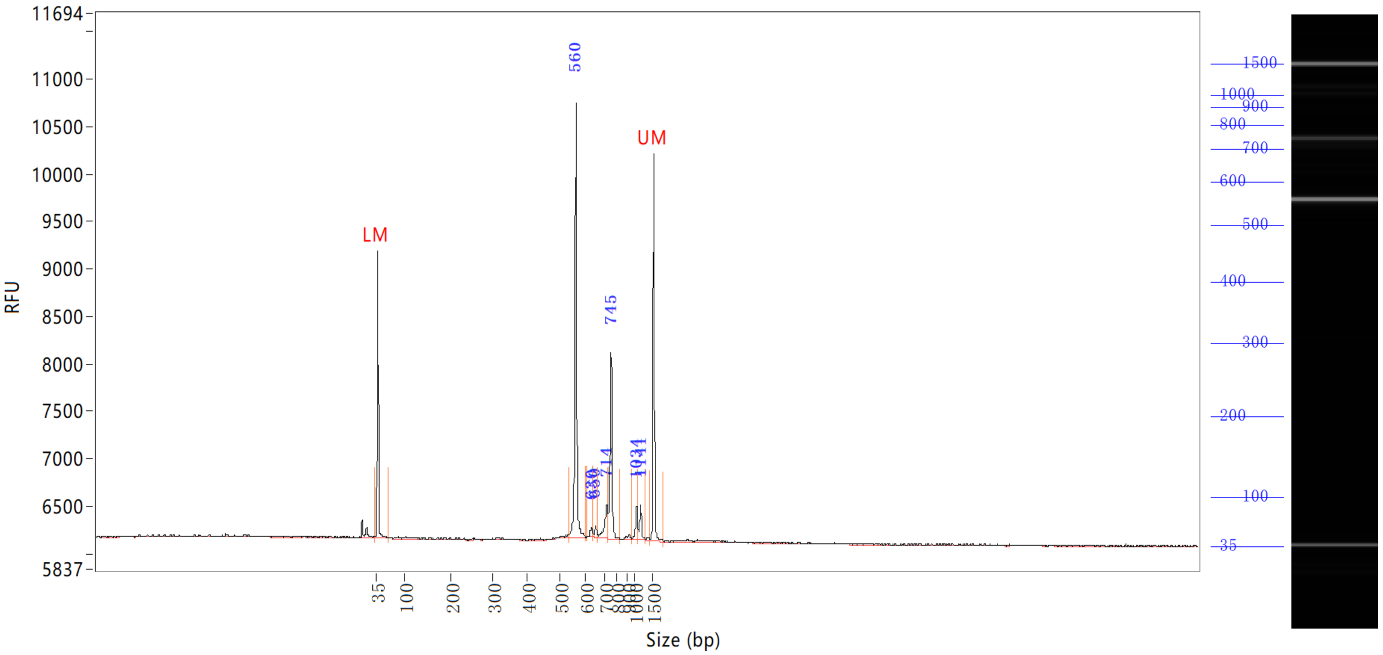

Peak	Size (bp)	Conc. (ng/uL)	From (bp)	To (bp)	Avg. Size (bp)	CV%	RFU	Corr. Peak Area
1	35 (LM)	0.5304	29	62	35	9.03	3032	14.641
2	560	9.0257	530	600	558	1.16	4586	20.760
3	630	0.2366	609	640	628	0.90	107	0.544
4	650	0.2539	640	666	651	0.89	125	0.584
5	714	1.1928	666	725	705	2.01	360	2.744
6	745	3.9975	725	825	744	1.43	1964	9.195
7	1034	0.6895	954	1081	1023	2.57	350	1.586
8	1141	0.7216	1081	1294	1138	3.22	371	1.660
9	1500 (UM)	0.5000	1414	1783	1498	2.23	4075	13.801

TIC:	16.1175	ng/uL
TIM:	41.6596	nmole/L
Total Conc.:	17.8106	ng/uL

```
Sample Peak Width (sec): 5     Sample Min Peak Height: 50    Sample Baseline V to V?: Y    Sample Baseline V to V pts: 3
Sample Filter: Binomial        # of Pts for Filter: 3        Sample Start Region (min): 0  Sample End Region (min): 75
Marker Peak Width (sec): 5     Marker Min Peak Height: 500   Marker Baseline V to V?: Y    Marker Baseline V to V pts: 3
Lower Marker Selection: First Peak > 500 RFU                 Upper Marker Selection: Last Peak > 500 RFU
Ladder Size (bp)35, 100, 200, 300, 400, 500, 600, 700, 800, 900, 1000, 1500
Quantification Using: Upper Marker          Final Concentration (ng/uL): 0.5000          Dilution Factor: 12.0
```

 Copyright 2015 Advanced Analytical Technologies, Inc.

Sample: SampA4
Well Location: A4

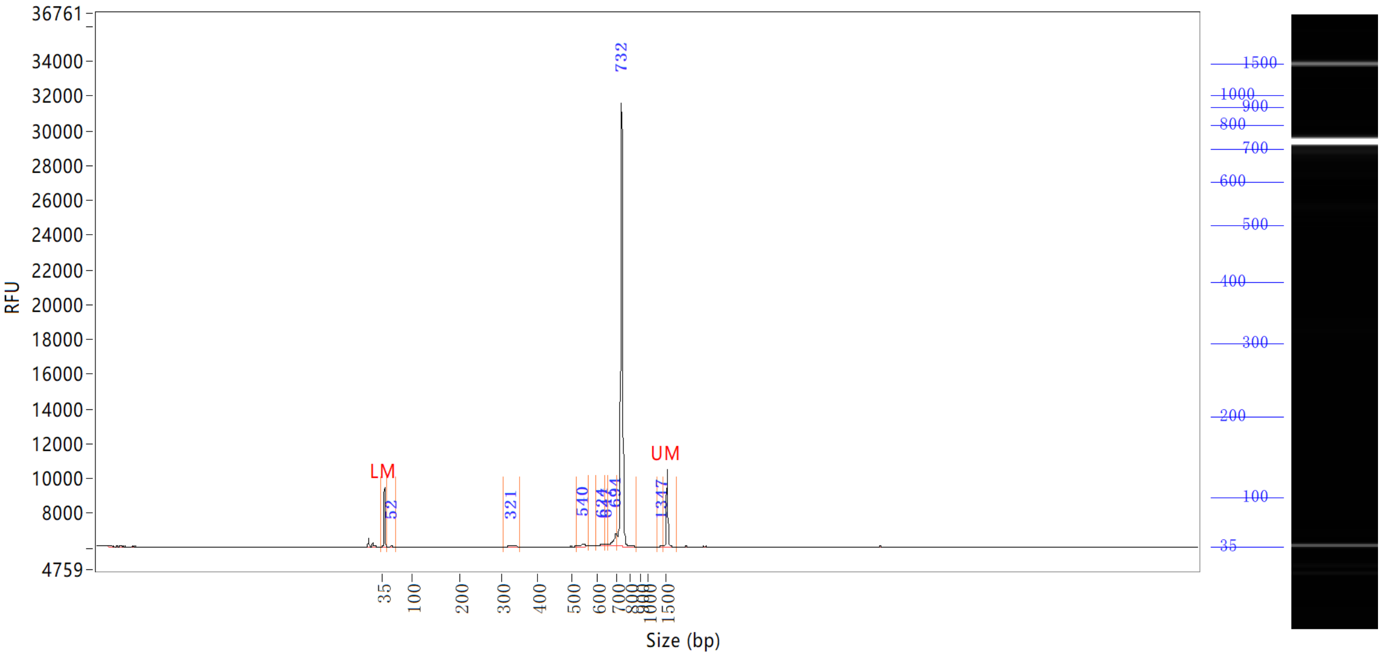

Peak	Size (bp)	Conc. (ng/uL)	From (bp)	To (bp)	Avg. Size (bp)	CV%	RFU	Corr. Peak Area
1	35 (LM)	0.5126	27	43	35	4.27	3413	15.571
2	52	0.2823	43	62	51	7.55	62	0.715
3	321	0.5684	302	350	323	3.04	64	1.439
4	540	0.6417	518	565	541	1.63	169	1.624
5	624	0.5334	595	642	623	1.65	136	1.350
6	647	0.1819	642	654	648	0.61	94	0.460
7	694	2.7661	654	705	688	1.72	744	7.002
8	732	45.5547	705	862	730	1.27	25548	115.308
9	1347	0.1257	1234	1400	1339	2.68	59	0.318
10	1500 (UM)	0.5000	1400	1757	1496	1.84	4458	15.187

TIC:	50.6541	ng/uL
TIM:	125.2573	nmole/L
Total Conc.:	51.9442	ng/uL

```
Sample Peak Width (sec): 5    Sample Min Peak Height: 50    Sample Baseline V to V?: Y    Sample Baseline V to V pts: 3
Sample Filter: Binomial       # of Pts for Filter: 3        Sample Start Region (min): 0  Sample End Region (min): 75
Marker Peak Width (sec): 5    Marker Min Peak Height: 500   Marker Baseline V to V?: Y    Marker Baseline V to V pts: 3
Lower Marker Selection: First Peak > 500 RFU                Upper Marker Selection: Last Peak > 500 RFU
Ladder Size (bp)35, 100, 200, 300, 400, 500, 600, 700, 800, 900, 1000, 1500
Quantification Using: Upper Marker          Final Concentration (ng/uL): 0.5000          Dilution Factor: 12.0
```


Copyright 2015 Advanced Analytical Technologies, Inc.

Sample: SampB4
Well Location: B4

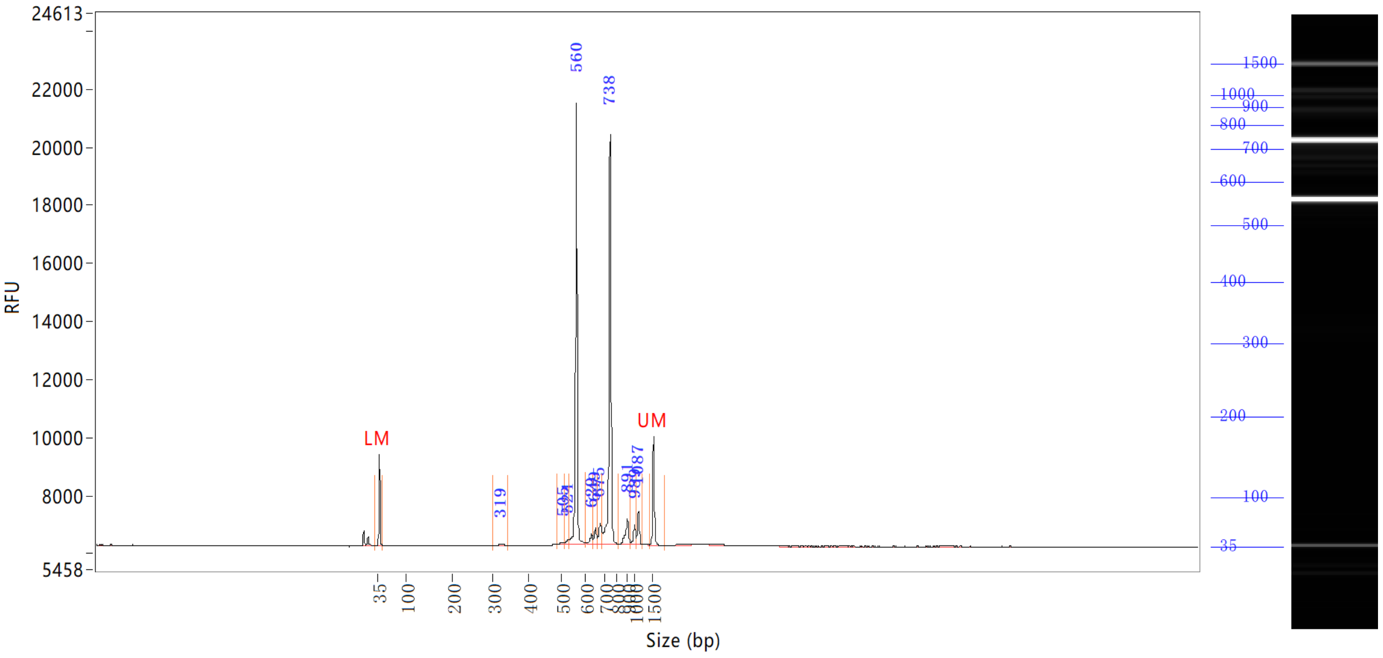

Peak	Size (bp)	Conc. (ng/uL)	From (bp)	To (bp)	Avg. Size (bp)	CV%	RFU	Corr. Peak Area
1	35 (LM)	0.5252	25	45	35	5.02	3145	14.941
2	319	0.6086	298	340	321	3.21	59	1.443
3	505	0.1925	490	513	502	1.11	59	0.456
4	524	0.3671	513	530	523	0.86	138	0.870
5	560	27.9753	530	600	558	0.99	15196	66.322
6	629	0.8108	600	638	626	1.19	318	1.922
7	649	1.1810	638	658	648	0.80	565	2.800
8	675	2.3675	658	690	675	1.21	711	5.613
9	738	27.4890	690	808	734	1.62	14109	65.169
10	891	2.7736	808	936	880	2.38	898	6.575
11	989	1.4358	936	1021	983	1.84	691	3.404
12	1087	2.0639	1021	1194	1075	2.47	1156	4.893
13	1500 (UM)	0.5000	1394	1829	1498	2.05	3730	14.224

TIC:	67.2651	ng/uL
TIM:	170.6977	nmole/L
Total Conc.:	68.6401	ng/uL

Sample Peak Width (sec): 5   Sample Min Peak Height: 50   Sample Baseline V to V?: Y   Sample Baseline V to V pts: 3
Sample Filter: Binomial   # of Pts for Filter: 3   Sample Start Region (min): 0   Sample End Region (min): 75
Marker Peak Width (sec): 5   Marker Min Peak Height: 500   Marker Baseline V to V?: Y   Marker Baseline V to V pts: 3
Lower Marker Selection: First Peak > 500 RFU   Upper Marker Selection: Last Peak > 500 RFU
Ladder Size (bp)35, 100, 200, 300, 400, 500, 600, 700, 800, 900, 1000, 1500
Quantification Using: Upper Marker   Final Concentration (ng/uL): 0.5000   Dilution Factor: 12.0

 Copyright 2015 Advanced Analytical Technologies, Inc.

Sample: SampC4
Well Location: C4

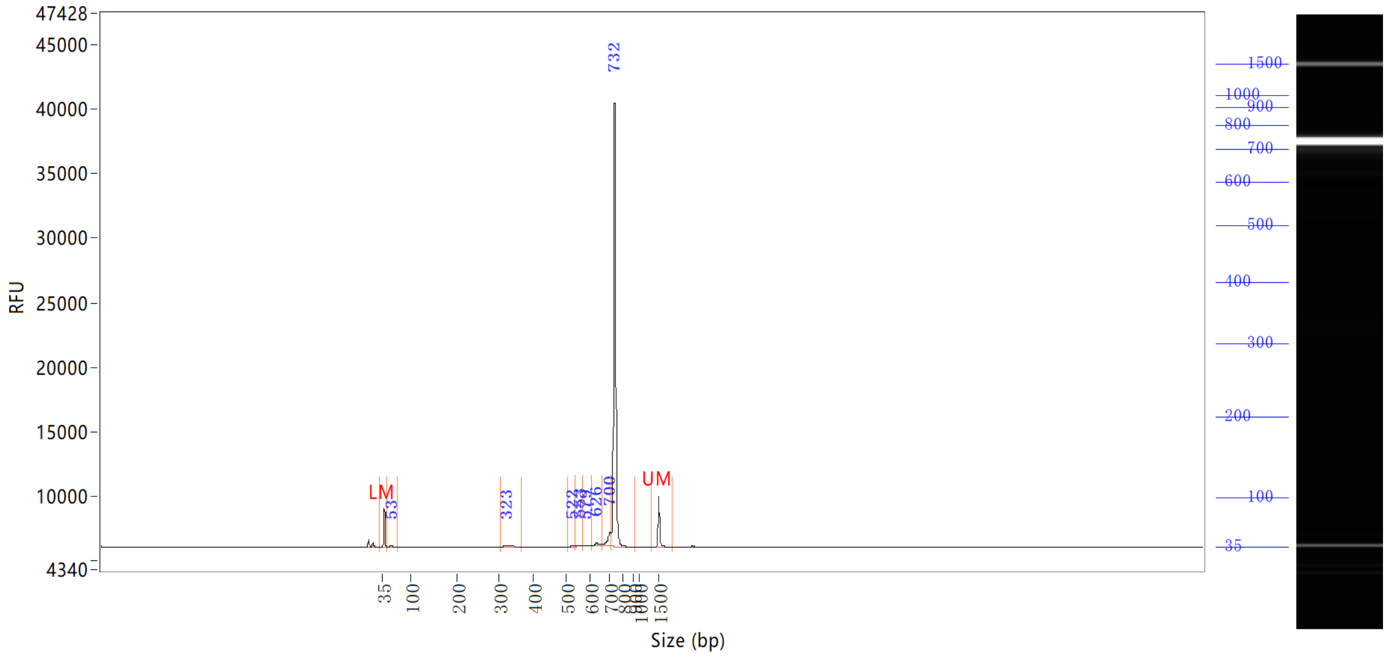

Peak	Size (bp)	Conc. (ng/uL)	From (bp)	To (bp)	Avg. Size (bp)	CV%	RFU	Corr. Peak Area
1	35 (LM)	0.5094	28	44	35	5.53	3043	14.647
2	53	0.7823	44	67	52	8.68	149	1.874
3	323	1.2141	305	363	329	3.60	115	2.909
4	522	0.1958	508	532	522	0.95	74	0.469
5	553	0.4560	532	566	550	1.55	104	1.093
6	579	0.2182	566	601	578	1.41	55	0.523
7	626	1.0004	601	657	632	2.09	212	2.397
8	700	3.9200	657	706	690	1.74	1095	9.393
9	732	68.5522	706	926	731	1.50	34399	164.254
10	1500 (UM)	0.5000	1294	1882	1499	2.96	3864	14.376

TIC:	76.3391	ng/uL
TIM:	199.6129	nmole/L
Total Conc.:	77.3743	ng/uL

```
Sample Peak Width (sec): 5     Sample Min Peak Height: 50    Sample Baseline V to V?: Y    Sample Baseline V to V pts: 3
Sample Filter: Binomial        # of Pts for Filter: 3        Sample Start Region (min): 0  Sample End Region (min): 75
Marker Peak Width (sec): 5     Marker Min Peak Height: 500   Marker Baseline V to V?: Y    Marker Baseline V to V pts: 3
Lower Marker Selection: First Peak > 500 RFU                 Upper Marker Selection: Last Peak > 500 RFU
Ladder Size (bp)35, 100, 200, 300, 400, 500, 600, 700, 800, 900, 1000, 1500
Quantification Using: Upper Marker          Final Concentration (ng/uL): 0.5000          Dilution Factor: 12.0
```

 Copyright 2015 Advanced Analytical Technologies, Inc.

Sample: SampD4
Well Location: D4

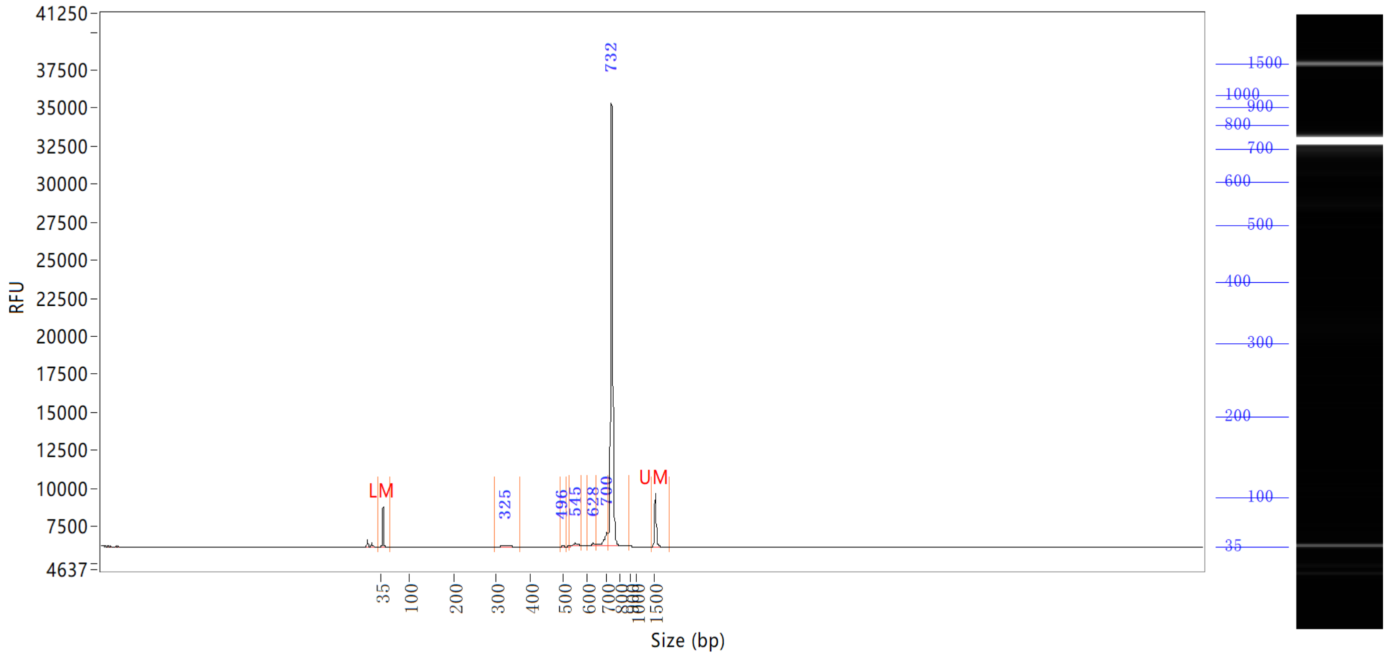

Peak	Size (bp)	Conc. (ng/uL)	From (bp)	To (bp)	Avg. Size (bp)	CV%	RFU	Corr. Peak Area
1	35 (LM)	0.5179	23	55	35	9.59	2620	13.588
2	325	1.2693	294	367	325	4.22	99	2.775
3	496	0.1212	488	510	496	0.77	53	0.265
4	545	1.0677	526	572	547	1.76	212	2.334
5	628	0.7046	595	648	627	1.85	163	1.540
6	700	3.9155	648	708	689	1.98	869	8.560
7	732	64.6247	708	895	734	1.39	29207	141.281
8	1500 (UM)	0.5000	1420	1921	1503	2.83	3484	13.117

TIC:	71.7031	ng/uL
TIM:	166.1429	nmole/L
Total Conc.:	73.3105	ng/uL

Sample Peak Width (sec): 5   Sample Min Peak Height: 50   Sample Baseline V to V?: Y   Sample Baseline V to V pts: 3
Sample Filter: Binomial   # of Pts for Filter: 3   Sample Start Region (min): 0   Sample End Region (min): 75
Marker Peak Width (sec): 5   Marker Min Peak Height: 500   Marker Baseline V to V?: Y   Marker Baseline V to V pts: 3
Lower Marker Selection: First Peak > 500 RFU   Upper Marker Selection: Last Peak > 500 RFU
Ladder Size (bp)35, 100, 200, 300, 400, 500, 600, 700, 800, 900, 1000, 1500
Quantification Using: Upper Marker   Final Concentration (ng/uL): 0.5000   Dilution Factor: 12.0

 Copyright 2015 Advanced Analytical Technologies, Inc.

Sample: SampE4
Well Location: E4

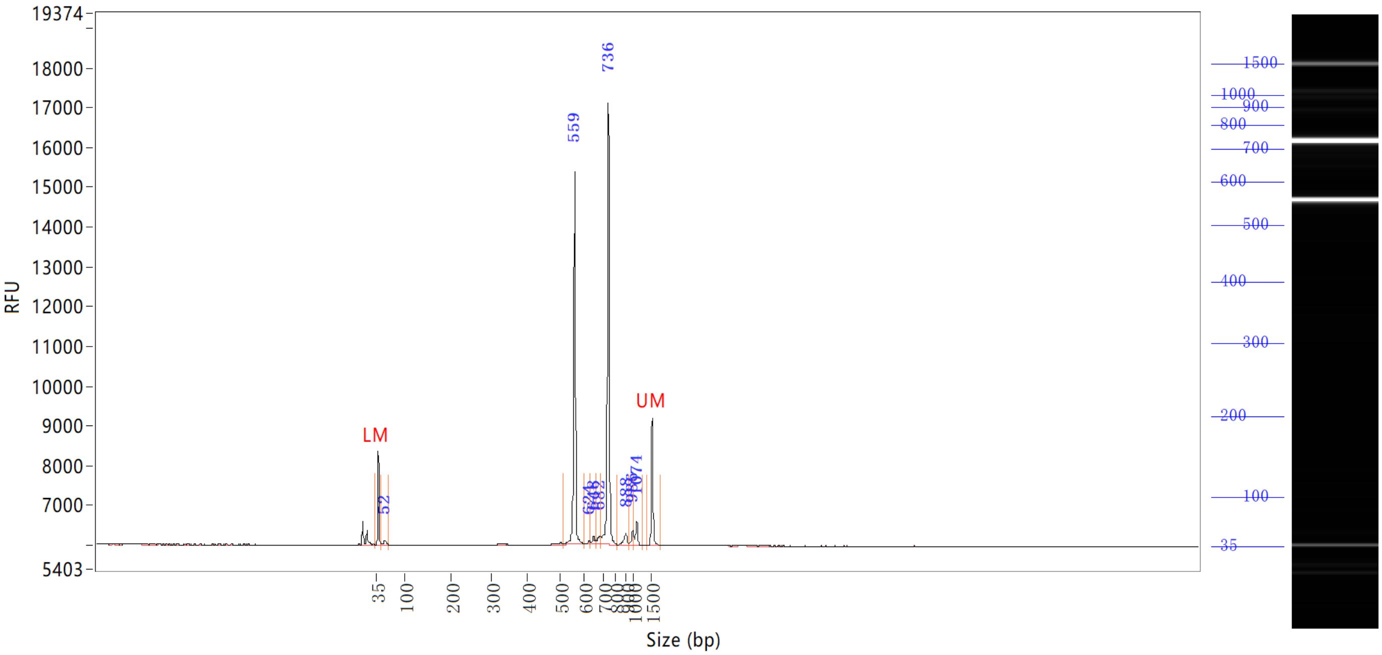

Peak	Size (bp)	Conc. (ng/uL)	From (bp)	To (bp)	Avg. Size (bp)	CV%	RFU	Corr. Peak Area
1	35 (LM)	0.4926	28	45	35	5.39	2339	11.190
2	52	0.6521	45	62	51	6.37	120	1.234
3	559	21.3247	516	597	556	1.12	9372	40.366
4	624	0.2466	597	634	621	1.33	69	0.467
5	648	0.6380	634	662	649	1.11	187	1.208
6	682	0.8881	662	689	677	1.11	208	1.681
7	736	25.9090	689	801	733	1.48	11115	49.043
8	888	1.1332	801	929	878	2.34	292	2.145
9	986	0.9970	929	1014	979	1.86	384	1.887
10	1074	1.3707	1014	1241	1067	2.88	590	2.595
11	1500 (UM)	0.5000	1387	1730	1500	2.06	3211	11.357

TIC:	53.1595	ng/uL
TIM:	152.5775	nmole/L
Total Conc.:	55.6058	ng/uL

Sample Peak Width (sec): 5   Sample Min Peak Height: 50   Sample Baseline V to V?: Y   Sample Baseline V to V pts: 3
Sample Filter: Binomial   # of Pts for Filter: 3   Sample Start Region (min): 0   Sample End Region (min): 75
Marker Peak Width (sec): 5   Marker Min Peak Height: 500   Marker Baseline V to V?: Y   Marker Baseline V to V pts: 3
Lower Marker Selection: First Peak > 500 RFU   Upper Marker Selection: Last Peak > 500 RFU
Ladder Size (bp)35, 100, 200, 300, 400, 500, 600, 700, 800, 900, 1000, 1500
Quantification Using: Upper Marker   Final Concentration (ng/uL): 0.5000   Dilution Factor: 12.0

 Copyright 2015 Advanced Analytical Technologies, Inc.

Sample: SampF4
Well Location: F4

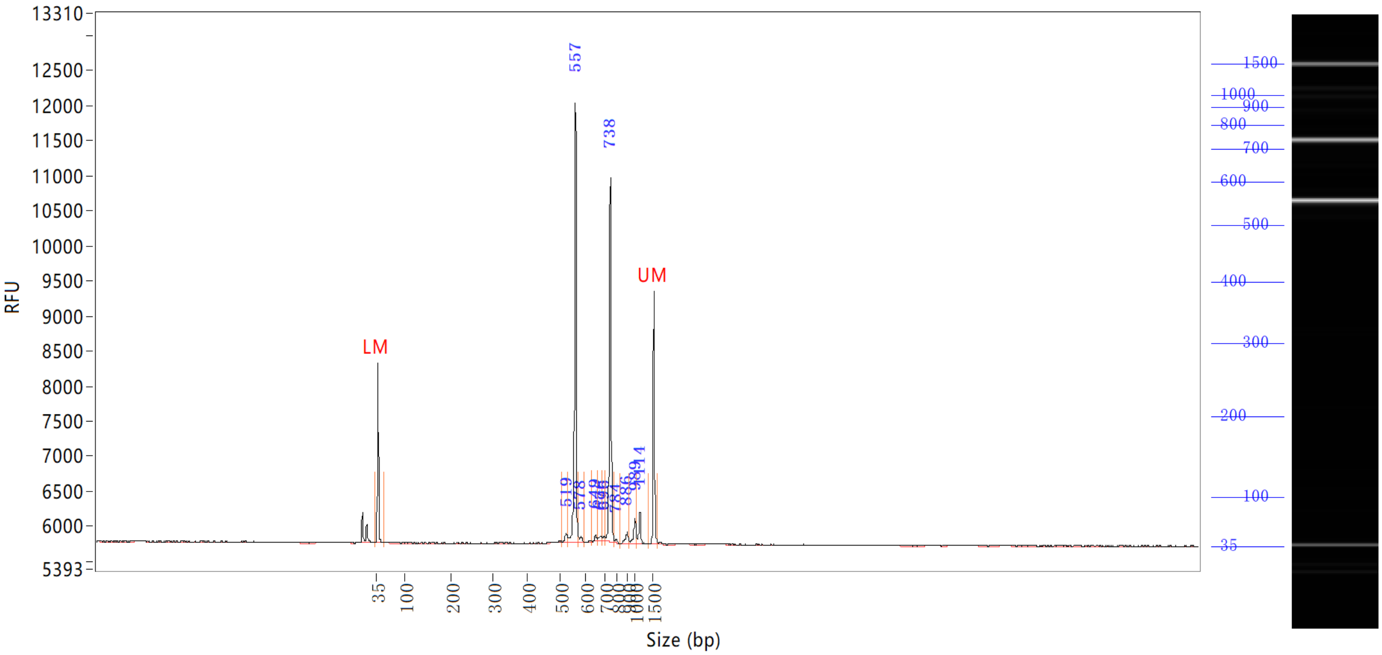

Peak	Size (bp)	Conc. (ng/uL)	From (bp)	To (bp)	Avg. Size (bp)	CV%	RFU	Corr. Peak Area
1	35 (LM)	0.4990	30	51	35	5.29	2568	12.099
2	519	0.4757	502	529	519	1.15	126	0.961
3	557	13.1119	529	573	554	0.92	6271	26.493
4	578	0.2551	573	597	579	0.75	89	0.515
5	649	0.2337	633	659	648	0.89	99	0.472
6	676	0.2878	659	686	674	1.11	68	0.581
7	696	0.2025	686	706	696	0.84	63	0.409
8	738	10.3588	706	769	736	1.01	5179	20.931
9	784	0.1390	769	815	782	1.08	51	0.281
10	886	0.5565	815	919	877	2.05	172	1.124
11	989	0.9098	919	1041	983	2.34	366	1.838
12	1114	0.9662	1041	1360	1109	3.91	440	1.952
13	1500 (UM)	0.5000	1360	1612	1491	1.54	3607	12.123

TIC:	27.4968	ng/uL
TIM:	70.4130	nmole/L
Total Conc.:	29.2206	ng/uL

Sample Peak Width (sec): 5 Sample Min Peak Height: 50 Sample Baseline V to V?: Y Sample Baseline V to V pts: 3
Sample Filter: Binomial # of Pts for Filter: 3 Sample Start Region (min): 0 Sample End Region (min): 75
Marker Peak Width (sec): 5 Marker Min Peak Height: 500 Marker Baseline V to V?: Y Marker Baseline V to V pts: 3
Lower Marker Selection: First Peak > 500 RFU Upper Marker Selection: Last Peak > 500 RFU
Ladder Size (bp)35, 100, 200, 300, 400, 500, 600, 700, 800, 900, 1000, 1500
Quantification Using: Upper Marker Final Concentration (ng/uL): 0.5000 Dilution Factor: 12.0

 Copyright 2015 Advanced Analytical Technologies, Inc.

Sample: SampG4
Well Location: G4

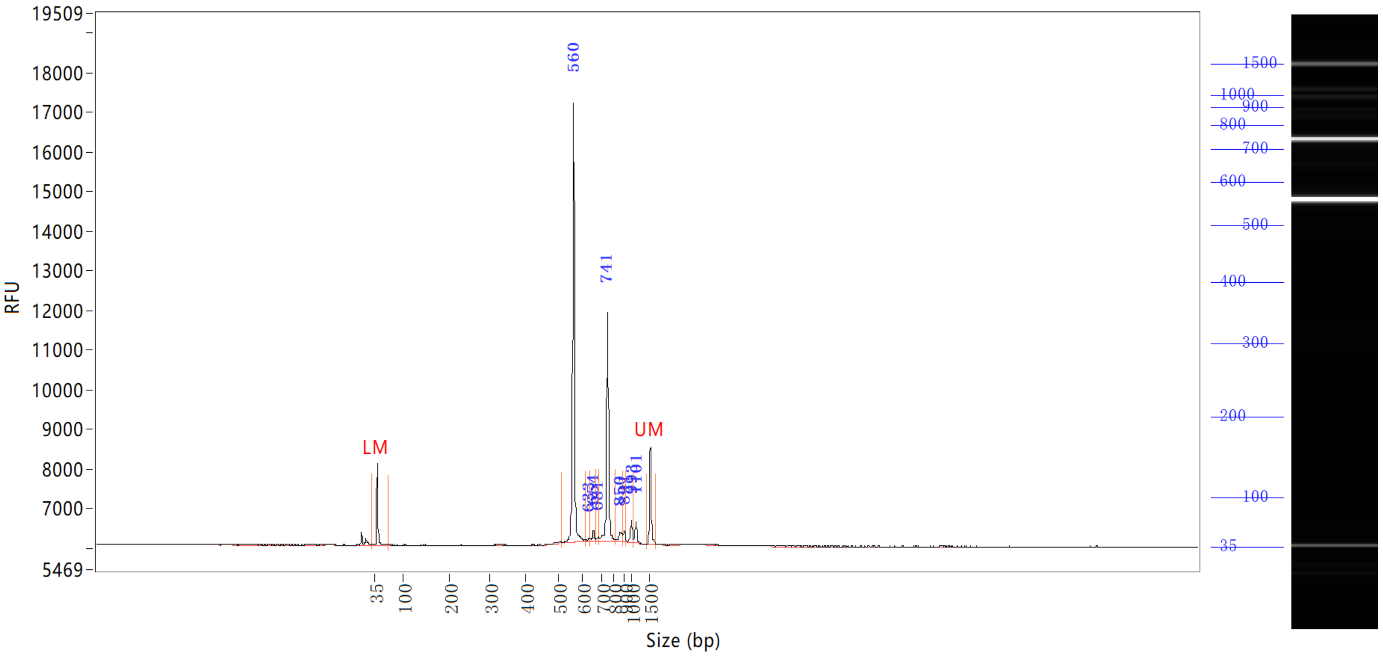

Peak	Size (bp)	Conc. (ng/uL)	From (bp)	To (bp)	Avg. Size (bp)	CV%	RFU	Corr. Peak Area
1	35 (LM)	0.6177	25	63	36	12.84	2075	11.408
2	560	32.9441	513	619	559	1.28	11096	50.700
3	633	0.2377	619	640	632	0.77	73	0.366
4	654	0.7864	640	670	654	0.99	239	1.210
5	681	0.3322	670	689	679	0.78	86	0.511
6	741	16.9605	689	808	740	1.62	5767	26.102
7	850	1.0407	808	874	851	1.68	220	1.602
8	891	0.8289	874	922	888	1.01	280	1.276
9	993	1.6352	922	1034	985	1.92	547	2.516
10	1101	1.5700	1034	1400	1101	4.25	554	2.416
11	1500 (UM)	0.5000	1400	1678	1501	2.24	2451	9.234

TIC:	56.3356	ng/uL
TIM:	146.7337	nmole/L
Total Conc.:	58.9597	ng/uL

```
Sample Peak Width (sec): 5     Sample Min Peak Height: 50     Sample Baseline V to V?: Y     Sample Baseline V to V pts: 3
Sample Filter: Binomial        # of Pts for Filter: 3         Sample Start Region (min): 0   Sample End Region (min): 75
Marker Peak Width (sec): 5     Marker Min Peak Height: 500    Marker Baseline V to V?: Y     Marker Baseline V to V pts: 3
Lower Marker Selection: First Peak > 500 RFU                  Upper Marker Selection: Last Peak > 500 RFU
Ladder Size (bp)35, 100, 200, 300, 400, 500, 600, 700, 800, 900, 1000, 1500
Quantification Using: Upper Marker          Final Concentration (ng/uL): 0.5000          Dilution Factor: 12.0
```

 Copyright 2015 Advanced Analytical Technologies, Inc.

Sample: SampH4
Well Location: H4

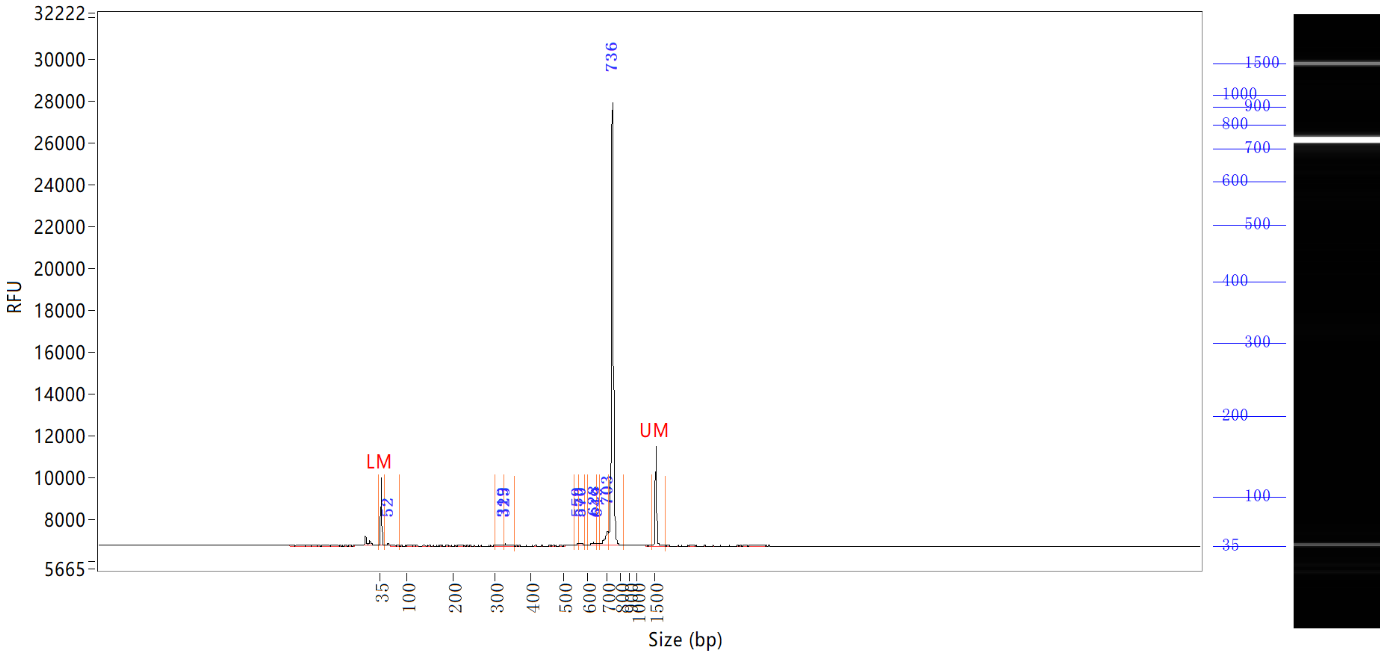

Peak	Size (bp)	Conc. (ng/uL)	From (bp)	To (bp)	Avg. Size (bp)	CV%	RFU	Corr. Peak Area
1	35 (LM)	0.4807	30	45	35	5.23	3233	15.316
2	52	0.5107	45	79	54	14.05	81	1.356
3	319	0.3416	297	323	314	2.06	73	0.907
4	325	0.4139	323	353	333	2.32	79	1.099
5	559	0.1482	547	563	556	0.79	64	0.394
6	570	0.2172	563	587	571	0.89	92	0.577
7	628	0.2914	601	644	626	1.48	103	0.774
8	649	0.1154	644	658	651	0.63	78	0.306
9	703	2.0852	658	710	693	1.75	617	5.537
10	736	36.5618	710	839	736	1.27	21165	97.081
11	1500 (UM)	0.5000	1420	1790	1498	2.22	4744	15.932

TIC:	40.6855	ng/uL
TIM:	108.1599	nmole/L
Total Conc.:	42.0942	ng/uL

```
Sample Peak Width (sec): 5     Sample Min Peak Height: 50    Sample Baseline V to V?: Y     Sample Baseline V to V pts: 3
Sample Filter: Binomial        # of Pts for Filter: 3        Sample Start Region (min): 0  Sample End Region (min): 75
Marker Peak Width (sec): 5     Marker Min Peak Height: 500   Marker Baseline V to V?: Y     Marker Baseline V to V pts: 3
Lower Marker Selection: First Peak > 500 RFU                 Upper Marker Selection: Last Peak > 500 RFU
Ladder Size (bp)35, 100, 200, 300, 400, 500, 600, 700, 800, 900, 1000, 1500
Quantification Using: Upper Marker          Final Concentration (ng/uL): 0.5000          Dilution Factor: 12.0
```

 Copyright 2015 Advanced Analytical Technologies, Inc.

Sample: SampA5
Well Location: A5

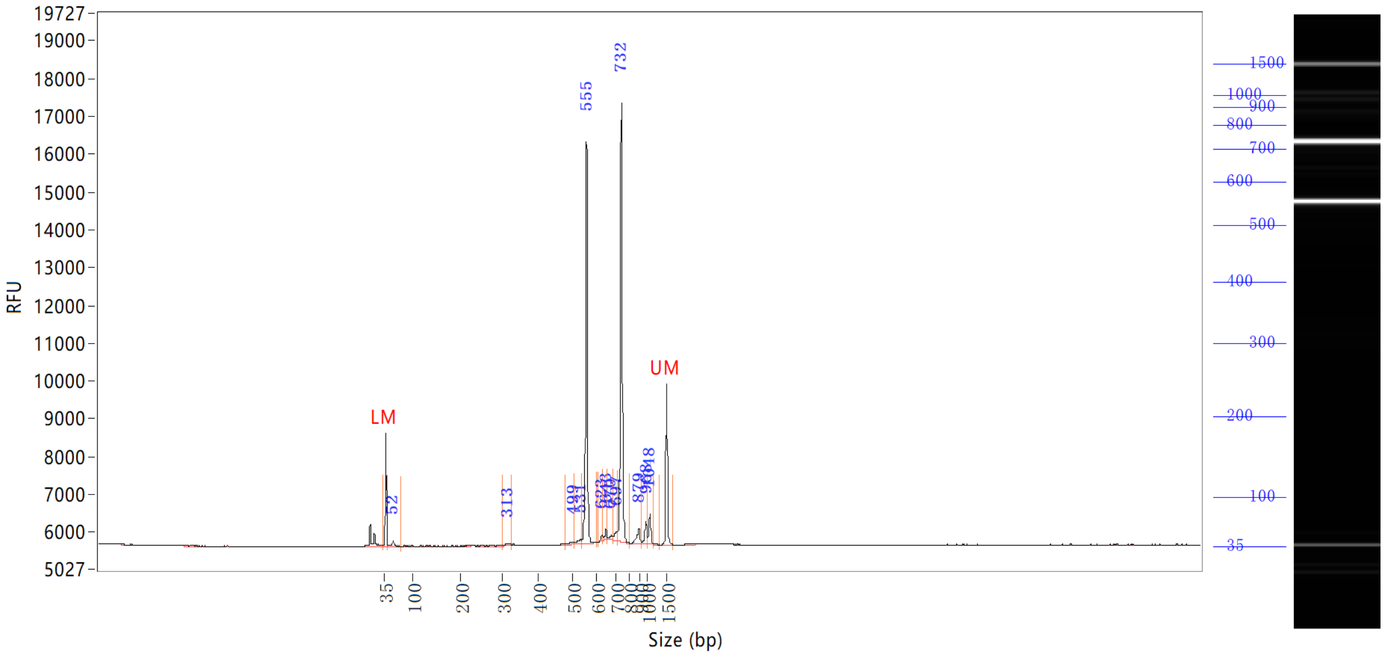

Peak	Size (bp)	Conc. (ng/uL)	From (bp)	To (bp)	Avg. Size (bp)	CV%	RFU	Corr. Peak Area
1	35 (LM)	0.4697	29	42	35	4.34	2972	13.826
2	52	0.5553	42	70	51	9.89	126	1.362
3	313	0.3888	298	323	311	2.24	56	0.954
4	499	0.3610	479	508	495	1.50	73	0.885
5	531	0.4481	508	535	524	1.48	112	1.099
6	555	18.2811	535	595	554	0.85	10632	44.843
7	623	0.3185	604	631	620	0.90	157	0.781
8	643	0.4459	631	653	641	0.67	275	1.094
9	670	0.3214	653	678	668	0.97	117	0.788
10	697	0.7132	678	703	692	0.99	242	1.749
11	732	20.0237	703	798	730	1.16	11627	49.118
12	879	1.2199	798	915	868	2.35	410	2.992
13	968	1.2011	915	996	963	1.82	625	2.946
14	1048	1.3830	996	1167	1038	2.54	797	3.392
15	1500 (UM)	0.5000	1334	1697	1492	1.83	4267	14.718

TIC:	45.6609	ng/uL
TIM:	132.9813	nmole/L
Total Conc.:	46.9763	ng/uL

```
Sample Peak Width (sec): 5     Sample Min Peak Height: 50    Sample Baseline V to V?: Y     Sample Baseline V to V pts: 3
Sample Filter: Binomial        # of Pts for Filter: 3        Sample Start Region (min): 0  Sample End Region (min): 75
Marker Peak Width (sec): 5     Marker Min Peak Height: 500   Marker Baseline V to V?: Y     Marker Baseline V to V pts: 3
Lower Marker Selection: First Peak > 500 RFU                 Upper Marker Selection: Last Peak > 500 RFU
Ladder Size (bp)35, 100, 200, 300, 400, 500, 600, 700, 800, 900, 1000, 1500
Quantification Using: Upper Marker          Final Concentration (ng/uL): 0.5000          Dilution Factor: 12.0
```

 Copyright 2015 Advanced Analytical Technologies, Inc.

Sample: SampB5
Well Location: B5

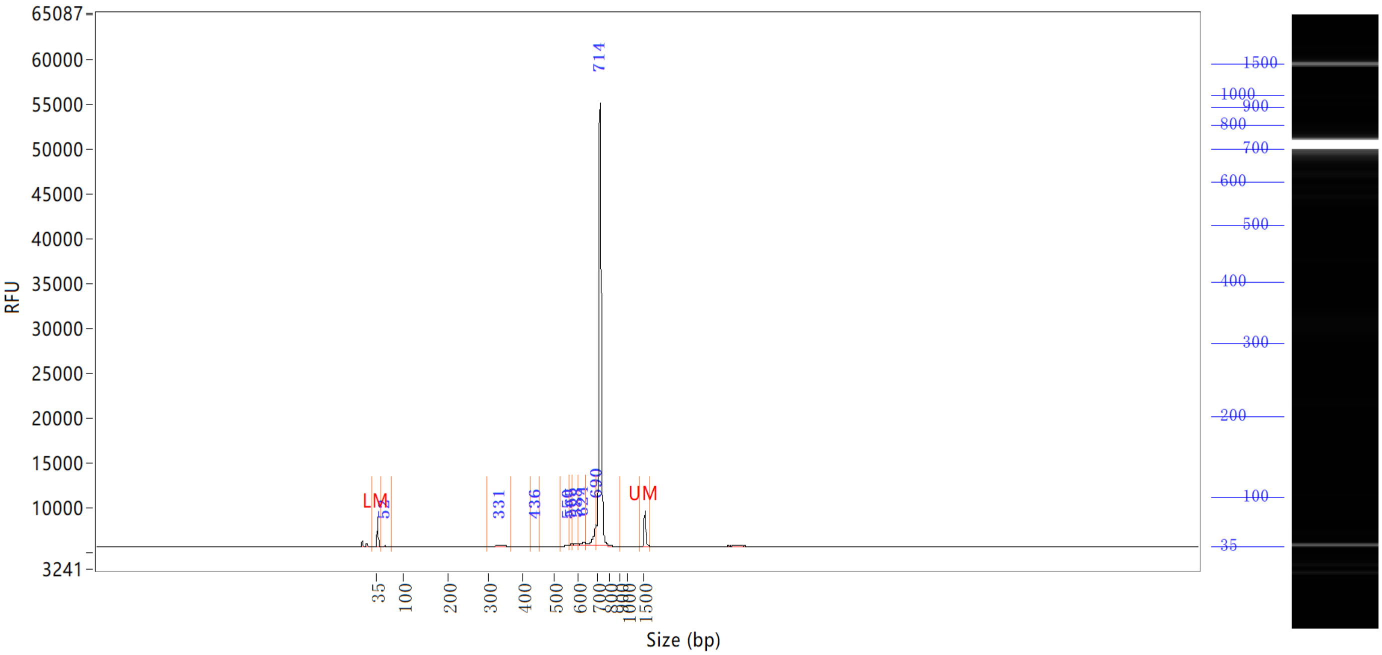

Peak	Size (bp)	Conc. (ng/uL)	From (bp)	To (bp)	Avg. Size (bp)	CV%	RFU	Corr. Peak Area
1	35 (LM)	0.5293	25	45	35	5.95	3272	15.702
2	52	0.4239	45	72	53	10.52	82	1.048
3	331	1.8053	295	364	330	3.77	172	4.463
4	436	0.1428	424	449	435	1.13	61	0.353
5	550	0.2221	521	557	548	1.23	94	0.549
6	565	0.5249	557	575	565	0.82	221	1.298
7	588	0.4462	575	600	587	1.05	140	1.103
8	624	1.0612	600	639	622	1.43	341	2.623
9	690	7.4781	639	695	680	1.84	2308	18.487
10	714	110.4819	695	898	717	1.57	49332	273.125
11	1500 (UM)	0.5000	1374	1665	1497	2.09	3992	14.833

TIC:	122.5865	ng/uL
TIM:	300.5918	nmole/L
Total Conc.:	123.3902	ng/uL

```
Sample Peak Width (sec): 5    Sample Min Peak Height: 50    Sample Baseline V to V?: Y    Sample Baseline V to V pts: 3
Sample Filter: Binomial       # of Pts for Filter: 3        Sample Start Region (min): 0  Sample End Region (min): 75
Marker Peak Width (sec): 5    Marker Min Peak Height: 500   Marker Baseline V to V?: Y    Marker Baseline V to V pts: 3
Lower Marker Selection: First Peak > 500 RFU                Upper Marker Selection: Last Peak > 500 RFU
Ladder Size (bp)35, 100, 200, 300, 400, 500, 600, 700, 800, 900, 1000, 1500
Quantification Using: Upper Marker          Final Concentration (ng/uL): 0.5000          Dilution Factor: 12.0
```

Sample: SampC5
Well Location: C5

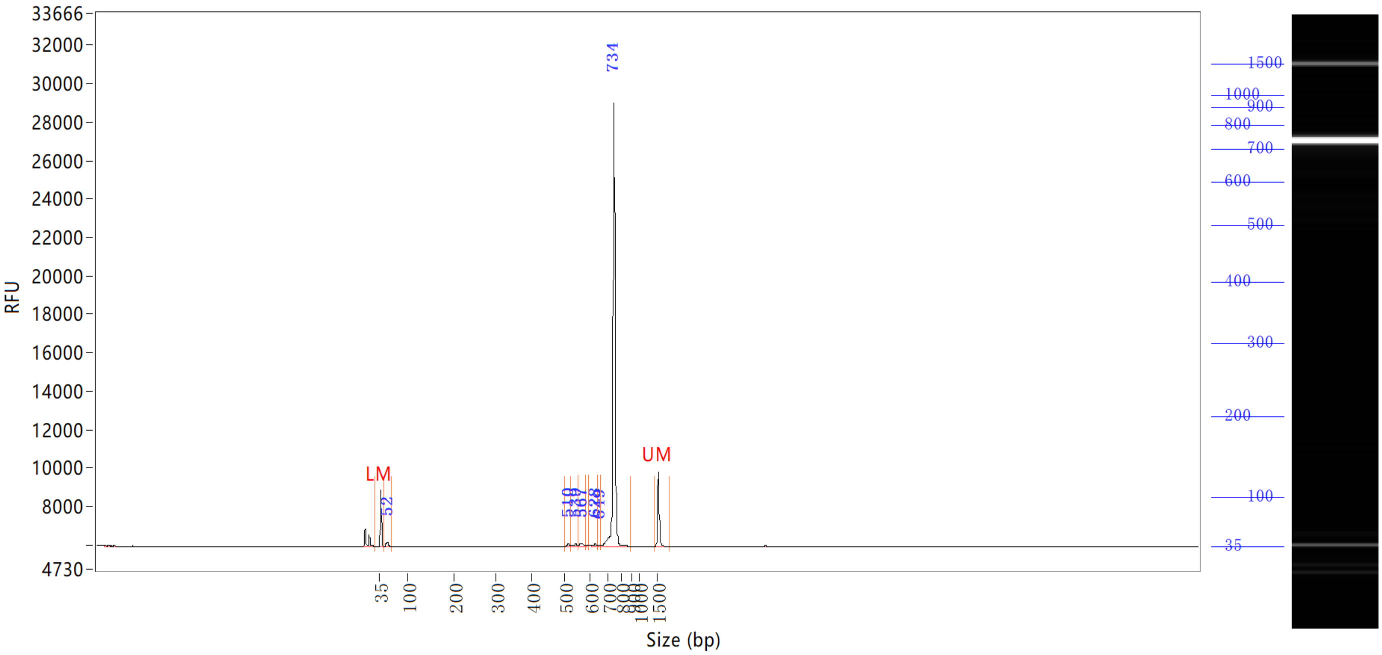

Peak	Size (bp)	Conc. (ng/uL)	From (bp)	To (bp)	Avg. Size (bp)	CV%	RFU	Corr. Peak Area
1	35 (LM)	0.4958	25	43	35	5.39	2926	14.083
2	52	1.1137	43	63	50	6.50	227	2.636
3	510	0.3532	499	521	510	0.96	132	0.836
4	539	0.4780	521	553	538	1.59	106	1.132
5	567	0.4492	553	586	565	1.26	130	1.063
6	628	0.4128	597	644	625	1.69	110	0.977
7	649	0.1062	644	654	649	0.49	64	0.251
8	734	47.2469	654	886	732	1.92	23116	111.848
9	1500 (UM)	0.5000	1420	1816	1501	2.32	3888	14.204

TIC:	50.1599	ng/uL
TIM:	147.9727	nmole/L
Total Conc.:	51.6268	ng/uL

Sample Peak Width (sec): 5   Sample Min Peak Height: 50   Sample Baseline V to V?: Y   Sample Baseline V to V pts: 3
Sample Filter: Binomial   # of Pts for Filter: 3   Sample Start Region (min): 0   Sample End Region (min): 75
Marker Peak Width (sec): 5   Marker Min Peak Height: 500   Marker Baseline V to V?: Y   Marker Baseline V to V pts: 3
Lower Marker Selection: First Peak > 500 RFU   Upper Marker Selection: Last Peak > 500 RFU
Ladder Size (bp)35, 100, 200, 300, 400, 500, 600, 700, 800, 900, 1000, 1500
Quantification Using: Upper Marker   Final Concentration (ng/uL): 0.5000   Dilution Factor: 12.0

 Copyright 2015 Advanced Analytical Technologies, Inc.

Sample: SampD5
Well Location: D5

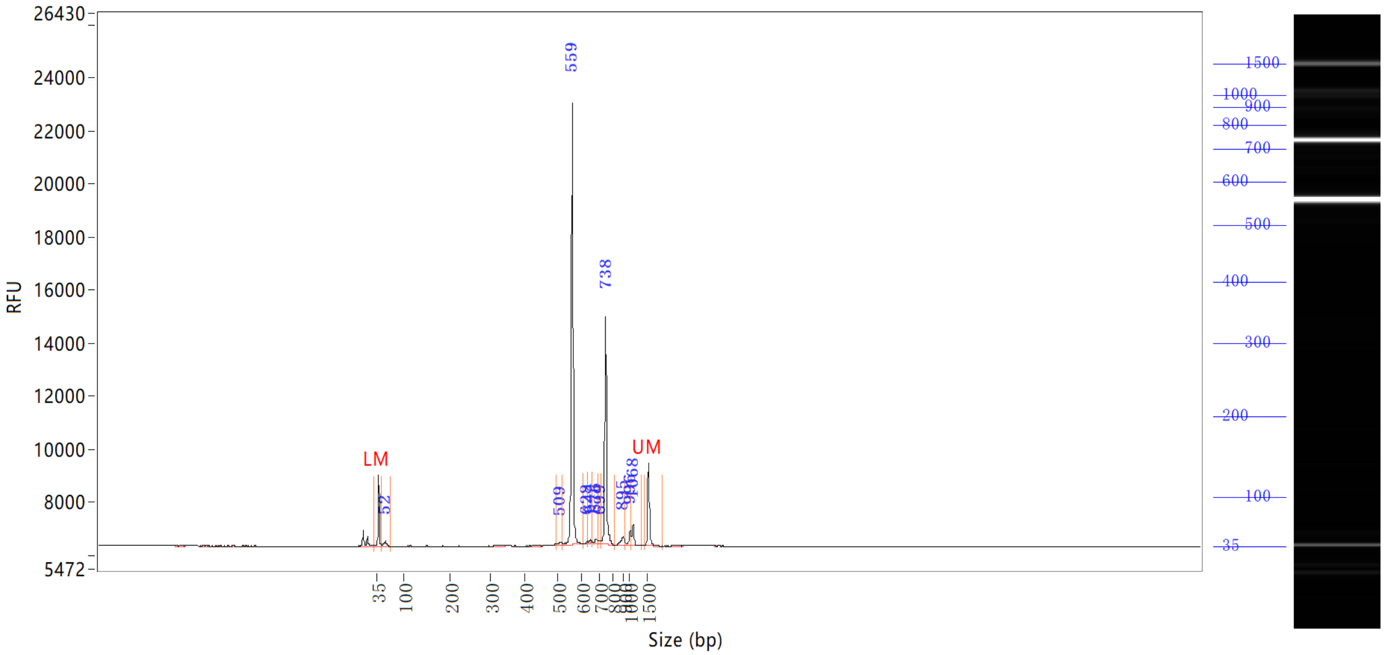

Peak	Size (bp)	Conc. (ng/uL)	From (bp)	To (bp)	Avg. Size (bp)	CV%	RFU	Corr. Peak Area
1	35 (LM)	0.5223	24	45	35	6.78	2672	13.400
2	52	1.0483	45	65	51	7.11	196	2.241
3	509	0.4256	496	518	507	1.13	114	0.910
4	559	34.2298	518	605	558	1.06	16676	73.185
5	628	0.1978	605	635	625	0.89	92	0.423
6	644	0.2919	635	659	645	0.86	137	0.624
7	676	0.5037	659	689	676	1.05	157	1.077
8	699	0.3528	689	706	697	0.73	129	0.754
9	738	17.5326	706	820	738	1.39	8584	37.485
10	895	1.2820	820	933	884	2.51	340	2.741
11	996	1.3306	933	1021	987	2.07	556	2.845
12	1068	1.7319	1021	1320	1070	3.38	783	3.703
13	1500 (UM)	0.5000	1400	1928	1506	2.69	3107	12.828

TIC:	58.9269	ng/uL
TIM:	185.6894	nmole/L
Total Conc.:	60.7020	ng/uL

Sample Peak Width (sec): 5   Sample Min Peak Height: 50   Sample Baseline V to V?: Y   Sample Baseline V to V pts: 3
Sample Filter: Binomial   # of Pts for Filter: 3   Sample Start Region (min): 0   Sample End Region (min): 75
Marker Peak Width (sec): 5   Marker Min Peak Height: 500   Marker Baseline V to V?: Y   Marker Baseline V to V pts: 3
Lower Marker Selection: First Peak > 500 RFU   Upper Marker Selection: Last Peak > 500 RFU
Ladder Size (bp)35, 100, 200, 300, 400, 500, 600, 700, 800, 900, 1000, 1500
Quantification Using: Upper Marker   Final Concentration (ng/uL): 0.5000   Dilution Factor: 12.0

 Copyright 2015 Advanced Analytical Technologies, Inc.

Sample: SampE5
Well Location: E5

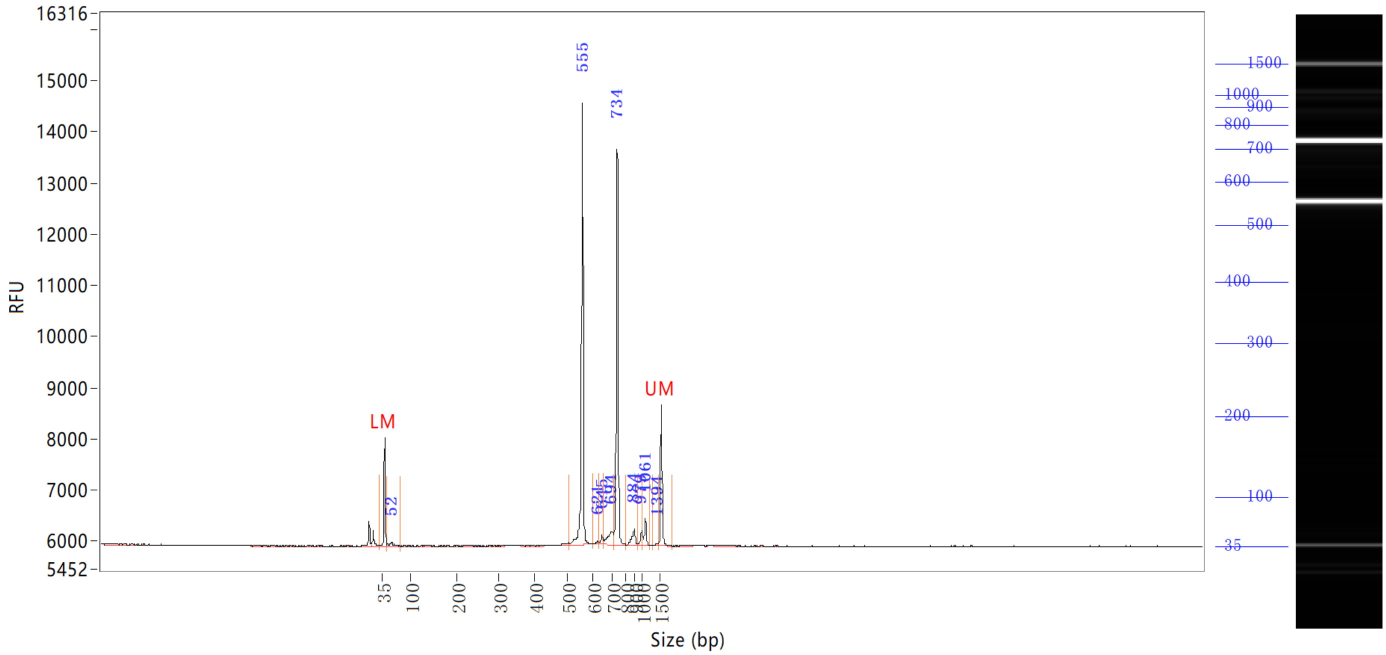

Peak	Size (bp)	Conc. (ng/uL)	From (bp)	To (bp)	Avg. Size (bp)	CV%	RFU	Corr. Peak Area
1	35 (LM)	0.5329	24	44	34	6.08	2112	10.448
2	52	0.7585	44	72	53	11.10	80	1.239
3	555	23.2810	506	602	552	1.41	8645	38.033
4	621	0.2028	602	630	620	0.99	56	0.331
5	645	0.5971	630	656	644	1.00	179	0.976
6	694	2.0791	656	708	686	2.04	262	3.396
7	734	19.4382	708	810	733	1.18	7739	31.755
8	884	1.5198	810	929	871	2.47	314	2.483
9	979	0.8524	929	1001	974	1.71	290	1.393
10	1061	1.3597	1001	1174	1054	2.47	523	2.221
11	1394	0.1900	1267	1427	1368	2.76	51	0.310
12	1500 (UM)	0.5000	1427	1809	1497	2.23	2756	9.802

TIC:	50.2786	ng/uL
TIM:	150.2119	nmole/L
Total Conc.:	52.5476	ng/uL

Sample Peak Width (sec): 5 Sample Min Peak Height: 50 Sample Baseline V to V?: Y Sample Baseline V to V pts: 3
Sample Filter: Binomial # of Pts for Filter: 3 Sample Start Region (min): 0 Sample End Region (min): 75
Marker Peak Width (sec): 5 Marker Min Peak Height: 500 Marker Baseline V to V?: Y Marker Baseline V to V pts: 3
Lower Marker Selection: First Peak > 500 RFU Upper Marker Selection: Last Peak > 500 RFU
Ladder Size (bp)35, 100, 200, 300, 400, 500, 600, 700, 800, 900, 1000, 1500
Quantification Using: Upper Marker Final Concentration (ng/uL): 0.5000 Dilution Factor: 12.0

 Copyright 2015 Advanced Analytical Technologies, Inc.

Sample: SampF5
Well Location: F5

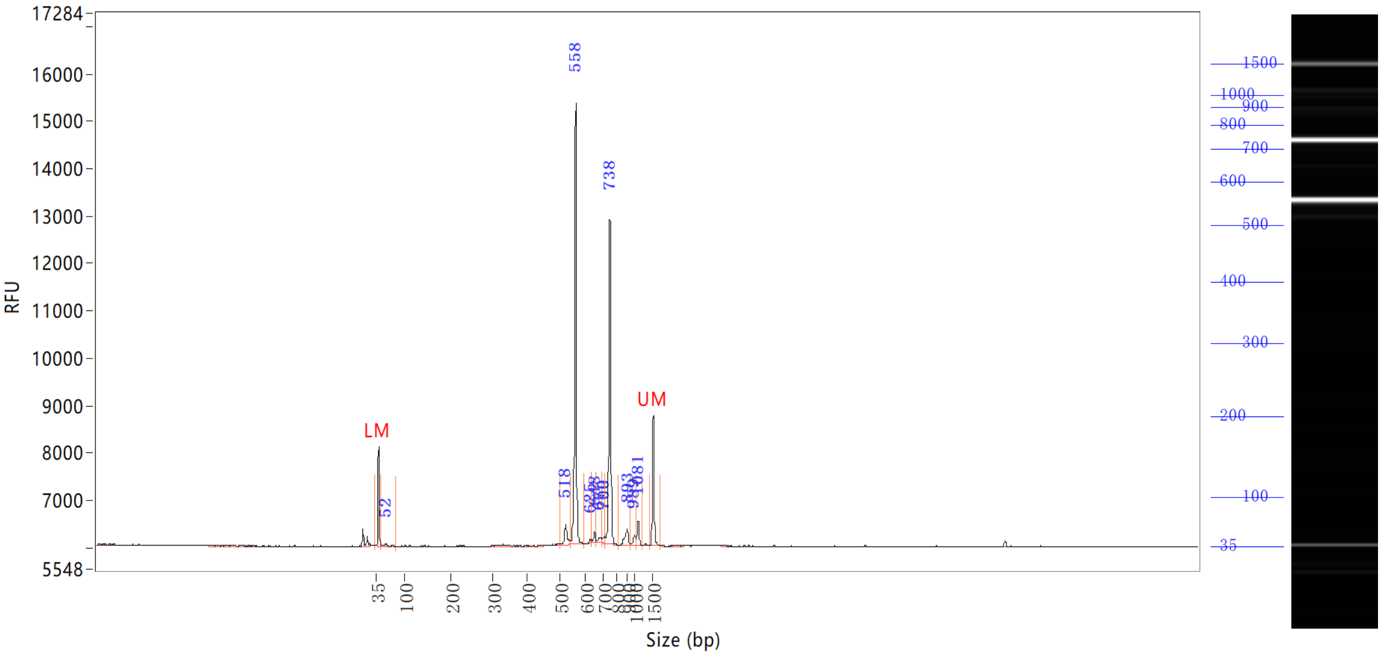

Peak	Size (bp)	Conc. (ng/uL)	From (bp)	To (bp)	Avg. Size (bp)	CV%	RFU	Corr. Peak Area
1	35 (LM)	0.4738	30	42	35	4.32	2092	9.839
2	52	0.3433	42	79	52	14.13	54	0.594
3	518	1.7030	499	538	520	1.46	425	2.947
4	558	23.2584	538	598	557	0.88	9336	40.249
5	625	0.2466	598	635	623	1.02	85	0.427
6	648	0.5281	635	658	647	0.72	209	0.914
7	675	0.4575	658	686	674	1.08	112	0.792
8	700	0.5328	686	708	697	0.89	143	0.922
9	738	16.7893	708	813	736	1.12	6865	29.054
10	893	1.6625	813	929	878	2.34	351	2.877
11	989	0.7343	929	1028	985	2.32	225	1.271
12	1081	1.3319	1028	1207	1077	2.59	517	2.305
13	1500 (UM)	0.5000	1414	1704	1497	1.99	2745	10.383

TIC:	47.5877	ng/uL
TIM:	133.2105	nmole/L
Total Conc.:	49.7234	ng/uL

```
Sample Peak Width (sec): 5    Sample Min Peak Height: 50    Sample Baseline V to V?: Y    Sample Baseline V to V pts: 3
Sample Filter: Binomial       # of Pts for Filter: 3        Sample Start Region (min): 0  Sample End Region (min): 75
Marker Peak Width (sec): 5    Marker Min Peak Height: 500   Marker Baseline V to V?: Y    Marker Baseline V to V pts: 3
Lower Marker Selection: First Peak > 500 RFU                Upper Marker Selection: Last Peak > 500 RFU
Ladder Size (bp)35, 100, 200, 300, 400, 500, 600, 700, 800, 900, 1000, 1500
Quantification Using: Upper Marker          Final Concentration (ng/uL): 0.5000          Dilution Factor: 12.0
```

 Copyright 2015 Advanced Analytical Technologies, Inc.

Sample: SampG5
Well Location: G5

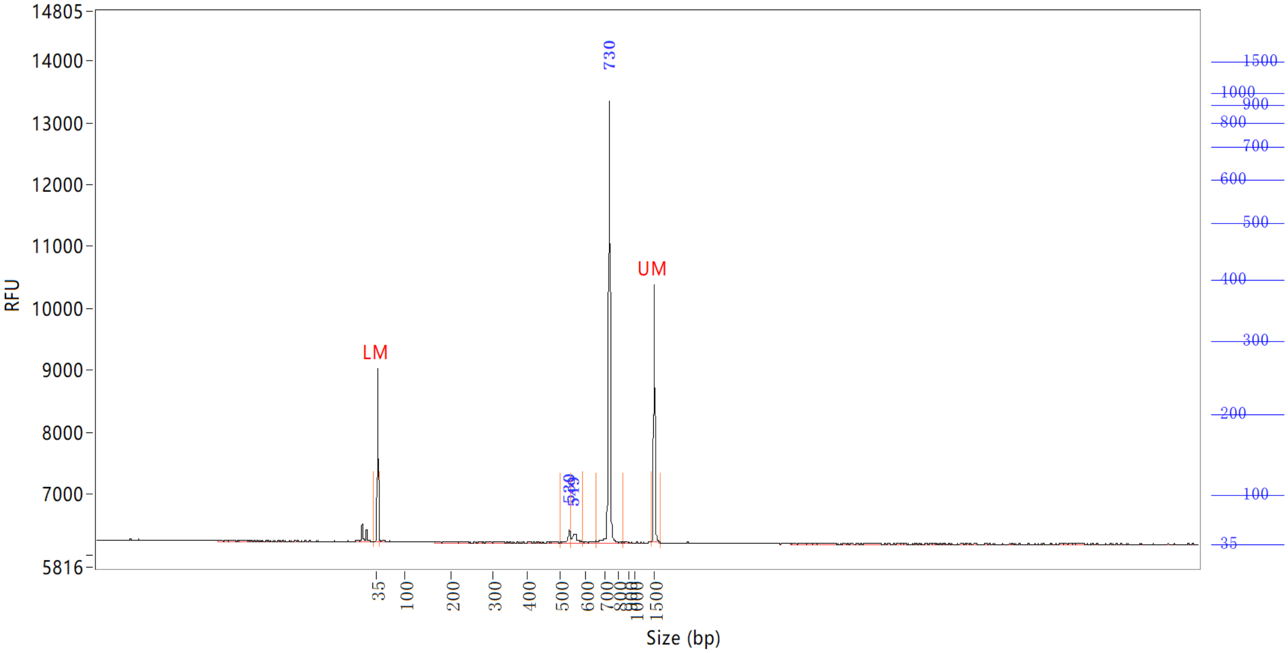

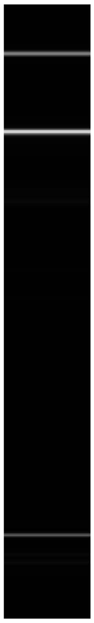

Peak	Size (bp)	Conc. (ng/uL)	From (bp)	To (bp)	Avg. Size (bp)	CV%	RFU	Corr. Peak Area
1	35 (LM)	0.4769	28	43	34	4.24	2810	13.066
2	530	0.5633	499	540	528	1.50	199	1.286
3	549	0.7154	540	589	555	1.84	148	1.633
4	730	12.8864	654	853	727	1.76	7158	29.423
5	1500 (UM)	0.5000	1420	1684	1497	1.55	4152	13.699

TIC:	14.1651	ng/uL
TIM:	33.0488	nmole/L
Total Conc.:	16.1677	ng/uL

Sample Peak Width (sec): 5   Sample Min Peak Height: 50   Sample Baseline V to V?: Y   Sample Baseline V to V pts: 3
Sample Filter: Binomial   # of Pts for Filter: 3   Sample Start Region (min): 0   Sample End Region (min): 75
Marker Peak Width (sec): 5   Marker Min Peak Height: 500   Marker Baseline V to V?: Y   Marker Baseline V to V pts: 3
Lower Marker Selection: First Peak > 500 RFU   Upper Marker Selection: Last Peak > 500 RFU
Ladder Size (bp)35, 100, 200, 300, 400, 500, 600, 700, 800, 900, 1000, 1500
Quantification Using: Upper Marker   Final Concentration (ng/uL): 0.5000   Dilution Factor: 12.0

 Copyright 2015 Advanced Analytical Technologies, Inc.

Sample: SampH5
Well Location: H5

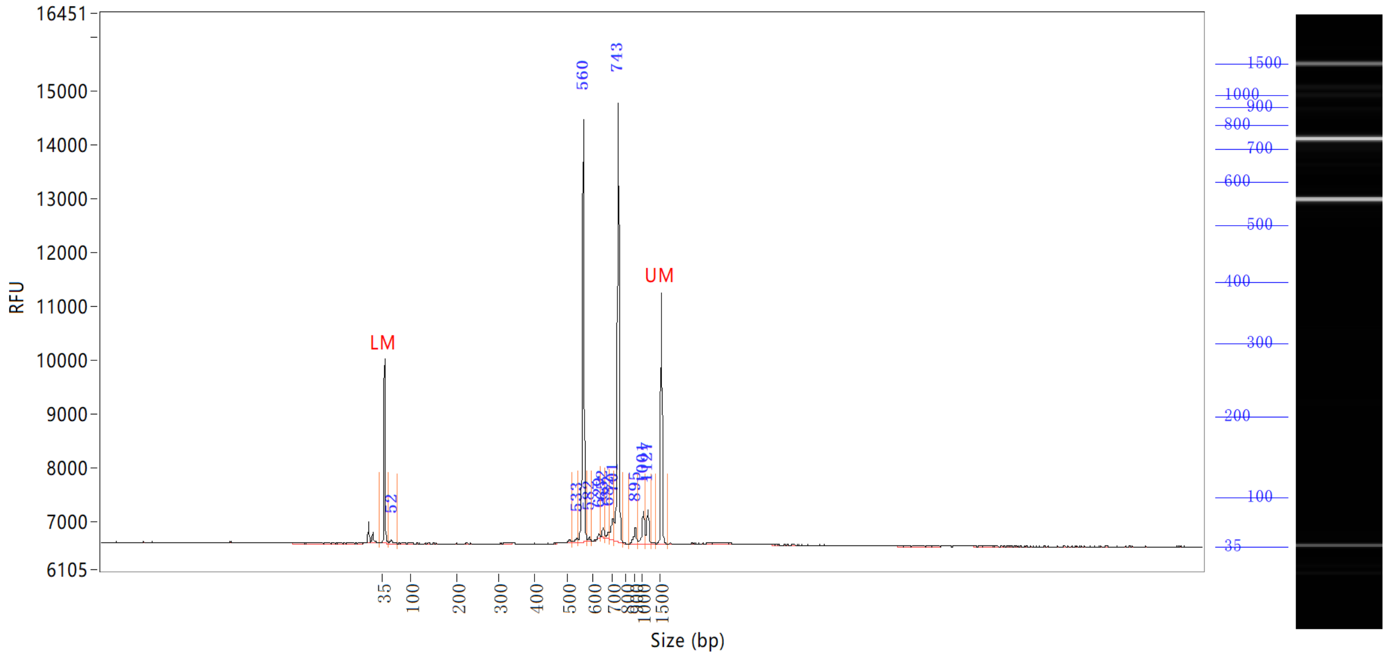

Peak	Size (bp)	Conc. (ng/uL)	From (bp)	To (bp)	Avg. Size (bp)	CV%	RFU	Corr. Peak Area
1	35 (LM)	0.5177	24	47	35	5.68	3433	16.303
2	52	0.2060	47	66	53	8.32	58	0.541
3	533	0.1891	515	539	529	1.25	60	0.496
4	560	12.3069	539	576	558	0.75	7850	32.293
5	582	0.1593	576	596	583	0.68	85	0.418
6	629	0.1368	596	639	627	1.07	77	0.359
7	652	0.3480	639	667	652	0.94	186	0.913
8	682	0.2991	667	687	679	0.82	133	0.785
9	701	1.0969	687	716	702	1.11	398	2.878
10	743	13.2820	716	782	741	1.07	8174	34.852
11	895	0.6743	827	926	885	2.00	298	1.769
12	1001	1.3323	926	1068	1001	2.81	614	3.496
13	1127	1.1283	1068	1241	1122	2.47	645	2.961
14	1500 (UM)	0.5000	1347	1691	1496	1.60	4683	15.744

TIC:	31.1588	ng/uL
TIM:	82.8338	nmole/L
Total Conc.:	32.6928	ng/uL

Sample Peak Width (sec): 5 Sample Min Peak Height: 50 Sample Baseline V to V?: Y Sample Baseline V to V pts: 3
Sample Filter: Binomial # of Pts for Filter: 3 Sample Start Region (min): 0 Sample End Region (min): 75
Marker Peak Width (sec): 5 Marker Min Peak Height: 500 Marker Baseline V to V?: Y Marker Baseline V to V pts: 3
Lower Marker Selection: First Peak > 500 RFU Upper Marker Selection: Last Peak > 500 RFU
Ladder Size (bp)35, 100, 200, 300, 400, 500, 600, 700, 800, 900, 1000, 1500
Quantification Using: Upper Marker Final Concentration (ng/uL): 0.5000 Dilution Factor: 12.0

 Copyright 2015 Advanced Analytical Technologies, Inc.

Sample: SampA6
Well Location: A6

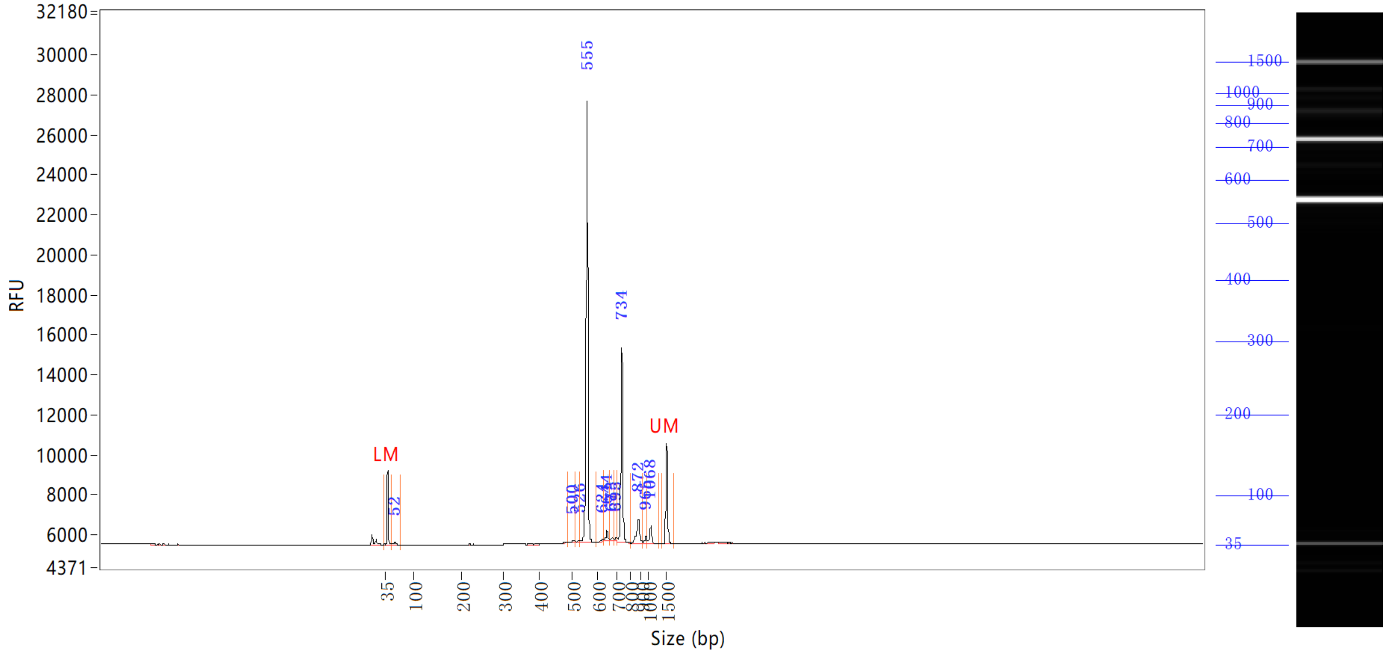

Peak	Size (bp)	Conc. (ng/uL)	From (bp)	To (bp)	Avg. Size (bp)	CV%	RFU	Corr. Peak Area
1	35 (LM)	0.4788	29	44	35	4.67	3733	17.123
2	52	0.3797	44	66	51	7.68	112	1.132
3	500	0.3316	487	512	500	1.21	111	0.988
4	526	0.2542	512	527	521	0.89	126	0.758
5	555	31.3525	527	595	553	0.85	22106	93.436
6	624	0.2648	595	630	618	1.36	126	0.789
7	644	0.8739	630	663	645	1.07	502	2.604
8	673	0.2783	663	684	673	0.85	130	0.829
9	695	0.3845	684	705	694	0.83	179	1.146
10	734	13.1458	705	800	731	1.11	9702	39.177
11	872	2.7333	800	919	866	2.19	1210	8.146
12	965	0.6889	919	996	960	2.00	371	2.053
13	1068	1.3000	996	1287	1062	3.63	881	3.874
14	1500 (UM)	0.5000	1400	1711	1494	1.75	4981	17.881

TIC:	51.9877	ng/uL
TIM:	149.9014	nmole/L
Total Conc.:	52.9404	ng/uL

```
Sample Peak Width (sec): 5    Sample Min Peak Height: 50    Sample Baseline V to V?: Y    Sample Baseline V to V pts: 3
Sample Filter: Binomial       # of Pts for Filter: 3        Sample Start Region (min): 0  Sample End Region (min): 75
Marker Peak Width (sec): 5    Marker Min Peak Height: 500   Marker Baseline V to V?: Y    Marker Baseline V to V pts: 3
Lower Marker Selection: First Peak > 500 RFU                Upper Marker Selection: Last Peak > 500 RFU
Ladder Size (bp)35, 100, 200, 300, 400, 500, 600, 700, 800, 900, 1000, 1500
Quantification Using: Upper Marker          Final Concentration (ng/uL): 0.5000          Dilution Factor: 12.0
```

 Copyright 2015 Advanced Analytical Technologies, Inc.

Sample: SampB6
Well Location: B6

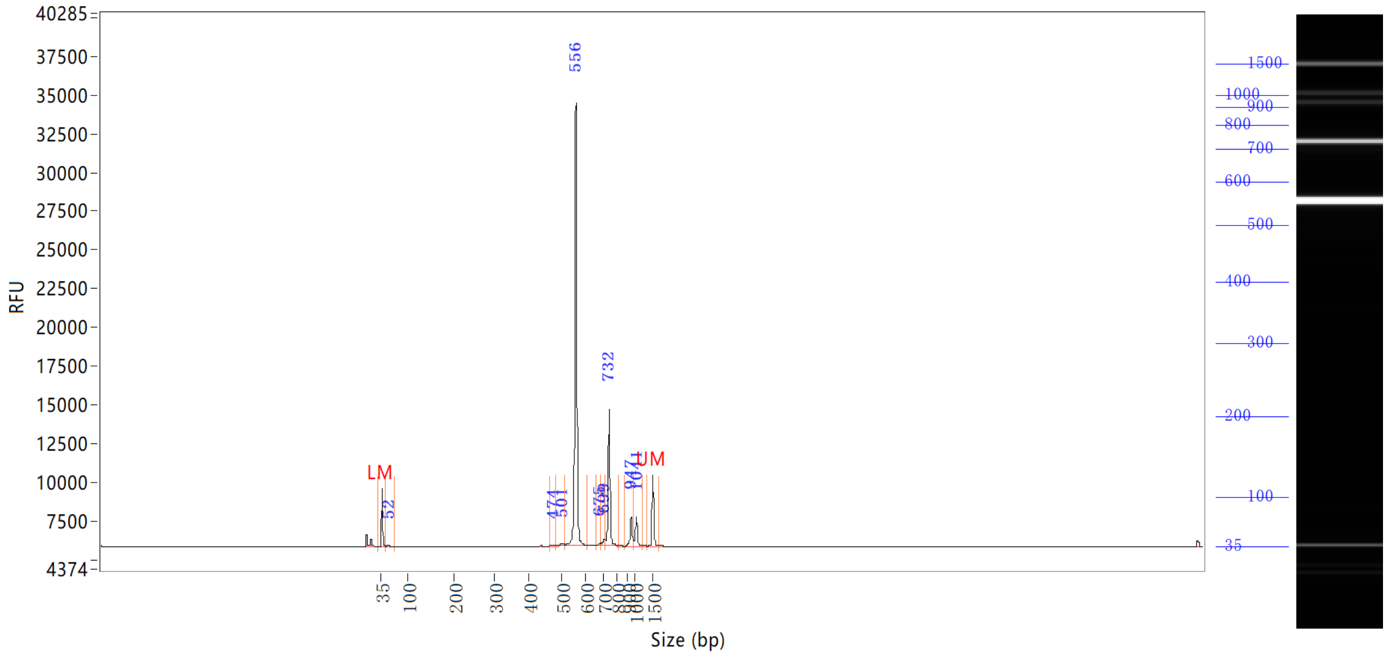

Peak	Size (bp)	Conc. (ng/uL)	From (bp)	To (bp)	Avg. Size (bp)	CV%	RFU	Corr. Peak Area
1	35 (LM)	0.5071	28	45	35	5.08	3758	17.796
2	52	0.3146	45	67	51	8.60	82	0.920
3	474	0.2250	462	479	472	0.99	79	0.658
4	501	0.6528	479	511	495	1.68	143	1.909
5	556	47.9679	511	605	555	1.19	28647	140.288
6	675	0.3300	653	680	671	0.89	176	0.965
7	699	0.9674	680	706	695	1.01	447	2.829
8	732	12.7660	706	808	731	1.48	8859	37.336
9	947	3.3884	865	989	941	2.32	1914	9.910
10	1041	2.8778	989	1227	1036	3.00	1884	8.417
11	1500 (UM)	0.5000	1340	1678	1497	1.98	4568	17.548

TIC:	69.4899	ng/uL
TIM:	197.6253	nmole/L
Total Conc.:	70.9318	ng/uL

Sample Peak Width (sec): 5 Sample Min Peak Height: 50 Sample Baseline V to V?: Y Sample Baseline V to V pts: 3
Sample Filter: Binomial # of Pts for Filter: 3 Sample Start Region (min): 0 Sample End Region (min): 75
Marker Peak Width (sec): 5 Marker Min Peak Height: 500 Marker Baseline V to V?: Y Marker Baseline V to V pts: 3
Lower Marker Selection: First Peak > 500 RFU Upper Marker Selection: Last Peak > 500 RFU
Ladder Size (bp)35, 100, 200, 300, 400, 500, 600, 700, 800, 900, 1000, 1500
Quantification Using: Upper Marker Final Concentration (ng/uL): 0.5000 Dilution Factor: 12.0

 Copyright 2015 Advanced Analytical Technologies, Inc.

Sample: SampC6
Well Location: C6

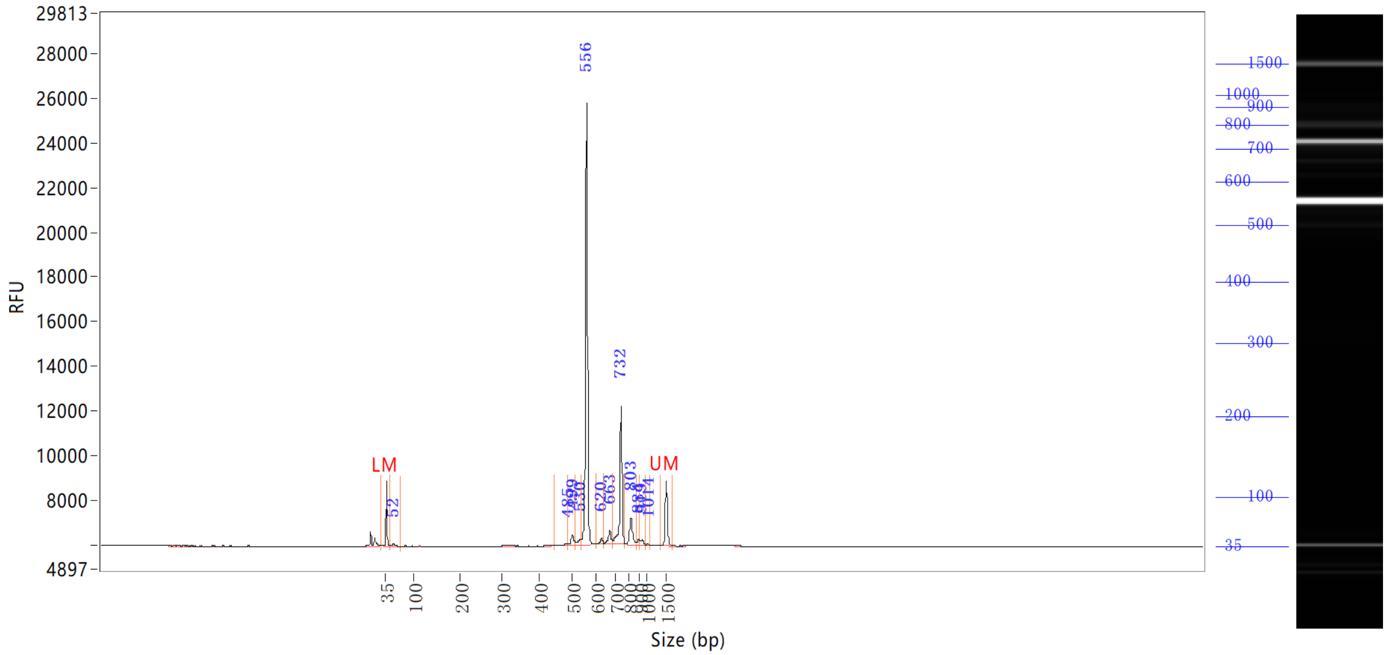

Peak	Size (bp)	Conc. (ng/uL)	From (bp)	To (bp)	Avg. Size (bp)	CV%	RFU	Corr. Peak Area
1	35 (LM)	0.5338	24	44	35	5.81	2908	14.024
2	52	0.5472	44	69	52	9.27	110	1.198
3	485	0.5433	446	489	477	1.99	91	1.189
4	499	1.5014	489	512	500	1.01	474	3.287
5	530	1.2181	512	536	526	1.30	283	2.667
6	556	42.5658	536	594	555	0.83	19831	93.191
7	620	0.6898	594	634	618	1.11	278	1.510
8	663	1.8298	634	680	661	1.47	607	4.006
9	732	14.5452	680	765	726	1.75	6165	31.844
10	803	3.9055	765	860	808	2.03	1207	8.550
11	884	0.7423	860	900	881	1.28	260	1.625
12	919	0.6280	900	975	922	1.80	241	1.375
13	1014	0.1380	975	1081	1008	1.90	65	0.302
14	1500 (UM)	0.5000	1360	1684	1495	2.14	2913	13.136

TIC:	68.8545	ng/uL
TIM:	204.1267	nmole/L
Total Conc.:	70.6793	ng/uL

Sample Peak Width (sec): 5   Sample Min Peak Height: 50   Sample Baseline V to V?: Y   Sample Baseline V to V pts: 3
Sample Filter: Binomial   # of Pts for Filter: 3   Sample Start Region (min): 0   Sample End Region (min): 75
Marker Peak Width (sec): 5   Marker Min Peak Height: 500   Marker Baseline V to V?: Y   Marker Baseline V to V pts: 3
Lower Marker Selection: First Peak > 500 RFU   Upper Marker Selection: Last Peak > 500 RFU
Ladder Size (bp)35, 100, 200, 300, 400, 500, 600, 700, 800, 900, 1000, 1500
Quantification Using: Upper Marker   Final Concentration (ng/uL): 0.5000   Dilution Factor: 12.0

 Copyright 2015 Advanced Analytical Technologies, Inc.

Sample: SampD6
Well Location: D6

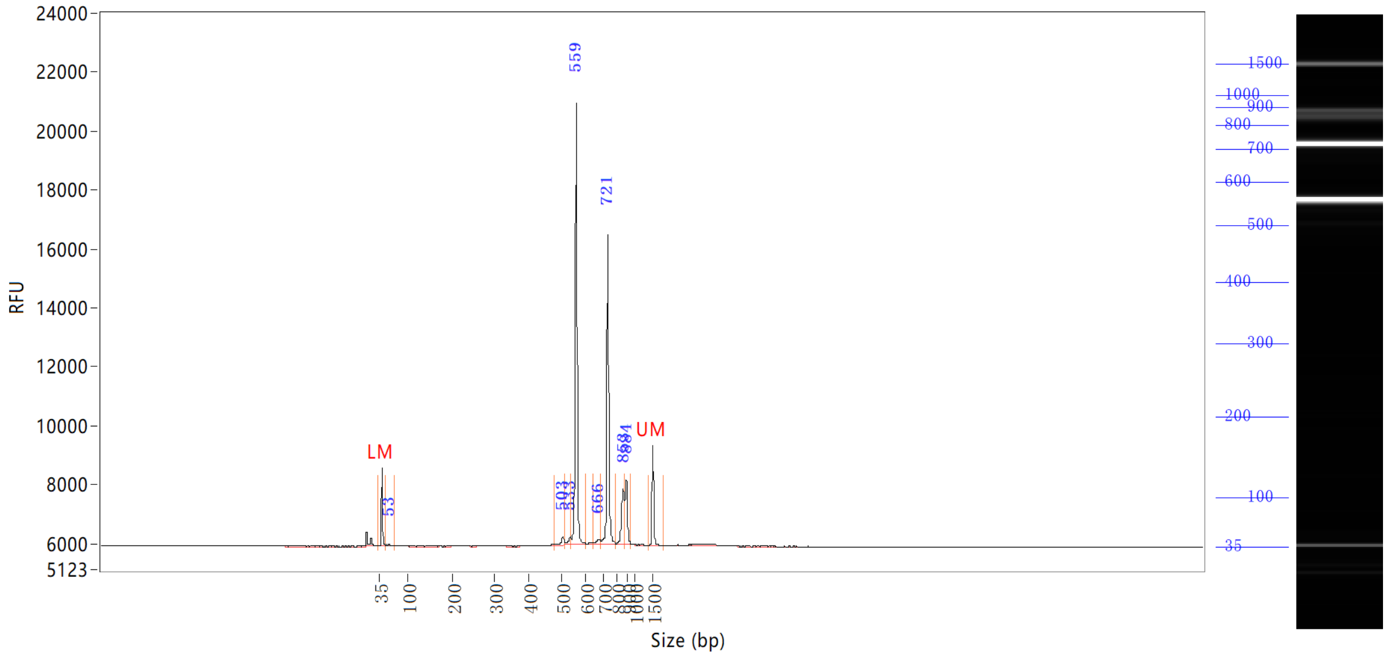

Peak	Size (bp)	Conc. (ng/uL)	From (bp)	To (bp)	Avg. Size (bp)	CV%	RFU	Corr. Peak Area
1	35 (LM)	0.5100	27	45	35	5.99	2666	13.157
2	53	0.4766	45	67	51	8.73	91	1.024
3	503	1.0993	479	514	499	1.59	295	2.363
4	533	0.8752	514	538	529	1.24	249	1.881
5	559	31.5805	538	598	557	0.93	14997	67.889
6	666	0.5282	642	677	664	1.34	132	1.136
7	721	21.8617	677	791	720	1.52	10514	46.996
8	853	6.0129	791	869	847	1.72	1867	12.926
9	884	4.9795	869	954	884	1.33	2170	10.704
10	1500 (UM)	0.5000	1394	1803	1499	2.36	3390	12.898

TIC:	67.4139	ng/uL
TIM:	187.1904	nmole/L
Total Conc.:	69.0553	ng/uL

Sample Peak Width (sec): 5 Sample Min Peak Height: 50 Sample Baseline V to V?: Y Sample Baseline V to V pts: 3
Sample Filter: Binomial # of Pts for Filter: 3 Sample Start Region (min): 0 Sample End Region (min): 75
Marker Peak Width (sec): 5 Marker Min Peak Height: 500 Marker Baseline V to V?: Y Marker Baseline V to V pts: 3
Lower Marker Selection: First Peak > 500 RFU Upper Marker Selection: Last Peak > 500 RFU
Ladder Size (bp)35, 100, 200, 300, 400, 500, 600, 700, 800, 900, 1000, 1500
Quantification Using: Upper Marker Final Concentration (ng/uL): 0.5000 Dilution Factor: 12.0

 Copyright 2015 Advanced Analytical Technologies, Inc.

Sample: SampE6
Well Location: E6

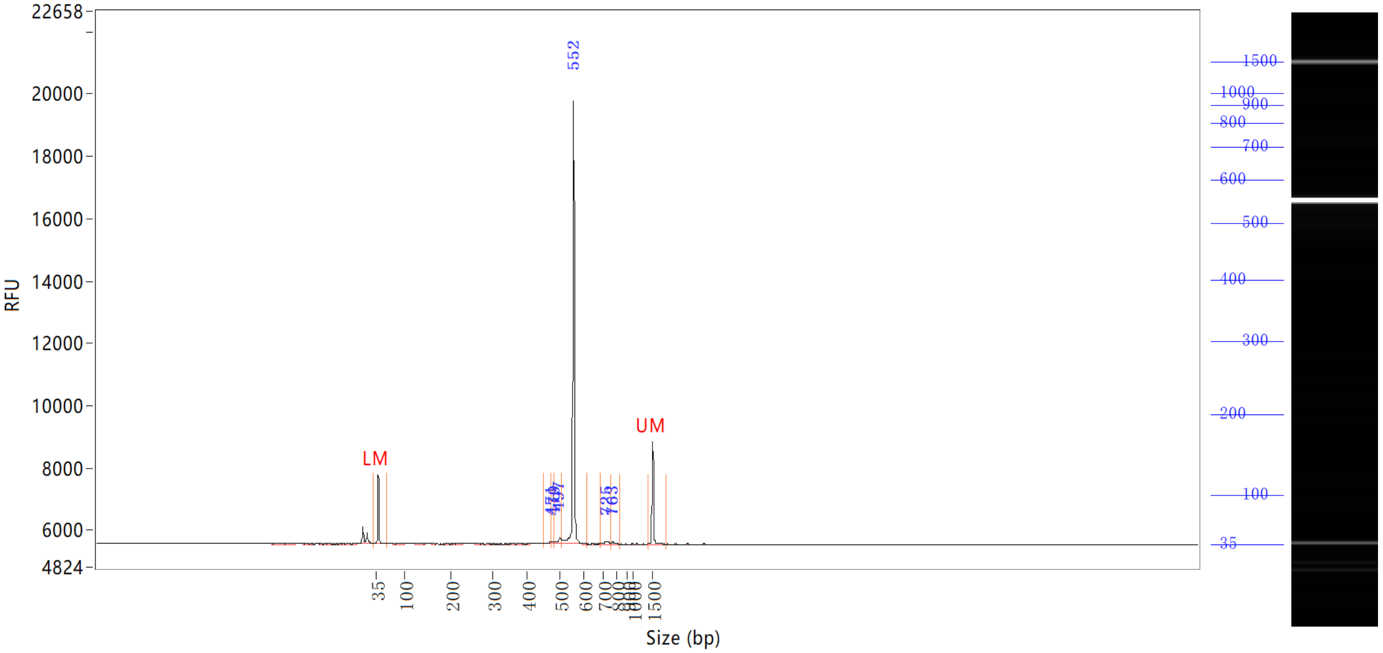

Peak	Size (bp)	Conc. (ng/uL)	From (bp)	To (bp)	Avg. Size (bp)	CV%	RFU	Corr. Peak Area
1	35 (LM)	0.4974	25	59	35	6.89	2246	10.979
2	471	0.2459	447	474	466	1.37	61	0.452
3	479	0.2346	474	482	478	0.56	68	0.432
4	497	1.0487	482	508	496	1.32	186	1.929
5	552	31.0144	508	614	550	1.31	14234	57.052
6	725	0.4721	685	749	719	2.10	81	0.868
7	763	0.2444	749	822	770	2.12	60	0.450
8	1500 (UM)	0.5000	1407	1869	1499	2.58	3296	11.037

TIC:	33.2601	ng/uL
TIM:	99.5521	nmole/L
Total Conc.:	35.1351	ng/uL

```
Sample Peak Width (sec): 5    Sample Min Peak Height: 50    Sample Baseline V to V?: Y    Sample Baseline V to V pts: 3
Sample Filter: Binomial       # of Pts for Filter: 3        Sample Start Region (min): 0  Sample End Region (min): 75
Marker Peak Width (sec): 5    Marker Min Peak Height: 500   Marker Baseline V to V?: Y    Marker Baseline V to V pts: 3
Lower Marker Selection: First Peak > 500 RFU                Upper Marker Selection: Last Peak > 500 RFU
Ladder Size (bp)35, 100, 200, 300, 400, 500, 600, 700, 800, 900, 1000, 1500
Quantification Using: Upper Marker          Final Concentration (ng/uL): 0.5000          Dilution Factor: 12.0
```

 Copyright 2015 Advanced Analytical Technologies, Inc.

Sample: SampF6
Well Location: F6

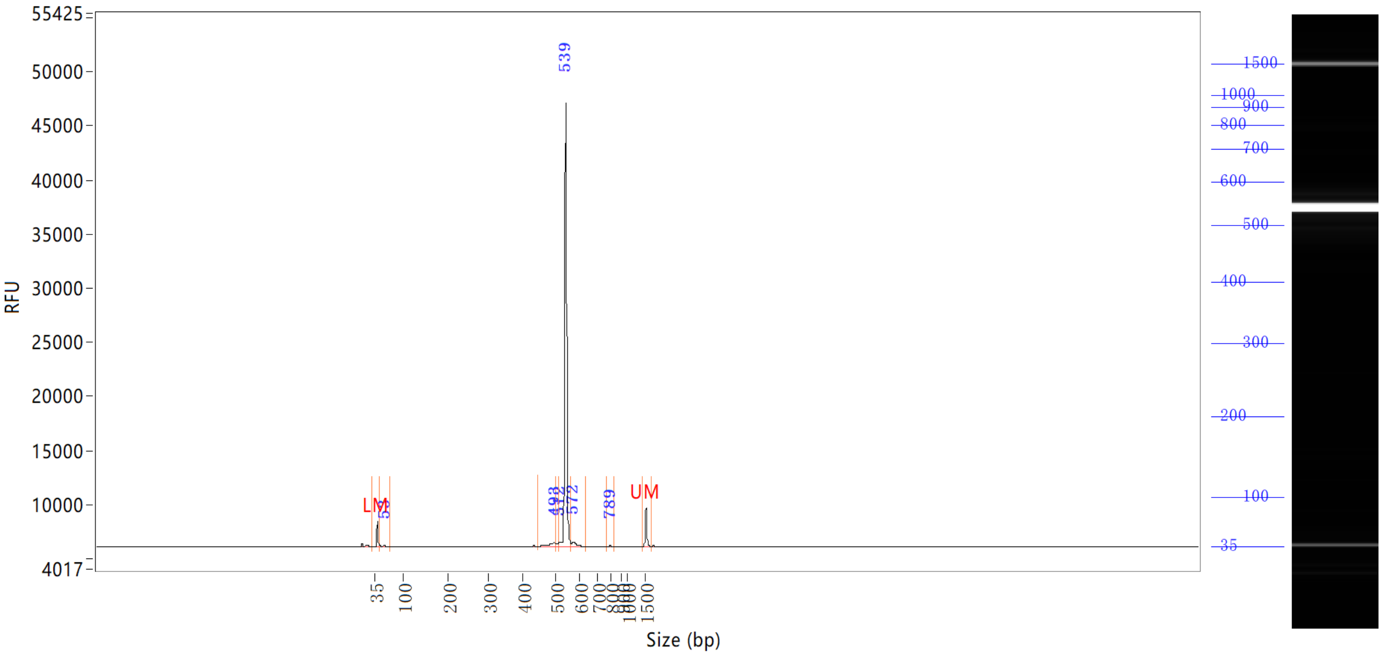

Peak	Size (bp)	Conc. (ng/uL)	From (bp)	To (bp)	Avg. Size (bp)	CV%	RFU	Corr. Peak Area
1	35 (LM)	0.4802	24	44	35	6.29	2384	11.854
2	53	0.5472	44	68	52	8.54	96	1.126
3	493	2.6778	444	501	483	2.61	342	5.509
4	512	0.8306	501	513	508	0.71	319	1.709
5	539	98.1480	513	564	539	0.94	41073	201.924
6	572	2.0522	564	631	578	2.15	434	4.222
7	789	0.2298	763	829	791	1.59	72	0.473
8	1500 (UM)	0.5000	1394	1645	1497	1.80	3514	12.344

TIC:	104.4856	ng/uL
TIM:	335.0198	nmole/L
Total Conc.:	106.0477	ng/uL

Sample Peak Width (sec): 5   Sample Min Peak Height: 50   Sample Baseline V to V?: Y   Sample Baseline V to V pts: 3
Sample Filter: Binomial   # of Pts for Filter: 3   Sample Start Region (min): 0   Sample End Region (min): 75
Marker Peak Width (sec): 5   Marker Min Peak Height: 500   Marker Baseline V to V?: Y   Marker Baseline V to V pts: 3
Lower Marker Selection: First Peak > 500 RFU   Upper Marker Selection: Last Peak > 500 RFU
Ladder Size (bp)35, 100, 200, 300, 400, 500, 600, 700, 800, 900, 1000, 1500
Quantification Using: Upper Marker   Final Concentration (ng/uL): 0.5000   Dilution Factor: 12.0

Copyright 2015 Advanced Analytical Technologies, Inc.

Sample: SampG6
Well Location: G6

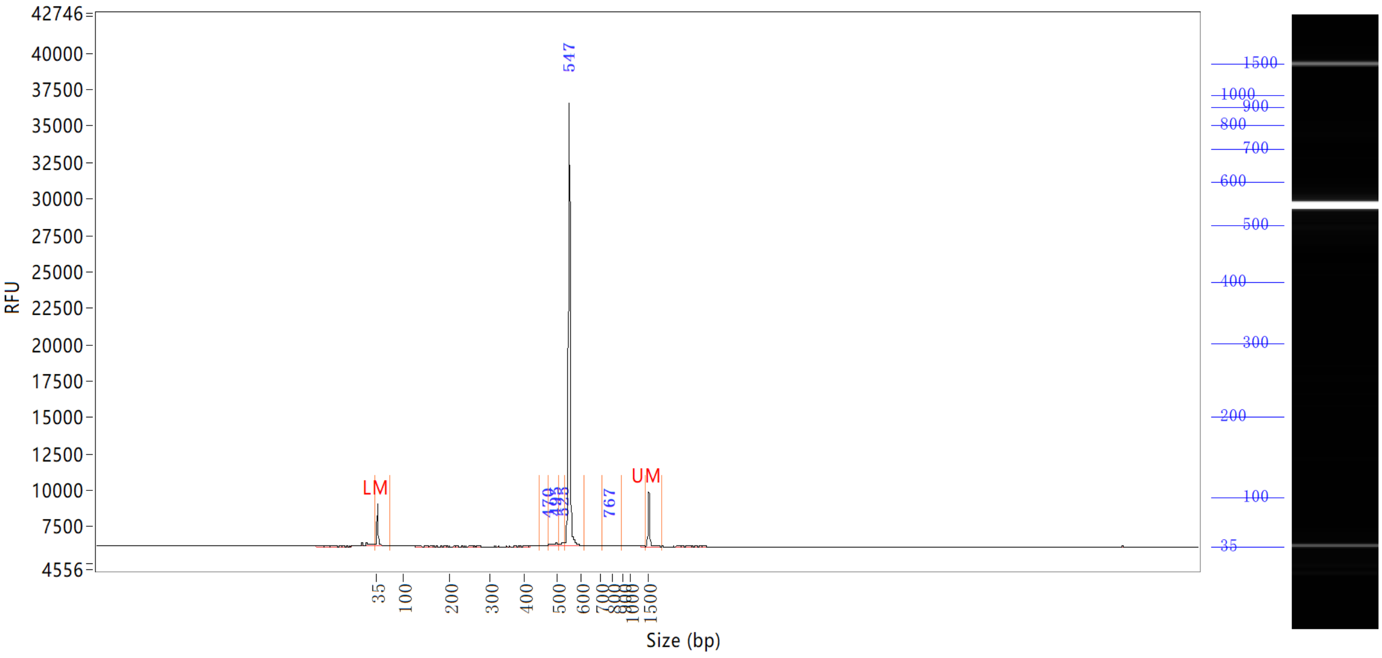

Peak	Size (bp)	Conc. (ng/uL)	From (bp)	To (bp)	Avg. Size (bp)	CV%	RFU	Corr. Peak Area
1	35 (LM)	0.6269	29	67	36	13.32	2860	16.129
2	470	0.4505	444	474	465	1.45	108	0.966
3	495	1.4342	474	504	490	1.65	247	3.075
4	525	1.0782	504	527	517	1.29	223	2.311
5	547	66.8591	527	615	546	1.16	30464	143.334
6	767	0.4769	718	886	778	4.25	81	1.022
7	1500 (UM)	0.5000	1414	1862	1503	2.69	3706	12.863

TIC:	70.2989	ng/uL
TIM:	212.3564	nmole/L
Total Conc.:	72.0048	ng/uL

Sample Peak Width (sec): 5 Sample Min Peak Height: 50 Sample Baseline V to V?: Y Sample Baseline V to V pts: 3
Sample Filter: Binomial # of Pts for Filter: 3 Sample Start Region (min): 0 Sample End Region (min): 75
Marker Peak Width (sec): 5 Marker Min Peak Height: 500 Marker Baseline V to V?: Y Marker Baseline V to V pts: 3
Lower Marker Selection: First Peak > 500 RFU Upper Marker Selection: Last Peak > 500 RFU
Ladder Size (bp)35, 100, 200, 300, 400, 500, 600, 700, 800, 900, 1000, 1500
Quantification Using: Upper Marker Final Concentration (ng/uL): 0.5000 Dilution Factor: 12.0

 Copyright 2015 Advanced Analytical Technologies, Inc.

Sample: SampH6
Well Location: H6

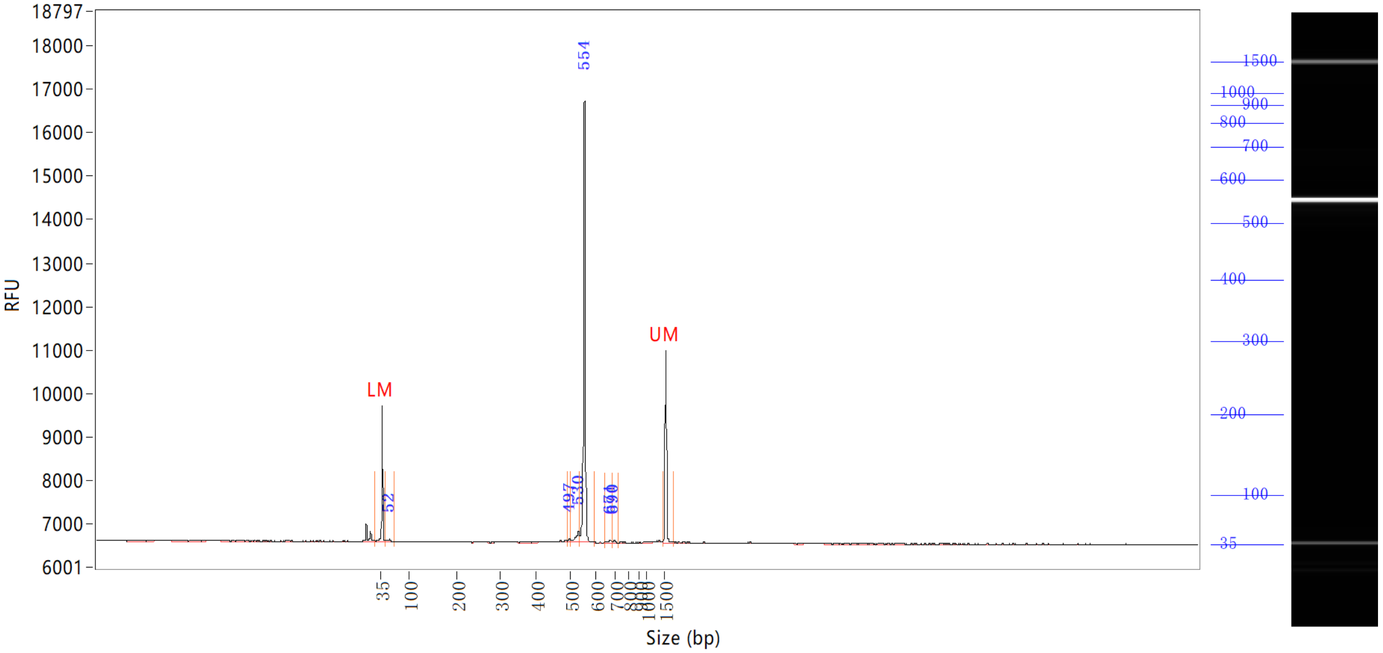

Peak	Size (bp)	Conc. (ng/uL)	From (bp)	To (bp)	Avg. Size (bp)	CV%	RFU	Corr. Peak Area
1	35 (LM)	0.5170	19	43	34	6.45	3132	14.944
2	52	0.3309	43	64	50	8.44	56	0.797
3	497	0.1493	490	503	496	0.69	57	0.360
4	530	0.8953	503	536	524	1.58	247	2.157
5	554	17.3617	536	595	552	0.77	10173	41.821
6	671	0.2066	648	684	668	1.44	53	0.498
7	690	0.1261	684	725	693	1.11	61	0.304
8	1500 (UM)	0.5000	1427	1744	1497	1.79	4434	14.453

TIC:	19.0699	ng/uL
TIM:	66.7125	nmole/L
Total Conc.:	20.7841	ng/uL

Sample Peak Width (sec): 5   Sample Min Peak Height: 50   Sample Baseline V to V?: Y   Sample Baseline V to V pts: 3
Sample Filter: Binomial   # of Pts for Filter: 3   Sample Start Region (min): 0   Sample End Region (min): 75
Marker Peak Width (sec): 5   Marker Min Peak Height: 500   Marker Baseline V to V?: Y   Marker Baseline V to V pts: 3
Lower Marker Selection: First Peak > 500 RFU   Upper Marker Selection: Last Peak > 500 RFU
Ladder Size (bp)35, 100, 200, 300, 400, 500, 600, 700, 800, 900, 1000, 1500
Quantification Using: Upper Marker   Final Concentration (ng/uL): 0.5000   Dilution Factor: 12.0

 Copyright 2015 Advanced Analytical Technologies, Inc.

Sample: SampA7
Well Location: A7

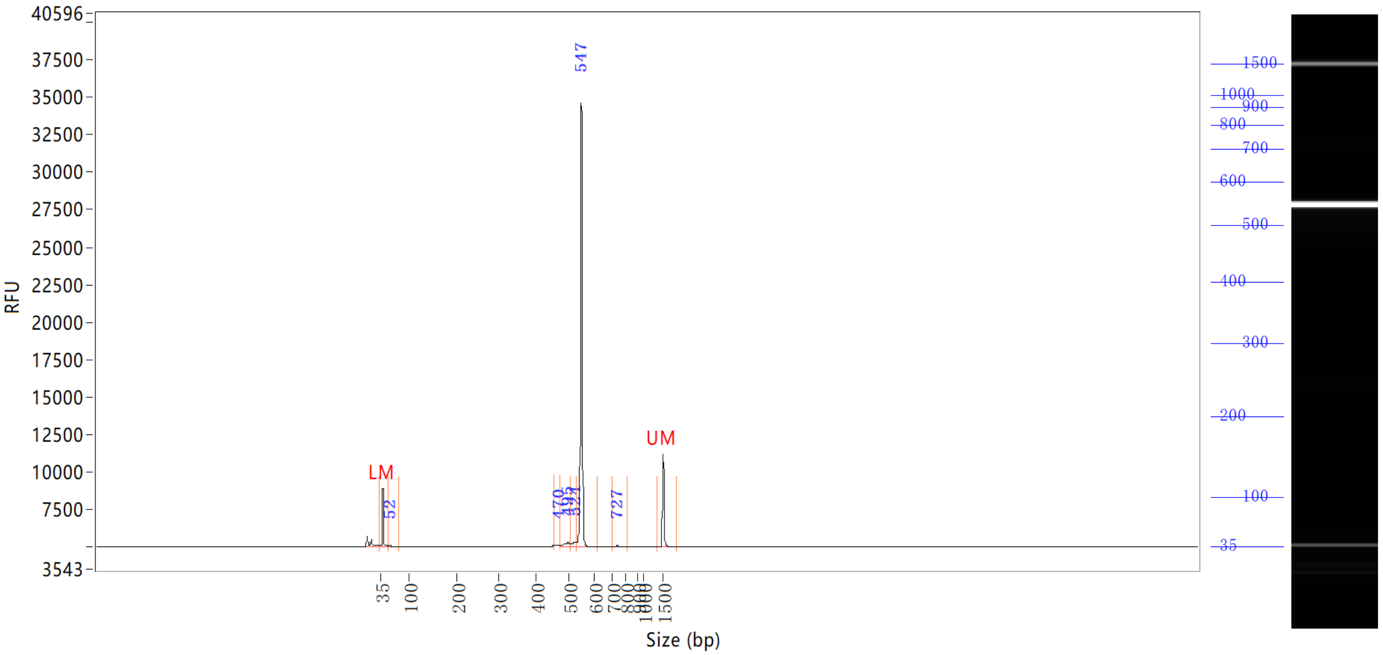

Peak	Size (bp)	Conc. (ng/uL)	From (bp)	To (bp)	Avg. Size (bp)	CV%	RFU	Corr. Peak Area
1	35 (LM)	0.4704	30	48	35	6.51	3926	19.205
2	52	0.2290	48	74	53	10.55	77	0.779
3	470	0.1376	456	472	467	0.72	70	0.468
4	495	0.8974	472	504	490	1.70	246	3.053
5	524	0.8413	504	528	517	1.35	279	2.862
6	547	36.0784	528	619	546	0.78	29605	122.750
7	727	0.1872	699	808	735	3.02	60	0.637
8	1500 (UM)	0.5000	1354	1875	1495	1.89	6194	20.414

TIC:	38.3709	ng/uL
TIM:	122.4096	nmole/L
Total Conc.:	39.3686	ng/uL

```
Sample Peak Width (sec): 5    Sample Min Peak Height: 50    Sample Baseline V to V?: Y    Sample Baseline V to V pts: 3
Sample Filter: Binomial       # of Pts for Filter: 3        Sample Start Region (min): 0  Sample End Region (min): 75
Marker Peak Width (sec): 5    Marker Min Peak Height: 500   Marker Baseline V to V?: Y    Marker Baseline V to V pts: 3
Lower Marker Selection: First Peak > 500 RFU                Upper Marker Selection: Last Peak > 500 RFU
Ladder Size (bp)35, 100, 200, 300, 400, 500, 600, 700, 800, 900, 1000, 1500
Quantification Using: Upper Marker          Final Concentration (ng/uL): 0.5000          Dilution Factor: 12.0
```

 Copyright 2015 Advanced Analytical Technologies, Inc.

Sample: SampB7
Well Location: B7

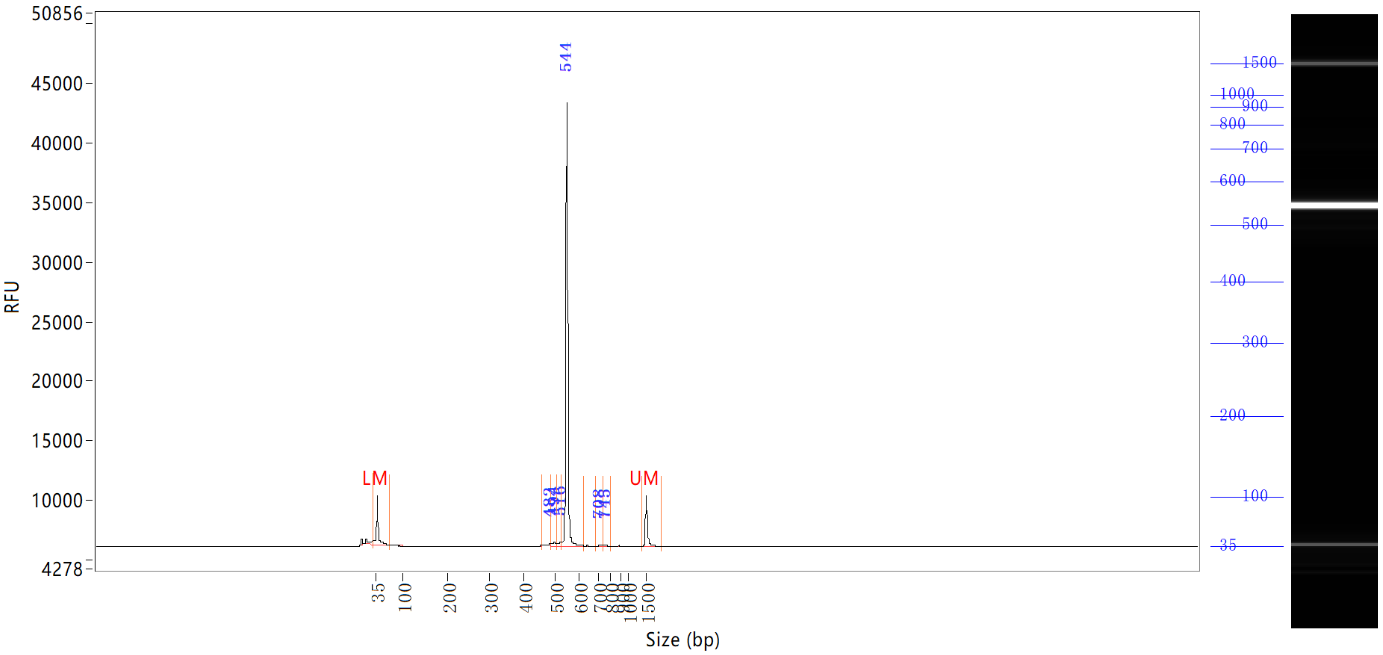

Peak	Size (bp)	Conc. (ng/uL)	From (bp)	To (bp)	Avg. Size (bp)	CV%	RFU	Corr. Peak Area
1	35 (LM)	0.8018	29	67	37	15.25	4036	27.564
2	482	0.6567	456	485	475	1.39	160	1.881
3	494	1.0771	485	506	495	1.12	325	3.086
4	516	1.0929	506	526	516	1.06	350	3.131
5	544	62.9874	526	625	544	1.16	37194	180.452
6	708	0.2960	687	732	709	1.58	105	0.848
7	745	0.2062	732	800	758	2.39	60	0.591
8	1500 (UM)	0.5000	1394	1934	1510	3.86	4164	17.189

TIC:	66.3163	ng/uL
TIM:	201.0096	nmole/L
Total Conc.:	67.5891	ng/uL

```
Sample Peak Width (sec): 5    Sample Min Peak Height: 50    Sample Baseline V to V?: Y    Sample Baseline V to V pts: 3
Sample Filter: Binomial       # of Pts for Filter: 3        Sample Start Region (min): 0  Sample End Region (min): 75
Marker Peak Width (sec): 5    Marker Min Peak Height: 500   Marker Baseline V to V?: Y    Marker Baseline V to V pts: 3
Lower Marker Selection: First Peak > 500 RFU                Upper Marker Selection: Last Peak > 500 RFU
Ladder Size (bp)35, 100, 200, 300, 400, 500, 600, 700, 800, 900, 1000, 1500
Quantification Using: Upper Marker          Final Concentration (ng/uL): 0.5000          Dilution Factor: 12.0
```

 Copyright 2015 Advanced Analytical Technologies, Inc.

Sample: SampC7
Well Location: C7

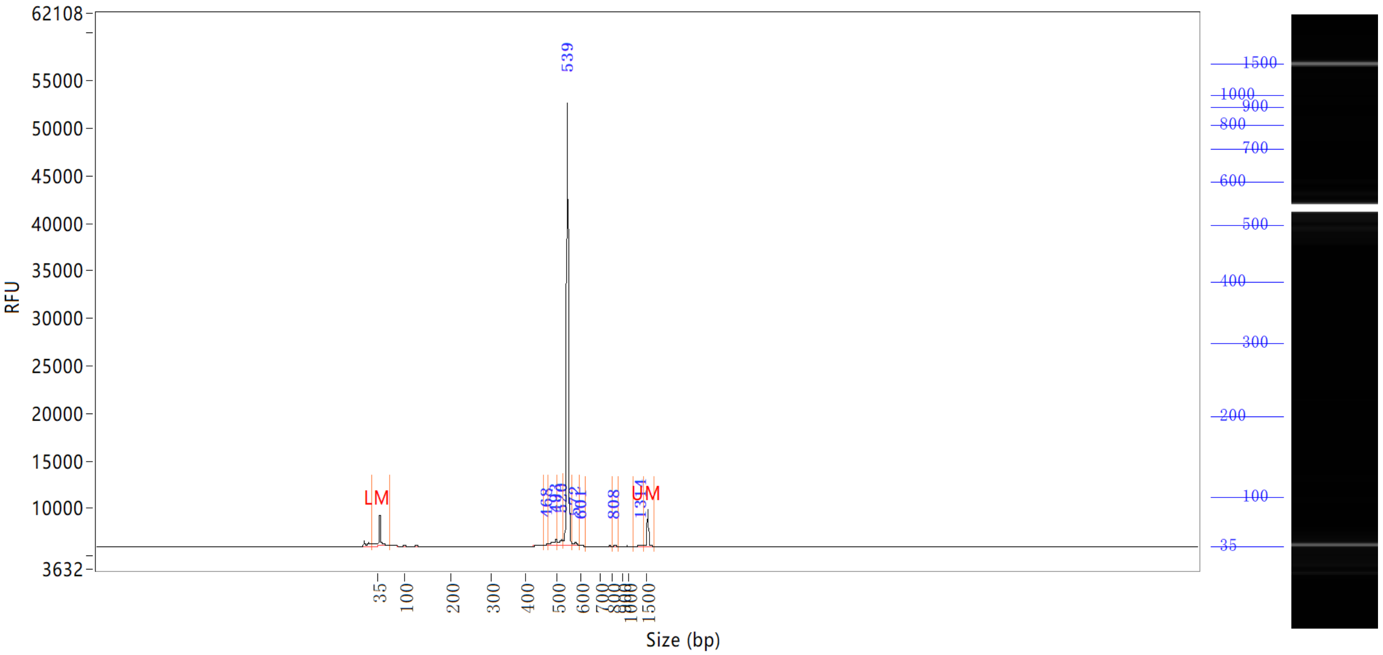

Peak	Size (bp)	Conc. (ng/uL)	From (bp)	To (bp)	Avg. Size (bp)	CV%	RFU	Corr. Peak Area
1	35 (LM)	1.0156	18	63	35	20.71	3269	27.081
2	468	0.2523	458	468	466	0.43	170	0.561
3	493	2.9653	468	501	486	1.80	553	6.589
4	520	2.3673	501	525	514	1.25	537	5.260
5	539	99.2831	525	563	538	0.76	46562	220.610
6	572	0.8866	563	590	572	0.93	302	1.970
7	601	0.2277	590	624	600	0.82	113	0.506
8	808	0.2142	791	860	816	1.91	55	0.476
9	1314	0.4000	1101	1400	1274	6.21	75	0.889
10	1500 (UM)	0.5000	1400	1711	1497	2.16	3921	13.332

TIC:	106.5966	ng/uL
TIM:	326.3085	nmole/L
Total Conc.:	108.4117	ng/uL

Sample Peak Width (sec): 5   Sample Min Peak Height: 50   Sample Baseline V to V?: Y   Sample Baseline V to V pts: 3
Sample Filter: Binomial   # of Pts for Filter: 3   Sample Start Region (min): 0   Sample End Region (min): 75
Marker Peak Width (sec): 5   Marker Min Peak Height: 500   Marker Baseline V to V?: Y   Marker Baseline V to V pts: 3
Lower Marker Selection: First Peak > 500 RFU   Upper Marker Selection: Last Peak > 500 RFU
Ladder Size (bp)35, 100, 200, 300, 400, 500, 600, 700, 800, 900, 1000, 1500
Quantification Using: Upper Marker   Final Concentration (ng/uL): 0.5000   Dilution Factor: 12.0

 Copyright 2015 Advanced Analytical Technologies, Inc.

Sample: SampD7
Well Location: D7

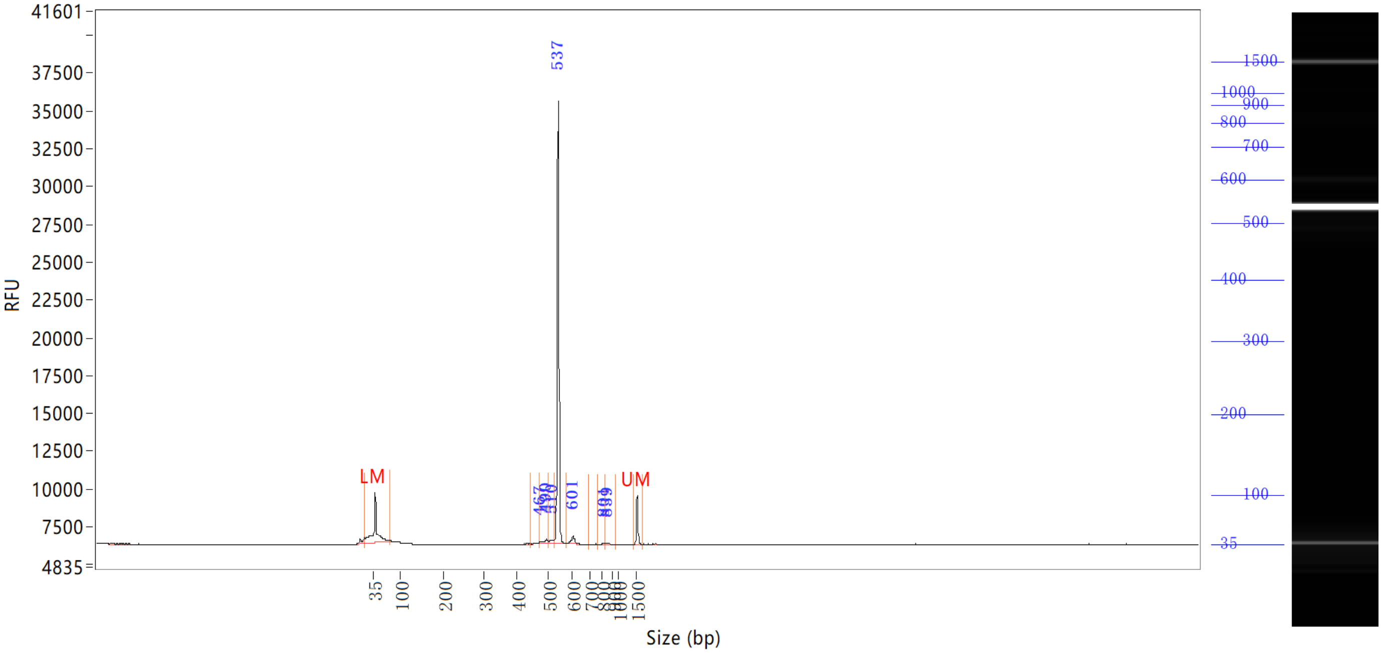

Peak	Size (bp)	Conc. (ng/uL)	From (bp)	To (bp)	Avg. Size (bp)	CV%	RFU	Corr. Peak Area
1	35 (LM)	1.9375	11	76	34	33.63	3277	42.817
2	467	0.4165	441	471	464	1.25	111	0.767
3	490	1.8450	471	499	486	1.52	311	3.398
4	510	1.3199	499	521	510	1.25	244	2.431
5	537	70.1422	521	575	536	0.77	29323	129.173
6	601	2.1391	575	692	602	2.21	505	3.939
7	801	0.3100	762	822	800	1.61	90	0.571
8	839	0.3451	822	936	848	2.38	82	0.636
9	1500 (UM)	0.5000	1407	1658	1498	1.69	3221	11.050

TIC:	76.5179	ng/uL
TIM:	234.4800	nmole/L
Total Conc.:	77.8076	ng/uL

```
Sample Peak Width (sec): 5     Sample Min Peak Height: 50    Sample Baseline V to V?: Y    Sample Baseline V to V pts: 3
Sample Filter: Binomial        # of Pts for Filter: 3        Sample Start Region (min): 0  Sample End Region (min): 75
Marker Peak Width (sec): 5     Marker Min Peak Height: 500   Marker Baseline V to V?: Y    Marker Baseline V to V pts: 3
Lower Marker Selection: First Peak > 500 RFU                 Upper Marker Selection: Last Peak > 500 RFU
Ladder Size (bp)35, 100, 200, 300, 400, 500, 600, 700, 800, 900, 1000, 1500
Quantification Using: Upper Marker          Final Concentration (ng/uL): 0.5000         Dilution Factor: 12.0
```

 Copyright 2015 Advanced Analytical Technologies, Inc.

Sample: SampE7
Well Location: E7

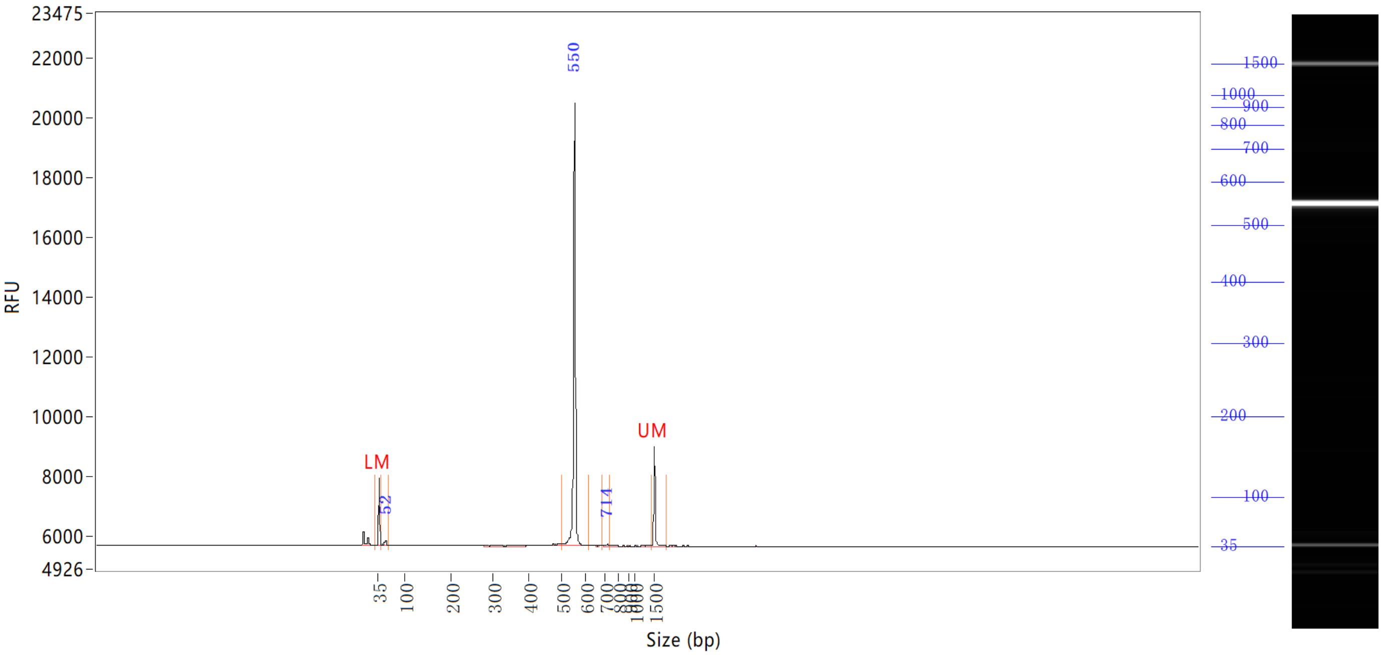

Peak	Size (bp)	Conc. (ng/uL)	From (bp)	To (bp)	Avg. Size (bp)	CV%	RFU	Corr. Peak Area
1	35 (LM)	0.4697	28	41	35	4.43	2264	10.637
2	52	0.8570	41	58	49	6.70	152	1.617
3	550	35.7752	502	612	548	1.19	14782	67.521
4	714	0.3260	682	738	709	1.96	58	0.615
5	1500 (UM)	0.5000	1414	1849	1501	2.45	3301	11.324

TIC:	36.9582	ng/uL
TIM:	136.8274	nmole/L
Total Conc.:	39.4306	ng/uL

```
Sample Peak Width (sec): 5    Sample Min Peak Height: 50    Sample Baseline V to V?: Y    Sample Baseline V to V pts: 3
Sample Filter: Binomial       # of Pts for Filter: 3        Sample Start Region (min): 0  Sample End Region (min): 75
Marker Peak Width (sec): 5    Marker Min Peak Height: 500   Marker Baseline V to V?: Y    Marker Baseline V to V pts: 3
Lower Marker Selection: First Peak > 500 RFU                Upper Marker Selection: Last Peak > 500 RFU
Ladder Size (bp)35, 100, 200, 300, 400, 500, 600, 700, 800, 900, 1000, 1500
Quantification Using: Upper Marker          Final Concentration (ng/uL): 0.5000          Dilution Factor: 12.0
```

 Copyright 2015 Advanced Analytical Technologies, Inc.

Sample: SampF7
Well Location: F7

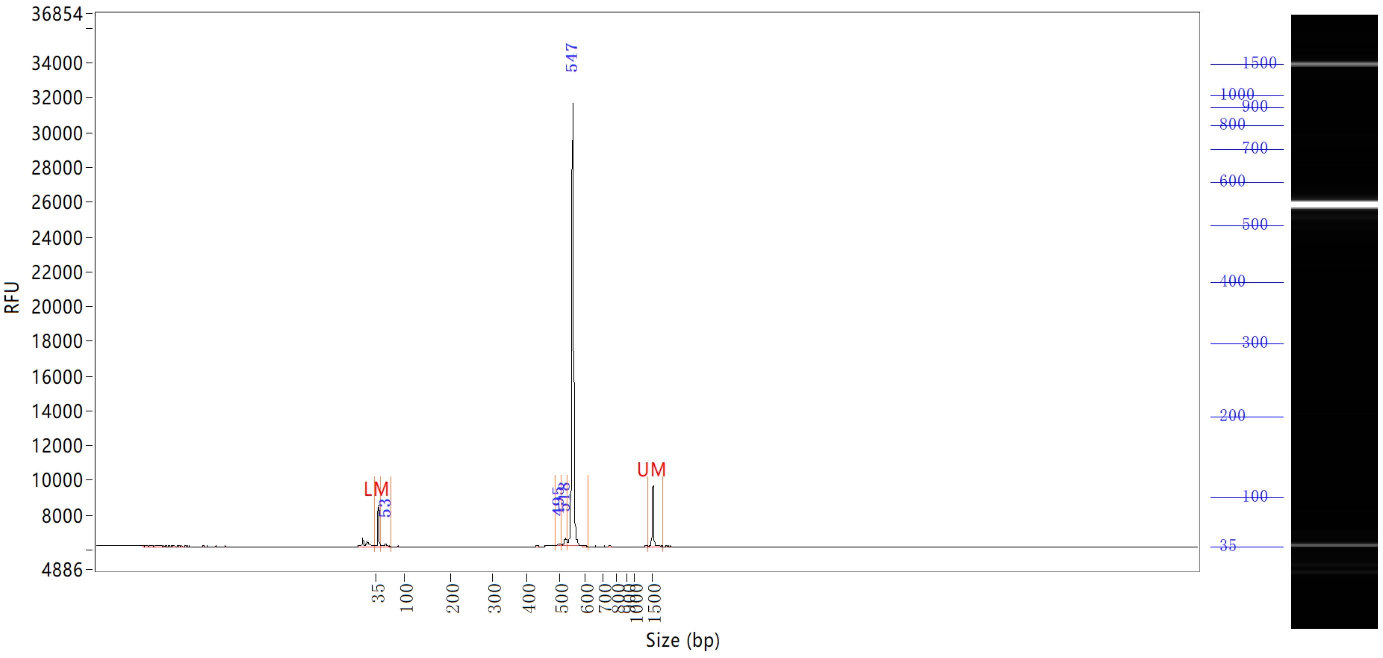

Peak	Size (bp)	Conc. (ng/uL)	From (bp)	To (bp)	Avg. Size (bp)	CV%	RFU	Corr. Peak Area
1	35 (LM)	0.5071	30	43	35	5.33	2413	12.239
2	53	0.8955	43	66	50	9.25	123	1.801
3	495	0.4675	484	505	494	0.98	117	0.940
4	518	1.4094	505	529	518	1.09	407	2.835
5	547	58.3548	529	619	547	0.99	25501	117.368
6	1500 (UM)	0.5000	1394	1790	1497	2.40	3467	12.068

TIC:	61.1272	ng/uL
TIM:	210.9223	nmole/L
Total Conc.:	63.5396	ng/uL

Sample Peak Width (sec): 5   Sample Min Peak Height: 50   Sample Baseline V to V?: Y   Sample Baseline V to V pts: 3
Sample Filter: Binomial   # of Pts for Filter: 3   Sample Start Region (min): 0   Sample End Region (min): 75
Marker Peak Width (sec): 5   Marker Min Peak Height: 500   Marker Baseline V to V?: Y   Marker Baseline V to V pts: 3
Lower Marker Selection: First Peak > 500 RFU   Upper Marker Selection: Last Peak > 500 RFU
Ladder Size (bp)35, 100, 200, 300, 400, 500, 600, 700, 800, 900, 1000, 1500
Quantification Using: Upper Marker   Final Concentration (ng/uL): 0.5000   Dilution Factor: 12.0

 Copyright 2015 Advanced Analytical Technologies, Inc.

Sample: SampG7
Well Location: G7

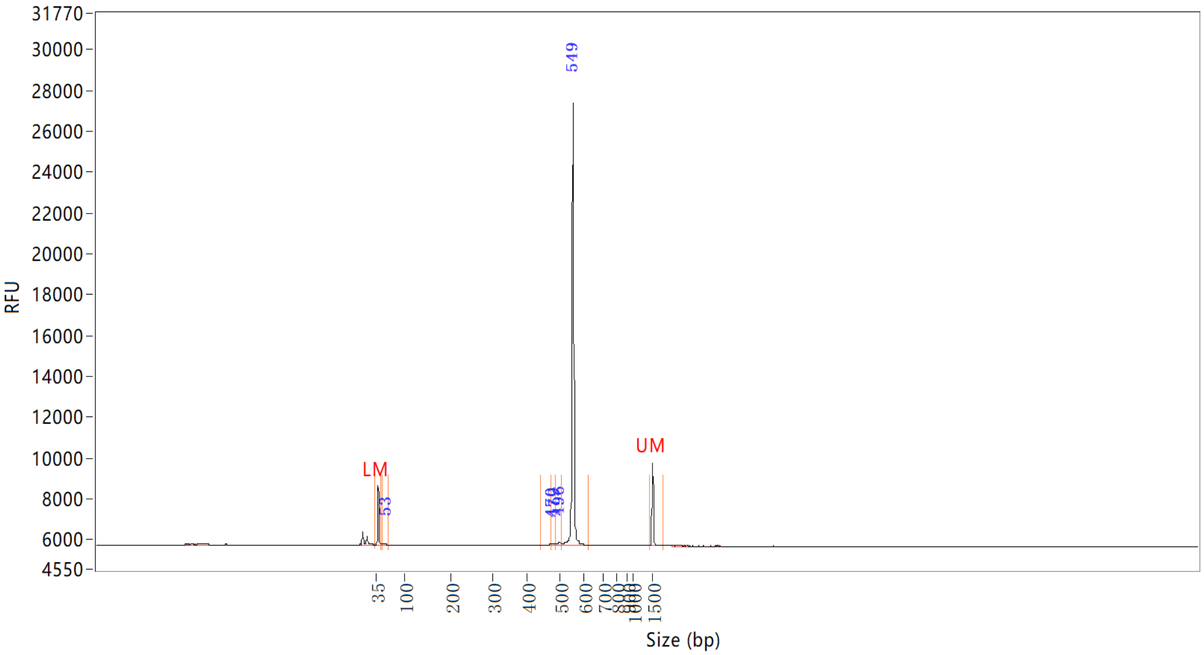

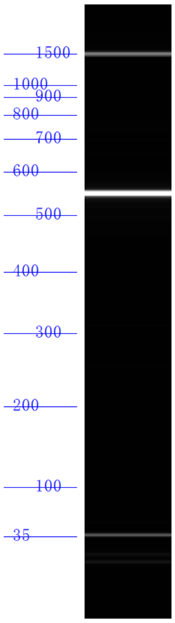

Peak	Size (bp)	Conc. (ng/uL)	From (bp)	To (bp)	Avg. Size (bp)	CV%	RFU	Corr. Peak Area
1	35 (LM)	0.4889	29	43	35	4.58	2895	13.496
2	53	0.2721	49	60	52	4.23	85	0.626
3	470	0.2543	441	472	464	1.50	68	0.585
4	479	0.3743	472	488	480	0.95	83	0.861
5	496	0.6012	488	508	497	1.10	155	1.383
6	549	42.2807	508	618	548	1.29	21695	97.262
7	1500 (UM)	0.5000	1414	1803	1497	2.68	4045	13.802

TIC:	43.7826	ng/uL
TIM:	139.7112	nmole/L
Total Conc.:	45.6579	ng/uL

```
Sample Peak Width (sec): 5    Sample Min Peak Height: 50    Sample Baseline V to V?: Y    Sample Baseline V to V pts: 3
Sample Filter: Binomial       # of Pts for Filter: 3        Sample Start Region (min): 0  Sample End Region (min): 75
Marker Peak Width (sec): 5    Marker Min Peak Height: 500   Marker Baseline V to V?: Y    Marker Baseline V to V pts: 3
Lower Marker Selection: First Peak > 500 RFU                Upper Marker Selection: Last Peak > 500 RFU
Ladder Size (bp)35, 100, 200, 300, 400, 500, 600, 700, 800, 900, 1000, 1500
Quantification Using: Upper Marker          Final Concentration (ng/uL): 0.5000          Dilution Factor: 12.0
```

 Copyright 2015 Advanced Analytical Technologies, Inc.

Sample: SampH7
Well Location: H7

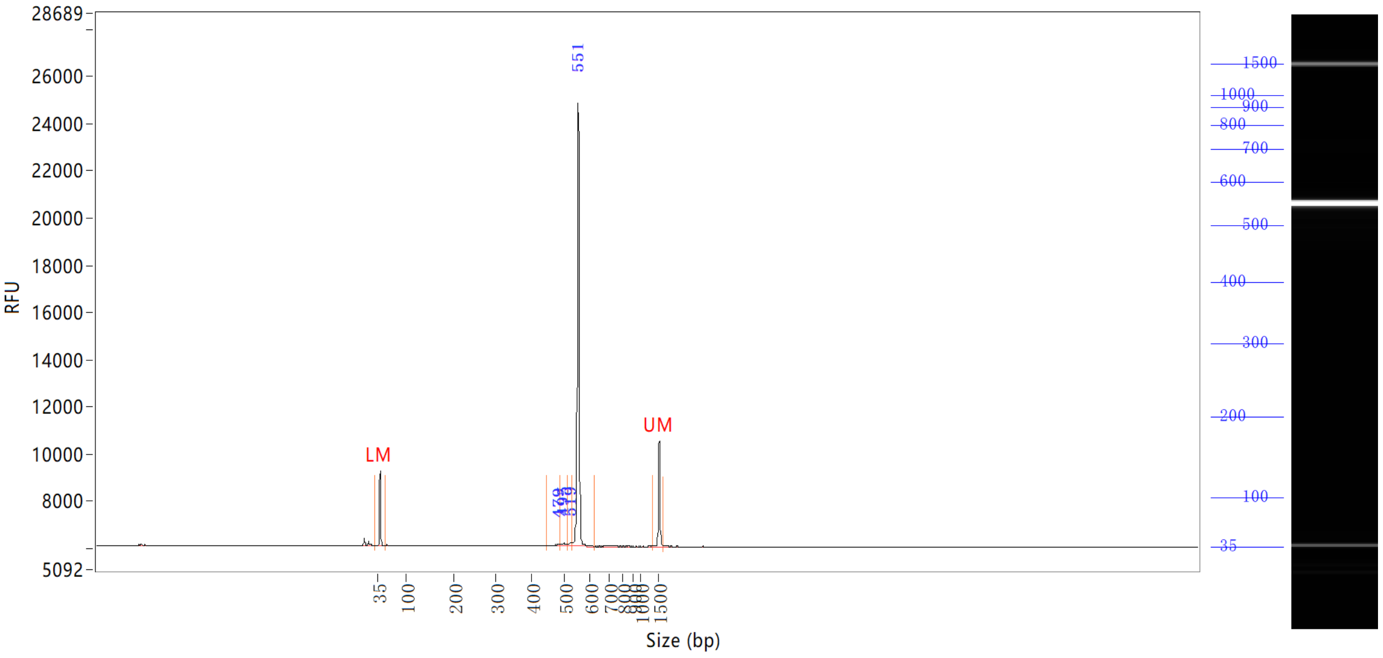

Peak	Size (bp)	Conc. (ng/uL)	From (bp)	To (bp)	Avg. Size (bp)	CV%	RFU	Corr. Peak Area
1	35 (LM)	0.5046	24	48	35	5.82	3192	15.112
2	479	0.5148	443	485	471	2.14	78	1.285
3	495	0.6056	485	510	497	1.35	132	1.511
4	519	0.5393	510	528	520	1.03	153	1.346
5	551	34.4827	528	621	549	0.99	18835	86.065
6	1500 (UM)	0.5000	1340	1632	1492	1.66	4506	14.975

TIC:	36.1423	ng/uL
TIM:	108.8690	nmole/L
Total Conc.:	37.7799	ng/uL

```
Sample Peak Width (sec): 5    Sample Min Peak Height: 50    Sample Baseline V to V?: Y    Sample Baseline V to V pts: 3
Sample Filter: Binomial       # of Pts for Filter: 3        Sample Start Region (min): 0  Sample End Region (min): 75
Marker Peak Width (sec): 5    Marker Min Peak Height: 500   Marker Baseline V to V?: Y    Marker Baseline V to V pts: 3
Lower Marker Selection: First Peak > 500 RFU                Upper Marker Selection: Last Peak > 500 RFU
Ladder Size (bp)35, 100, 200, 300, 400, 500, 600, 700, 800, 900, 1000, 1500
Quantification Using: Upper Marker          Final Concentration (ng/uL): 0.5000          Dilution Factor: 12.0
```

 Copyright 2015 Advanced Analytical Technologies, Inc.

Sample: SampA8
Well Location: A8

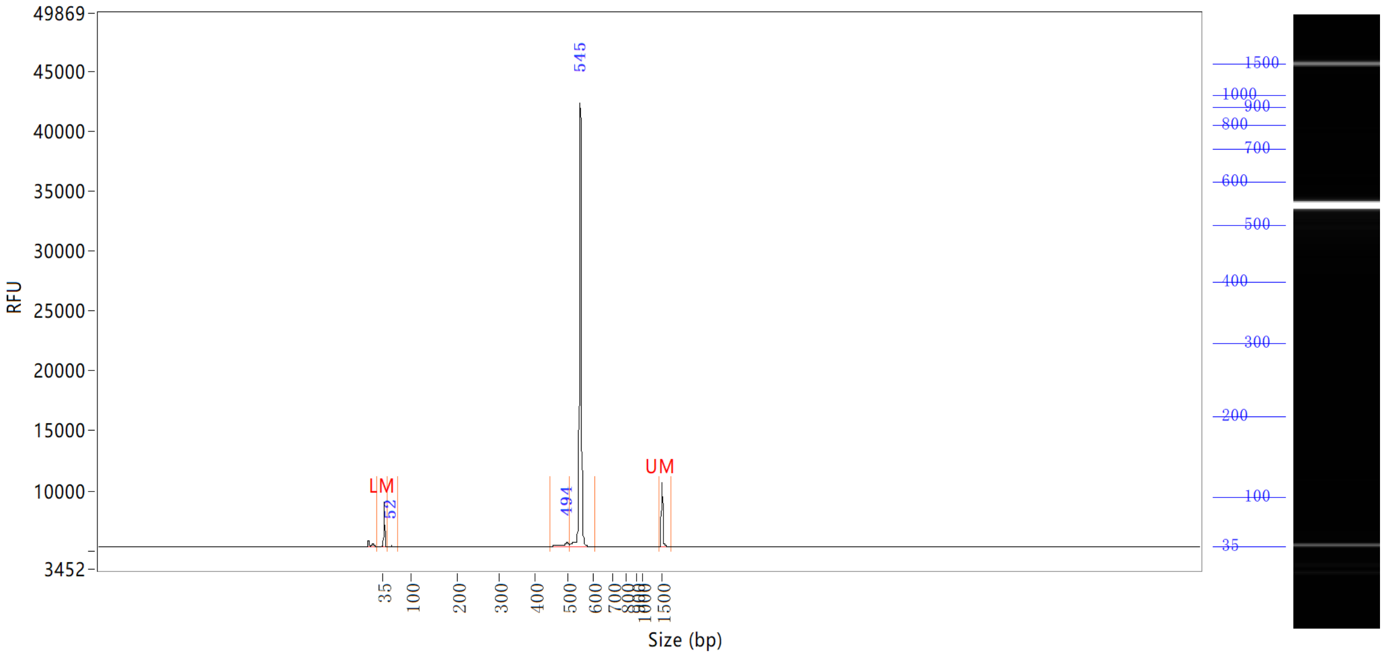

Peak	Size (bp)	Conc. (ng/uL)	From (bp)	To (bp)	Avg. Size (bp)	CV%	RFU	Corr. Peak Area
1	35 (LM)	0.5113	20	44	34	6.81	3778	18.503
2	52	0.2951	44	67	51	9.69	71	0.890
3	494	1.9658	444	503	481	3.04	327	5.928
4	545	58.6180	503	611	544	1.19	37117	176.780
5	1500 (UM)	0.5000	1420	1757	1497	1.80	5323	18.095

TIC:	60.8788	ng/uL
TIM:	193.5180	nmole/L
Total Conc.:	62.4858	ng/uL

```
Sample Peak Width (sec): 5    Sample Min Peak Height: 50    Sample Baseline V to V?: Y    Sample Baseline V to V pts: 3
Sample Filter: Binomial       # of Pts for Filter: 3        Sample Start Region (min): 0  Sample End Region (min): 75
Marker Peak Width (sec): 5    Marker Min Peak Height: 500   Marker Baseline V to V?: Y    Marker Baseline V to V pts: 3
Lower Marker Selection: First Peak > 500 RFU                Upper Marker Selection: Last Peak > 500 RFU
Ladder Size (bp)35, 100, 200, 300, 400, 500, 600, 700, 800, 900, 1000, 1500
Quantification Using: Upper Marker          Final Concentration (ng/uL): 0.5000          Dilution Factor: 12.0
```

 Copyright 2015 Advanced Analytical Technologies, Inc.

Sample: SampB8
Well Location: B8

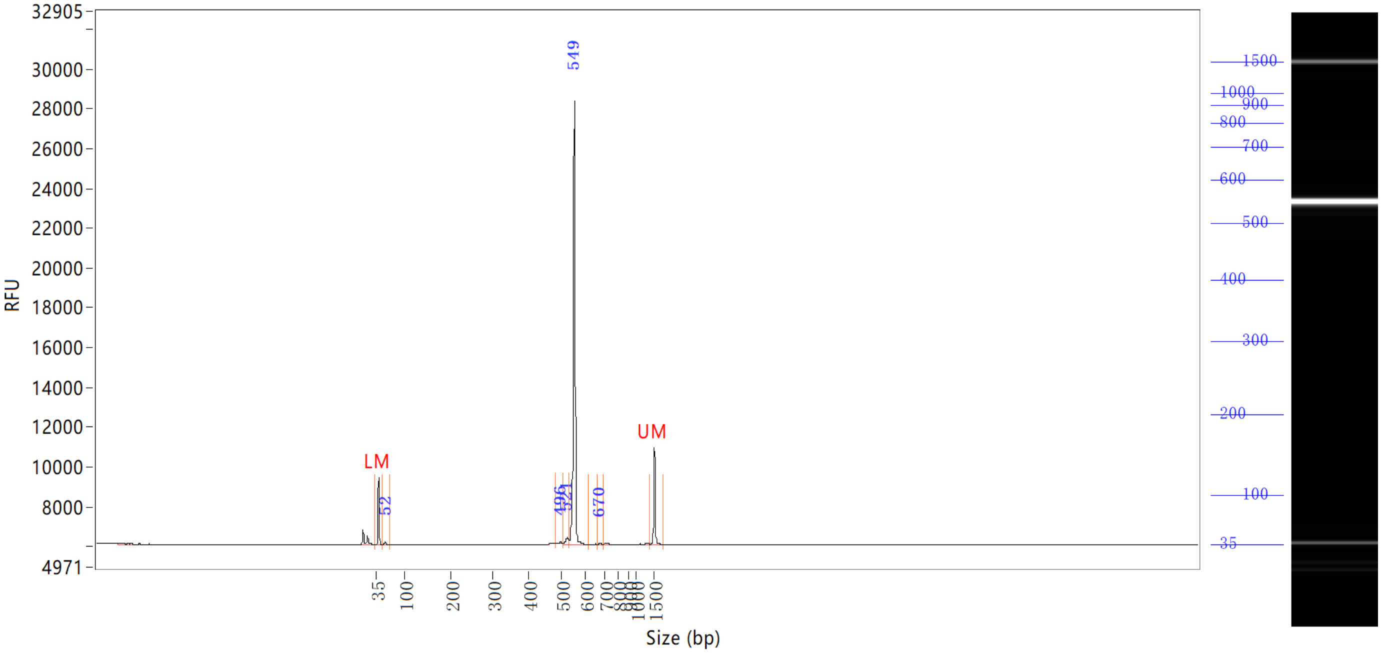

Peak	Size (bp)	Conc. (ng/uL)	From (bp)	To (bp)	Avg. Size (bp)	CV%	RFU	Corr. Peak Area
1	35 (LM)	0.4924	29	45	35	4.88	3352	16.155
2	52	0.4977	45	63	51	6.24	129	1.361
3	496	0.2818	482	504	494	1.11	82	0.771
4	521	0.8241	504	528	519	1.13	310	2.253
5	549	36.9701	528	615	548	0.96	22311	101.085
6	670	0.1663	656	687	671	1.12	62	0.455
7	1500 (UM)	0.5000	1400	1744	1498	1.87	4853	16.405

TIC:	38.7400	ng/uL
TIM:	130.9618	nmole/L
Total Conc.:	40.4470	ng/uL

Sample Peak Width (sec): 5 Sample Min Peak Height: 50 Sample Baseline V to V?: Y Sample Baseline V to V pts: 3
Sample Filter: Binomial # of Pts for Filter: 3 Sample Start Region (min): 0 Sample End Region (min): 75
Marker Peak Width (sec): 5 Marker Min Peak Height: 500 Marker Baseline V to V?: Y Marker Baseline V to V pts: 3
Lower Marker Selection: First Peak > 500 RFU Upper Marker Selection: Last Peak > 500 RFU
Ladder Size (bp)35, 100, 200, 300, 400, 500, 600, 700, 800, 900, 1000, 1500
Quantification Using: Upper Marker Final Concentration (ng/uL): 0.5000 Dilution Factor: 12.0

 Copyright 2015 Advanced Analytical Technologies, Inc.

Sample: SampC8
Well Location: C8

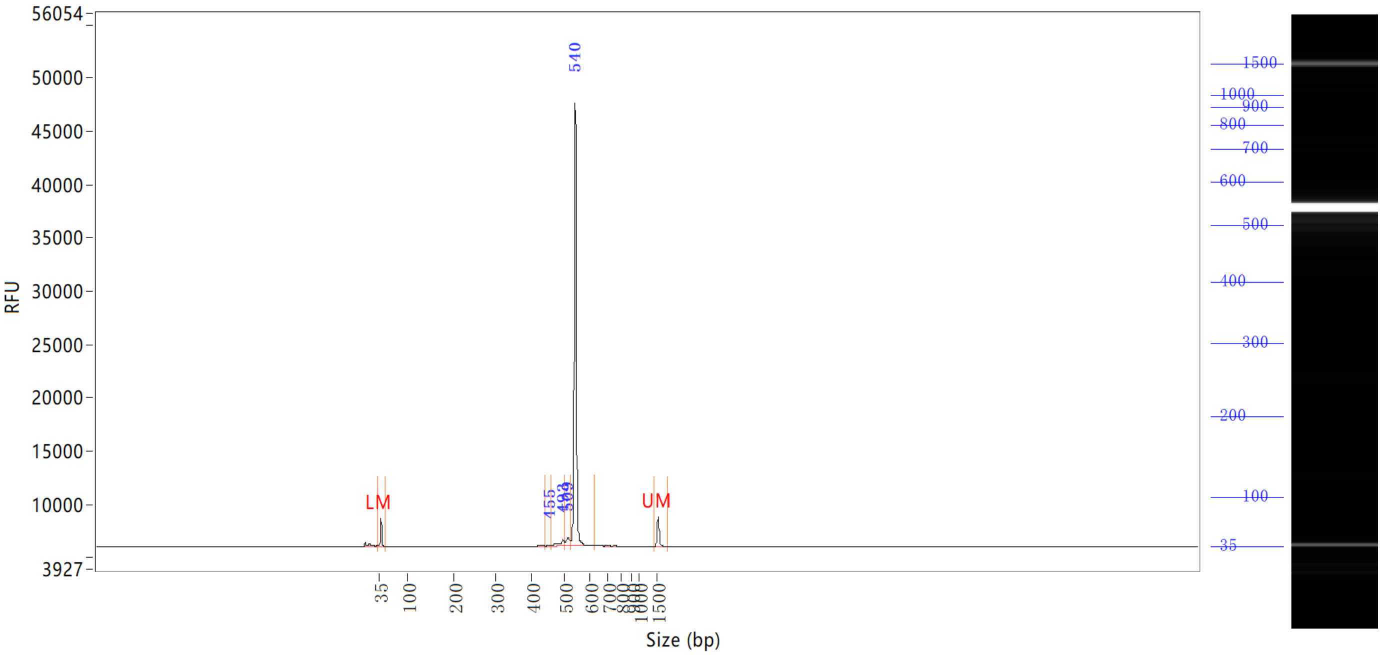

Peak	Size (bp)	Conc. (ng/uL)	From (bp)	To (bp)	Avg. Size (bp)	CV%	RFU	Corr. Peak Area
1	35 (LM)	0.5351	29	49	35	5.39	2559	12.642
2	455	0.1889	441	457	452	0.86	58	0.372
3	493	4.0361	457	500	484	2.28	578	7.946
4	509	3.5255	500	523	511	1.22	807	6.941
5	540	113.2977	523	624	540	1.14	41609	223.056
6	1500 (UM)	0.5000	1407	1783	1501	2.28	2750	11.813

TIC:	121.0482	ng/uL
TIM:	371.0199	nmole/L
Total Conc.:	123.3513	ng/uL

```
Sample Peak Width (sec): 5    Sample Min Peak Height: 50    Sample Baseline V to V?: Y    Sample Baseline V to V pts: 3
Sample Filter: Binomial       # of Pts for Filter: 3        Sample Start Region (min): 0  Sample End Region (min): 75
Marker Peak Width (sec): 5    Marker Min Peak Height: 500   Marker Baseline V to V?: Y    Marker Baseline V to V pts: 3
Lower Marker Selection: First Peak > 500 RFU                Upper Marker Selection: Last Peak > 500 RFU
Ladder Size (bp)35, 100, 200, 300, 400, 500, 600, 700, 800, 900, 1000, 1500
Quantification Using: Upper Marker          Final Concentration (ng/uL): 0.5000          Dilution Factor: 12.0
```

 Copyright 2015 Advanced Analytical Technologies, Inc.

Sample: SampD8
Well Location: D8

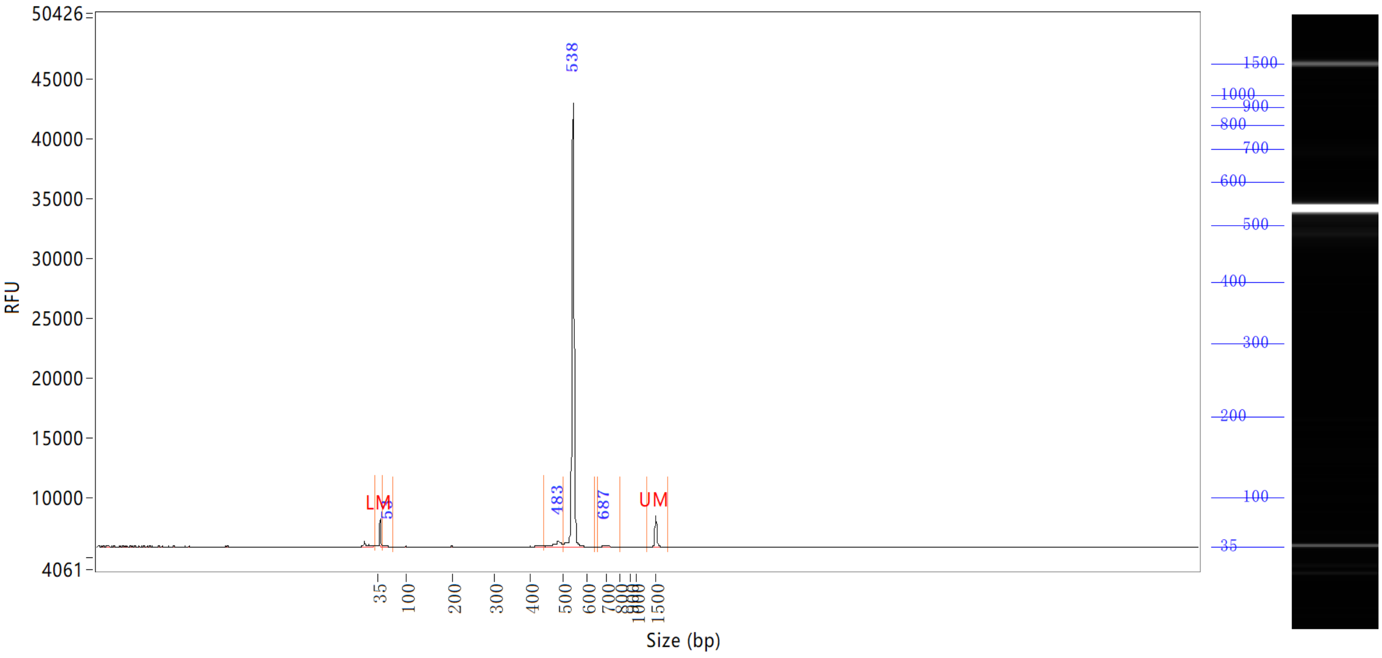

Peak	Size (bp)	Conc. (ng/uL)	From (bp)	To (bp)	Avg. Size (bp)	CV%	RFU	Corr. Peak Area
1	35 (LM)	0.4971	25	44	34	5.61	2285	11.339
2	53	0.3572	44	67	52	9.16	63	0.679
3	483	3.9763	443	499	480	2.52	488	7.558
4	538	103.6489	499	634	537	1.27	37036	197.017
5	687	0.7976	653	798	698	3.56	106	1.516
6	1500 (UM)	0.5000	1254	1849	1495	3.04	2588	11.405

TIC:	108.7800	ng/uL
TIM:	344.3842	nmole/L
Total Conc.:	111.1415	ng/uL

Sample Peak Width (sec): 5   Sample Min Peak Height: 50   Sample Baseline V to V?: Y   Sample Baseline V to V pts: 3
Sample Filter: Binomial   # of Pts for Filter: 3   Sample Start Region (min): 0   Sample End Region (min): 75
Marker Peak Width (sec): 5   Marker Min Peak Height: 500   Marker Baseline V to V?: Y   Marker Baseline V to V pts: 3
Lower Marker Selection: First Peak > 500 RFU   Upper Marker Selection: Last Peak > 500 RFU
Ladder Size (bp)35, 100, 200, 300, 400, 500, 600, 700, 800, 900, 1000, 1500
Quantification Using: Upper Marker   Final Concentration (ng/uL): 0.5000   Dilution Factor: 12.0

 Copyright 2015 Advanced Analytical Technologies, Inc.

Sample: SampE8
Well Location: E8

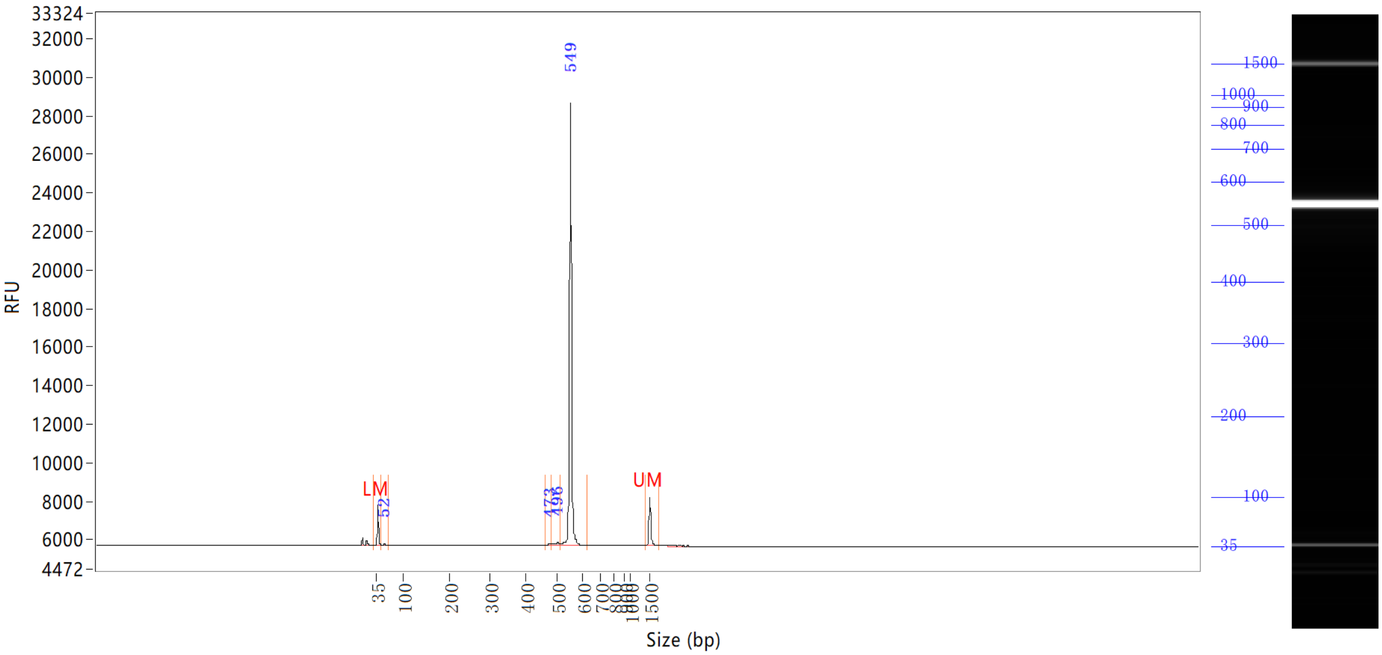

Peak	Size (bp)	Conc. (ng/uL)	From (bp)	To (bp)	Avg. Size (bp)	CV%	RFU	Corr. Peak Area
1	35 (LM)	0.4895	27	45	35	5.87	2073	10.126
2	52	0.5121	45	62	51	6.77	83	0.883
3	473	0.4305	458	477	469	1.10	76	0.742
4	496	1.2739	477	509	493	1.71	154	2.196
5	549	66.4124	509	626	548	1.30	23011	114.486
6	1500 (UM)	0.5000	1374	1763	1505	3.07	2536	10.343

TIC:	68.6290	ng/uL
TIM:	221.6402	nmole/L
Total Conc.:	70.9839	ng/uL

Sample Peak Width (sec): 5   Sample Min Peak Height: 50   Sample Baseline V to V?: Y   Sample Baseline V to V pts: 3
Sample Filter: Binomial   # of Pts for Filter: 3   Sample Start Region (min): 0   Sample End Region (min): 75
Marker Peak Width (sec): 5   Marker Min Peak Height: 500   Marker Baseline V to V?: Y   Marker Baseline V to V pts: 3
Lower Marker Selection: First Peak > 500 RFU   Upper Marker Selection: Last Peak > 500 RFU
Ladder Size (bp)35, 100, 200, 300, 400, 500, 600, 700, 800, 900, 1000, 1500
Quantification Using: Upper Marker   Final Concentration (ng/uL): 0.5000   Dilution Factor: 12.0

 Copyright 2015 Advanced Analytical Technologies, Inc.

Sample: SampF8
Well Location: F8

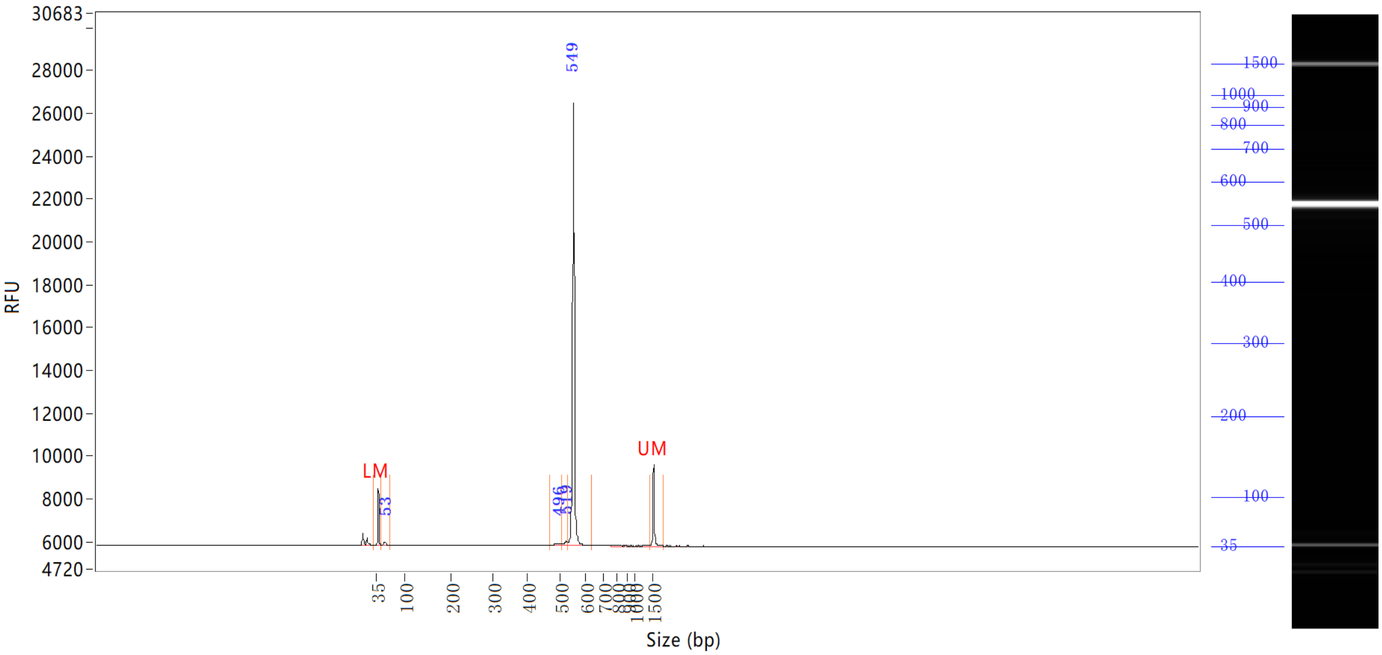

Peak	Size (bp)	Conc. (ng/uL)	From (bp)	To (bp)	Avg. Size (bp)	CV%	RFU	Corr. Peak Area
1	35 (LM)	0.4745	25	44	35	5.02	2627	12.495
2	53	0.7410	44	66	51	7.22	132	1.626
3	496	0.8317	466	504	487	2.13	118	1.825
4	519	0.8781	504	527	517	1.21	216	1.927
5	549	43.3101	527	635	548	1.26	20719	95.042
6	1500 (UM)	0.5000	1414	1796	1495	2.25	3791	13.167

TIC:	45.7607	ng/uL
TIM:	159.4580	nmole/L
Total Conc.:	47.8399	ng/uL

```
Sample Peak Width (sec): 5    Sample Min Peak Height: 50    Sample Baseline V to V?: Y    Sample Baseline V to V pts: 3
Sample Filter: Binomial       # of Pts for Filter: 3        Sample Start Region (min): 0  Sample End Region (min): 75
Marker Peak Width (sec): 5    Marker Min Peak Height: 500   Marker Baseline V to V?: Y    Marker Baseline V to V pts: 3
Lower Marker Selection: First Peak > 500 RFU                Upper Marker Selection: Last Peak > 500 RFU
Ladder Size (bp)35, 100, 200, 300, 400, 500, 600, 700, 800, 900, 1000, 1500
Quantification Using: Upper Marker          Final Concentration (ng/uL): 0.5000          Dilution Factor: 12.0
```

 Copyright 2015 Advanced Analytical Technologies, Inc.

Sample: SampG8
Well Location: G8

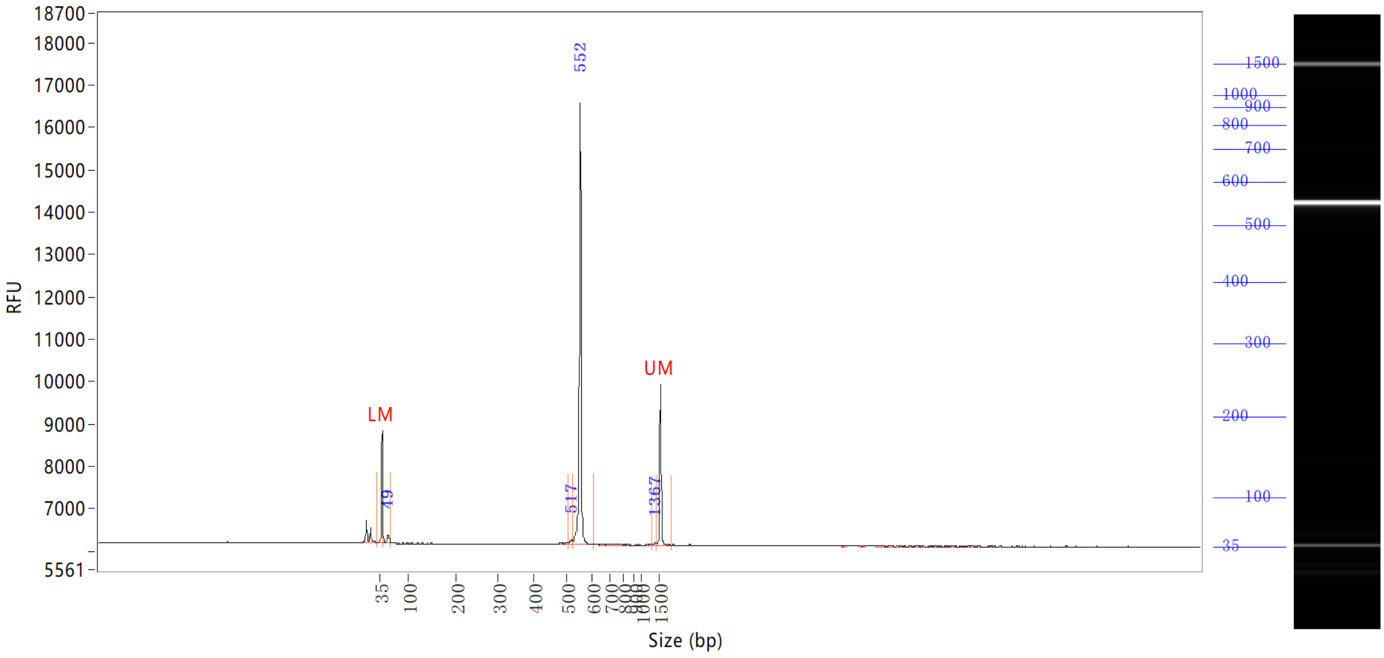

Peak	Size (bp)	Conc. (ng/uL)	From (bp)	To (bp)	Avg. Size (bp)	CV%	RFU	Corr. Peak Area
1	35 (LM)	0.4945	26	41	35	4.77	2665	12.650
2	49	0.9106	41	57	49	5.73	192	1.941
3	517	0.3311	505	523	515	0.90	107	0.706
4	552	21.8145	523	611	550	1.19	10443	46.500
5	1367	0.1816	1267	1420	1355	2.80	57	0.387
6	1500 (UM)	0.5000	1420	1809	1493	1.94	3818	12.790

TIC:	23.2377	ng/uL
TIM:	96.9805	nmole/L
Total Conc.:	25.2254	ng/uL

```
Sample Peak Width (sec): 5    Sample Min Peak Height: 50    Sample Baseline V to V?: Y    Sample Baseline V to V pts: 3
Sample Filter: Binomial       # of Pts for Filter: 3        Sample Start Region (min): 0  Sample End Region (min): 75
Marker Peak Width (sec): 5    Marker Min Peak Height: 500   Marker Baseline V to V?: Y    Marker Baseline V to V pts: 3
Lower Marker Selection: First Peak > 500 RFU                Upper Marker Selection: Last Peak > 500 RFU
Ladder Size (bp)35, 100, 200, 300, 400, 500, 600, 700, 800, 900, 1000, 1500
Quantification Using: Upper Marker          Final Concentration (ng/uL): 0.5000          Dilution Factor: 12.0
```

 Copyright 2015 Advanced Analytical Technologies, Inc.

Sample: SampH8
Well Location: H8

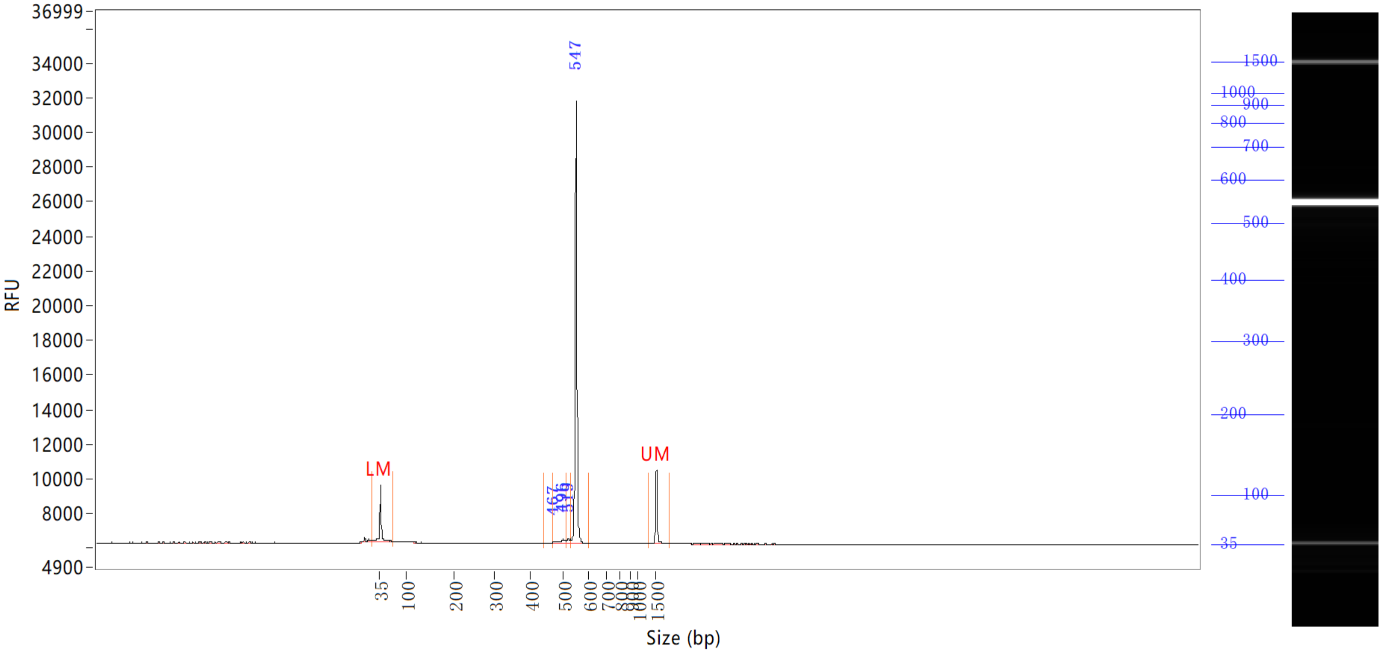

Peak	Size (bp)	Conc. (ng/uL)	From (bp)	To (bp)	Avg. Size (bp)	CV%	RFU	Corr. Peak Area
1	35 (LM)	0.7437	15	66	34	20.23	3292	22.517
2	467	0.2116	441	468	460	1.42	78	0.534
3	496	1.4230	468	508	489	2.21	223	3.590
4	519	0.9604	508	529	519	1.18	246	2.423
5	547	46.5997	529	605	547	0.93	25594	117.579
6	1500 (UM)	0.5000	1287	1869	1500	3.41	4289	15.139

TIC:	49.1946	ng/uL
TIM:	148.7794	nmole/L
Total Conc.:	50.7643	ng/uL

Sample Peak Width (sec): 5 Sample Min Peak Height: 50 Sample Baseline V to V?: Y Sample Baseline V to V pts: 3
Sample Filter: Binomial # of Pts for Filter: 3 Sample Start Region (min): 0 Sample End Region (min): 75
Marker Peak Width (sec): 5 Marker Min Peak Height: 500 Marker Baseline V to V?: Y Marker Baseline V to V pts: 3
Lower Marker Selection: First Peak > 500 RFU Upper Marker Selection: Last Peak > 500 RFU
Ladder Size (bp)35, 100, 200, 300, 400, 500, 600, 700, 800, 900, 1000, 1500
Quantification Using: Upper Marker Final Concentration (ng/uL): 0.5000 Dilution Factor: 12.0

 Copyright 2015 Advanced Analytical Technologies, Inc.

Sample: SampA9
Well Location: A9

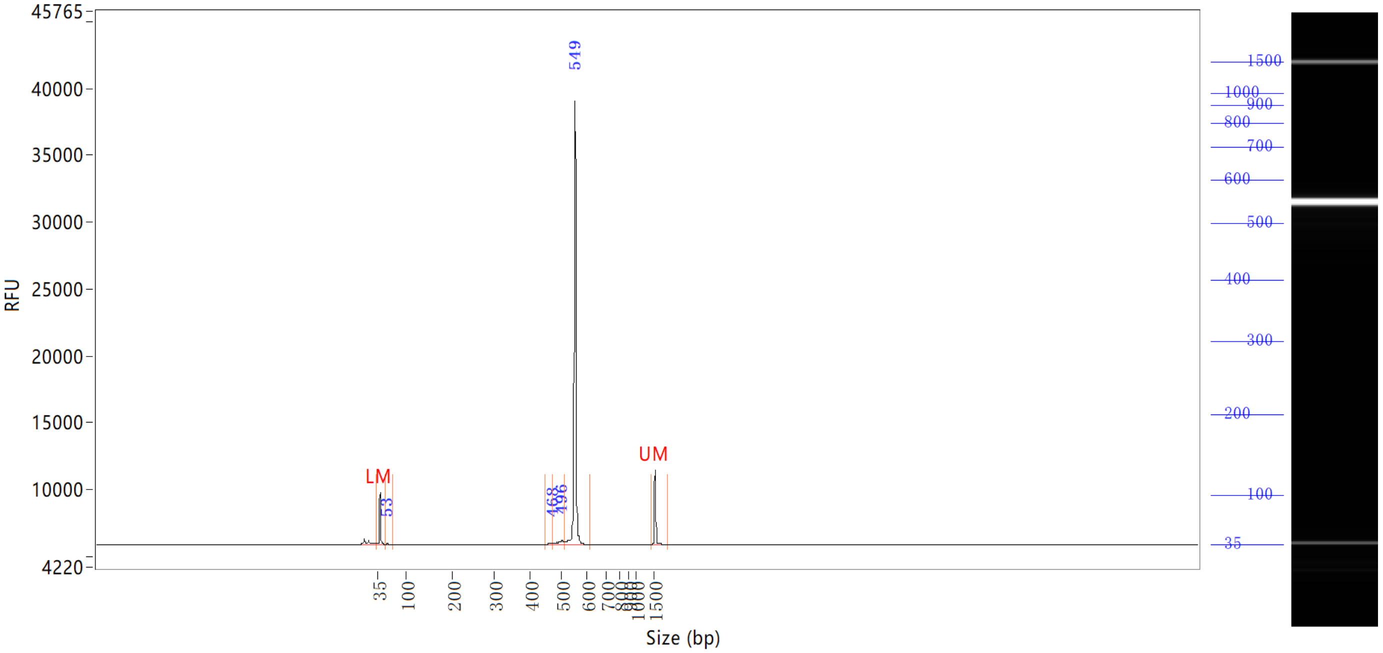

Peak	Size (bp)	Conc. (ng/uL)	From (bp)	To (bp)	Avg. Size (bp)	CV%	RFU	Corr. Peak Area
1	35 (LM)	0.5068	29	48	35	6.61	3899	19.170
2	53	0.2336	48	66	54	8.21	61	0.736
3	468	0.2329	445	468	461	1.32	101	0.734
4	496	1.4236	468	506	489	2.05	309	4.487
5	549	49.8543	506	619	547	1.24	33209	157.140
6	1500 (UM)	0.5000	1400	1855	1497	2.35	5620	18.912

TIC:	51.7444	ng/uL
TIM:	162.6893	nmole/L
Total Conc.:	53.0402	ng/uL

Sample Peak Width (sec): 5 Sample Min Peak Height: 50 Sample Baseline V to V?: Y Sample Baseline V to V pts: 3
Sample Filter: Binomial # of Pts for Filter: 3 Sample Start Region (min): 0 Sample End Region (min): 75
Marker Peak Width (sec): 5 Marker Min Peak Height: 500 Marker Baseline V to V?: Y Marker Baseline V to V pts: 3
Lower Marker Selection: First Peak > 500 RFU Upper Marker Selection: Last Peak > 500 RFU
Ladder Size (bp)35, 100, 200, 300, 400, 500, 600, 700, 800, 900, 1000, 1500
Quantification Using: Upper Marker Final Concentration (ng/uL): 0.5000 Dilution Factor: 12.0

 Copyright 2015 Advanced Analytical Technologies, Inc.

Sample: SampB9
Well Location: B9

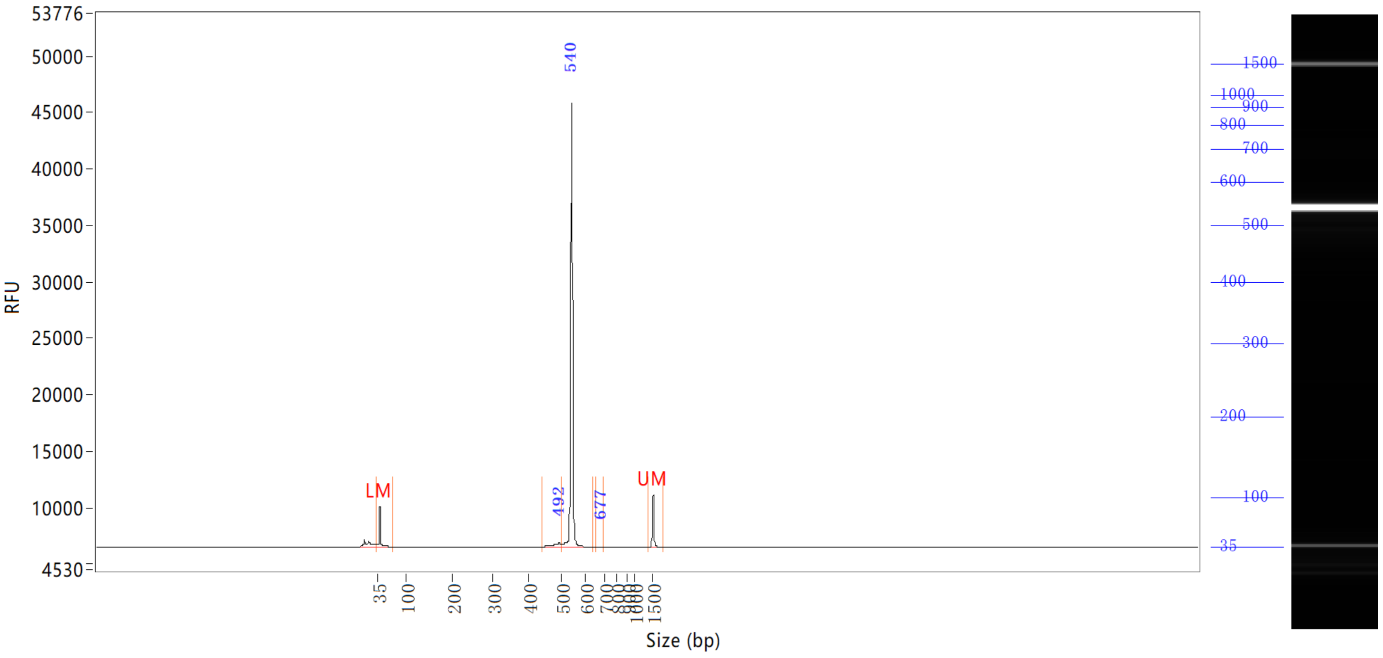

Peak	Size (bp)	Conc. (ng/uL)	From (bp)	To (bp)	Avg. Size (bp)	CV%	RFU	Corr. Peak Area
1	35 (LM)	0.6578	28	67	36	14.42	3620	21.749
2	492	2.4532	442	499	480	2.84	394	6.759
3	540	68.3784	499	640	539	1.33	39364	188.388
4	677	0.1410	654	694	677	1.27	51	0.388
5	1500 (UM)	0.5000	1380	1776	1495	2.20	4591	16.530

TIC:	70.9726	ng/uL
TIM:	217.5118	nmole/L
Total Conc.:	72.6550	ng/uL

```
Sample Peak Width (sec): 5    Sample Min Peak Height: 50    Sample Baseline V to V?: Y    Sample Baseline V to V pts: 3
Sample Filter: Binomial       # of Pts for Filter: 3        Sample Start Region (min): 0  Sample End Region (min): 75
Marker Peak Width (sec): 5    Marker Min Peak Height: 500   Marker Baseline V to V?: Y    Marker Baseline V to V pts: 3
Lower Marker Selection: First Peak > 500 RFU                Upper Marker Selection: Last Peak > 500 RFU
Ladder Size (bp)35, 100, 200, 300, 400, 500, 600, 700, 800, 900, 1000, 1500
Quantification Using: Upper Marker          Final Concentration (ng/uL): 0.5000          Dilution Factor: 12.0
```

 Copyright 2015 Advanced Analytical Technologies, Inc.

Sample: SampC9
Well Location: C9

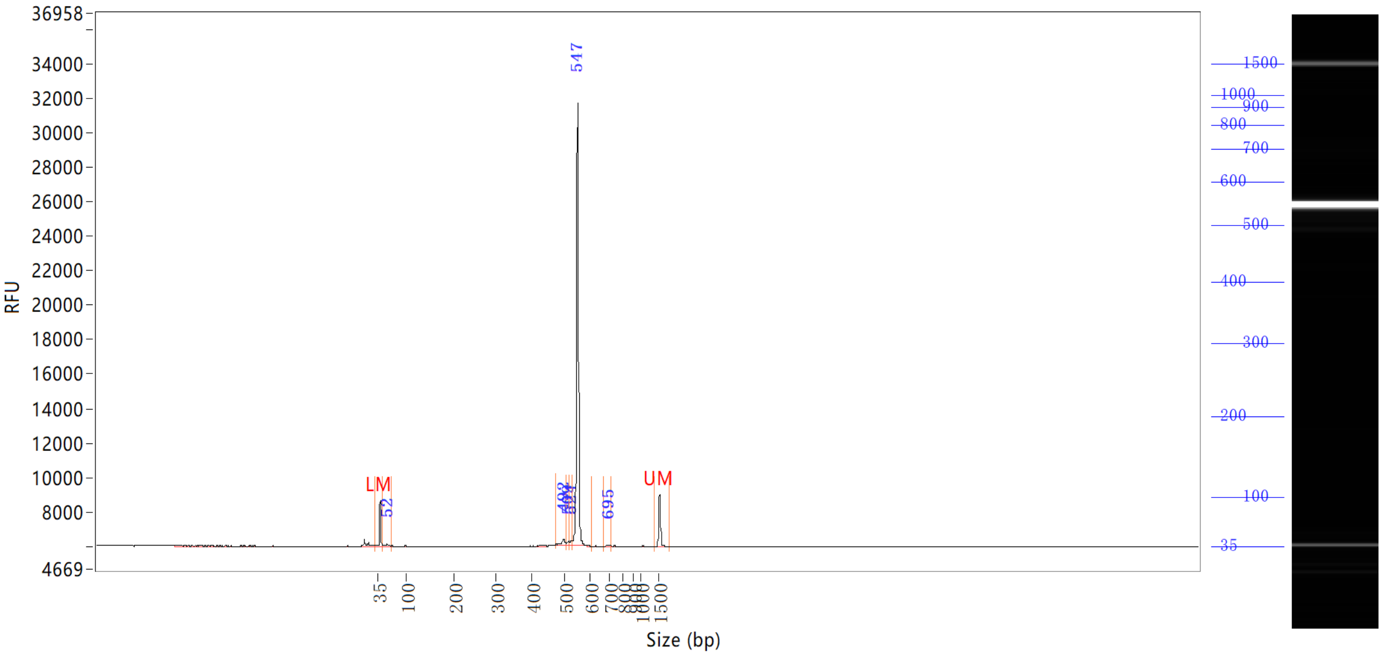

Peak	Size (bp)	Conc. (ng/uL)	From (bp)	To (bp)	Avg. Size (bp)	CV%	RFU	Corr. Peak Area
1	35 (LM)	0.5767	24	44	35	6.69	2672	13.365
2	52	0.7162	44	65	51	8.83	101	1.383
3	492	1.2823	473	500	491	0.95	365	2.477
4	509	0.6263	500	513	507	0.73	208	1.210
5	524	0.9192	513	526	520	0.80	259	1.775
6	547	63.4542	526	609	546	0.95	25749	122.556
7	695	0.2520	667	714	690	1.59	54	0.487
8	1500 (UM)	0.5000	1394	1776	1499	2.05	3048	11.588

TIC:	67.2502	ng/uL
TIM:	224.0850	nmole/L
Total Conc.:	69.1666	ng/uL

```
Sample Peak Width (sec): 5    Sample Min Peak Height: 50    Sample Baseline V to V?: Y    Sample Baseline V to V pts: 3
Sample Filter: Binomial       # of Pts for Filter: 3        Sample Start Region (min): 0  Sample End Region (min): 75
Marker Peak Width (sec): 5    Marker Min Peak Height: 500   Marker Baseline V to V?: Y    Marker Baseline V to V pts: 3
Lower Marker Selection: First Peak > 500 RFU                Upper Marker Selection: Last Peak > 500 RFU
Ladder Size (bp)35, 100, 200, 300, 400, 500, 600, 700, 800, 900, 1000, 1500
Quantification Using: Upper Marker          Final Concentration (ng/uL): 0.5000          Dilution Factor: 12.0
```

 Copyright 2015 Advanced Analytical Technologies, Inc.

Sample: SampD9
Well Location: D9

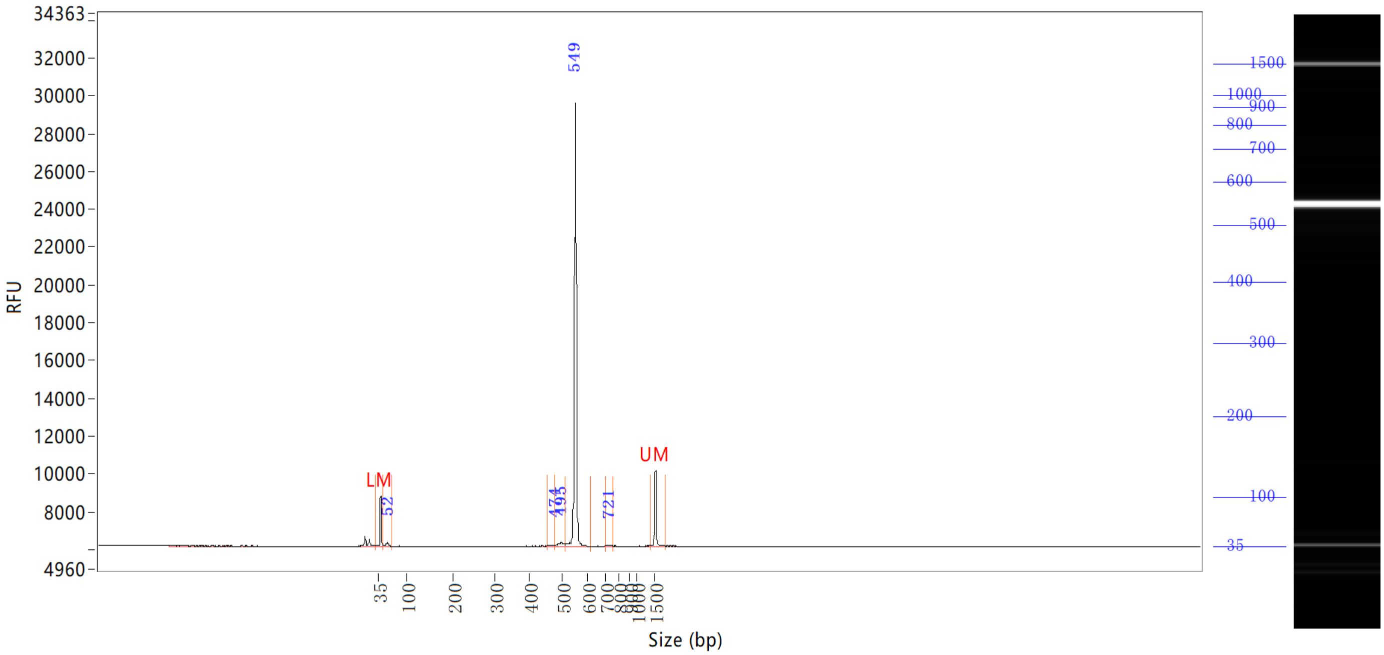

Peak	Size (bp)	Conc. (ng/uL)	From (bp)	To (bp)	Avg. Size (bp)	CV%	RFU	Corr. Peak Area
1	35 (LM)	0.4849	24	42	34	5.90	2684	13.241
2	52	0.9792	42	63	50	7.24	200	2.228
3	474	0.3736	452	477	467	1.37	80	0.850
4	495	1.0978	477	508	492	1.62	175	2.498
5	549	48.5035	508	614	547	1.16	23488	110.371
6	721	0.2798	696	745	716	1.78	66	0.637
7	1500 (UM)	0.5000	1394	1796	1495	2.20	4016	13.653

TIC:	51.2339	ng/uL
TIM:	183.6237	nmole/L
Total Conc.:	52.7282	ng/uL

Sample Peak Width (sec): 5 Sample Min Peak Height: 50 Sample Baseline V to V?: Y Sample Baseline V to V pts: 3
Sample Filter: Binomial # of Pts for Filter: 3 Sample Start Region (min): 0 Sample End Region (min): 75
Marker Peak Width (sec): 5 Marker Min Peak Height: 500 Marker Baseline V to V?: Y Marker Baseline V to V pts: 3
Lower Marker Selection: First Peak > 500 RFU Upper Marker Selection: Last Peak > 500 RFU
Ladder Size (bp)35, 100, 200, 300, 400, 500, 600, 700, 800, 900, 1000, 1500
Quantification Using: Upper Marker Final Concentration (ng/uL): 0.5000 Dilution Factor: 12.0

 Copyright 2015 Advanced Analytical Technologies, Inc.

Sample: SampE9
Well Location: E9

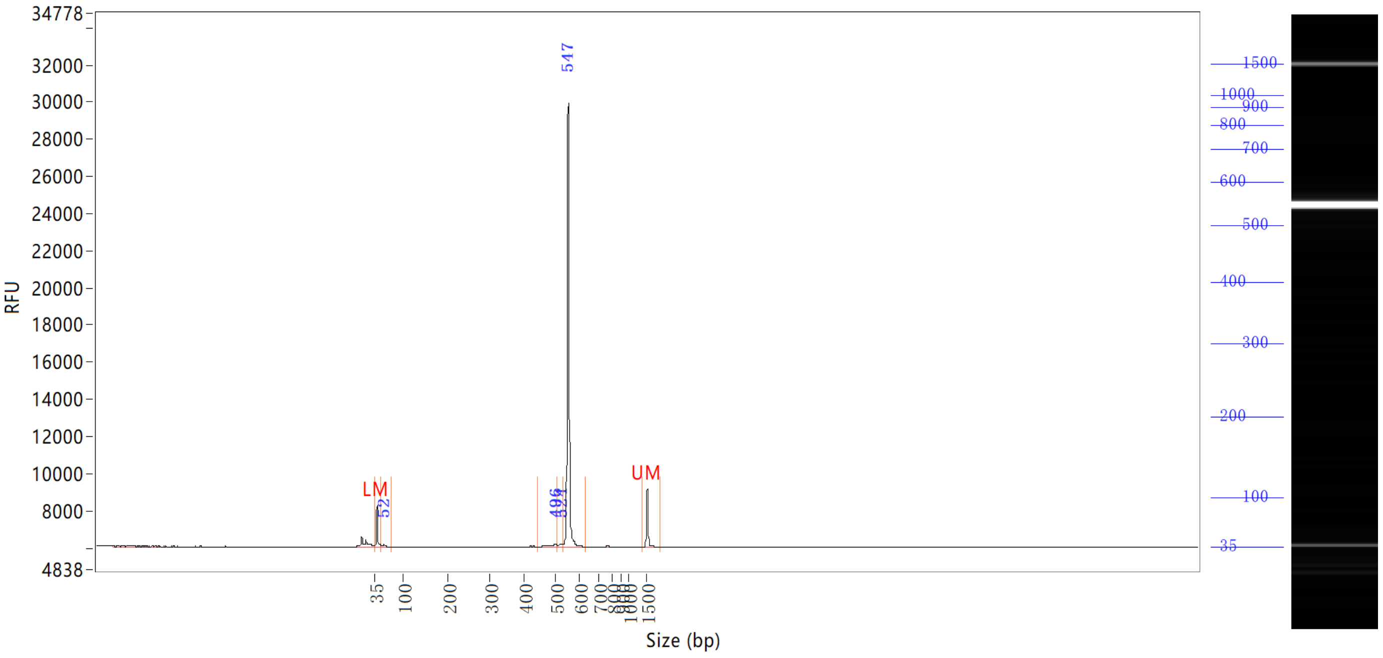

Peak	Size (bp)	Conc. (ng/uL)	From (bp)	To (bp)	Avg. Size (bp)	CV%	RFU	Corr. Peak Area
1	35 (LM)	0.5384	31	45	35	7.07	2215	11.787
2	52	0.8235	45	71	52	8.79	107	1.502
3	496	1.5128	443	504	484	2.82	161	2.760
4	524	0.9328	504	528	517	1.37	165	1.702
5	547	60.9266	528	624	547	1.21	23921	111.148
6	1500 (UM)	0.5000	1367	1882	1500	2.81	3151	10.946

TIC:	64.1957	ng/uL
TIM:	217.3457	nmole/L
Total Conc.:	66.5902	ng/uL

```
Sample Peak Width (sec): 5    Sample Min Peak Height: 50    Sample Baseline V to V?: Y    Sample Baseline V to V pts: 3
Sample Filter: Binomial       # of Pts for Filter: 3        Sample Start Region (min): 0  Sample End Region (min): 75
Marker Peak Width (sec): 5    Marker Min Peak Height: 500   Marker Baseline V to V?: Y    Marker Baseline V to V pts: 3
Lower Marker Selection: First Peak > 500 RFU                Upper Marker Selection: Last Peak > 500 RFU
Ladder Size (bp)35, 100, 200, 300, 400, 500, 600, 700, 800, 900, 1000, 1500
Quantification Using: Upper Marker          Final Concentration (ng/uL): 0.5000          Dilution Factor: 12.0
```

 Copyright 2015 Advanced Analytical Technologies, Inc.

Sample: SampF9
Well Location: F9

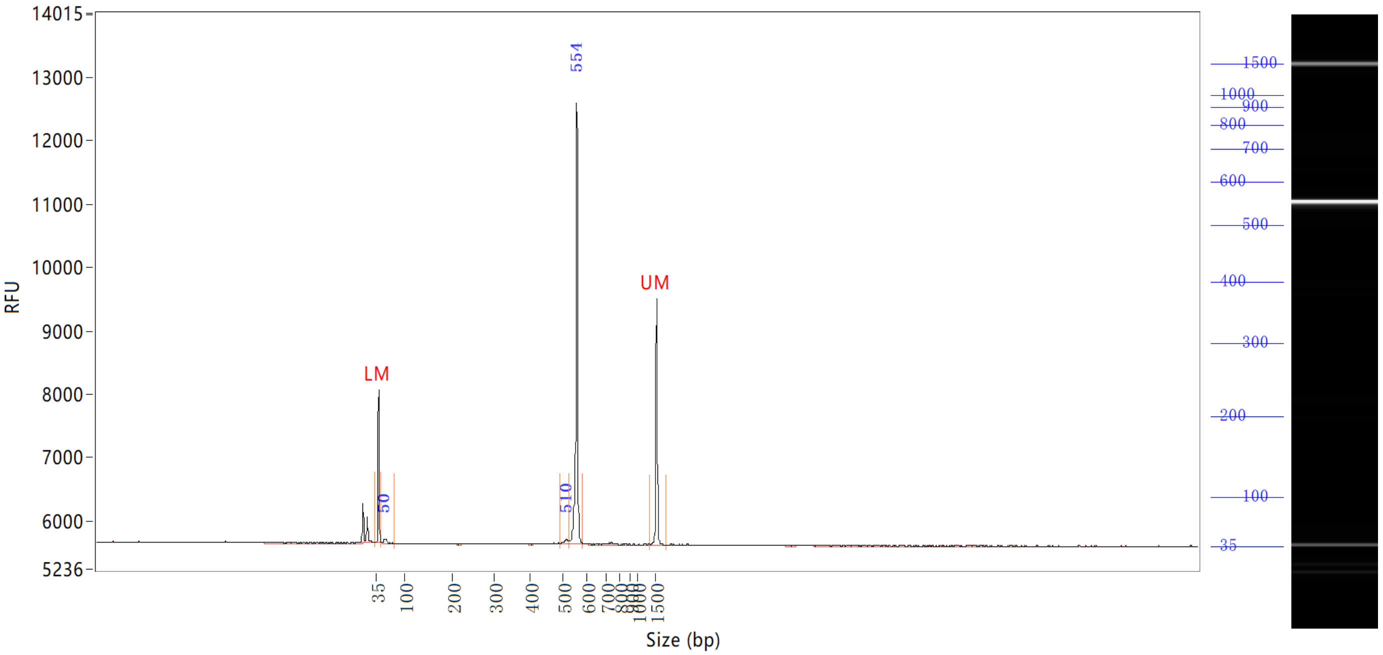

Peak	Size (bp)	Conc. (ng/uL)	From (bp)	To (bp)	Avg. Size (bp)	CV%	RFU	Corr. Peak Area
1	35 (LM)	0.4409	31	44	35	4.17	2412	11.337
2	50	0.4393	44	74	53	11.52	61	0.941
3	510	0.3128	490	522	509	1.49	68	0.670
4	554	14.4278	522	579	551	1.03	6978	30.918
5	1500 (UM)	0.5000	1334	1750	1493	1.85	3877	12.858

TIC:	15.1799	ng/uL
TIM:	57.6812	nmole/L
Total Conc.:	17.0347	ng/uL

```
Sample Peak Width (sec): 5     Sample Min Peak Height: 50    Sample Baseline V to V?: Y    Sample Baseline V to V pts: 3
Sample Filter: Binomial        # of Pts for Filter: 3        Sample Start Region (min): 0  Sample End Region (min): 75
Marker Peak Width (sec): 5     Marker Min Peak Height: 500   Marker Baseline V to V?: Y    Marker Baseline V to V pts: 3
Lower Marker Selection: First Peak > 500 RFU                 Upper Marker Selection: Last Peak > 500 RFU
Ladder Size (bp)35, 100, 200, 300, 400, 500, 600, 700, 800, 900, 1000, 1500
Quantification Using: Upper Marker          Final Concentration (ng/uL): 0.5000          Dilution Factor: 12.0
```

 Copyright 2015 Advanced Analytical Technologies, Inc.

Sample: SampG9
Well Location: G9

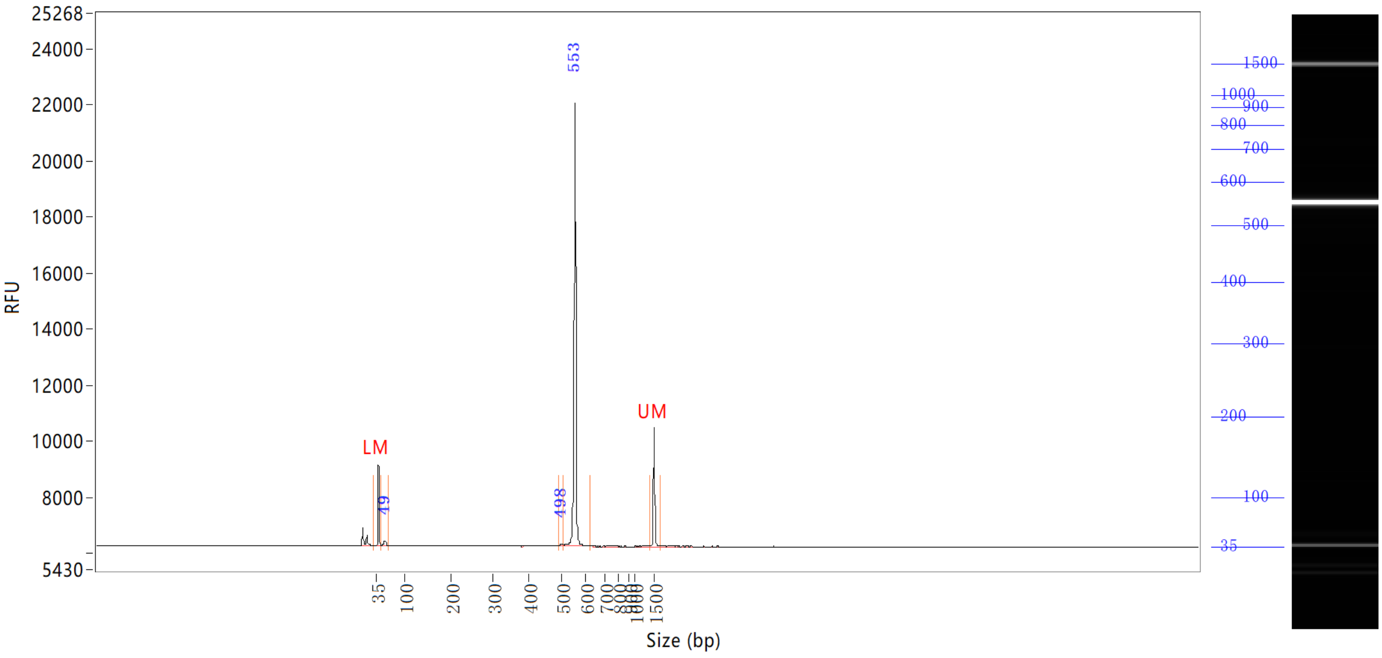

Peak	Size (bp)	Conc. (ng/uL)	From (bp)	To (bp)	Avg. Size (bp)	CV%	RFU	Corr. Peak Area
1	35 (LM)	0.4769	25	44	35	4.72	2863	13.738
2	49	0.8254	44	61	50	5.97	173	1.981
3	498	0.2248	490	508	498	0.97	62	0.540
4	553	29.9096	508	625	551	1.22	15829	71.797
5	1500 (UM)	0.5000	1400	1684	1492	1.80	4233	14.403

TIC:	30.9598	ng/uL
TIM:	117.1053	nmole/L
Total Conc.:	32.9322	ng/uL

Sample Peak Width (sec): 5   Sample Min Peak Height: 50   Sample Baseline V to V?: Y   Sample Baseline V to V pts: 3
Sample Filter: Binomial   # of Pts for Filter: 3   Sample Start Region (min): 0   Sample End Region (min): 75
Marker Peak Width (sec): 5   Marker Min Peak Height: 500   Marker Baseline V to V?: Y   Marker Baseline V to V pts: 3
Lower Marker Selection: First Peak > 500 RFU   Upper Marker Selection: Last Peak > 500 RFU
Ladder Size (bp)35, 100, 200, 300, 400, 500, 600, 700, 800, 900, 1000, 1500
Quantification Using: Upper Marker   Final Concentration (ng/uL): 0.5000   Dilution Factor: 12.0

 Copyright 2015 Advanced Analytical Technologies, Inc.

Sample: SampH9
Well Location: H9

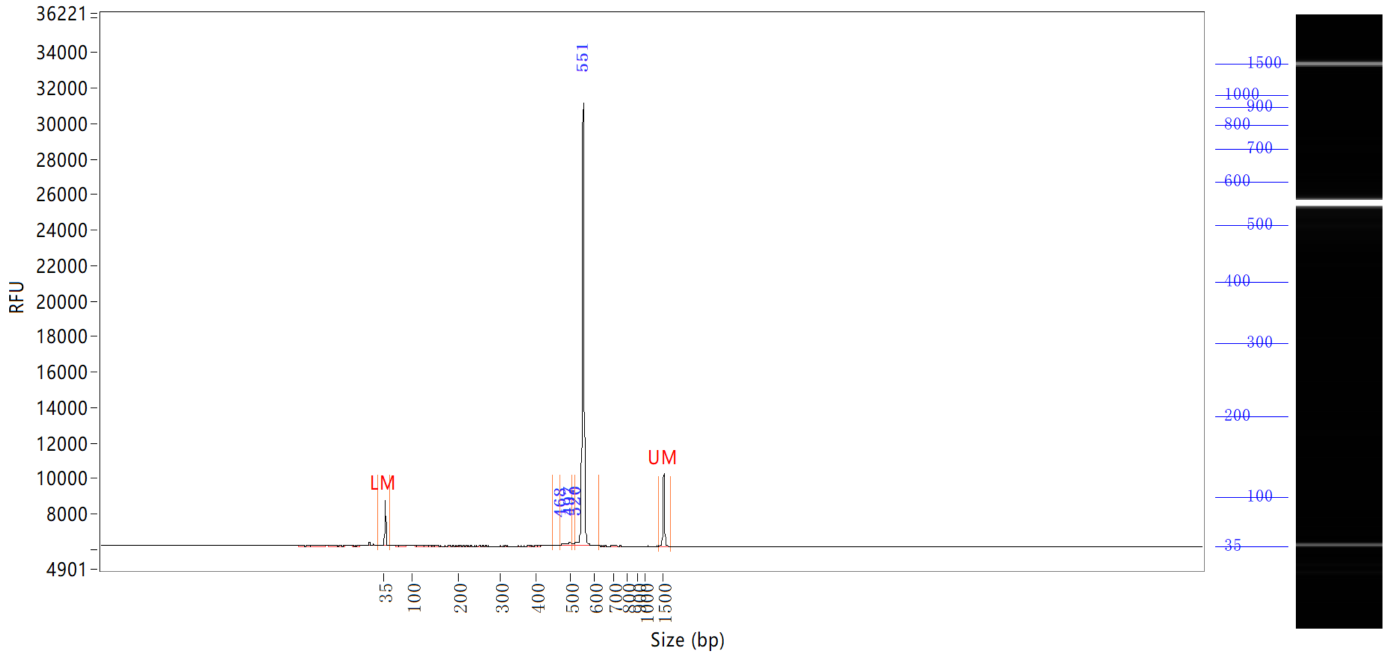

Peak	Size (bp)	Conc. (ng/uL)	From (bp)	To (bp)	Avg. Size (bp)	CV%	RFU	Corr. Peak Area
1	35 (LM)	0.4635	20	47	35	6.35	2597	12.620
2	468	0.1791	449	469	463	1.17	64	0.406
3	497	1.3604	469	508	490	2.11	211	3.087
4	520	0.5738	508	523	516	0.84	199	1.302
5	551	51.3031	523	618	550	1.03	24998	116.410
6	1500 (UM)	0.5000	1367	1684	1494	1.70	4072	13.614

TIC:	53.4165	ng/uL
TIM:	160.5325	nmole/L
Total Conc.:	55.3209	ng/uL

Sample Peak Width (sec): 5 Sample Min Peak Height: 50 Sample Baseline V to V?: Y Sample Baseline V to V pts: 3
Sample Filter: Binomial # of Pts for Filter: 3 Sample Start Region (min): 0 Sample End Region (min): 75
Marker Peak Width (sec): 5 Marker Min Peak Height: 500 Marker Baseline V to V?: Y Marker Baseline V to V pts: 3
Lower Marker Selection: First Peak > 500 RFU Upper Marker Selection: Last Peak > 500 RFU
Ladder Size (bp)35, 100, 200, 300, 400, 500, 600, 700, 800, 900, 1000, 1500
Quantification Using: Upper Marker Final Concentration (ng/uL): 0.5000 Dilution Factor: 12.0

 Copyright 2015 Advanced Analytical Technologies, Inc.

Sample: SampA10
Well Location: A10

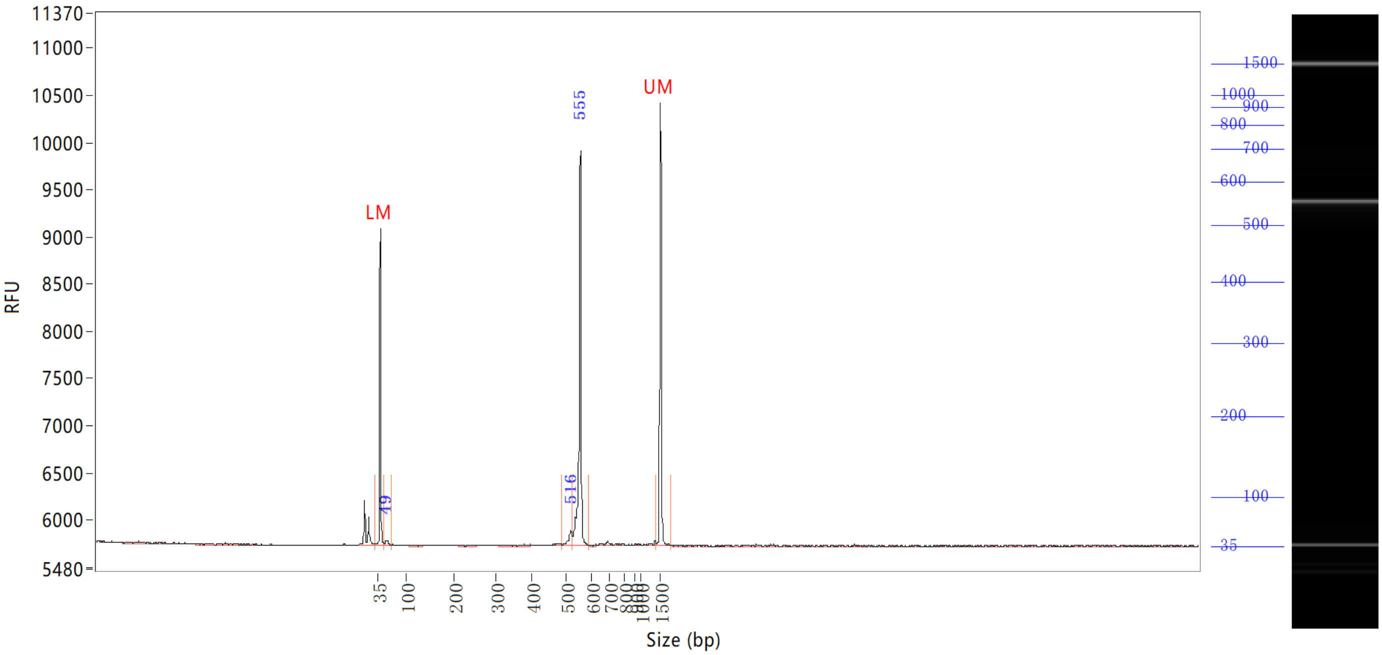

Peak	Size (bp)	Conc. (ng/uL)	From (bp)	To (bp)	Avg. Size (bp)	CV%	RFU	Corr. Peak Area
1	35 (LM)	0.5144	27	45	35	5.21	3365	15.877
2	49	0.3483	45	65	52	8.37	57	0.896
3	516	0.5314	487	525	512	1.70	156	1.367
4	555	7.7434	525	592	551	1.36	4188	19.918
5	1500 (UM)	0.5000	1394	1776	1496	1.87	4696	15.434

TIC:	8.6230	ng/uL
TIM:	35.8054	nmole/L
Total Conc.:	10.2887	ng/uL

Sample Peak Width (sec): 5   Sample Min Peak Height: 50   Sample Baseline V to V?: Y   Sample Baseline V to V pts: 3
Sample Filter: Binomial   # of Pts for Filter: 3   Sample Start Region (min): 0   Sample End Region (min): 75
Marker Peak Width (sec): 5   Marker Min Peak Height: 500   Marker Baseline V to V?: Y   Marker Baseline V to V pts: 3
Lower Marker Selection: First Peak > 500 RFU   Upper Marker Selection: Last Peak > 500 RFU
Ladder Size (bp)35, 100, 200, 300, 400, 500, 600, 700, 800, 900, 1000, 1500
Quantification Using: Upper Marker   Final Concentration (ng/uL): 0.5000   Dilution Factor: 12.0

 Copyright 2015 Advanced Analytical Technologies, Inc.

Sample: SampB10
Well Location: B10

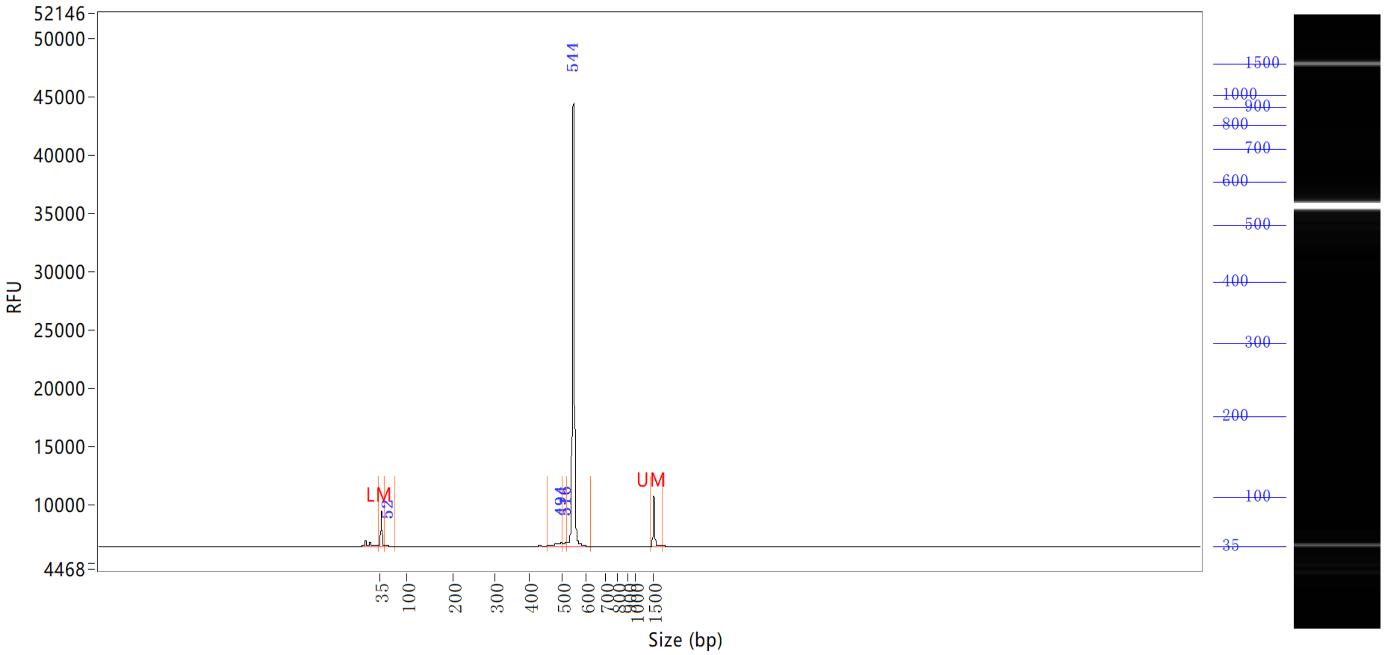

Peak	Size (bp)	Conc. (ng/uL)	From (bp)	To (bp)	Avg. Size (bp)	CV%	RFU	Corr. Peak Area
1	35 (LM)	0.5142	29	44	35	5.95	3101	15.844
2	52	0.5123	44	70	51	8.90	114	1.316
3	494	2.1392	457	504	485	2.51	330	5.494
4	516	0.9478	504	519	512	0.83	372	2.434
5	544	73.8581	519	624	543	1.17	38096	189.670
6	1500 (UM)	0.5000	1420	1744	1503	2.53	4326	15.408

TIC:	77.4575	ng/uL
TIM:	250.5876	nmole/L
Total Conc.:	79.2084	ng/uL

```
Sample Peak Width (sec): 5     Sample Min Peak Height: 50    Sample Baseline V to V?: Y    Sample Baseline V to V pts: 3
Sample Filter: Binomial        # of Pts for Filter: 3        Sample Start Region (min): 0  Sample End Region (min): 75
Marker Peak Width (sec): 5     Marker Min Peak Height: 500   Marker Baseline V to V?: Y    Marker Baseline V to V pts: 3
Lower Marker Selection: First Peak > 500 RFU                 Upper Marker Selection: Last Peak > 500 RFU
Ladder Size (bp)35, 100, 200, 300, 400, 500, 600, 700, 800, 900, 1000, 1500
Quantification Using: Upper Marker          Final Concentration (ng/uL): 0.5000          Dilution Factor: 12.0
```

 Copyright 2015 Advanced Analytical Technologies, Inc.

Sample: SampC10
Well Location: C10

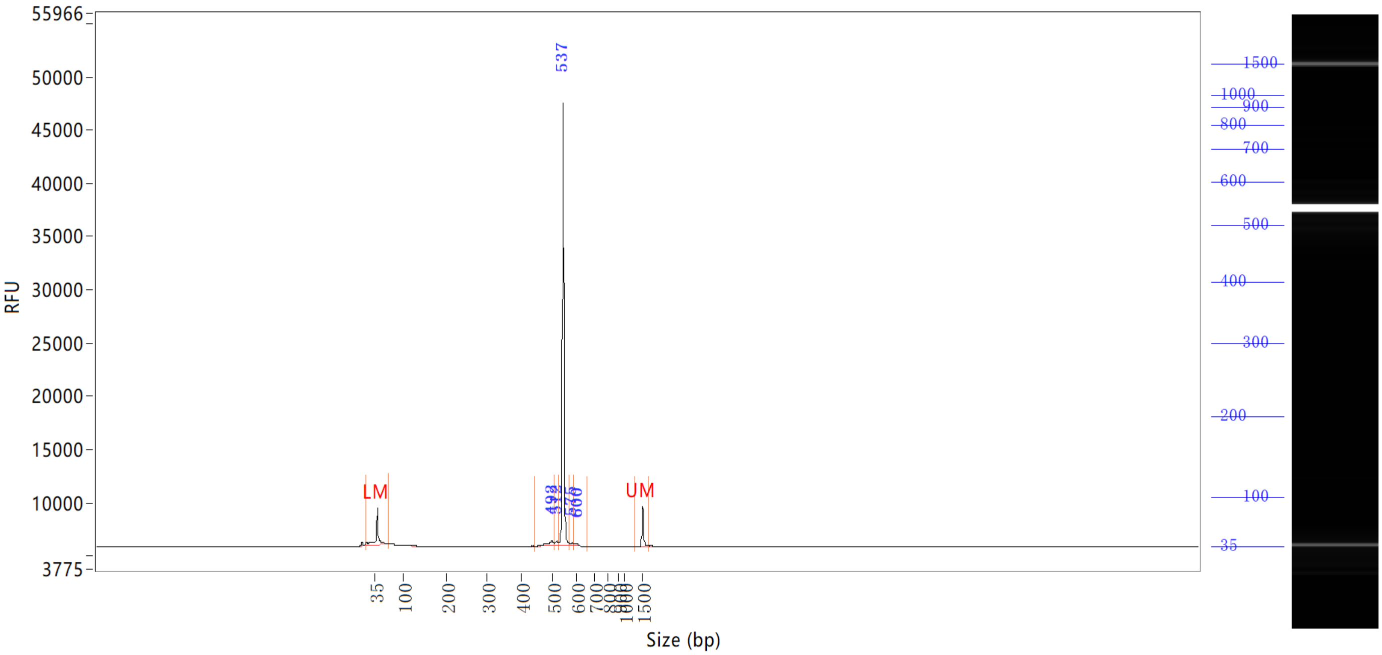

Peak	Size (bp)	Conc. (ng/uL)	From (bp)	To (bp)	Avg. Size (bp)	CV%	RFU	Corr. Peak Area
1	35 (LM)	1.0964	11	63	33	27.36	3432	30.875
2	492	2.4286	442	500	483	2.54	427	5.699
3	512	1.4845	500	522	511	1.18	368	3.484
4	537	81.5947	522	567	537	0.83	41572	191.476
5	575	0.6363	567	586	575	0.90	198	1.493
6	600	0.4600	586	658	598	1.46	154	1.079
7	1500 (UM)	0.5000	1287	1691	1499	2.56	3769	14.080

TIC:	86.6041	ng/uL
TIM:	266.1773	nmole/L
Total Conc.:	88.2302	ng/uL

```
Sample Peak Width (sec): 5    Sample Min Peak Height: 50    Sample Baseline V to V?: Y    Sample Baseline V to V pts: 3
Sample Filter: Binomial       # of Pts for Filter: 3        Sample Start Region (min): 0  Sample End Region (min): 75
Marker Peak Width (sec): 5    Marker Min Peak Height: 500   Marker Baseline V to V?: Y    Marker Baseline V to V pts: 3
Lower Marker Selection: First Peak > 500 RFU                Upper Marker Selection: Last Peak > 500 RFU
Ladder Size (bp)35, 100, 200, 300, 400, 500, 600, 700, 800, 900, 1000, 1500
Quantification Using: Upper Marker          Final Concentration (ng/uL): 0.5000          Dilution Factor: 12.0
```


Copyright 2015 Advanced Analytical Technologies, Inc.

Sample: SampD10
Well Location: D10

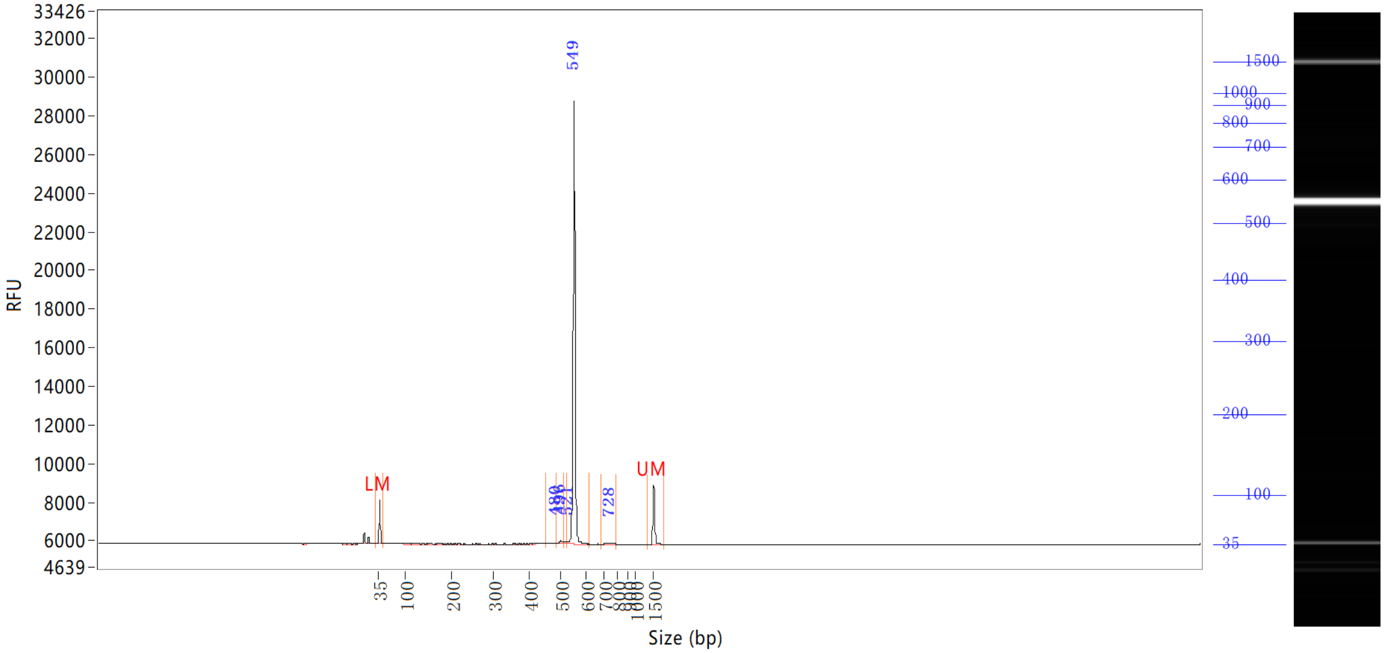

Peak	Size (bp)	Conc. (ng/uL)	From (bp)	To (bp)	Avg. Size (bp)	CV%	RFU	Corr. Peak Area
1	35 (LM)	0.4620	29	46	35	5.34	2293	11.155
2	480	0.3895	451	482	472	1.57	65	0.784
3	496	0.8903	482	510	496	1.40	168	1.791
4	521	0.4444	510	525	518	0.83	137	0.894
5	549	52.9456	525	616	548	1.00	22982	106.529
6	728	0.4781	681	782	727	3.16	65	0.962
7	1500 (UM)	0.5000	1354	1783	1500	2.58	3098	12.072

TIC:	55.1479	ng/uL
TIM:	165.7952	nmole/L
Total Conc.:	57.1717	ng/uL

```
Sample Peak Width (sec): 5    Sample Min Peak Height: 50    Sample Baseline V to V?: Y    Sample Baseline V to V pts: 3
Sample Filter: Binomial       # of Pts for Filter: 3        Sample Start Region (min): 0  Sample End Region (min): 75
Marker Peak Width (sec): 5    Marker Min Peak Height: 500   Marker Baseline V to V?: Y    Marker Baseline V to V pts: 3
Lower Marker Selection: First Peak > 500 RFU                Upper Marker Selection: Last Peak > 500 RFU
Ladder Size (bp)35, 100, 200, 300, 400, 500, 600, 700, 800, 900, 1000, 1500
Quantification Using: Upper Marker          Final Concentration (ng/uL): 0.5000          Dilution Factor: 12.0
```

 Copyright 2015 Advanced Analytical Technologies, Inc.

Sample: SampE10
Well Location: E10

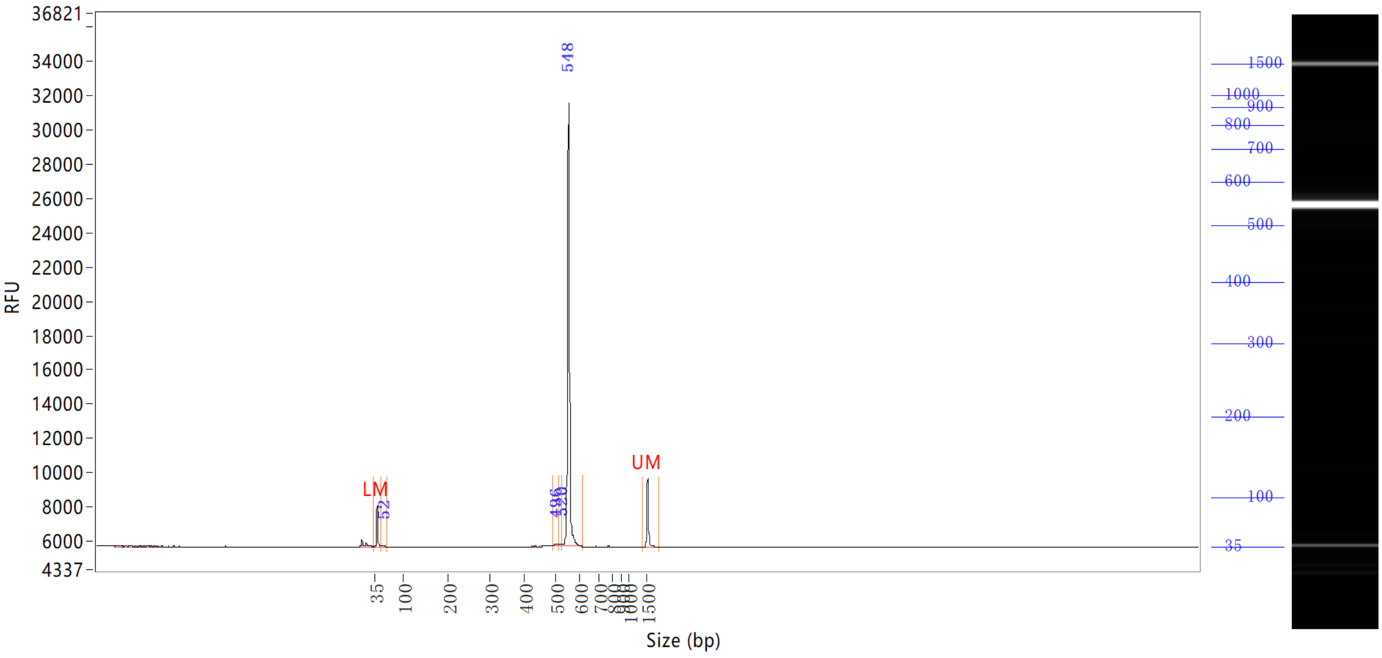

Peak	Size (bp)	Conc. (ng/uL)	From (bp)	To (bp)	Avg. Size (bp)	CV%	RFU	Corr. Peak Area
1	35 (LM)	0.4294	29	47	35	6.74	2446	12.008
2	52	0.3055	47	60	51	5.19	70	0.712
3	496	0.3476	487	510	498	1.20	101	0.810
4	520	0.3065	510	524	518	0.75	130	0.714
5	548	53.5075	524	611	547	1.18	25909	124.706
6	1500 (UM)	0.5000	1354	1842	1501	2.51	3968	13.984

TIC:	54.4671	ng/uL
TIM:	172.9046	nmole/L
Total Conc.:	56.3922	ng/uL

```
Sample Peak Width (sec): 5    Sample Min Peak Height: 50    Sample Baseline V to V?: Y    Sample Baseline V to V pts: 3
Sample Filter: Binomial       # of Pts for Filter: 3        Sample Start Region (min): 0  Sample End Region (min): 75
Marker Peak Width (sec): 5    Marker Min Peak Height: 500   Marker Baseline V to V?: Y    Marker Baseline V to V pts: 3
Lower Marker Selection: First Peak > 500 RFU                Upper Marker Selection: Last Peak > 500 RFU
Ladder Size (bp)35, 100, 200, 300, 400, 500, 600, 700, 800, 900, 1000, 1500
Quantification Using: Upper Marker          Final Concentration (ng/uL): 0.5000          Dilution Factor: 12.0
```

 Copyright 2015 Advanced Analytical Technologies, Inc.

Sample: SampF10
Well Location: F10

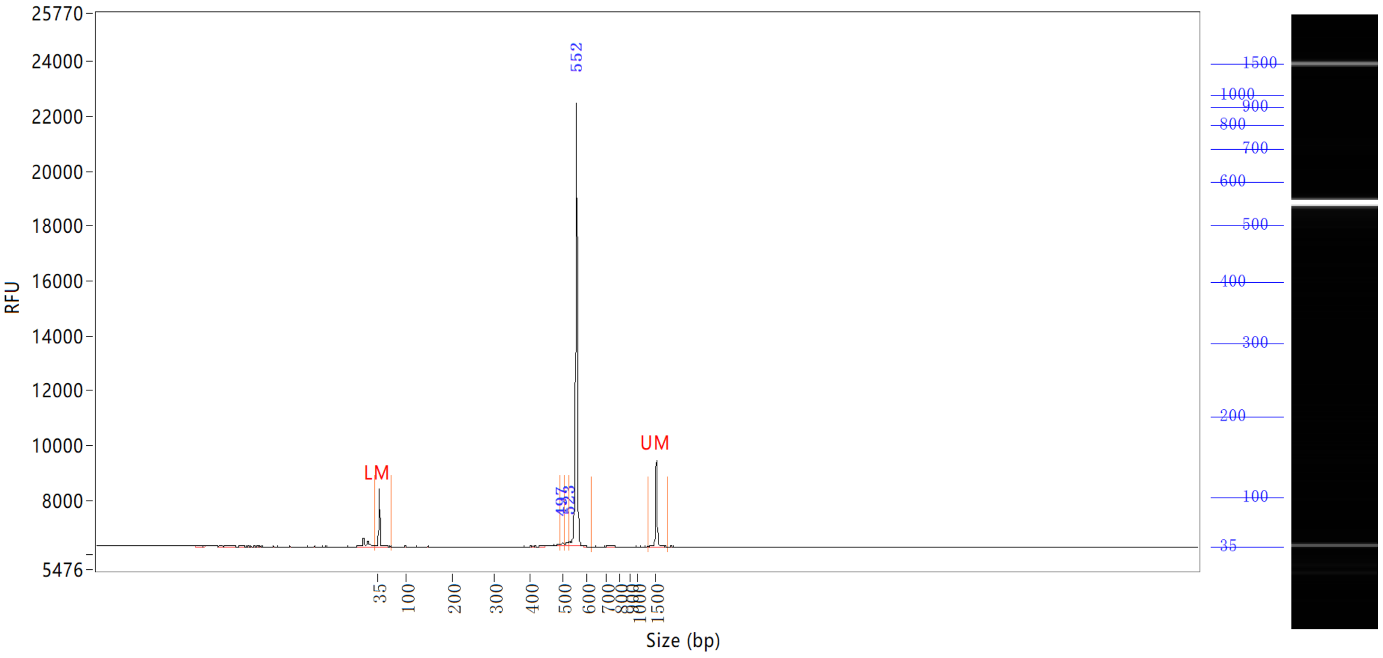

Peak	Size (bp)	Conc. (ng/uL)	From (bp)	To (bp)	Avg. Size (bp)	CV%	RFU	Corr. Peak Area
1	35 (LM)	0.5049	28	65	35	10.40	2116	10.875
2	497	0.3589	490	503	497	0.69	101	0.644
3	523	0.6692	503	526	516	1.28	147	1.201
4	552	41.9352	526	621	551	0.93	16190	75.273
5	1500 (UM)	0.5000	1300	1816	1497	2.40	3130	10.770

TIC:	42.9632	ng/uL
TIM:	128.5634	nmole/L
Total Conc.:	45.4773	ng/uL

```
Sample Peak Width (sec): 5    Sample Min Peak Height: 50    Sample Baseline V to V?: Y    Sample Baseline V to V pts: 3
Sample Filter: Binomial       # of Pts for Filter: 3        Sample Start Region (min): 0  Sample End Region (min): 75
Marker Peak Width (sec): 5    Marker Min Peak Height: 500   Marker Baseline V to V?: Y    Marker Baseline V to V pts: 3
Lower Marker Selection: First Peak > 500 RFU                Upper Marker Selection: Last Peak > 500 RFU
Ladder Size (bp)35, 100, 200, 300, 400, 500, 600, 700, 800, 900, 1000, 1500
Quantification Using: Upper Marker          Final Concentration (ng/uL): 0.5000          Dilution Factor: 12.0
```

 Copyright 2015 Advanced Analytical Technologies, Inc.

Sample: SampG10
Well Location: G10

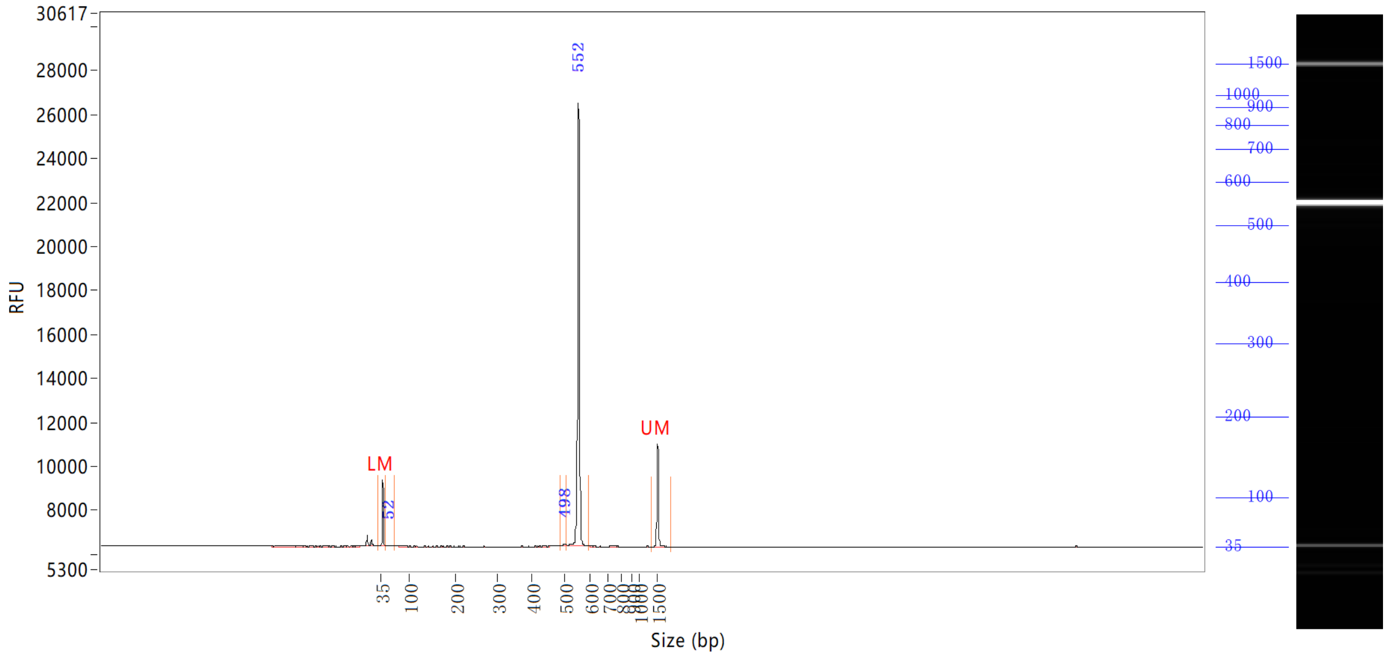

Peak	Size (bp)	Conc. (ng/uL)	From (bp)	To (bp)	Avg. Size (bp)	CV%	RFU	Corr. Peak Area
1	35 (LM)	0.4355	27	44	35	4.70	3003	14.122
2	52	0.3150	44	66	52	8.98	56	0.851
3	498	0.3497	485	507	496	1.15	100	0.945
4	552	33.1988	507	597	550	1.04	20207	89.711
5	1500 (UM)	0.5000	1360	1855	1497	2.46	4698	16.213

TIC:	33.8634	ng/uL
TIM:	110.4122	nmole/L
Total Conc.:	35.4597	ng/uL

```
Sample Peak Width (sec): 5    Sample Min Peak Height: 50    Sample Baseline V to V?: Y    Sample Baseline V to V pts: 3
Sample Filter: Binomial       # of Pts for Filter: 3        Sample Start Region (min): 0  Sample End Region (min): 75
Marker Peak Width (sec): 5    Marker Min Peak Height: 500   Marker Baseline V to V?: Y    Marker Baseline V to V pts: 3
Lower Marker Selection: First Peak > 500 RFU                Upper Marker Selection: Last Peak > 500 RFU
Ladder Size (bp)35, 100, 200, 300, 400, 500, 600, 700, 800, 900, 1000, 1500
Quantification Using: Upper Marker          Final Concentration (ng/uL): 0.5000          Dilution Factor: 12.0
```

 Copyright 2015 Advanced Analytical Technologies, Inc.

Sample: SampH10
Well Location: H10

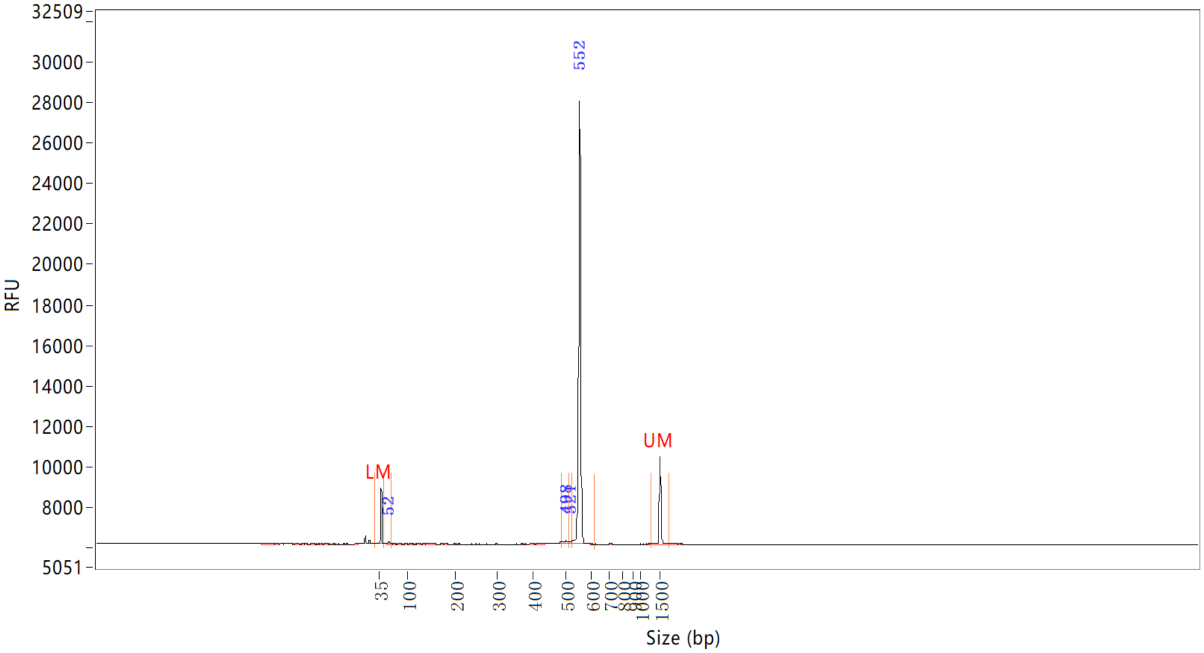

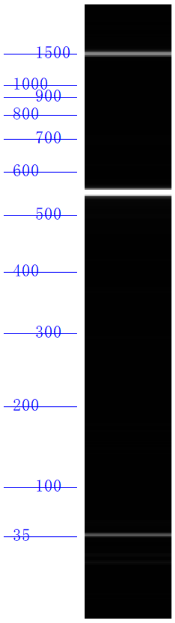

Peak	Size (bp)	Conc. (ng/uL)	From (bp)	To (bp)	Avg. Size (bp)	CV%	RFU	Corr. Peak Area
1	35 (LM)	0.4265	23	42	35	5.06	2770	13.121
2	52	0.4260	42	62	50	7.94	100	1.092
3	498	0.3848	487	510	498	1.14	108	0.986
4	521	0.2293	510	522	517	0.68	129	0.588
5	552	40.3650	522	616	551	0.92	21902	103.489
6	1500 (UM)	0.5000	1274	1763	1496	2.35	4341	15.383

TIC:	41.4050	ng/uL
TIM:	136.5078	nmole/L
Total Conc.:	43.3011	ng/uL

```
Sample Peak Width (sec): 5    Sample Min Peak Height: 50    Sample Baseline V to V?: Y    Sample Baseline V to V pts: 3
Sample Filter: Binomial       # of Pts for Filter: 3        Sample Start Region (min): 0  Sample End Region (min): 75
Marker Peak Width (sec): 5    Marker Min Peak Height: 500   Marker Baseline V to V?: Y    Marker Baseline V to V pts: 3
Lower Marker Selection: First Peak > 500 RFU                Upper Marker Selection: Last Peak > 500 RFU
Ladder Size (bp)35, 100, 200, 300, 400, 500, 600, 700, 800, 900, 1000, 1500
Quantification Using: Upper Marker          Final Concentration (ng/uL): 0.5000          Dilution Factor: 12.0
```

 Copyright 2015 Advanced Analytical Technologies, Inc.

Sample: SampA11
Well Location: A11

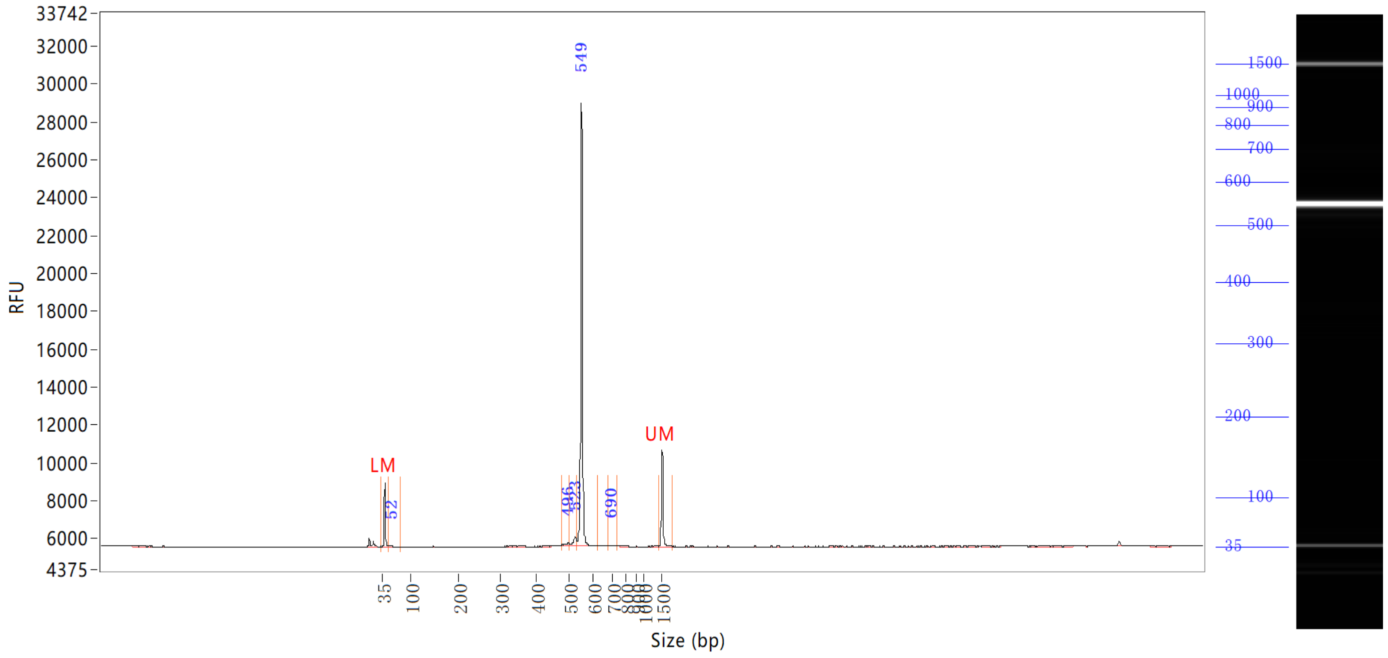

Peak	Size (bp)	Conc. (ng/uL)	From (bp)	To (bp)	Avg. Size (bp)	CV%	RFU	Corr. Peak Area
1	35 (LM)	0.4576	28	44	34	4.82	3345	15.841
2	52	0.3206	44	72	53	11.20	78	0.925
3	496	0.3386	480	504	494	1.14	123	0.977
4	523	1.1748	504	530	520	1.18	481	3.389
5	549	36.5942	530	624	548	0.93	23413	105.574
6	690	0.2561	673	730	694	1.86	67	0.739
7	1500 (UM)	0.5000	1400	1763	1495	1.98	5067	17.310

TIC:	38.6842	ng/uL
TIM:	125.2522	nmole/L
Total Conc.:	40.2432	ng/uL

```
Sample Peak Width (sec): 5     Sample Min Peak Height: 50    Sample Baseline V to V?: Y    Sample Baseline V to V pts: 3
Sample Filter: Binomial        # of Pts for Filter: 3        Sample Start Region (min): 0  Sample End Region (min): 75
Marker Peak Width (sec): 5     Marker Min Peak Height: 500   Marker Baseline V to V?: Y    Marker Baseline V to V pts: 3
Lower Marker Selection: First Peak > 500 RFU                 Upper Marker Selection: Last Peak > 500 RFU
Ladder Size (bp)35, 100, 200, 300, 400, 500, 600, 700, 800, 900, 1000, 1500
Quantification Using: Upper Marker          Final Concentration (ng/uL): 0.5000          Dilution Factor: 12.0
```

 Copyright 2015 Advanced Analytical Technologies, Inc.

Sample: SampB11
Well Location: B11

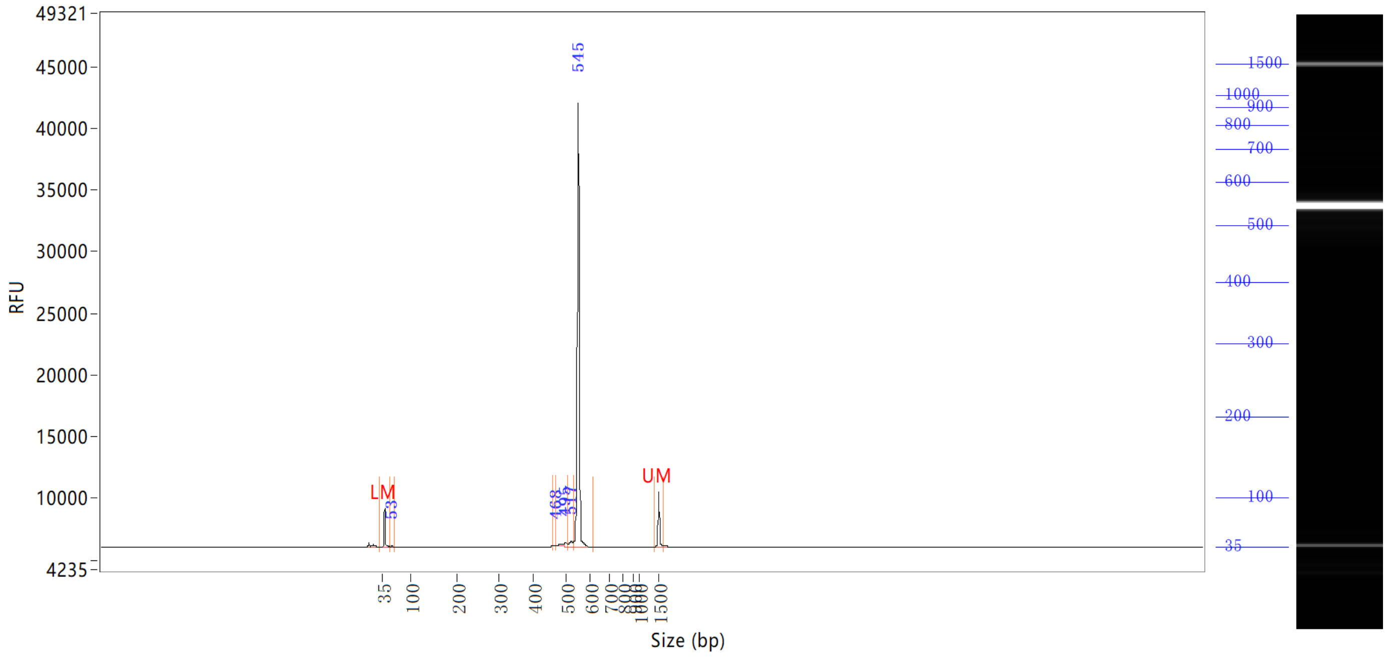

Peak	Size (bp)	Conc. (ng/uL)	From (bp)	To (bp)	Avg. Size (bp)	CV%	RFU	Corr. Peak Area
1	35 (LM)	0.5052	24	49	35	7.99	3137	15.672
2	53	0.2718	49	62	52	4.87	85	0.703
3	468	0.1360	457	468	465	0.55	90	0.352
4	495	1.6982	468	504	489	1.94	326	4.390
5	517	1.4270	504	526	517	1.22	382	3.689
6	545	67.2987	526	610	544	0.99	36014	173.962
7	1500 (UM)	0.5000	1374	1651	1493	1.87	4469	15.510

TIC:	70.8318	ng/uL
TIM:	222.8766	nmole/L
Total Conc.:	72.3118	ng/uL

```
Sample Peak Width (sec): 5    Sample Min Peak Height: 50    Sample Baseline V to V?: Y    Sample Baseline V to V pts: 3
Sample Filter: Binomial       # of Pts for Filter: 3        Sample Start Region (min): 0  Sample End Region (min): 75
Marker Peak Width (sec): 5    Marker Min Peak Height: 500   Marker Baseline V to V?: Y    Marker Baseline V to V pts: 3
Lower Marker Selection: First Peak > 500 RFU                Upper Marker Selection: Last Peak > 500 RFU
Ladder Size (bp)35, 100, 200, 300, 400, 500, 600, 700, 800, 900, 1000, 1500
Quantification Using: Upper Marker          Final Concentration (ng/uL): 0.5000          Dilution Factor: 12.0
```

 Copyright 2015 Advanced Analytical Technologies, Inc.

Sample: SampC11
Well Location: C11

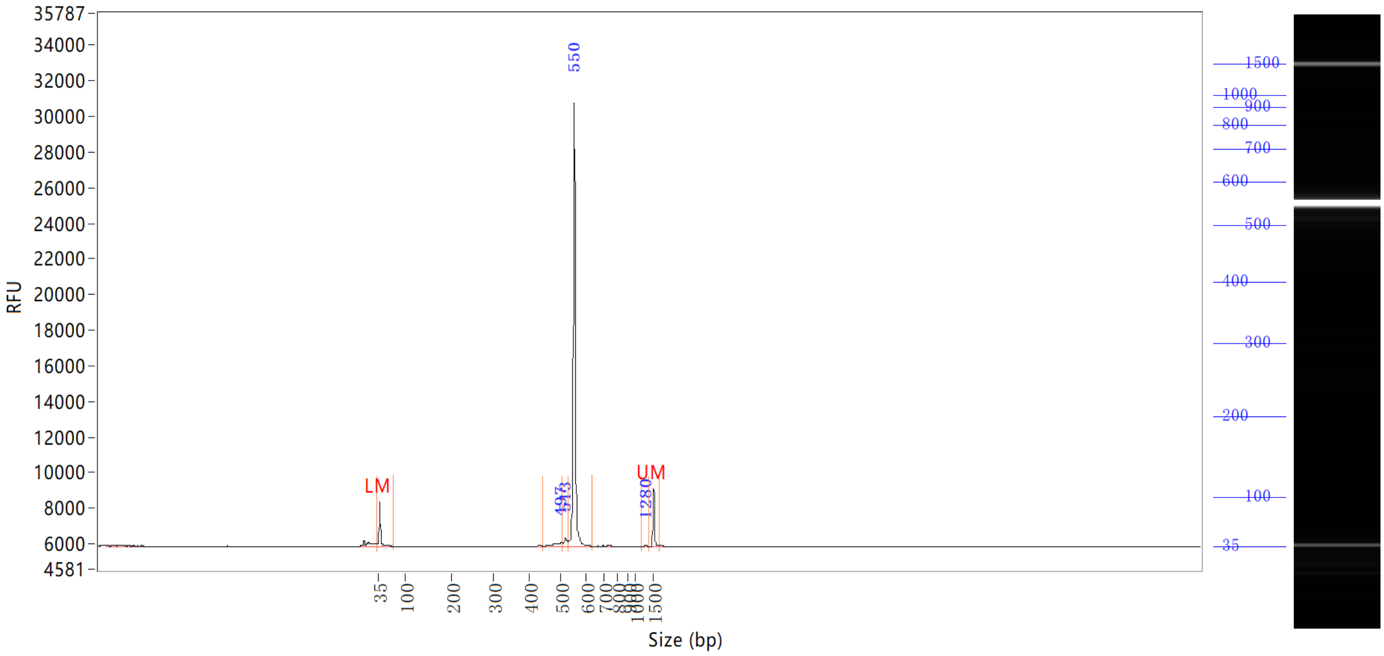

Peak	Size (bp)	Conc. (ng/uL)	From (bp)	To (bp)	Avg. Size (bp)	CV%	RFU	Corr. Peak Area
1	35 (LM)	0.6700	29	68	36	13.19	2491	14.907
2	497	2.2064	444	504	484	2.83	237	4.091
3	513	2.0541	504	525	514	1.07	478	3.809
4	550	67.7015	525	630	549	1.34	24926	125.537
5	1280	0.1622	1174	1367	1271	3.42	50	0.301
6	1500 (UM)	0.5000	1367	1678	1494	2.02	3266	11.126

TIC:	72.1243	ng/uL
TIM:	217.2173	nmole/L
Total Conc.:	74.1132	ng/uL

```
Sample Peak Width (sec): 5     Sample Min Peak Height: 50    Sample Baseline V to V?: Y    Sample Baseline V to V pts: 3
Sample Filter: Binomial        # of Pts for Filter: 3        Sample Start Region (min): 0  Sample End Region (min): 75
Marker Peak Width (sec): 5     Marker Min Peak Height: 500   Marker Baseline V to V?: Y    Marker Baseline V to V pts: 3
Lower Marker Selection: First Peak > 500 RFU                 Upper Marker Selection: Last Peak > 500 RFU
Ladder Size (bp)35, 100, 200, 300, 400, 500, 600, 700, 800, 900, 1000, 1500
Quantification Using: Upper Marker          Final Concentration (ng/uL): 0.5000          Dilution Factor: 12.0
```

 Copyright 2015 Advanced Analytical Technologies, Inc.

Sample: SampD11
Well Location: D11

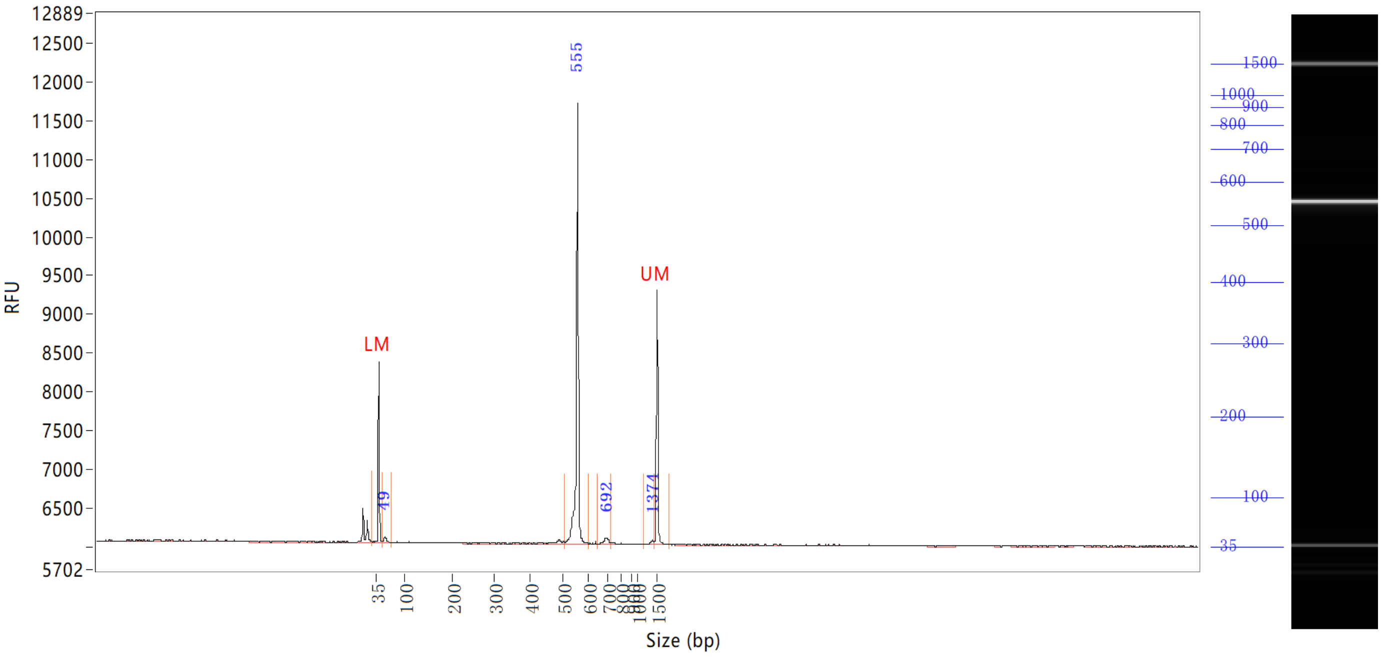

Peak	Size (bp)	Conc. (ng/uL)	From (bp)	To (bp)	Avg. Size (bp)	CV%	RFU	Corr. Peak Area
1	35 (LM)	0.5049	21	45	35	5.63	2331	11.107
2	49	0.4650	45	68	51	9.32	67	0.852
3	555	15.3399	504	604	551	1.71	5704	28.120
4	692	0.4708	649	723	689	1.89	77	0.863
5	1374	0.2514	1147	1420	1334	4.91	65	0.461
6	1500 (UM)	0.5000	1420	1823	1498	1.86	3297	10.999

TIC:	16.5270	ng/uL
TIM:	62.1817	nmole/L
Total Conc.:	18.7351	ng/uL

Sample Peak Width (sec): 5 Sample Min Peak Height: 50 Sample Baseline V to V?: Y Sample Baseline V to V pts: 3
Sample Filter: Binomial # of Pts for Filter: 3 Sample Start Region (min): 0 Sample End Region (min): 75
Marker Peak Width (sec): 5 Marker Min Peak Height: 500 Marker Baseline V to V?: Y Marker Baseline V to V pts: 3
Lower Marker Selection: First Peak > 500 RFU Upper Marker Selection: Last Peak > 500 RFU
Ladder Size (bp)35, 100, 200, 300, 400, 500, 600, 700, 800, 900, 1000, 1500
Quantification Using: Upper Marker Final Concentration (ng/uL): 0.5000 Dilution Factor: 12.0

Sample: SampE11
Well Location: E11

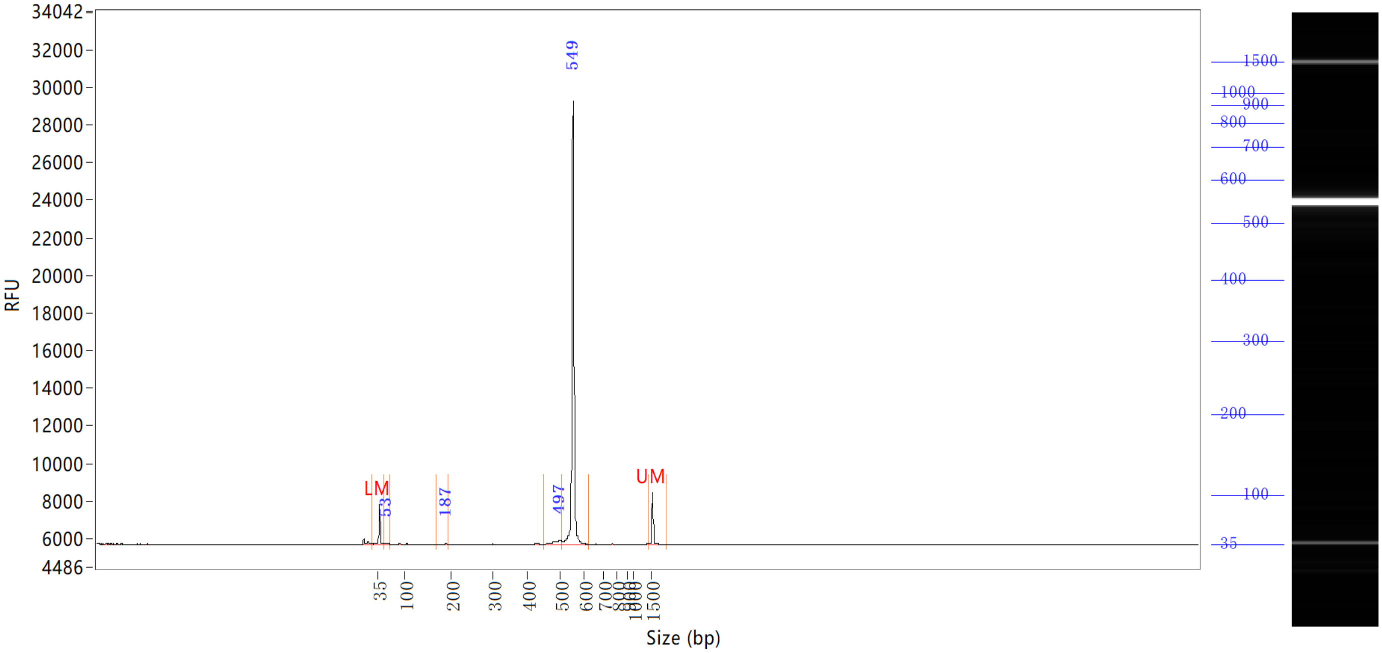

Peak	Size (bp)	Conc. (ng/uL)	From (bp)	To (bp)	Avg. Size (bp)	CV%	RFU	Corr. Peak Area
1	35 (LM)	0.5772	20	47	35	9.29	2105	11.407
2	53	0.6046	47	62	53	6.58	66	0.996
3	187	0.3930	167	193	183	3.08	55	0.647
4	497	2.5018	447	508	486	2.80	235	4.120
5	549	71.9253	508	623	548	1.42	23615	118.447
6	1500 (UM)	0.5000	1414	1908	1504	3.69	2777	9.881

TIC:	75.4247	ng/uL
TIM:	246.6748	nmole/L
Total Conc.:	78.5051	ng/uL

```
Sample Peak Width (sec): 5    Sample Min Peak Height: 50    Sample Baseline V to V?: Y    Sample Baseline V to V pts: 3
Sample Filter: Binomial       # of Pts for Filter: 3        Sample Start Region (min): 0  Sample End Region (min): 75
Marker Peak Width (sec): 5    Marker Min Peak Height: 500   Marker Baseline V to V?: Y    Marker Baseline V to V pts: 3
Lower Marker Selection: First Peak > 500 RFU                Upper Marker Selection: Last Peak > 500 RFU
Ladder Size (bp)35, 100, 200, 300, 400, 500, 600, 700, 800, 900, 1000, 1500
Quantification Using: Upper Marker         Final Concentration (ng/uL): 0.5000         Dilution Factor: 12.0
```

 Copyright 2015 Advanced Analytical Technologies, Inc.

Sample: SampF11
Well Location: F11

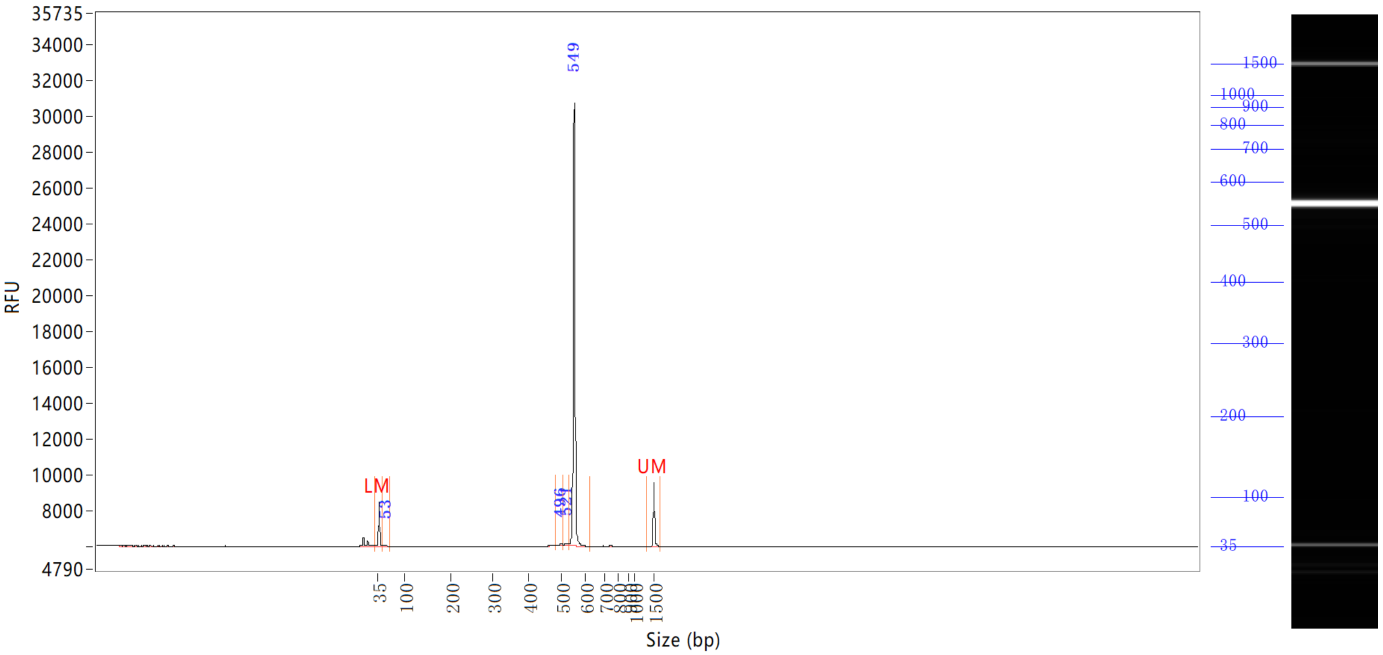

Peak	Size (bp)	Conc. (ng/uL)	From (bp)	To (bp)	Avg. Size (bp)	CV%	RFU	Corr. Peak Area
1	35 (LM)	0.5065	28	44	35	5.44	2461	12.236
2	53	0.3929	44	62	50	6.67	71	0.791
3	496	0.4254	484	506	495	1.07	107	0.856
4	521	0.7259	506	531	521	1.29	145	1.461
5	549	54.0666	531	623	549	0.95	24699	108.845
6	1500 (UM)	0.5000	1327	1697	1497	1.94	3570	12.079

TIC:	55.6108	ng/uL
TIM:	178.6378	nmole/L
Total Conc.:	57.7244	ng/uL

```
Sample Peak Width (sec): 5    Sample Min Peak Height: 50    Sample Baseline V to V?: Y    Sample Baseline V to V pts: 3
Sample Filter: Binomial       # of Pts for Filter: 3        Sample Start Region (min): 0  Sample End Region (min): 75
Marker Peak Width (sec): 5    Marker Min Peak Height: 500   Marker Baseline V to V?: Y    Marker Baseline V to V pts: 3
Lower Marker Selection: First Peak > 500 RFU                Upper Marker Selection: Last Peak > 500 RFU
Ladder Size (bp)35, 100, 200, 300, 400, 500, 600, 700, 800, 900, 1000, 1500
Quantification Using: Upper Marker          Final Concentration (ng/uL): 0.5000          Dilution Factor: 12.0
```

 Copyright 2015 Advanced Analytical Technologies, Inc.

Sample: SampG11
Well Location: G11

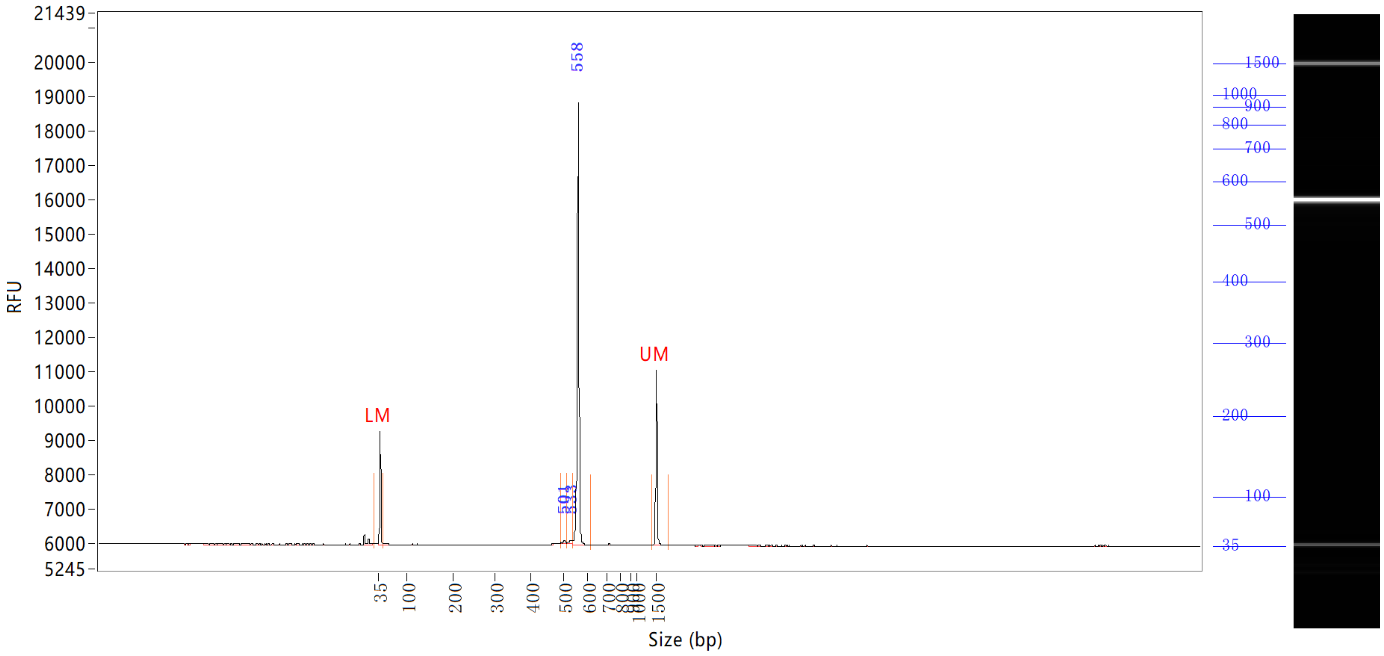

Peak	Size (bp)	Conc. (ng/uL)	From (bp)	To (bp)	Avg. Size (bp)	CV%	RFU	Corr. Peak Area
1	35 (LM)	0.4534	24	44	35	4.54	3308	15.388
2	501	0.2084	493	512	501	1.02	76	0.589
3	533	0.3676	512	536	526	1.27	108	1.040
4	558	20.8538	536	616	556	0.86	12893	58.987
5	1500 (UM)	0.5000	1380	1823	1497	1.82	5119	16.971

TIC:	21.4298	ng/uL
TIM:	63.5554	nmole/L
Total Conc.:	23.1379	ng/uL

```
Sample Peak Width (sec): 5     Sample Min Peak Height: 50    Sample Baseline V to V?: Y    Sample Baseline V to V pts: 3
Sample Filter: Binomial        # of Pts for Filter: 3        Sample Start Region (min): 0  Sample End Region (min): 75
Marker Peak Width (sec): 5     Marker Min Peak Height: 500   Marker Baseline V to V?: Y    Marker Baseline V to V pts: 3
Lower Marker Selection: First Peak > 500 RFU                 Upper Marker Selection: Last Peak > 500 RFU
Ladder Size (bp)35, 100, 200, 300, 400, 500, 600, 700, 800, 900, 1000, 1500
Quantification Using: Upper Marker          Final Concentration (ng/uL): 0.5000          Dilution Factor: 12.0
```

 Copyright 2015 Advanced Analytical Technologies, Inc.

Sample: SampH11
Well Location: H11

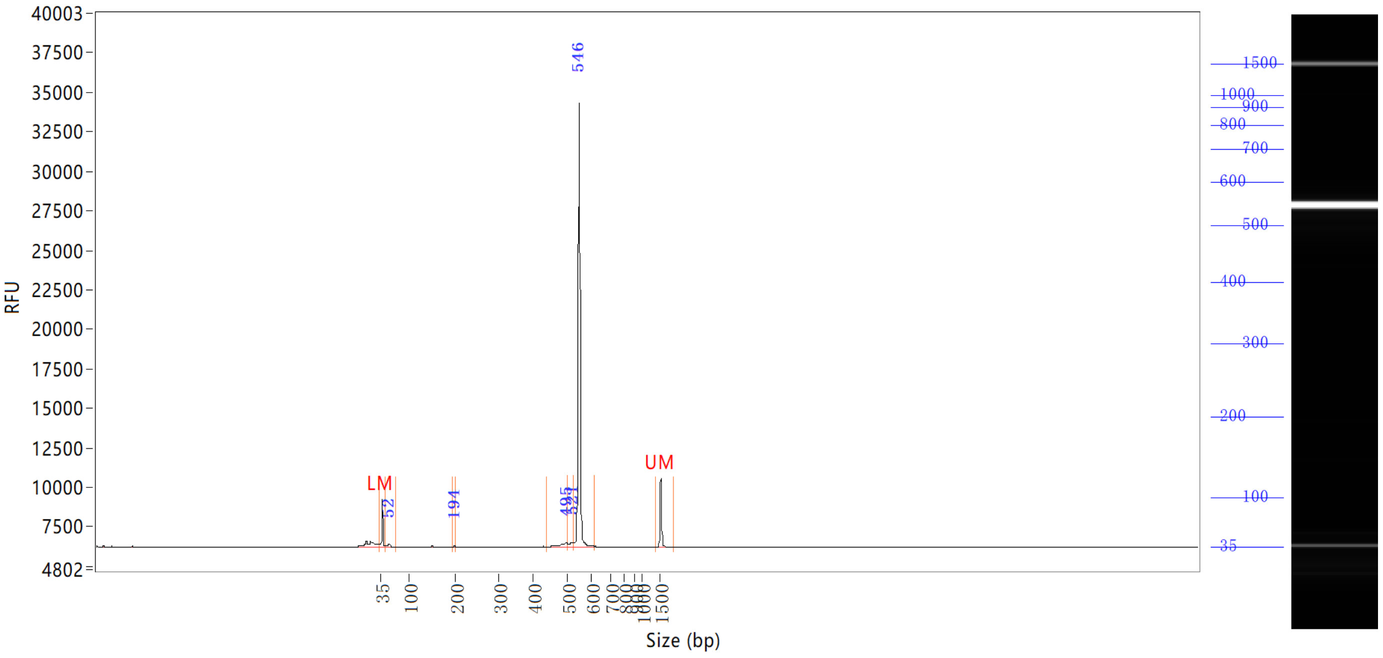

Peak	Size (bp)	Conc. (ng/uL)	From (bp)	To (bp)	Avg. Size (bp)	CV%	RFU	Corr. Peak Area
1	35 (LM)	0.5367	29	44	35	6.65	3006	15.879
2	52	0.9569	44	67	50	7.98	197	2.359
3	194	0.1232	191	199	194	0.87	55	0.304
4	495	1.6443	440	504	484	2.73	224	4.054
5	521	1.2489	504	528	517	1.32	285	3.079
6	546	53.8021	528	614	546	1.02	28109	132.639
7	1500 (UM)	0.5000	1394	1862	1500	2.53	4366	14.792

TIC:	57.7755	ng/uL
TIM:	204.1085	nmole/L
Total Conc.:	59.2368	ng/uL

```
Sample Peak Width (sec): 5     Sample Min Peak Height: 50    Sample Baseline V to V?: Y    Sample Baseline V to V pts: 3
Sample Filter: Binomial        # of Pts for Filter: 3        Sample Start Region (min): 0  Sample End Region (min): 75
Marker Peak Width (sec): 5     Marker Min Peak Height: 500   Marker Baseline V to V?: Y    Marker Baseline V to V pts: 3
Lower Marker Selection: First Peak > 500 RFU                 Upper Marker Selection: Last Peak > 500 RFU
Ladder Size (bp)35, 100, 200, 300, 400, 500, 600, 700, 800, 900, 1000, 1500
Quantification Using: Upper Marker          Final Concentration (ng/uL): 0.5000          Dilution Factor: 12.0
```

 Copyright 2015 Advanced Analytical Technologies, Inc.

Sample: SampA12
Well Location: A12

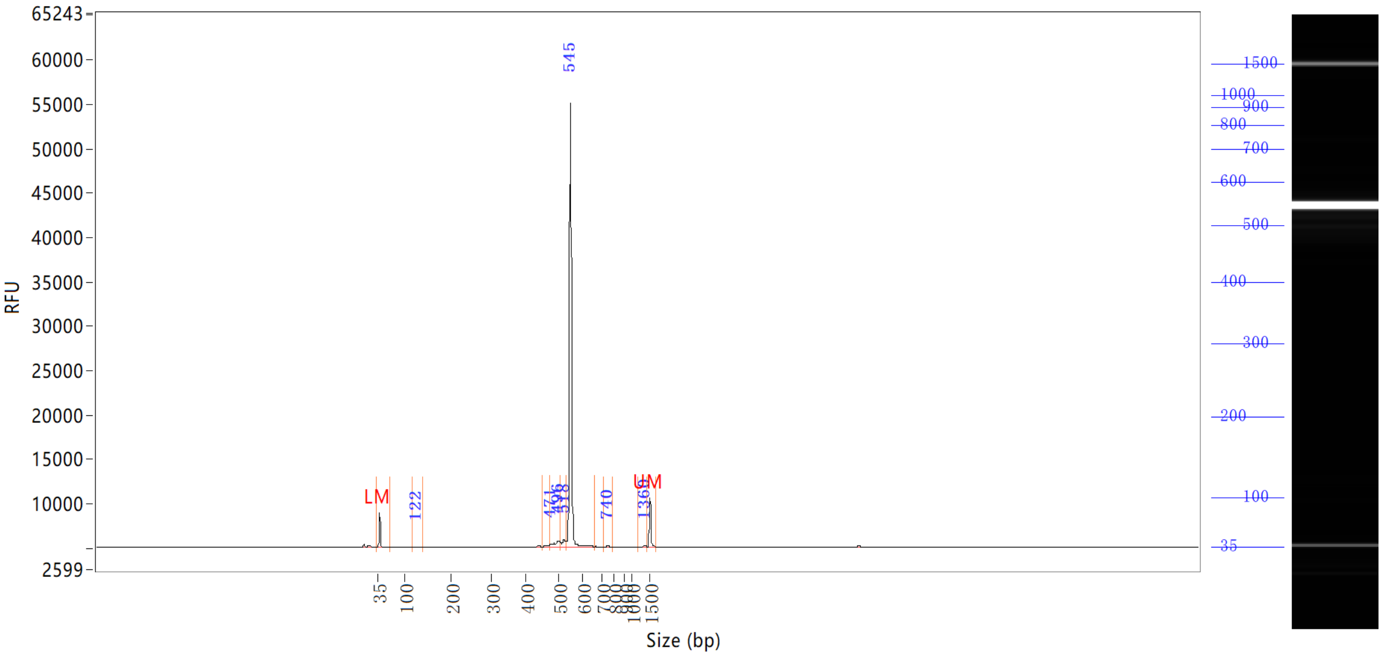

Peak	Size (bp)	Conc. (ng/uL)	From (bp)	To (bp)	Avg. Size (bp)	CV%	RFU	Corr. Peak Area
1	35 (LM)	0.4929	29	64	35	8.55	3824	18.873
2	122	0.2444	115	136	123	3.96	55	0.780
3	471	0.4259	449	472	466	1.17	231	1.359
4	496	2.9527	472	508	491	1.96	656	9.421
5	518	1.9498	508	528	519	1.12	706	6.221
6	545	79.2717	528	661	545	1.15	50016	252.917
7	740	0.1667	712	785	741	1.65	77	0.532
8	1360	0.1456	1187	1427	1338	4.15	53	0.465
9	1500 (UM)	0.5000	1427	1665	1498	1.74	5577	19.143

TIC:	85.1570	ng/uL
TIM:	260.7491	nmole/L
Total Conc.:	86.1331	ng/uL

Sample Peak Width (sec): 5 Sample Min Peak Height: 50 Sample Baseline V to V?: Y Sample Baseline V to V pts: 3
Sample Filter: Binomial # of Pts for Filter: 3 Sample Start Region (min): 0 Sample End Region (min): 75
Marker Peak Width (sec): 5 Marker Min Peak Height: 500 Marker Baseline V to V?: Y Marker Baseline V to V pts: 3
Lower Marker Selection: First Peak > 500 RFU Upper Marker Selection: Last Peak > 500 RFU
Ladder Size (bp)35, 100, 200, 300, 400, 500, 600, 700, 800, 900, 1000, 1500
Quantification Using: Upper Marker Final Concentration (ng/uL): 0.5000 Dilution Factor: 12.0

 Copyright 2015 Advanced Analytical Technologies, Inc.

Sample: SampB12
Well Location: B12

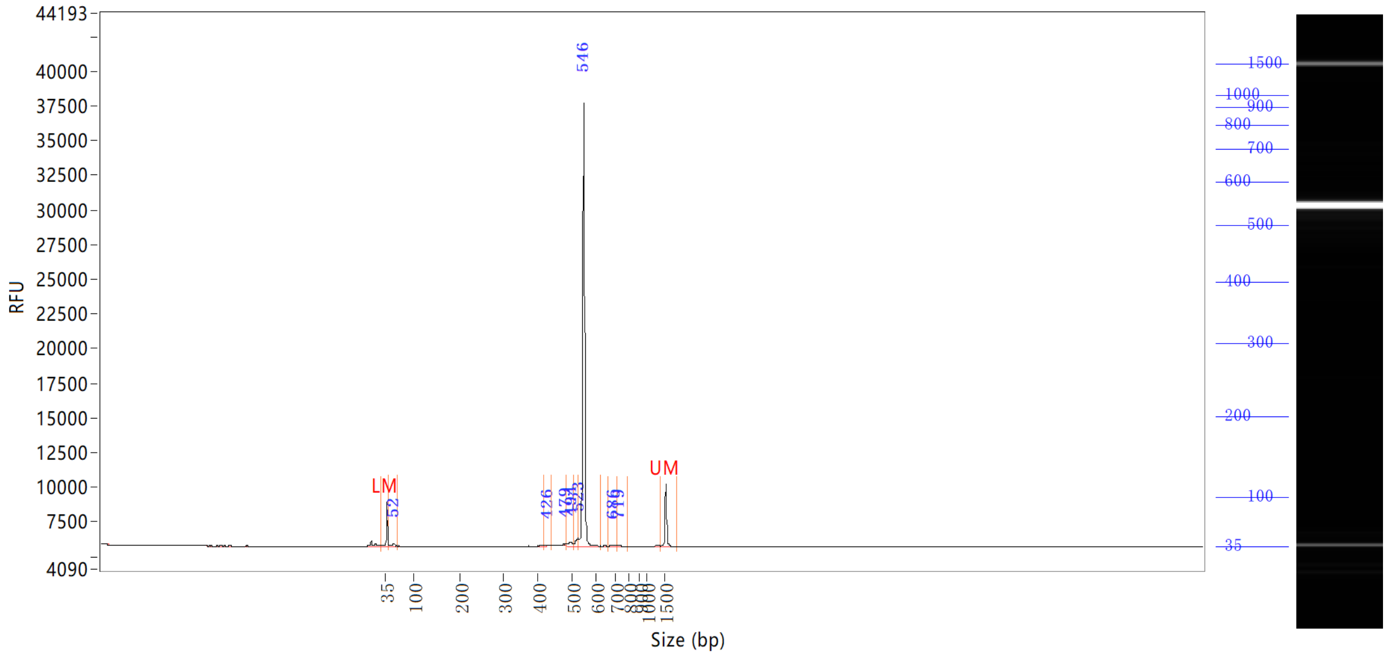

Peak	Size (bp)	Conc. (ng/uL)	From (bp)	To (bp)	Avg. Size (bp)	CV%	RFU	Corr. Peak Area
1	35 (LM)	0.4986	23	41	34	6.12	3226	15.675
2	52	0.8553	41	61	49	7.93	192	2.241
3	426	0.1489	416	440	426	1.14	61	0.390
4	479	0.8406	440	485	471	2.10	139	2.202
5	494	1.0540	485	505	495	1.11	261	2.761
6	523	1.9331	505	528	518	1.23	546	5.064
7	546	57.0906	528	621	545	0.98	32022	149.565
8	686	0.2778	658	703	681	1.69	64	0.728
9	719	0.1838	703	789	721	2.04	80	0.482
10	1500 (UM)	0.5000	1367	1823	1498	2.21	4456	15.719

TIC:	62.3841	ng/uL
TIM:	215.2141	nmole/L
Total Conc.:	63.6010	ng/uL

```
Sample Peak Width (sec): 5    Sample Min Peak Height: 50    Sample Baseline V to V?: Y    Sample Baseline V to V pts: 3
Sample Filter: Binomial       # of Pts for Filter: 3        Sample Start Region (min): 0  Sample End Region (min): 75
Marker Peak Width (sec): 5    Marker Min Peak Height: 500   Marker Baseline V to V?: Y    Marker Baseline V to V pts: 3
Lower Marker Selection: First Peak > 500 RFU                Upper Marker Selection: Last Peak > 500 RFU
Ladder Size (bp)35, 100, 200, 300, 400, 500, 600, 700, 800, 900, 1000, 1500
Quantification Using: Upper Marker          Final Concentration (ng/uL): 0.5000          Dilution Factor: 12.0
```

 Copyright 2015 Advanced Analytical Technologies, Inc.

Sample: SampC12
Well Location: C12

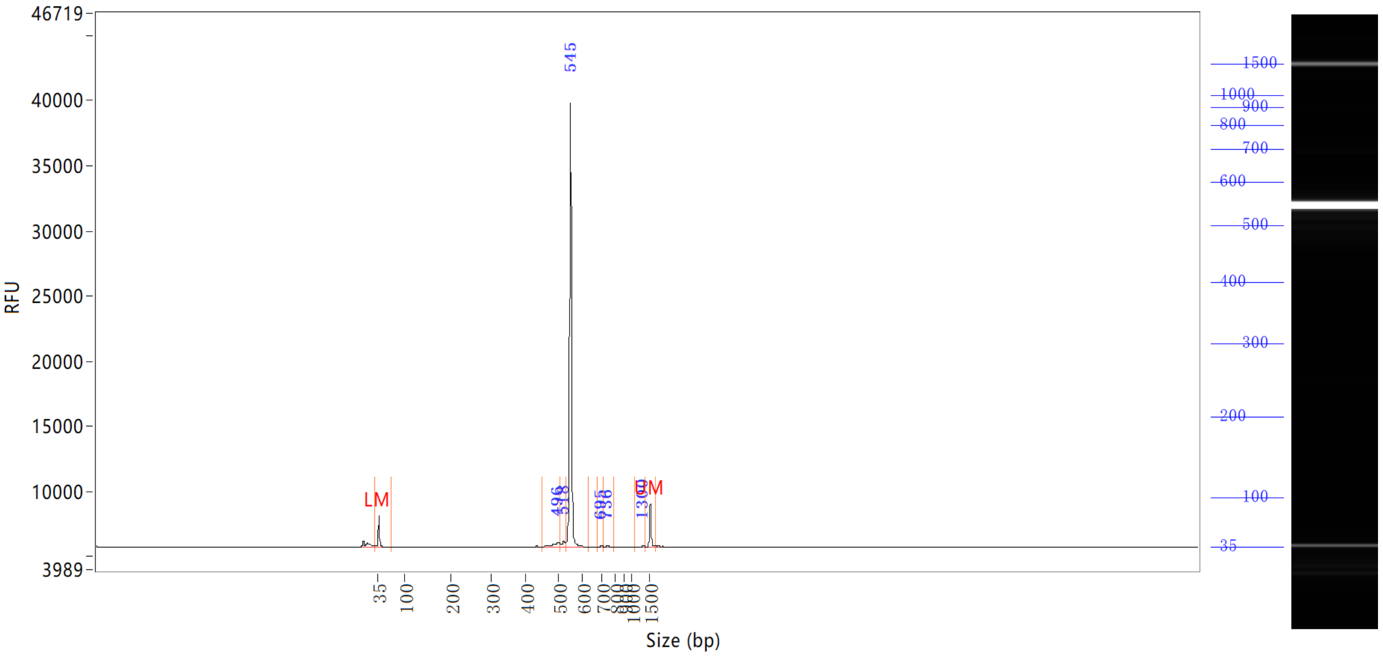

Peak	Size (bp)	Conc. (ng/uL)	From (bp)	To (bp)	Avg. Size (bp)	CV%	RFU	Corr. Peak Area
1	35 (LM)	0.5866	28	65	35	11.16	2453	13.569
2	496	3.0871	446	507	484	2.97	328	5.951
3	518	1.9884	507	527	518	1.11	425	3.833
4	545	85.0371	527	628	545	1.11	34148	163.924
5	695	0.3016	676	714	695	1.09	98	0.581
6	736	0.2532	714	791	741	2.28	55	0.488
7	1300	0.2454	1094	1380	1274	5.45	65	0.473
8	1500 (UM)	0.5000	1380	1678	1497	2.05	3284	11.566

TIC:	90.9127	ng/uL
TIM:	275.1668	nmole/L
Total Conc.:	92.7212	ng/uL

```
Sample Peak Width (sec): 5    Sample Min Peak Height: 50    Sample Baseline V to V?: Y    Sample Baseline V to V pts: 3
Sample Filter: Binomial       # of Pts for Filter: 3        Sample Start Region (min): 0  Sample End Region (min): 75
Marker Peak Width (sec): 5    Marker Min Peak Height: 500   Marker Baseline V to V?: Y    Marker Baseline V to V pts: 3
Lower Marker Selection: First Peak > 500 RFU                Upper Marker Selection: Last Peak > 500 RFU
Ladder Size (bp)35, 100, 200, 300, 400, 500, 600, 700, 800, 900, 1000, 1500
Quantification Using: Upper Marker          Final Concentration (ng/uL): 0.5000         Dilution Factor: 12.0
```

 Copyright 2015 Advanced Analytical Technologies, Inc.

Sample: SampD12
Well Location: D12

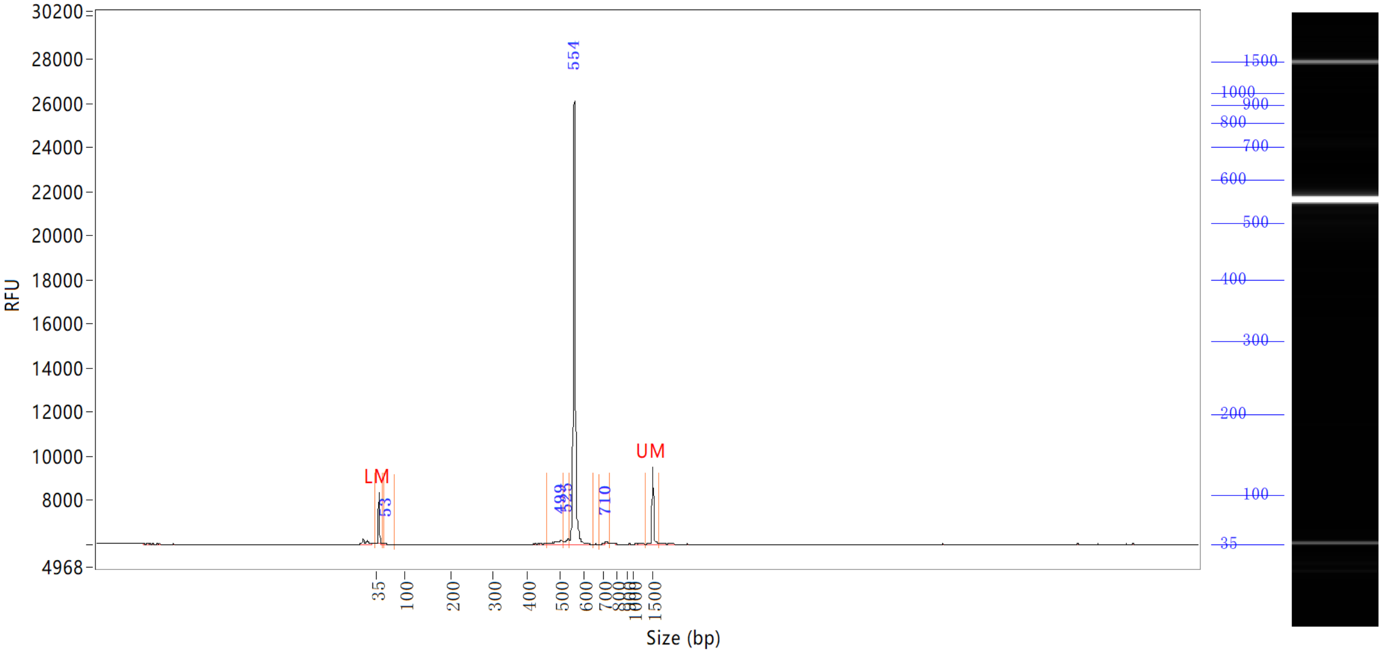

Peak	Size (bp)	Conc. (ng/uL)	From (bp)	To (bp)	Avg. Size (bp)	CV%	RFU	Corr. Peak Area
1	35 (LM)	0.4521	29	44	35	4.93	2333	11.199
2	53	0.2042	50	73	56	10.94	52	0.422
3	499	1.6645	459	513	491	2.66	182	3.436
4	525	0.9752	513	533	524	1.11	214	2.013
5	554	49.9620	533	640	554	1.28	20151	103.131
6	710	0.4368	680	749	712	1.80	100	0.902
7	1500 (UM)	0.5000	1327	1704	1498	2.32	3497	12.385

TIC:	53.2427	ng/uL
TIM:	164.0331	nmole/L
Total Conc.:	54.8549	ng/uL

```
Sample Peak Width (sec): 5     Sample Min Peak Height: 50    Sample Baseline V to V?: Y     Sample Baseline V to V pts: 3
Sample Filter: Binomial        # of Pts for Filter: 3        Sample Start Region (min): 0  Sample End Region (min): 75
Marker Peak Width (sec): 5     Marker Min Peak Height: 500   Marker Baseline V to V?: Y     Marker Baseline V to V pts: 3
Lower Marker Selection: First Peak > 500 RFU                 Upper Marker Selection: Last Peak > 500 RFU
Ladder Size (bp)35, 100, 200, 300, 400, 500, 600, 700, 800, 900, 1000, 1500
Quantification Using: Upper Marker          Final Concentration (ng/uL): 0.5000          Dilution Factor: 12.0
```

 Copyright 2015 Advanced Analytical Technologies, Inc.

Sample: SampE12
Well Location: E12

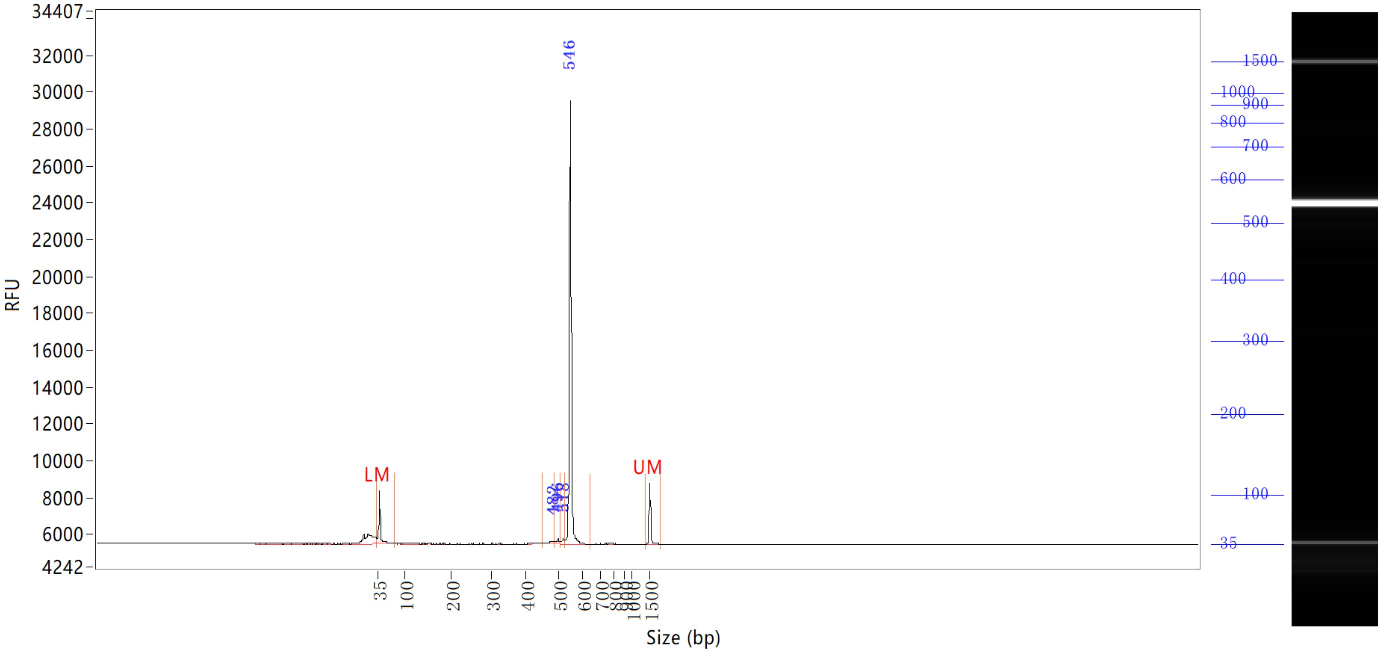

Peak	Size (bp)	Conc. (ng/uL)	From (bp)	To (bp)	Avg. Size (bp)	CV%	RFU	Corr. Peak Area
1	35 (LM)	0.8582	29	73	37	17.33	2903	19.919
2	482	0.9317	448	487	475	1.85	129	1.802
3	496	1.1704	487	508	496	1.12	243	2.264
4	518	1.1121	508	527	518	1.07	245	2.151
5	546	60.5576	527	637	545	1.25	24087	117.125
6	1500 (UM)	0.5000	1380	1823	1502	2.90	3293	11.605

TIC:	63.7717	ng/uL
TIM:	193.4910	nmole/L
Total Conc.:	65.7819	ng/uL

Sample Peak Width (sec): 5   Sample Min Peak Height: 50   Sample Baseline V to V?: Y   Sample Baseline V to V pts: 3
Sample Filter: Binomial   # of Pts for Filter: 3   Sample Start Region (min): 0   Sample End Region (min): 75
Marker Peak Width (sec): 5   Marker Min Peak Height: 500   Marker Baseline V to V?: Y   Marker Baseline V to V pts: 3
Lower Marker Selection: First Peak > 500 RFU   Upper Marker Selection: Last Peak > 500 RFU
Ladder Size (bp)35, 100, 200, 300, 400, 500, 600, 700, 800, 900, 1000, 1500
Quantification Using: Upper Marker   Final Concentration (ng/uL): 0.5000   Dilution Factor: 12.0

 Copyright 2015 Advanced Analytical Technologies, Inc.

Sample: SampH12
Well Location: H12

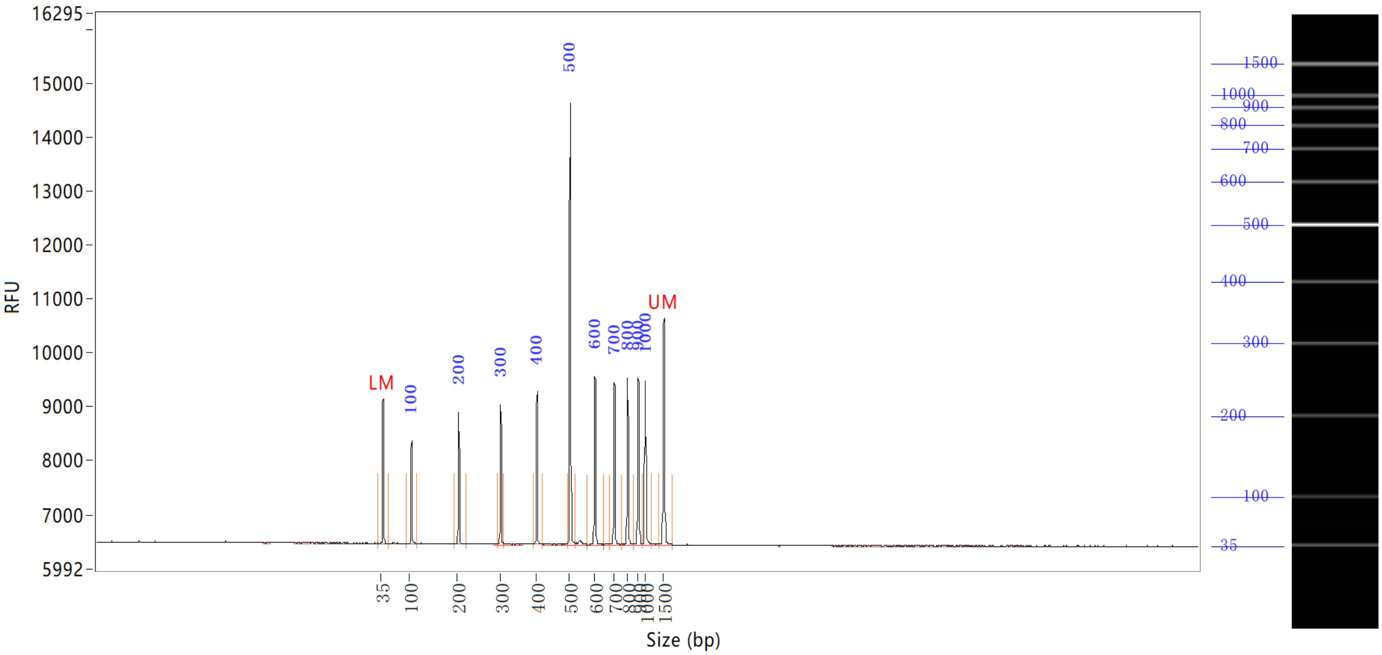

Peak	Size (bp)	Conc. (ng/uL)	From (bp)	To (bp)	Avg. Size (bp)	CV%	RFU	Corr. Peak Area
1	35 (LM)	0.4564	24	48	34	4.59	2693	12.423
2	100	3.3592	92	113	100	1.65	1901	7.621
3	200	3.8291	192	218	200	0.98	2443	8.686
4	300	3.8421	294	309	299	0.53	2591	8.716
5	400	3.8958	393	419	399	0.49	2824	8.838
6	500	11.2787	493	523	499	0.51	8198	25.586
7	600	4.5096	571	645	600	0.75	3117	10.230
8	700	4.1626	680	762	700	0.79	3006	9.443
9	800	4.2182	762	860	798	0.83	3088	9.569
10	900	4.2750	860	965	899	0.99	3081	9.698
11	1000	4.3714	965	1174	998	1.93	3045	9.917
12	1500 (UM)	0.5000	1367	1757	1493	1.84	4194	13.611

TIC:	47.7416	ng/uL
TIM:	206.9259	nmole/L
Total Conc.:	49.4039	ng/uL

```
Sample Peak Width (sec): 5    Sample Min Peak Height: 500   Sample Baseline V to V?: Y    Sample Baseline V to V pts: 3
Sample Filter: Binomial       # of Pts for Filter: 3        Sample Start Region (min): 0  Sample End Region (min): 75
Marker Peak Width (sec): 5    Marker Min Peak Height: 500   Marker Baseline V to V?: Y    Marker Baseline V to V pts: 3
Lower Marker Selection: First Peak > 500 RFU                Upper Marker Selection: Last Peak > 500 RFU
Ladder Size (bp)35, 100, 200, 300, 400, 500, 600, 700, 800, 900, 1000, 1500
Quantification Using: Upper Marker          Final Concentration (ng/uL): 0.5000          Dilution Factor: 12.0
```

 Copyright 2015 Advanced Analytical Technologies, Inc.

Sample: SampH12
Well Location: H12
Created:
Fit Type: Point to Point

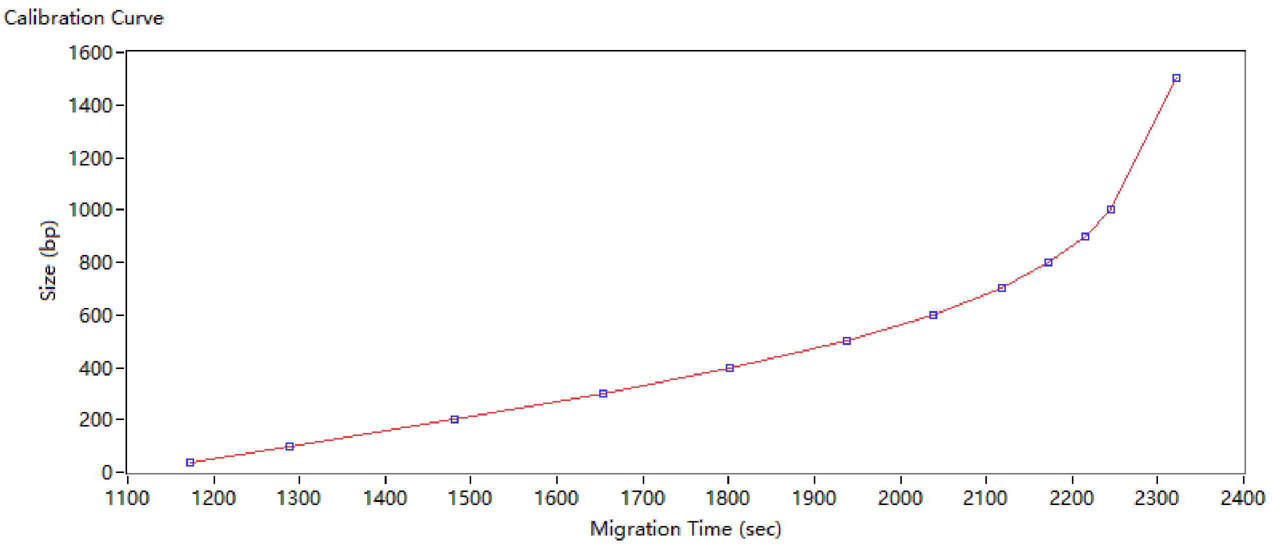

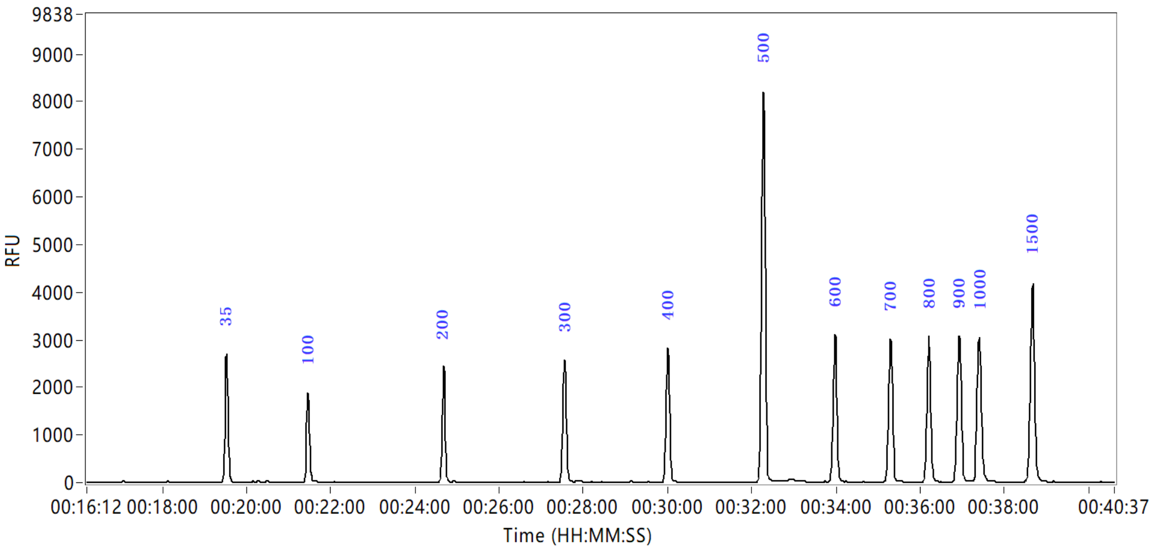

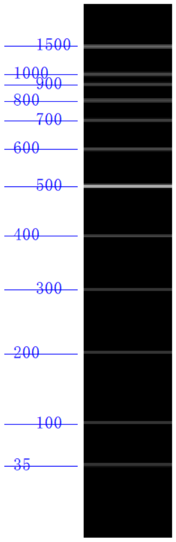

 Copyright 2015 Advanced Analytical Technologies, Inc.

Fragment Analyzer Run Summary:

Analysis Mode: DNA

 Copyright 2015 Advanced Analytical Technologies, Inc.

Gel Image

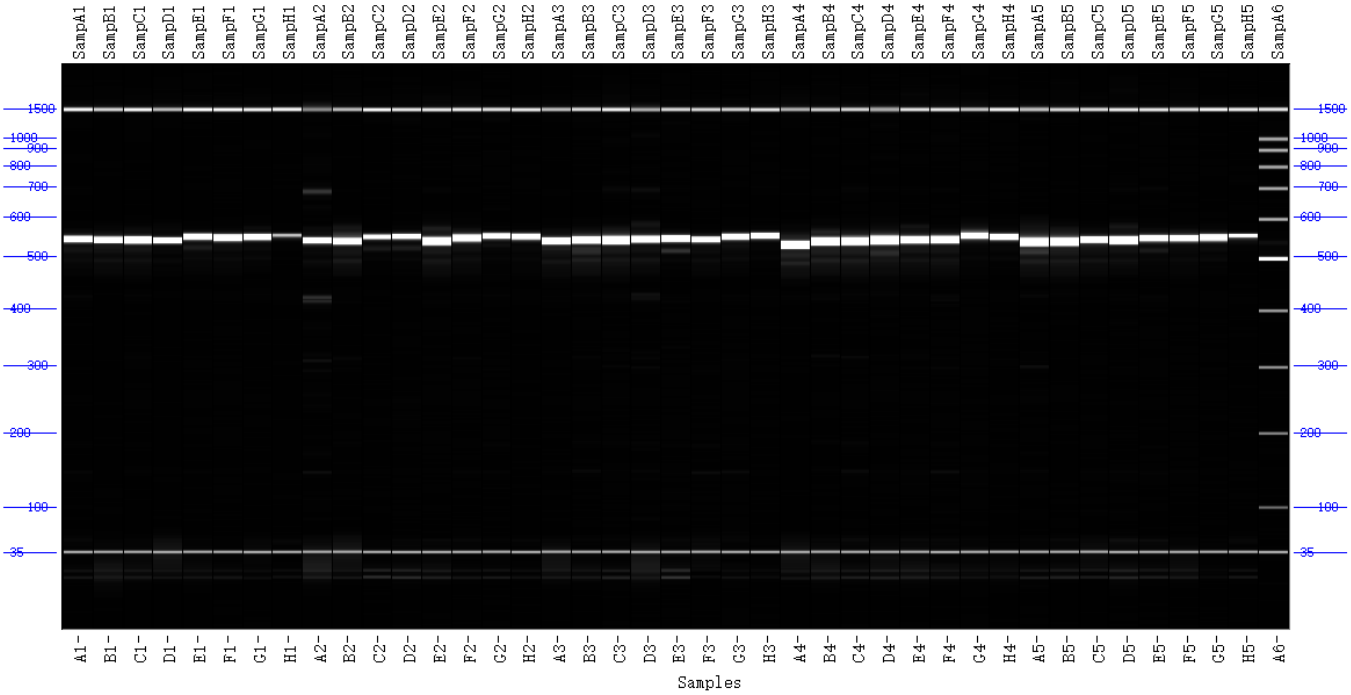

 Copyright 2015 Advanced Analytical Technologies, Inc.

Sample: SampA1
Well Location: A1

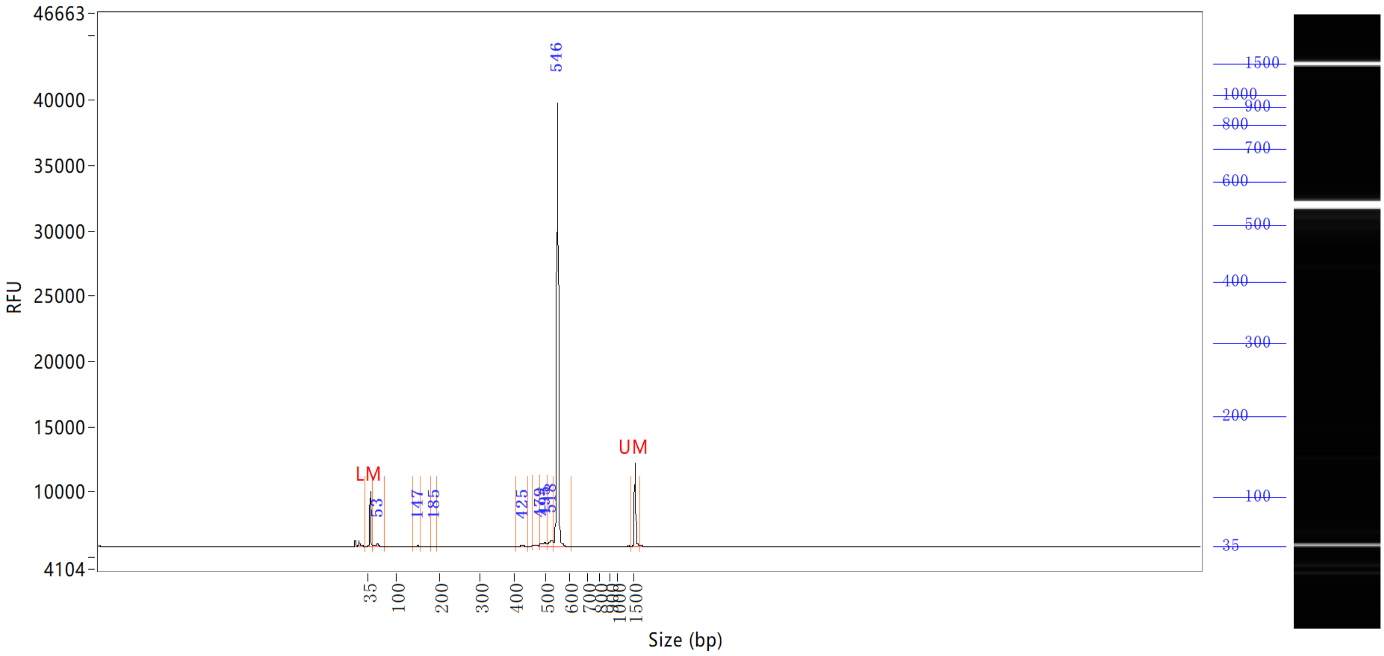

Peak	Size (bp)	Conc. (ng/uL)	From (bp)	To (bp)	Avg. Size (bp)	CV%	RFU	Corr. Peak Area
1	35 (LM)	0.4574	23	41	34	5.09	4228	19.868
2	53	0.7489	41	71	51	11.63	182	2.711
3	147	0.2380	138	154	146	2.01	123	0.861
4	185	0.1121	177	191	184	1.45	56	0.406
5	425	0.2575	405	441	423	1.50	125	0.932
6	479	0.2963	459	480	472	1.16	118	1.072
7	495	0.9075	480	506	494	1.40	279	3.285
8	518	1.1853	506	529	518	1.23	470	4.290
9	546	41.5659	529	609	546	0.85	34003	150.456
10	1500 (UM)	0.5000	1394	1658	1497	1.80	6361	21.718

TIC:	45.3114	ng/uL
TIM:	161.8301	nmole/L
Total Conc.:	46.4032	ng/uL

Sample Peak Width (sec): 5   Sample Min Peak Height: 50   Sample Baseline V to V?: Y   Sample Baseline V to V pts: 3
Sample Filter: Binomial   # of Pts for Filter: 3   Sample Start Region (min): 0   Sample End Region (min): 80
Marker Peak Width (sec): 5   Marker Min Peak Height: 500   Marker Baseline V to V?: Y   Marker Baseline V to V pts: 3
Lower Marker Selection: First Peak > 500 RFU   Upper Marker Selection: Last Peak > 500 RFU
Ladder Size (bp)35, 100, 200, 300, 400, 500, 600, 700, 800, 900, 1000, 1500
Quantification Using: Upper Marker   Final Concentration (ng/uL): 0.5000   Dilution Factor: 12.0

Copyright 2015 Advanced Analytical Technologies, Inc.

Sample: SampB1
Well Location: B1

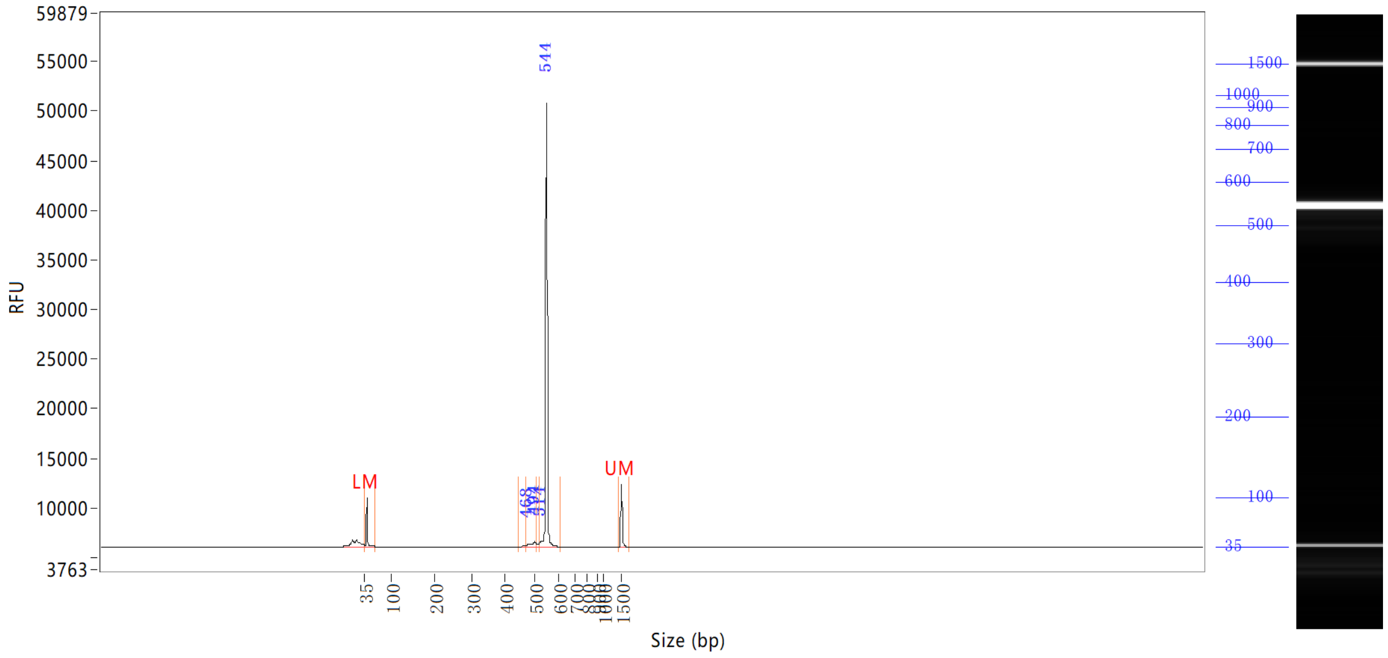

Peak	Size (bp)	Conc. (ng/uL)	From (bp)	To (bp)	Avg. Size (bp)	CV%	RFU	Corr. Peak Area
1	35 (LM)	0.5961	31	59	36	11.55	4940	26.661
2	468	0.2420	442	468	463	1.17	165	0.902
3	494	1.8819	468	505	488	2.01	507	7.014
4	514	0.5108	505	515	511	0.60	437	1.904
5	544	54.5864	515	612	543	1.06	44827	203.455
6	1500 (UM)	0.5000	1420	1730	1499	2.18	6324	22.363

TIC:	57.2212	ng/uL
TIM:	174.2760	nmole/L
Total Conc.:	58.3611	ng/uL

Sample Peak Width (sec): 5   Sample Min Peak Height: 50   Sample Baseline V to V?: Y   Sample Baseline V to V pts: 3
Sample Filter: Binomial   # of Pts for Filter: 3   Sample Start Region (min): 0   Sample End Region (min): 80
Marker Peak Width (sec): 5   Marker Min Peak Height: 500   Marker Baseline V to V?: Y   Marker Baseline V to V pts: 3
Lower Marker Selection: First Peak > 500 RFU   Upper Marker Selection: Last Peak > 500 RFU
Ladder Size (bp)35, 100, 200, 300, 400, 500, 600, 700, 800, 900, 1000, 1500
Quantification Using: Upper Marker   Final Concentration (ng/uL): 0.5000   Dilution Factor: 12.0

 Copyright 2015 Advanced Analytical Technologies, Inc.

Sample: SampC1
Well Location: C1

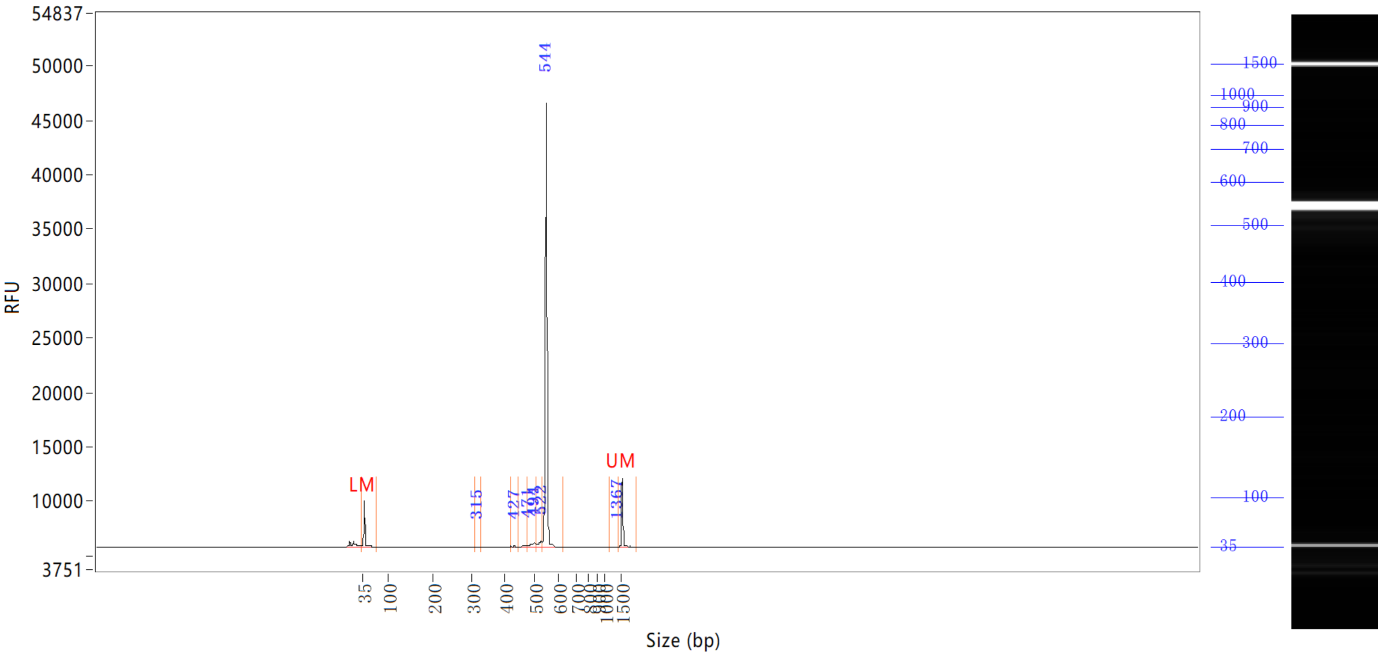

Peak	Size (bp)	Conc. (ng/uL)	From (bp)	To (bp)	Avg. Size (bp)	CV%	RFU	Corr. Peak Area
1	35 (LM)	0.5179	28	66	36	13.08	4253	23.147
2	315	0.1022	310	327	316	1.24	62	0.381
3	427	0.1288	422	443	429	1.09	67	0.480
4	471	0.4670	443	475	466	1.52	162	1.739
5	494	1.2514	475	503	490	1.52	380	4.661
6	522	1.3669	503	526	516	1.27	539	5.091
7	544	51.3554	526	623	544	1.03	40843	191.290
8	1367	0.1599	1134	1420	1295	6.05	54	0.596
9	1500 (UM)	0.5000	1420	1908	1501	3.17	6311	22.349

TIC:	54.8316	ng/uL
TIM:	166.7873	nmole/L
Total Conc.:	56.0254	ng/uL

Sample Peak Width (sec): 5   Sample Min Peak Height: 50   Sample Baseline V to V?: Y   Sample Baseline V to V pts: 3
Sample Filter: Binomial   # of Pts for Filter: 3   Sample Start Region (min): 0   Sample End Region (min): 80
Marker Peak Width (sec): 5   Marker Min Peak Height: 500   Marker Baseline V to V?: Y   Marker Baseline V to V pts: 3
Lower Marker Selection: First Peak > 500 RFU   Upper Marker Selection: Last Peak > 500 RFU
Ladder Size (bp)35, 100, 200, 300, 400, 500, 600, 700, 800, 900, 1000, 1500
Quantification Using: Upper Marker   Final Concentration (ng/uL): 0.5000   Dilution Factor: 12.0

Copyright 2015 Advanced Analytical Technologies, Inc.

Sample: SampD1
Well Location: D1

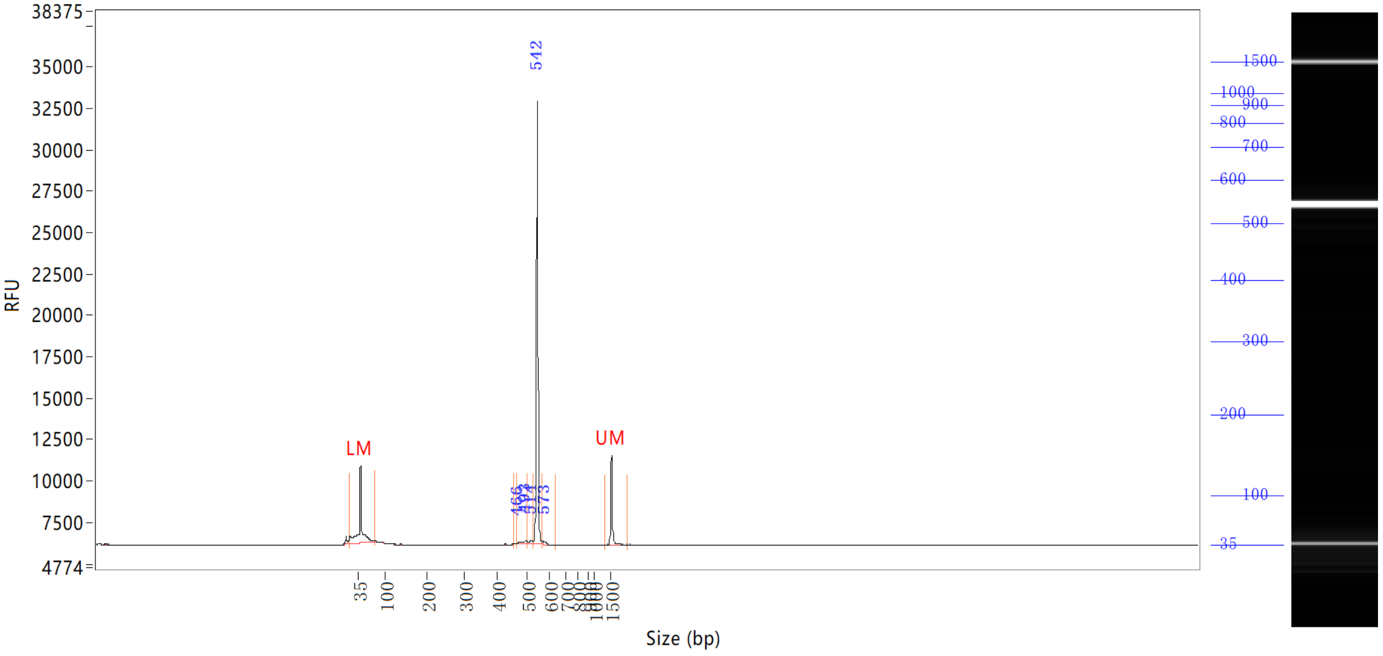

Peak	Size (bp)	Conc. (ng/uL)	From (bp)	To (bp)	Avg. Size (bp)	CV%	RFU	Corr. Peak Area
1	35 (LM)	1.2327	11	74	34	31.82	4689	50.658
2	466	0.0515	455	467	465	0.39	60	0.177
3	493	0.7330	467	503	487	1.95	219	2.510
4	514	0.4845	503	526	515	1.16	195	1.659
5	542	33.5470	526	567	542	0.78	26802	114.883
6	573	0.5050	567	638	577	1.46	198	1.730
7	1500 (UM)	0.5000	1314	2013	1514	5.05	5368	20.547

TIC:	35.3211	ng/uL
TIM:	107.5001	nmole/L
Total Conc.:	36.3721	ng/uL

```
Sample Peak Width (sec): 5    Sample Min Peak Height: 50    Sample Baseline V to V?: Y    Sample Baseline V to V pts: 3
Sample Filter: Binomial       # of Pts for Filter: 3        Sample Start Region (min): 0  Sample End Region (min): 80
Marker Peak Width (sec): 5    Marker Min Peak Height: 500   Marker Baseline V to V?: Y    Marker Baseline V to V pts: 3
Lower Marker Selection: First Peak > 500 RFU                Upper Marker Selection: Last Peak > 500 RFU
Ladder Size (bp)35, 100, 200, 300, 400, 500, 600, 700, 800, 900, 1000, 1500
Quantification Using: Upper Marker          Final Concentration (ng/uL): 0.5000          Dilution Factor: 12.0
```

 Copyright 2015 Advanced Analytical Technologies, Inc.

Sample: SampE1
Well Location: E1

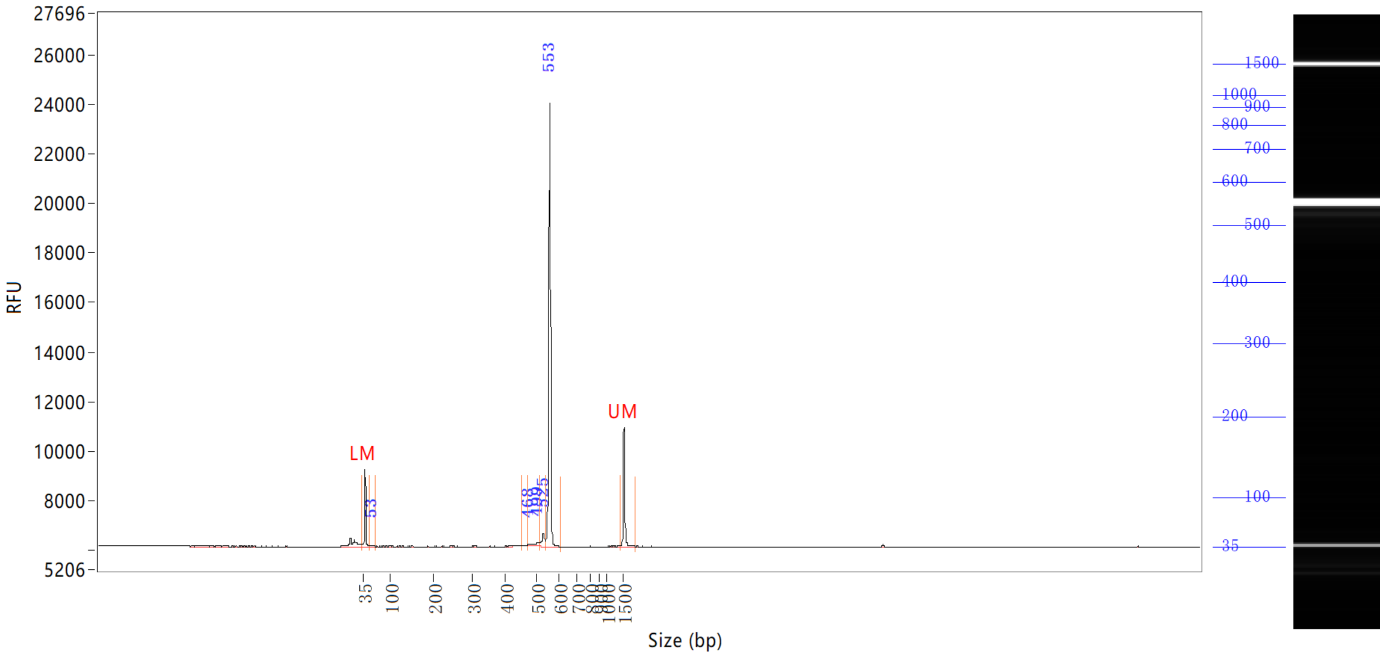

Peak	Size (bp)	Conc. (ng/uL)	From (bp)	To (bp)	Avg. Size (bp)	CV%	RFU	Corr. Peak Area
1	35 (LM)	0.4703	28	46	35	6.40	3117	15.857
2	53	0.2449	46	64	52	6.49	62	0.688
3	468	0.0956	452	469	464	1.00	53	0.269
4	499	0.9884	469	509	491	2.21	165	2.777
5	525	1.4771	509	535	523	1.25	519	4.150
6	553	29.4074	535	602	552	0.84	17960	82.628
7	1500 (UM)	0.5000	1400	1829	1500	2.73	4786	16.859

TIC:	32.2135	ng/uL
TIM:	103.6820	nmole/L
Total Conc.:	33.7015	ng/uL

```
Sample Peak Width (sec): 5    Sample Min Peak Height: 50    Sample Baseline V to V?: Y    Sample Baseline V to V pts: 3
Sample Filter: Binomial       # of Pts for Filter: 3        Sample Start Region (min): 0  Sample End Region (min): 80
Marker Peak Width (sec): 5    Marker Min Peak Height: 500   Marker Baseline V to V?: Y    Marker Baseline V to V pts: 3
Lower Marker Selection: First Peak > 500 RFU                Upper Marker Selection: Last Peak > 500 RFU
Ladder Size (bp)35, 100, 200, 300, 400, 500, 600, 700, 800, 900, 1000, 1500
Quantification Using: Upper Marker          Final Concentration (ng/uL): 0.5000          Dilution Factor: 12.0
```

 Copyright 2015 Advanced Analytical Technologies, Inc.

Sample: SampF1
Well Location: F1

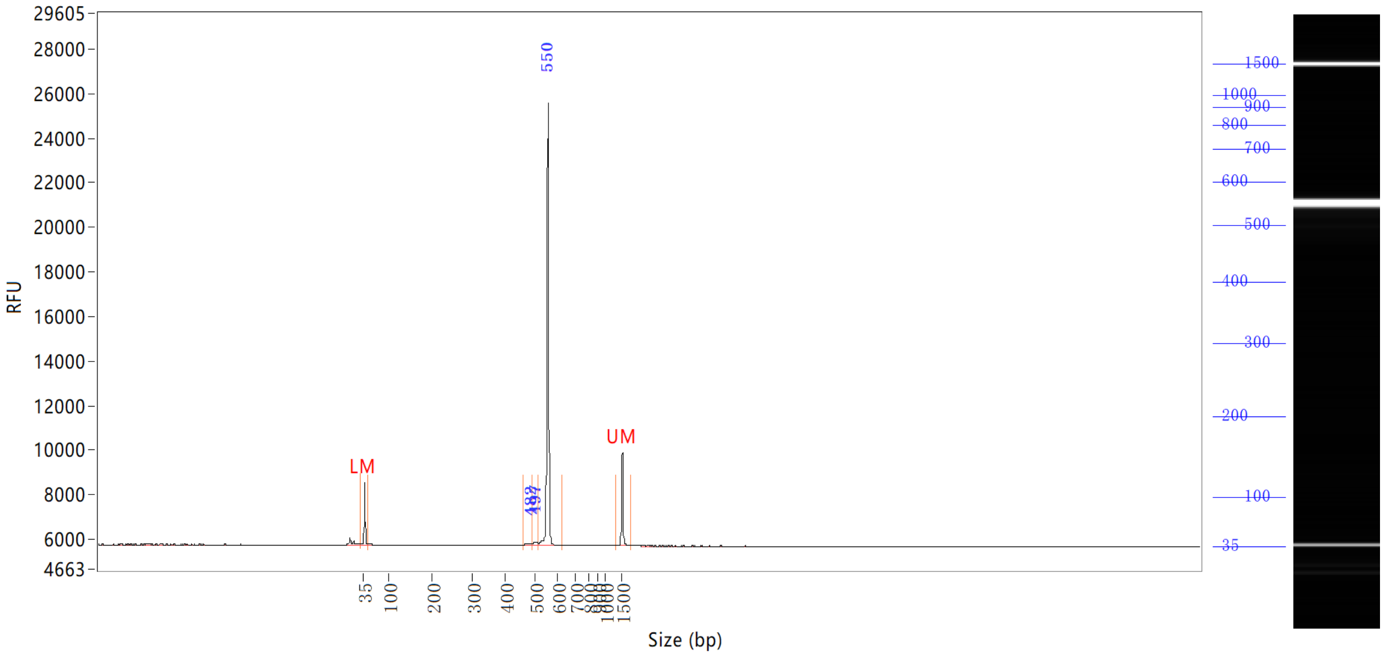

Peak	Size (bp)	Conc. (ng/uL)	From (bp)	To (bp)	Avg. Size (bp)	CV%	RFU	Corr. Peak Area
1	35 (LM)	0.4530	28	44	35	4.66	2785	13.205
2	482	0.5518	457	490	477	1.74	84	1.340
3	497	0.6463	490	512	499	1.23	159	1.570
4	550	37.4048	512	616	549	1.09	19880	90.851
5	1500 (UM)	0.5000	1314	1744	1496	2.46	4172	14.573

TIC:	38.6029	ng/uL
TIM:	116.1526	nmole/L
Total Conc.:	40.5686	ng/uL

```
Sample Peak Width (sec): 5    Sample Min Peak Height: 50    Sample Baseline V to V?: Y    Sample Baseline V to V pts: 3
Sample Filter: Binomial       # of Pts for Filter: 3        Sample Start Region (min): 0  Sample End Region (min): 80
Marker Peak Width (sec): 5    Marker Min Peak Height: 500   Marker Baseline V to V?: Y    Marker Baseline V to V pts: 3
Lower Marker Selection: First Peak > 500 RFU                Upper Marker Selection: Last Peak > 500 RFU
Ladder Size (bp)35, 100, 200, 300, 400, 500, 600, 700, 800, 900, 1000, 1500
Quantification Using: Upper Marker          Final Concentration (ng/uL): 0.5000          Dilution Factor: 12.0
```

Sample: SampG1
Well Location: G1

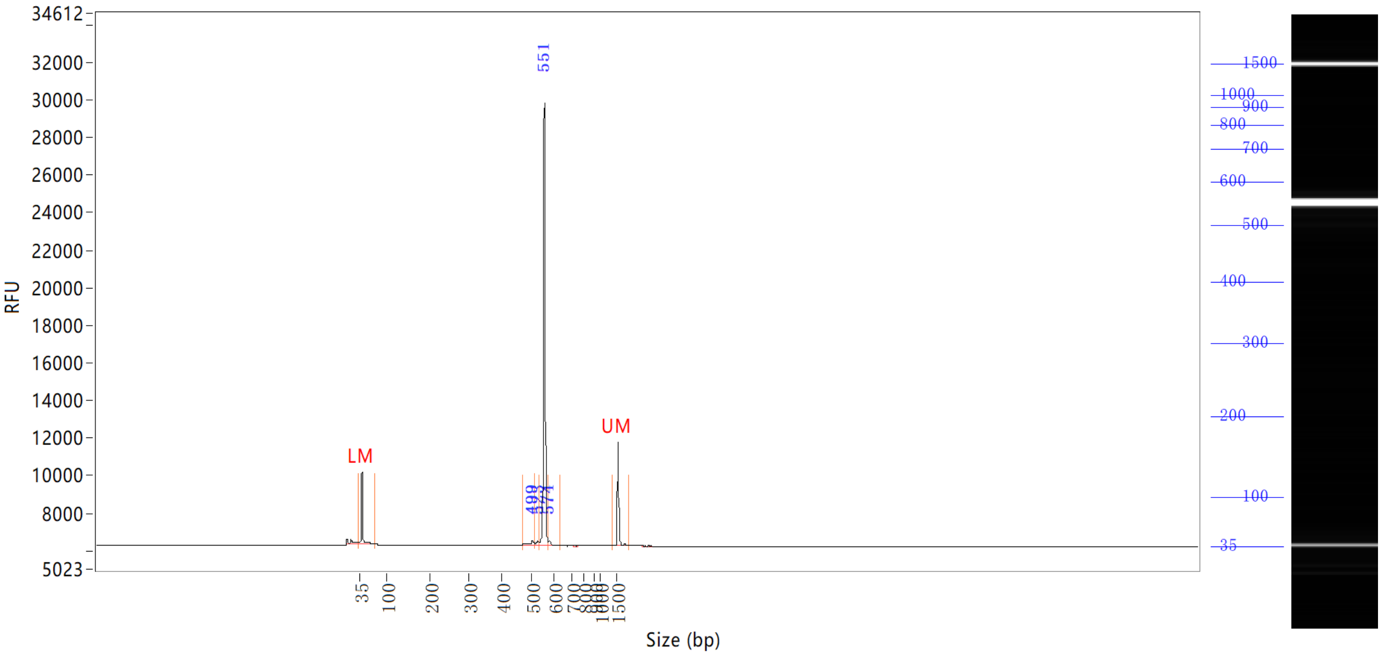

Peak	Size (bp)	Conc. (ng/uL)	From (bp)	To (bp)	Avg. Size (bp)	CV%	RFU	Corr. Peak Area
1	35 (LM)	0.5793	29	70	36	14.81	3797	22.120
2	499	0.7073	471	512	494	2.12	186	2.251
3	523	0.5457	512	533	523	1.14	200	1.737
4	551	32.2984	533	569	550	0.76	23573	102.782
5	574	0.5276	569	631	578	1.74	256	1.679
6	1500 (UM)	0.5000	1360	1822	1500	2.90	5483	19.094

TIC:	34.0791	ng/uL
TIM:	102.2112	nmole/L
Total Conc.:	35.4063	ng/uL

Sample Peak Width (sec): 5 Sample Min Peak Height: 50 Sample Baseline V to V?: Y Sample Baseline V to V pts: 3
Sample Filter: Binomial # of Pts for Filter: 3 Sample Start Region (min): 0 Sample End Region (min): 80
Marker Peak Width (sec): 5 Marker Min Peak Height: 500 Marker Baseline V to V?: Y Marker Baseline V to V pts: 3
Lower Marker Selection: First Peak > 500 RFU Upper Marker Selection: Last Peak > 500 RFU
Ladder Size (bp)35, 100, 200, 300, 400, 500, 600, 700, 800, 900, 1000, 1500
Quantification Using: Upper Marker Final Concentration (ng/uL): 0.5000 Dilution Factor: 12.0

 Copyright 2015 Advanced Analytical Technologies, Inc.

Sample: SampH1
Well Location: H1

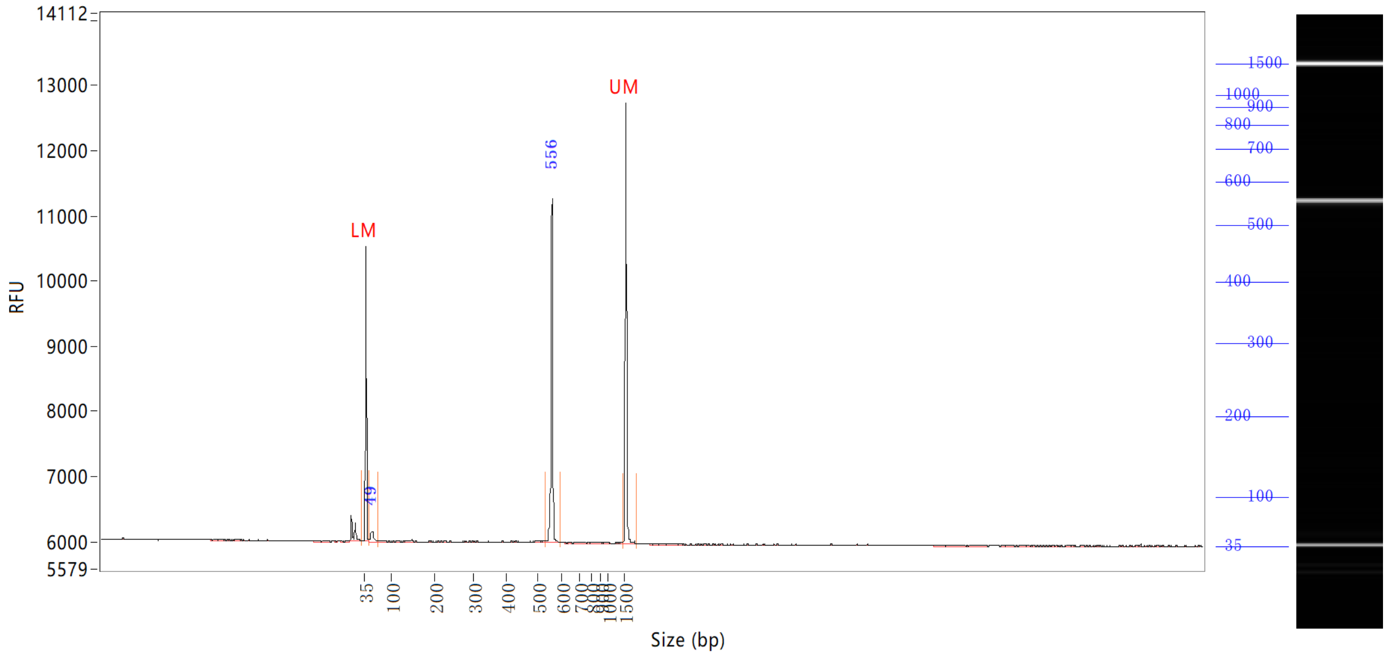

Peak	Size (bp)	Conc. (ng/uL)	From (bp)	To (bp)	Avg. Size (bp)	CV%	RFU	Corr. Peak Area
1	35 (LM)	0.4462	26	44	35	4.51	4523	20.977
2	49	0.5336	44	66	51	8.85	151	2.091
3	556	5.6046	533	592	554	0.89	5283	21.958
4	1500 (UM)	0.5000	1420	1836	1500	2.29	6777	23.508

TIC:	6.1382	ng/uL
TIM:	33.7860	nmole/L
Total Conc.:	7.5413	ng/uL

```
Sample Peak Width (sec): 5    Sample Min Peak Height: 50    Sample Baseline V to V?: Y    Sample Baseline V to V pts: 3
Sample Filter: Binomial       # of Pts for Filter: 3        Sample Start Region (min): 0  Sample End Region (min): 80
Marker Peak Width (sec): 5    Marker Min Peak Height: 500   Marker Baseline V to V?: Y    Marker Baseline V to V pts: 3
Lower Marker Selection: First Peak > 500 RFU                Upper Marker Selection: Last Peak > 500 RFU
Ladder Size (bp)35, 100, 200, 300, 400, 500, 600, 700, 800, 900, 1000, 1500
Quantification Using: Upper Marker          Final Concentration (ng/uL): 0.5000          Dilution Factor: 12.0
```


Copyright 2015 Advanced Analytical Technologies, Inc.

Sample: SampA2
Well Location: A2

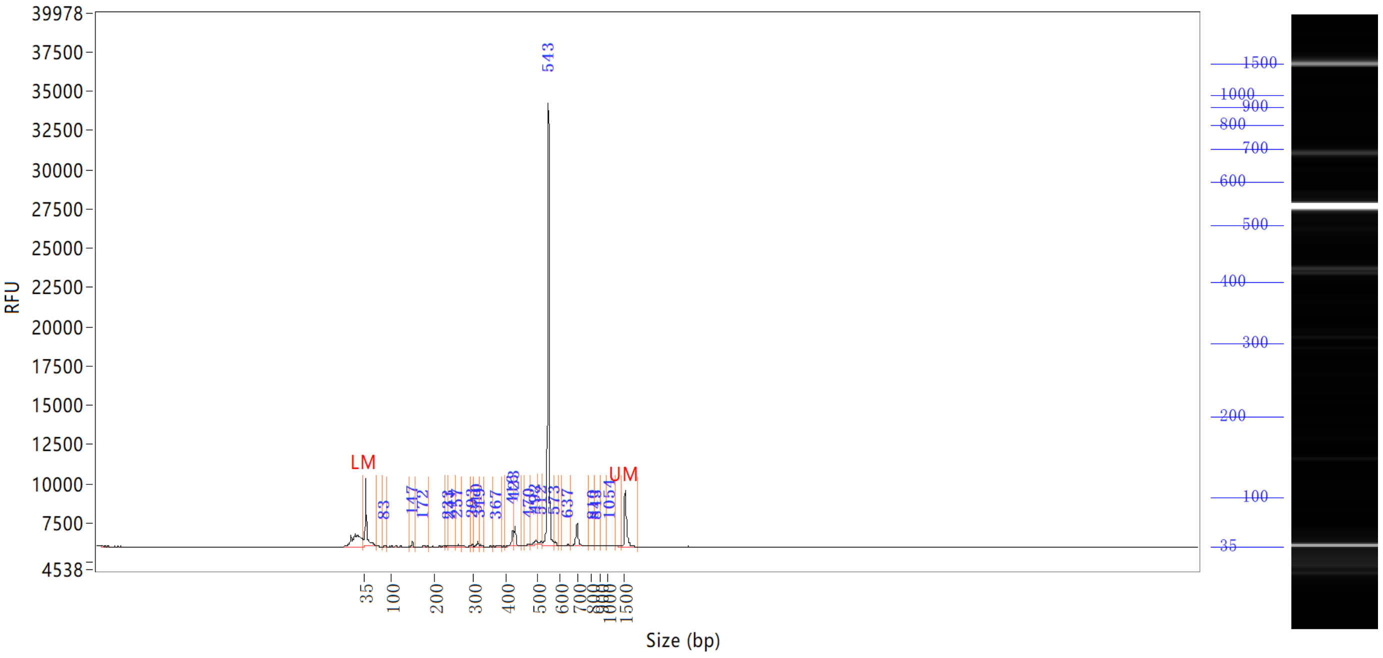

Peak	Size (bp)	Conc. (ng/uL)	From (bp)	To (bp)	Avg. Size (bp)	CV%	RFU	Corr. Peak Area
1	35 (LM)	0.8516	31	65	38	15.48	4376	29.650
2	83	0.1317	78	89	82	2.05	60	0.382
3	147	0.6270	142	155	146	1.13	352	1.819
4	172	0.3582	155	184	173	2.95	92	1.039
5	233	0.0739	227	234	232	0.65	52	0.214
6	241	0.2443	234	251	242	1.82	75	0.709
7	257	0.2655	251	268	259	1.58	110	0.770
8	293	0.2960	289	299	293	0.74	182	0.859
9	310	0.6060	299	315	309	0.88	405	1.758
10	319	0.2896	315	331	320	0.92	127	0.840
11	367	0.2630	359	386	371	1.46	83	0.763
12	416	1.7730	393	419	414	1.13	961	5.144
13	423	2.1281	419	445	424	0.91	1238	6.174
14	470	0.1648	457	473	467	0.79	66	0.478
15	492	1.0087	473	500	488	1.33	291	2.927
16	512	0.5259	500	517	509	0.92	221	1.526
17	543	41.9881	517	568	542	0.91	28221	121.818
18	573	0.4745	568	590	573	0.67	270	1.377
19	637	0.2490	605	650	629	1.52	95	0.722
20	810	0.2327	774	829	801	1.92	66	0.675
21	843	0.1507	829	898	850	1.82	58	0.437
22	1054	0.1638	982	1221	1070	5.20	66	0.475
23	1500 (UM)	0.5000	1400	1895	1515	3.53	3604	17.407
	TIC:	52.0145	ng/uL					

Sample Peak Width (sec): 5   Sample Min Peak Height: 50   Sample Baseline V to V?: Y   Sample Baseline V to V pts: 3
Sample Filter: Binomial   # of Pts for Filter: 3   Sample Start Region (min): 0   Sample End Region (min): 80
Marker Peak Width (sec): 5   Marker Min Peak Height: 500   Marker Baseline V to V?: Y   Marker Baseline V to V pts: 3
Lower Marker Selection: First Peak > 500 RFU   Upper Marker Selection: Last Peak > 500 RFU
Ladder Size (bp)35, 100, 200, 300, 400, 500, 600, 700, 800, 900, 1000, 1500
Quantification Using: Upper Marker   Final Concentration (ng/uL): 0.5000   Dilution Factor: 12.0

 Copyright 2015 Advanced Analytical Technologies, Inc.

Sample: SampA2
Well Location: A2

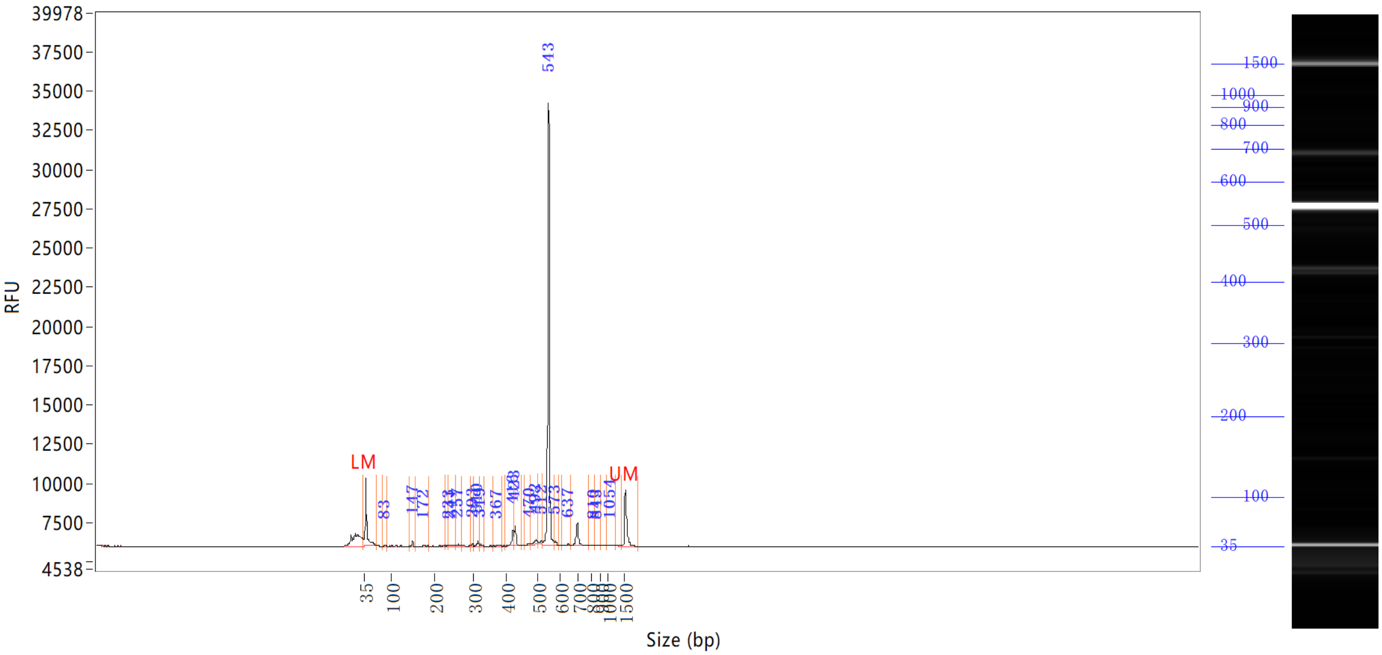

Peak	Size (bp)	Conc. (ng/uL)	From (bp)	To (bp)	Avg. Size (bp)	CV%	RFU	Corr. Peak Area

TIM:	176.0138	nmole/L
Total Conc.:	55.8479	ng/uL

```
Sample Peak Width (sec): 5    Sample Min Peak Height: 50    Sample Baseline V to V?: Y    Sample Baseline V to V pts: 3
Sample Filter: Binomial       # of Pts for Filter: 3        Sample Start Region (min): 0  Sample End Region (min): 80
Marker Peak Width (sec): 5    Marker Min Peak Height: 500   Marker Baseline V to V?: Y    Marker Baseline V to V pts: 3
Lower Marker Selection: First Peak > 500 RFU                Upper Marker Selection: Last Peak > 500 RFU
Ladder Size (bp)35, 100, 200, 300, 400, 500, 600, 700, 800, 900, 1000, 1500
Quantification Using: Upper Marker          Final Concentration (ng/uL): 0.5000          Dilution Factor: 12.0
```

 Copyright 2015 Advanced Analytical Technologies, Inc.

Sample: SampB2
Well Location: B2

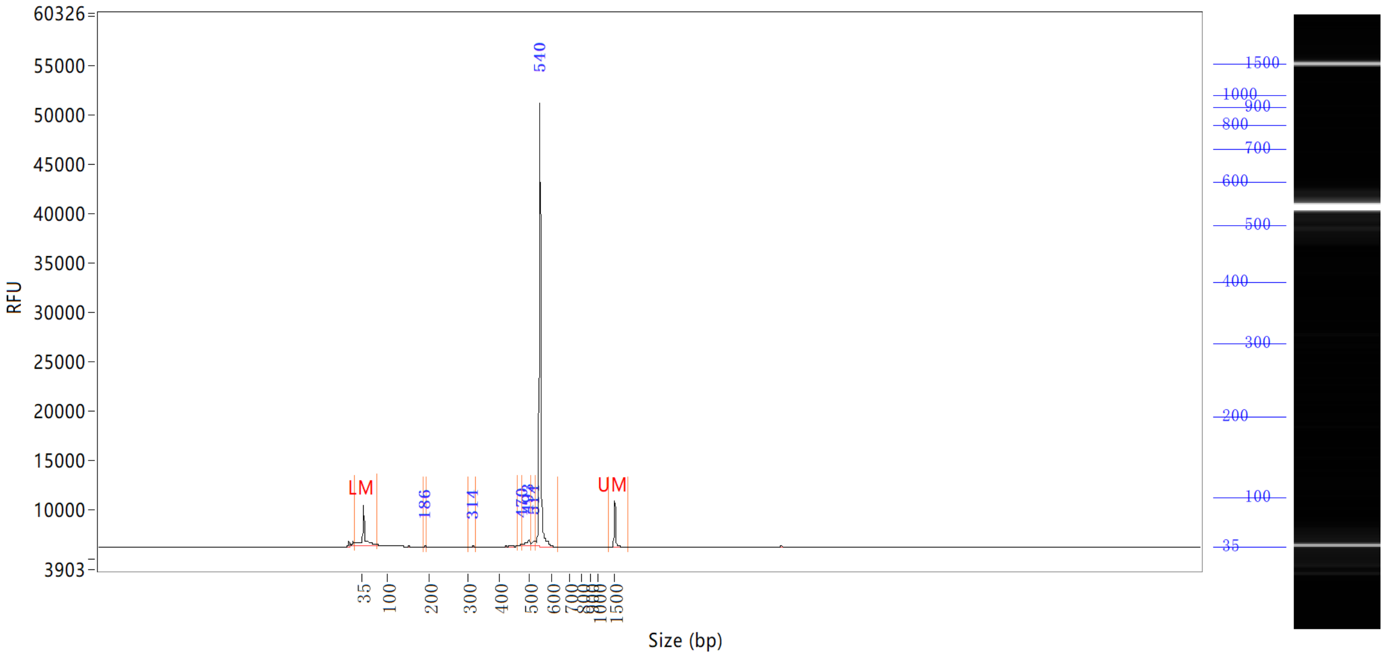

Peak	Size (bp)	Conc. (ng/uL)	From (bp)	To (bp)	Avg. Size (bp)	CV%	RFU	Corr. Peak Area
1	35 (LM)	1.2028	16	74	36	26.60	4163	42.155
2	186	0.0930	183	190	186	0.77	67	0.272
3	314	0.2534	300	324	312	1.24	144	0.740
4	470	0.4899	457	474	469	0.73	213	1.431
5	493	2.3214	474	501	489	1.49	614	6.780
6	514	1.8949	501	525	513	1.31	511	5.534
7	540	74.2518	525	633	541	1.38	45046	216.867
8	1500 (UM)	0.5000	1314	1908	1508	3.81	4764	17.524

TIC:	79.3044	ng/uL
TIM:	243.6197	nmole/L
Total Conc.:	80.7174	ng/uL

```
Sample Peak Width (sec): 5    Sample Min Peak Height: 50    Sample Baseline V to V?: Y    Sample Baseline V to V pts: 3
Sample Filter: Binomial       # of Pts for Filter: 3        Sample Start Region (min): 0  Sample End Region (min): 80
Marker Peak Width (sec): 5    Marker Min Peak Height: 500   Marker Baseline V to V?: Y    Marker Baseline V to V pts: 3
Lower Marker Selection: First Peak > 500 RFU                Upper Marker Selection: Last Peak > 500 RFU
Ladder Size (bp)35, 100, 200, 300, 400, 500, 600, 700, 800, 900, 1000, 1500
Quantification Using: Upper Marker          Final Concentration (ng/uL): 0.5000          Dilution Factor: 12.0
```

 Copyright 2015 Advanced Analytical Technologies, Inc.

Sample: SampC2
Well Location: C2

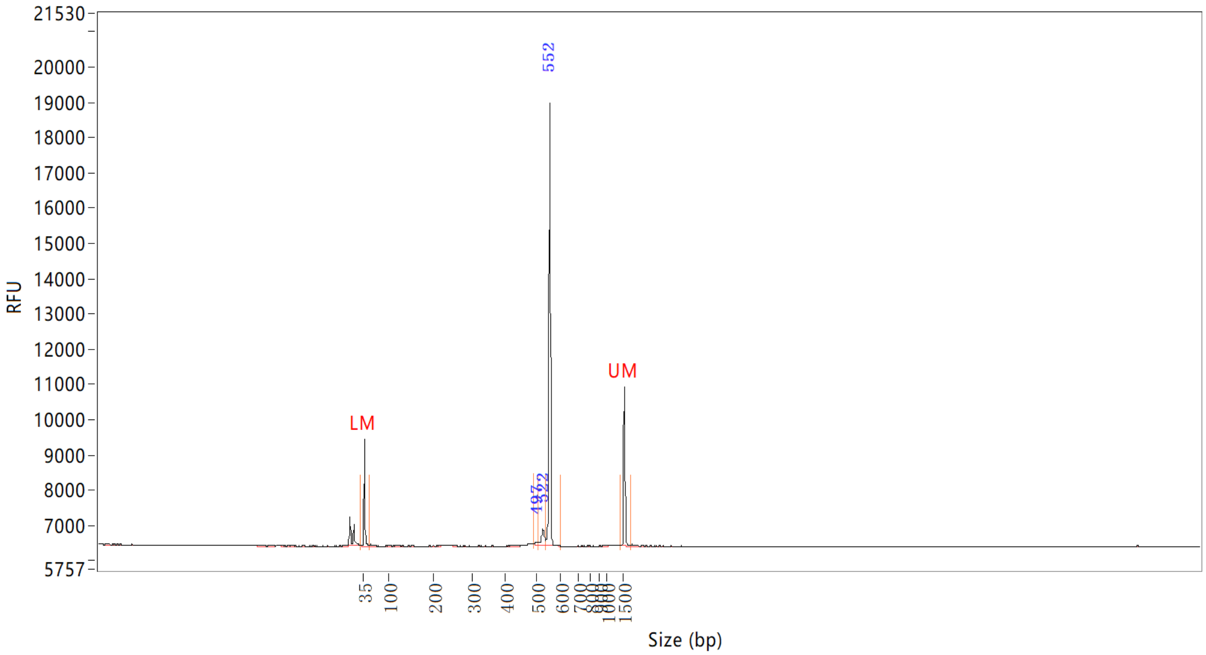

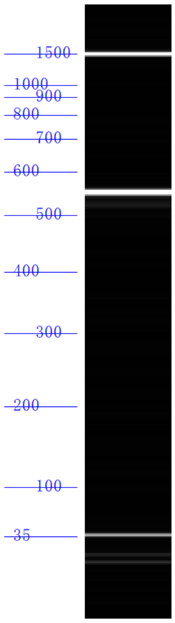

Peak	Size (bp)	Conc. (ng/uL)	From (bp)	To (bp)	Avg. Size (bp)	CV%	RFU	Corr. Peak Area
1	35 (LM)	0.4925	29	49	35	6.80	3045	14.821
2	497	0.2780	489	508	499	0.99	83	0.697
3	522	1.3920	508	534	522	1.13	461	3.491
4	552	19.4169	534	606	551	0.74	12576	48.697
5	1500 (UM)	0.5000	1387	1691	1492	1.95	4499	15.048

TIC:	21.0869	ng/uL
TIM:	63.2942	nmole/L
Total Conc.:	22.8488	ng/uL

```
Sample Peak Width (sec): 5     Sample Min Peak Height: 50    Sample Baseline V to V?: Y     Sample Baseline V to V pts: 3
Sample Filter: Binomial        # of Pts for Filter: 3        Sample Start Region (min): 0  Sample End Region (min): 80
Marker Peak Width (sec): 5     Marker Min Peak Height: 500   Marker Baseline V to V?: Y     Marker Baseline V to V pts: 3
Lower Marker Selection: First Peak > 500 RFU                 Upper Marker Selection: Last Peak > 500 RFU
Ladder Size (bp)35, 100, 200, 300, 400, 500, 600, 700, 800, 900, 1000, 1500
Quantification Using: Upper Marker          Final Concentration (ng/uL): 0.5000          Dilution Factor: 12.0
```

Sample: SampD2
Well Location: D2

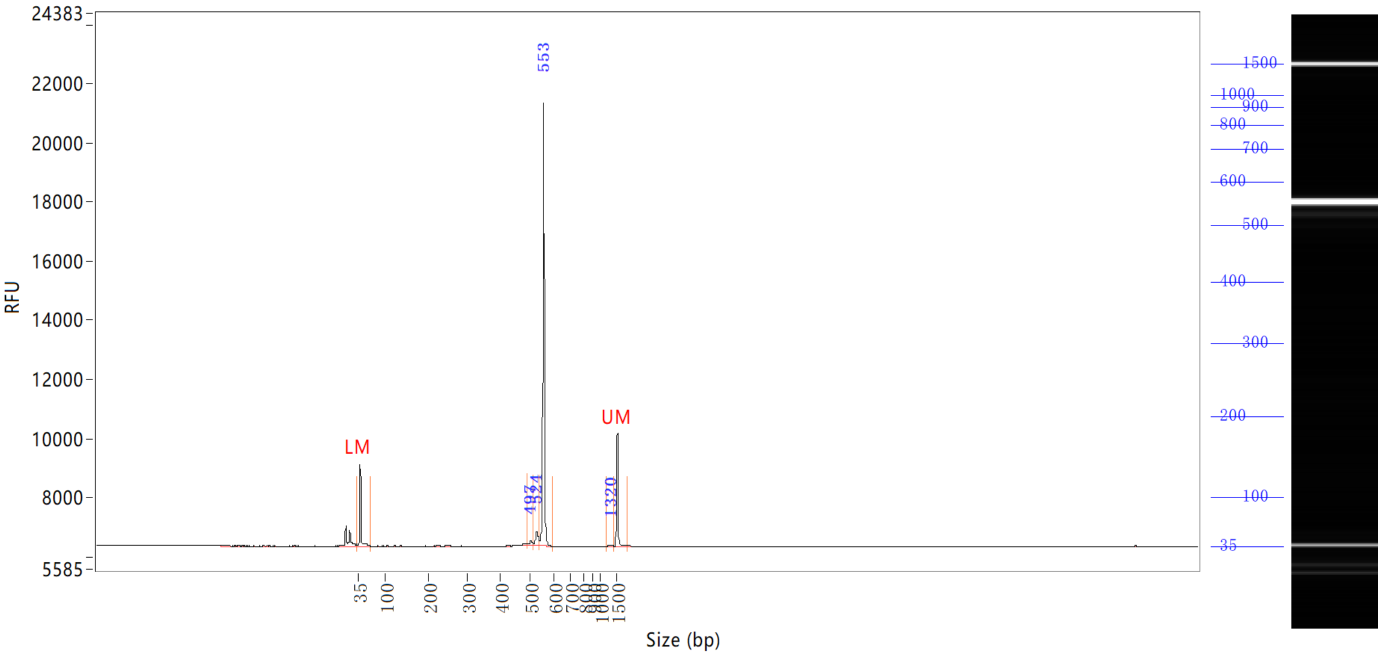

Peak	Size (bp)	Conc. (ng/uL)	From (bp)	To (bp)	Avg. Size (bp)	CV%	RFU	Corr. Peak Area
1	35 (LM)	0.5892	29	64	36	13.24	2767	15.338
2	497	0.4030	490	508	498	0.94	119	0.874
3	524	1.7080	508	536	523	1.19	474	3.705
4	553	27.6044	536	598	552	0.79	14999	59.883
5	1320	0.2132	1187	1420	1306	3.99	59	0.463
6	1500 (UM)	0.5000	1420	1809	1499	2.87	3836	13.016

TIC:	29.9287	ng/uL
TIM:	89.2669	nmole/L
Total Conc.:	32.1228	ng/uL

Sample Peak Width (sec): 5 Sample Min Peak Height: 50 Sample Baseline V to V?: Y Sample Baseline V to V pts: 3
Sample Filter: Binomial # of Pts for Filter: 3 Sample Start Region (min): 0 Sample End Region (min): 80
Marker Peak Width (sec): 5 Marker Min Peak Height: 500 Marker Baseline V to V?: Y Marker Baseline V to V pts: 3
Lower Marker Selection: First Peak > 500 RFU Upper Marker Selection: Last Peak > 500 RFU
Ladder Size (bp)35, 100, 200, 300, 400, 500, 600, 700, 800, 900, 1000, 1500
Quantification Using: Upper Marker Final Concentration (ng/uL): 0.5000 Dilution Factor: 12.0

 Copyright 2015 Advanced Analytical Technologies, Inc.

Sample: SampE2
Well Location: E2

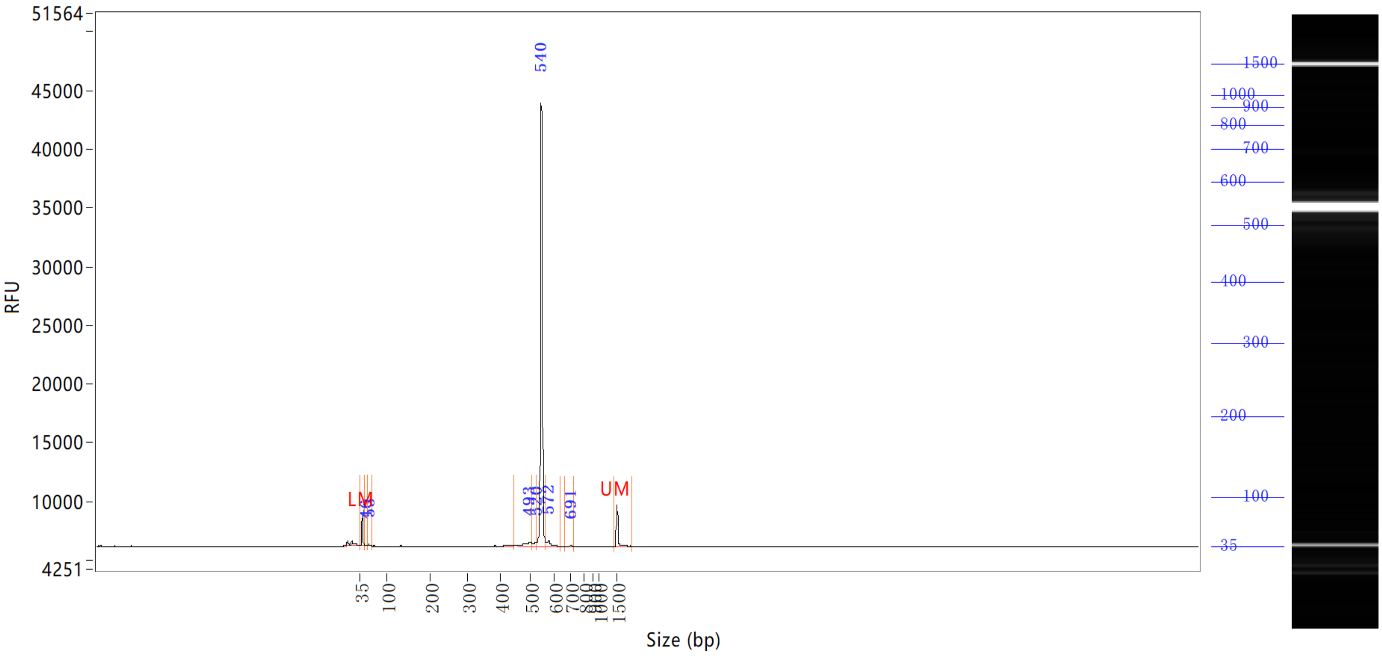

Peak	Size (bp)	Conc. (ng/uL)	From (bp)	To (bp)	Avg. Size (bp)	CV%	RFU	Corr. Peak Area
1	35 (LM)	0.4984	30	44	35	5.73	2599	13.065
2	46	0.1893	44	49	47	2.98	62	0.414
3	53	0.3609	49	61	52	3.82	119	0.788
4	493	2.7041	443	503	483	2.68	337	5.907
5	520	1.5619	503	524	514	1.13	372	3.412
6	540	82.1053	524	563	540	0.85	37784	179.345
7	572	1.8967	563	638	576	2.02	450	4.143
8	691	0.1627	664	712	690	1.00	58	0.355
9	1500 (UM)	0.5000	1420	1941	1505	3.77	3561	13.106

TIC:	88.9808	ng/uL
TIM:	288.1869	nmole/L
Total Conc.:	90.8706	ng/uL

```
Sample Peak Width (sec): 5     Sample Min Peak Height: 50    Sample Baseline V to V?: Y    Sample Baseline V to V pts: 3
Sample Filter: Binomial        # of Pts for Filter: 3        Sample Start Region (min): 0  Sample End Region (min): 80
Marker Peak Width (sec): 5     Marker Min Peak Height: 500   Marker Baseline V to V?: Y    Marker Baseline V to V pts: 3
Lower Marker Selection: First Peak > 500 RFU                 Upper Marker Selection: Last Peak > 500 RFU
Ladder Size (bp)35, 100, 200, 300, 400, 500, 600, 700, 800, 900, 1000, 1500
Quantification Using: Upper Marker          Final Concentration (ng/uL): 0.5000          Dilution Factor: 12.0
```

 Copyright 2015 Advanced Analytical Technologies, Inc.

Sample: SampF2
Well Location: F2

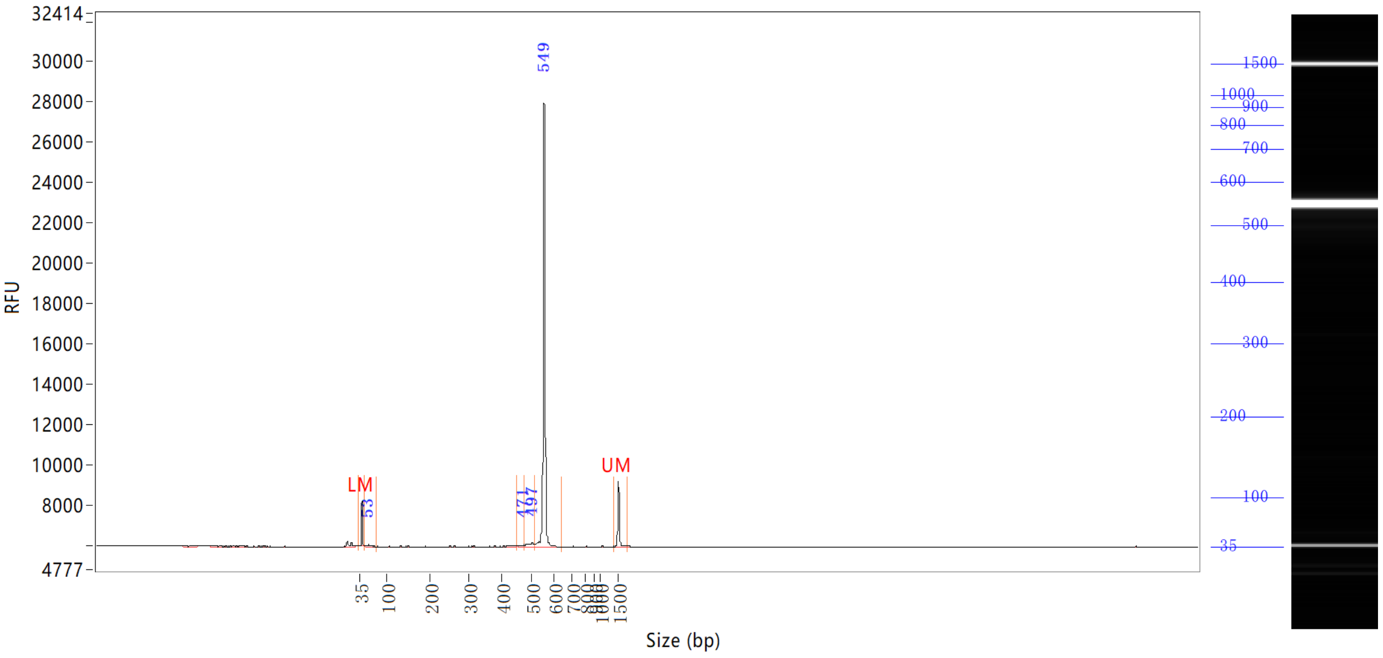

Peak	Size (bp)	Conc. (ng/uL)	From (bp)	To (bp)	Avg. Size (bp)	CV%	RFU	Corr. Peak Area
1	35 (LM)	0.4743	30	44	35	5.34	2294	11.088
2	53	0.6444	44	72	52	10.82	92	1.255
3	471	0.3930	447	474	465	1.49	79	0.766
4	497	1.3973	474	509	492	1.85	175	2.722
5	549	55.0128	509	638	548	1.26	22073	107.172
6	1500 (UM)	0.5000	1380	1757	1501	2.70	3280	11.689

TIC:	57.4475	ng/uL
TIM:	191.5611	nmole/L
Total Conc.:	59.5474	ng/uL

```
Sample Peak Width (sec): 5     Sample Min Peak Height: 50    Sample Baseline V to V?: Y     Sample Baseline V to V pts: 3
Sample Filter: Binomial        # of Pts for Filter: 3        Sample Start Region (min): 0   Sample End Region (min): 80
Marker Peak Width (sec): 5     Marker Min Peak Height: 500   Marker Baseline V to V?: Y     Marker Baseline V to V pts: 3
Lower Marker Selection: First Peak > 500 RFU                 Upper Marker Selection: Last Peak > 500 RFU
Ladder Size (bp)35, 100, 200, 300, 400, 500, 600, 700, 800, 900, 1000, 1500
Quantification Using: Upper Marker          Final Concentration (ng/uL): 0.5000          Dilution Factor: 12.0
```

 Copyright 2015 Advanced Analytical Technologies, Inc.

Sample: SampG2
Well Location: G2

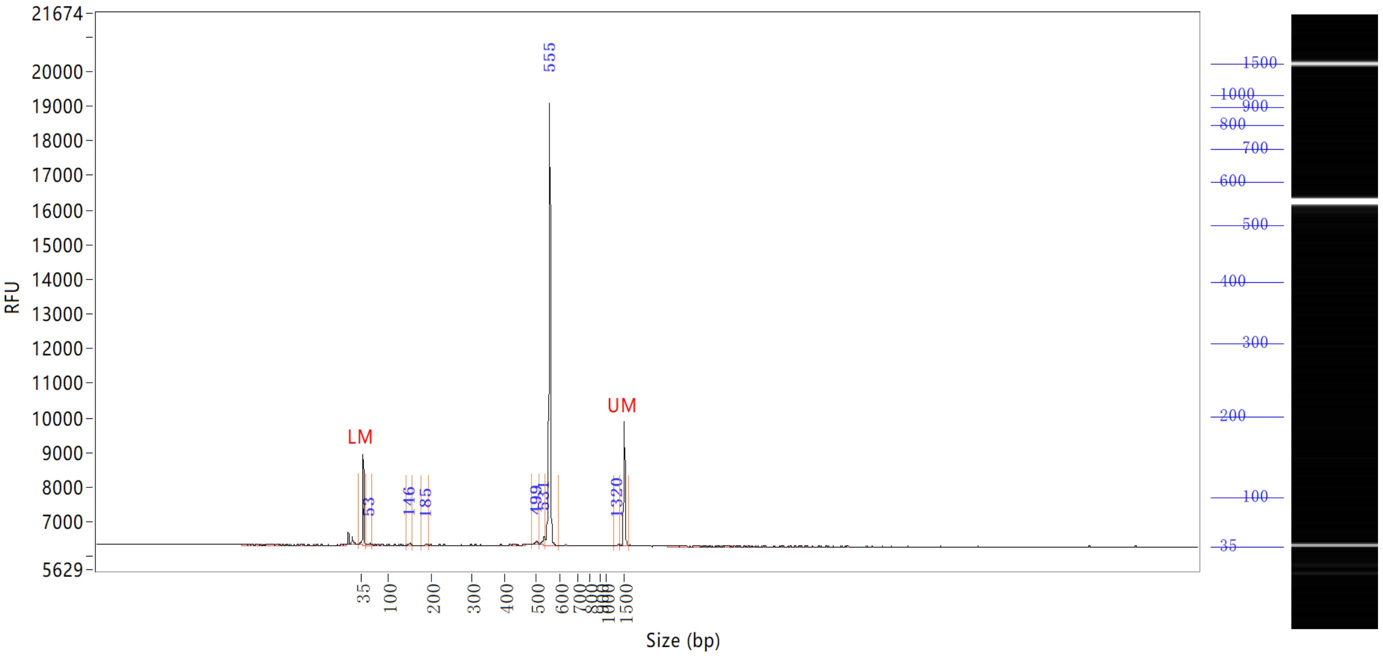

Peak	Size (bp)	Conc. (ng/uL)	From (bp)	To (bp)	Avg. Size (bp)	CV%	RFU	Corr. Peak Area
1	35 (LM)	0.5328	25	45	35	5.71	2631	13.043
2	53	0.2790	45	59	51	6.14	56	0.569
3	146	0.2195	139	154	146	1.90	55	0.448
4	185	0.2504	173	190	183	2.05	52	0.511
5	499	0.3199	486	509	497	1.14	79	0.653
6	531	0.8767	509	537	527	1.35	237	1.789
7	555	27.6256	537	595	553	0.79	12775	56.359
8	1320	0.1264	1234	1367	1315	2.25	53	0.258
9	1500 (UM)	0.5000	1367	1638	1495	1.77	3595	12.241

TIC:	29.6975	ng/uL
TIM:	99.8421	nmole/L
Total Conc.:	32.0188	ng/uL

```
Sample Peak Width (sec): 5    Sample Min Peak Height: 50    Sample Baseline V to V?: Y    Sample Baseline V to V pts: 3
Sample Filter: Binomial       # of Pts for Filter: 3        Sample Start Region (min): 0  Sample End Region (min): 80
Marker Peak Width (sec): 5    Marker Min Peak Height: 500   Marker Baseline V to V?: Y    Marker Baseline V to V pts: 3
Lower Marker Selection: First Peak > 500 RFU                Upper Marker Selection: Last Peak > 500 RFU
Ladder Size (bp)35, 100, 200, 300, 400, 500, 600, 700, 800, 900, 1000, 1500
Quantification Using: Upper Marker          Final Concentration (ng/uL): 0.5000          Dilution Factor: 12.0
```

 Copyright 2015 Advanced Analytical Technologies, Inc.

Sample: SampH2
Well Location: H2

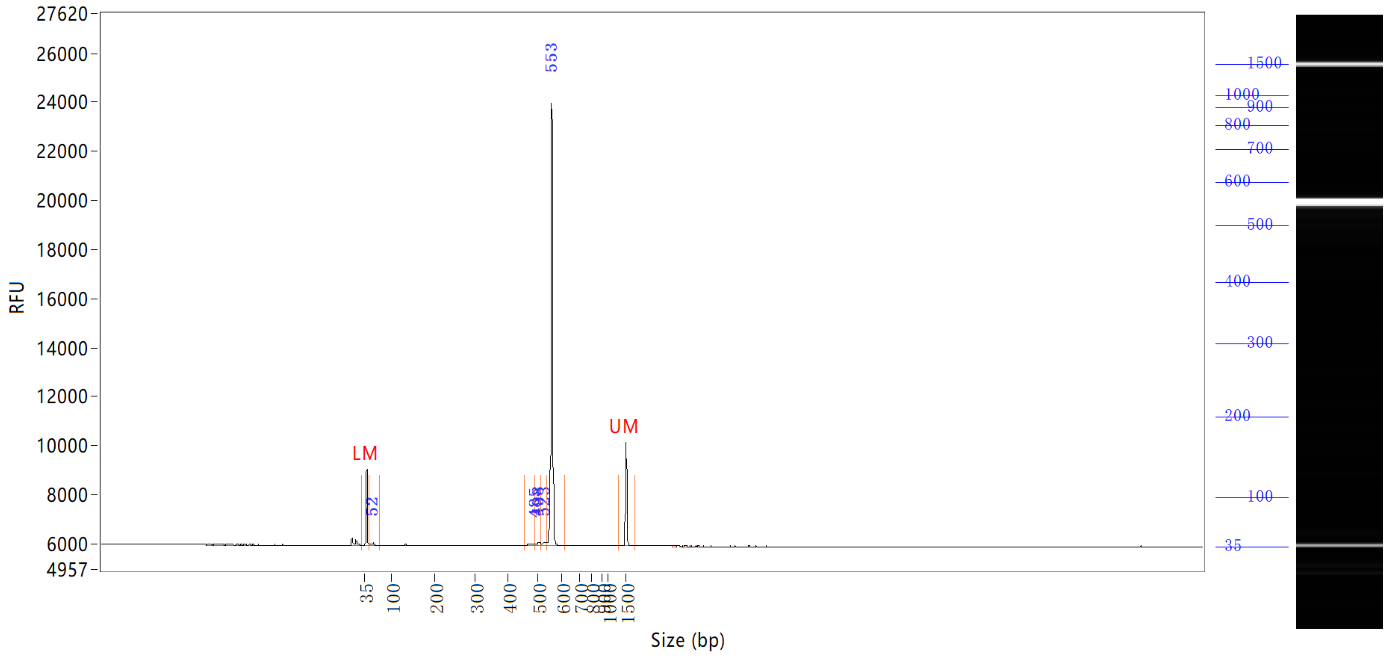

Peak	Size (bp)	Conc. (ng/uL)	From (bp)	To (bp)	Avg. Size (bp)	CV%	RFU	Corr. Peak Area
1	35 (LM)	0.4976	26	42	34	4.24	3088	14.481
2	52	0.4416	42	71	51	9.87	91	1.071
3	485	0.3870	454	488	476	1.77	64	0.938
4	498	0.4934	488	510	498	1.15	120	1.197
5	523	0.5135	510	531	522	1.17	126	1.245
6	553	33.1744	531	616	552	0.85	18061	80.450
7	1500 (UM)	0.5000	1267	1770	1492	2.30	4238	14.550

TIC:	35.0098	ng/uL
TIM:	117.6675	nmole/L
Total Conc.:	36.3848	ng/uL

Sample Peak Width (sec): 5   Sample Min Peak Height: 50   Sample Baseline V to V?: Y   Sample Baseline V to V pts: 3
Sample Filter: Binomial   # of Pts for Filter: 3   Sample Start Region (min): 0   Sample End Region (min): 80
Marker Peak Width (sec): 5   Marker Min Peak Height: 500   Marker Baseline V to V?: Y   Marker Baseline V to V pts: 3
Lower Marker Selection: First Peak > 500 RFU   Upper Marker Selection: Last Peak > 500 RFU
Ladder Size (bp)35, 100, 200, 300, 400, 500, 600, 700, 800, 900, 1000, 1500
Quantification Using: Upper Marker   Final Concentration (ng/uL): 0.5000   Dilution Factor: 12.0

Copyright 2015 Advanced Analytical Technologies, Inc.

Sample: SampA3
Well Location: A3

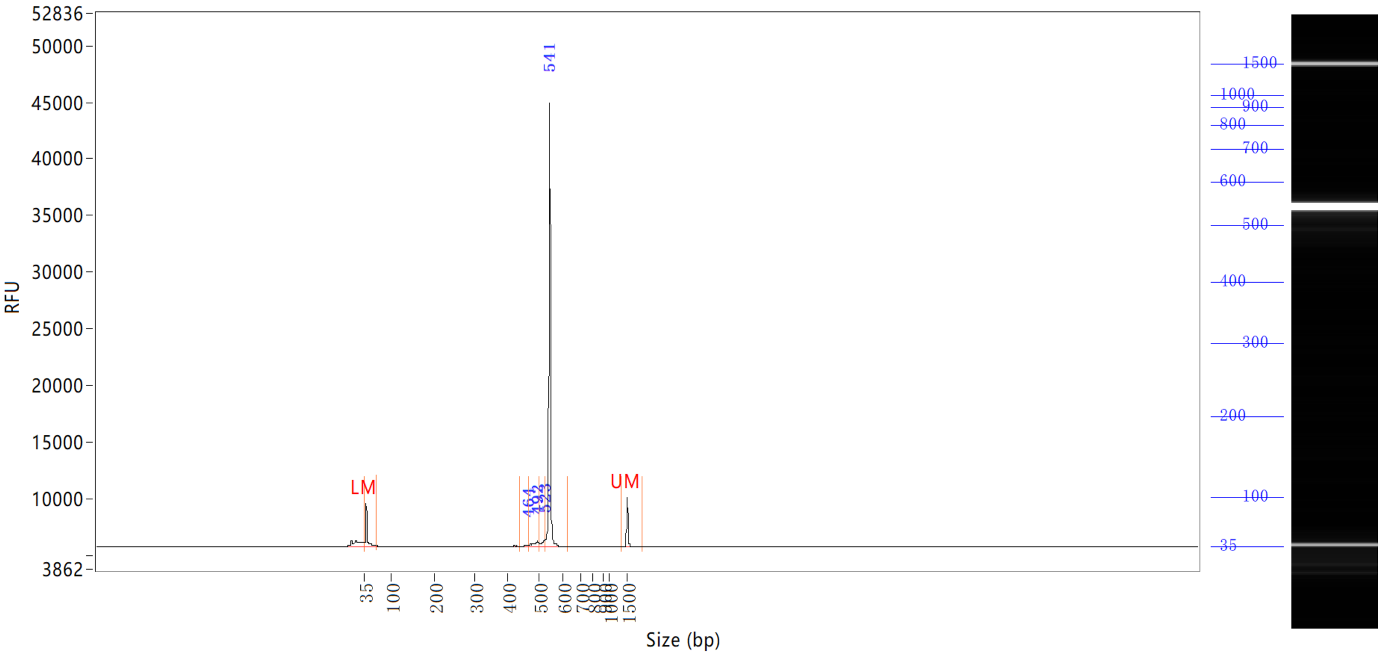

Peak	Size (bp)	Conc. (ng/uL)	From (bp)	To (bp)	Avg. Size (bp)	CV%	RFU	Corr. Peak Area
1	35 (LM)	0.7740	31	63	37	13.14	3742	23.678
2	464	0.3481	439	465	458	1.34	145	0.887
3	492	2.4513	465	500	485	2.00	441	6.249
4	523	2.1928	500	526	515	1.43	650	5.590
5	541	68.4864	526	628	541	0.99	39140	174.590
6	1500 (UM)	0.5000	1327	1954	1501	2.99	4334	15.296

TIC:	73.4786	ng/uL
TIM:	224.8901	nmole/L
Total Conc.:	75.1542	ng/uL

Sample Peak Width (sec): 5   Sample Min Peak Height: 50   Sample Baseline V to V?: Y   Sample Baseline V to V pts: 3
Sample Filter: Binomial   # of Pts for Filter: 3   Sample Start Region (min): 0   Sample End Region (min): 80
Marker Peak Width (sec): 5   Marker Min Peak Height: 500   Marker Baseline V to V?: Y   Marker Baseline V to V pts: 3
Lower Marker Selection: First Peak > 500 RFU   Upper Marker Selection: Last Peak > 500 RFU
Ladder Size (bp)35, 100, 200, 300, 400, 500, 600, 700, 800, 900, 1000, 1500
Quantification Using: Upper Marker   Final Concentration (ng/uL): 0.5000   Dilution Factor: 12.0

 Copyright 2015 Advanced Analytical Technologies, Inc.

Sample: SampB3
Well Location: B3

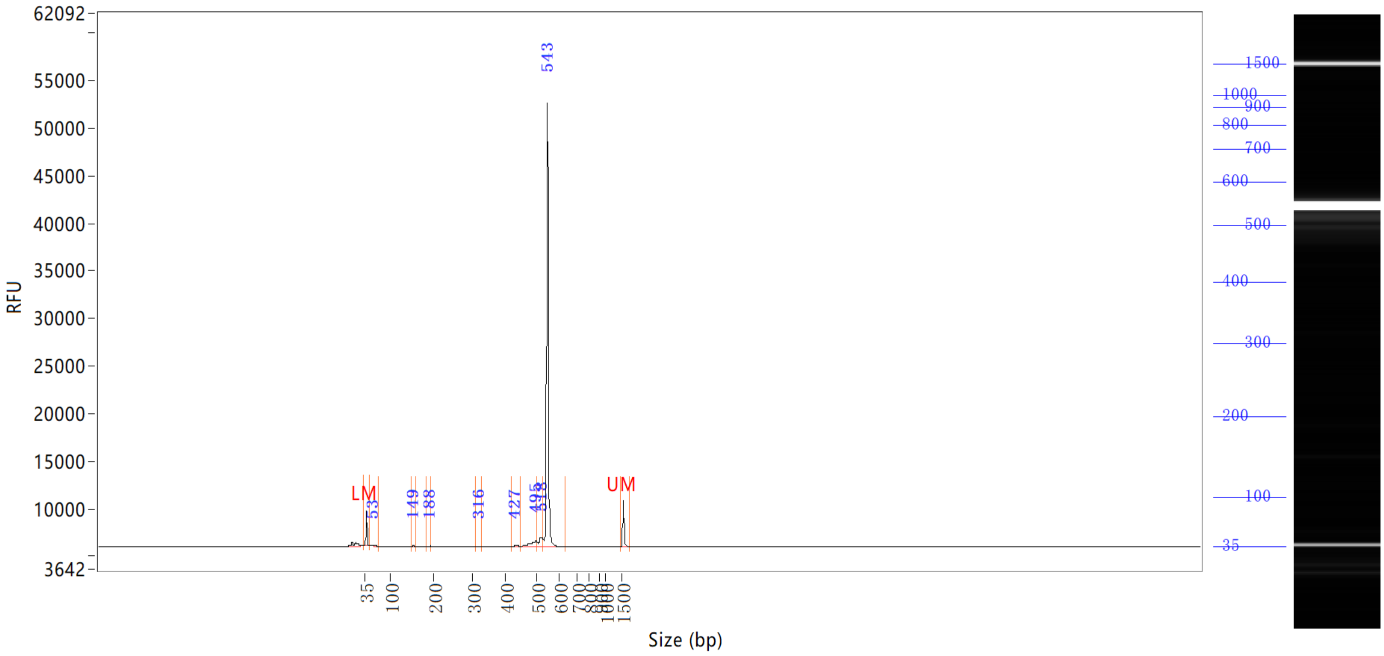

Peak	Size (bp)	Conc. (ng/uL)	From (bp)	To (bp)	Avg. Size (bp)	CV%	RFU	Corr. Peak Area
1	35 (LM)	0.5115	31	44	35	4.48	3624	17.171
2	53	0.3538	44	68	52	8.20	92	0.990
3	149	0.3205	144	158	149	1.63	150	0.897
4	188	0.1451	181	191	186	1.11	68	0.406
5	316	0.1445	305	324	314	1.09	73	0.404
6	427	0.2140	418	447	431	1.68	61	0.599
7	495	3.7106	447	503	484	2.58	655	10.380
8	518	3.6058	503	528	516	1.33	955	10.087
9	543	79.3264	528	629	543	1.01	46707	221.906
10	1500 (UM)	0.5000	1433	1717	1501	2.07	4859	16.784

TIC:	87.8208	ng/uL
TIM:	282.0526	nmole/L
Total Conc.:	89.3746	ng/uL

Sample Peak Width (sec): 5 Sample Min Peak Height: 50 Sample Baseline V to V?: Y Sample Baseline V to V pts: 3
Sample Filter: Binomial # of Pts for Filter: 3 Sample Start Region (min): 0 Sample End Region (min): 80
Marker Peak Width (sec): 5 Marker Min Peak Height: 500 Marker Baseline V to V?: Y Marker Baseline V to V pts: 3
Lower Marker Selection: First Peak > 500 RFU Upper Marker Selection: Last Peak > 500 RFU
Ladder Size (bp)35, 100, 200, 300, 400, 500, 600, 700, 800, 900, 1000, 1500
Quantification Using: Upper Marker Final Concentration (ng/uL): 0.5000 Dilution Factor: 12.0

Sample: SampC3
Well Location: C3

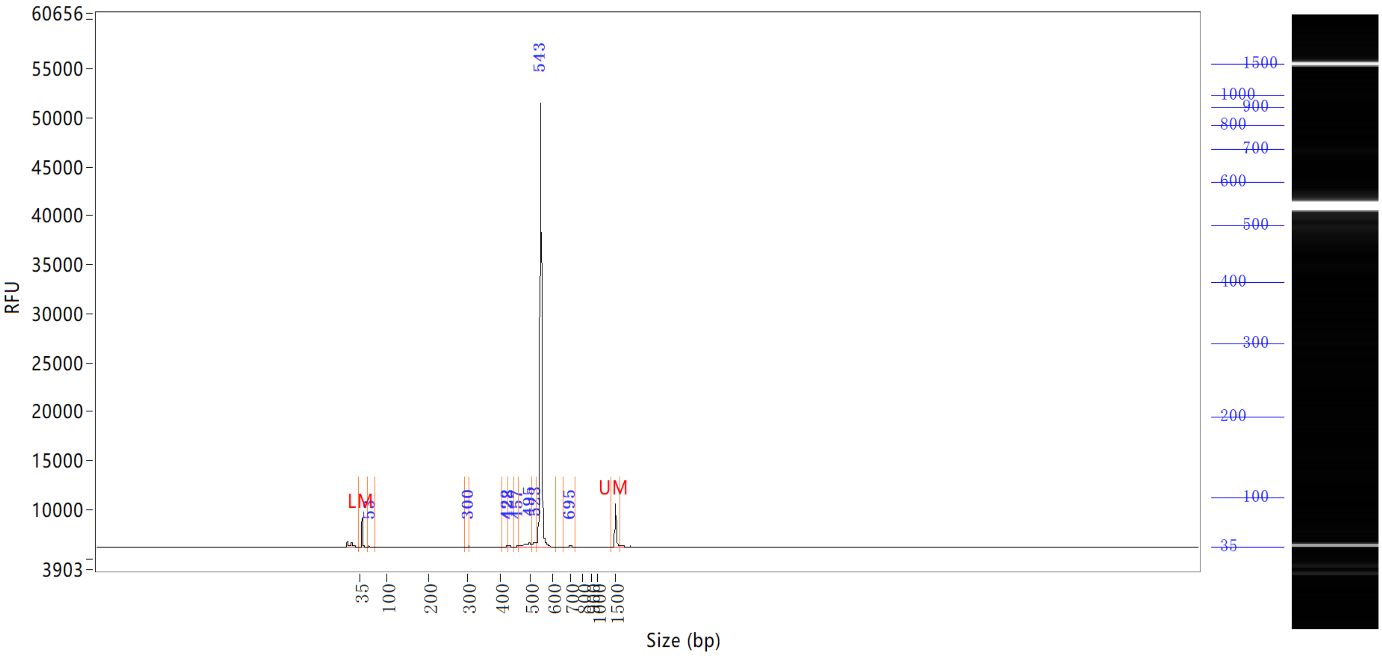

Peak	Size (bp)	Conc. (ng/uL)	From (bp)	To (bp)	Avg. Size (bp)	CV%	RFU	Corr. Peak Area
1	35 (LM)	0.4779	28	50	35	7.76	3052	15.413
2	53	0.2856	50	69	55	7.20	80	0.768
3	300	0.1196	292	306	299	0.84	71	0.322
4	422	0.1477	405	425	419	1.11	59	0.397
5	428	0.1868	425	445	431	1.04	74	0.502
6	457	0.1705	445	460	455	0.75	84	0.458
7	495	2.5401	460	505	486	2.34	413	6.826
8	523	1.6197	505	526	517	1.18	456	4.353
9	543	85.7066	526	619	543	1.09	45342	230.329
10	695	0.3893	661	741	693	1.85	128	1.046
11	1500 (UM)	0.5000	1394	1665	1499	2.15	4452	16.124

TIC:	91.1659	ng/uL
TIM:	285.4923	nmole/L
Total Conc.:	92.8220	ng/uL

```
Sample Peak Width (sec): 5     Sample Min Peak Height: 50    Sample Baseline V to V?: Y    Sample Baseline V to V pts: 3
Sample Filter: Binomial        # of Pts for Filter: 3        Sample Start Region (min): 0  Sample End Region (min): 80
Marker Peak Width (sec): 5     Marker Min Peak Height: 500   Marker Baseline V to V?: Y    Marker Baseline V to V pts: 3
Lower Marker Selection: First Peak > 500 RFU                 Upper Marker Selection: Last Peak > 500 RFU
Ladder Size (bp)35, 100, 200, 300, 400, 500, 600, 700, 800, 900, 1000, 1500
Quantification Using: Upper Marker          Final Concentration (ng/uL): 0.5000          Dilution Factor: 12.0
```

 Copyright 2015 Advanced Analytical Technologies, Inc.

Sample: SampD3
Well Location: D3

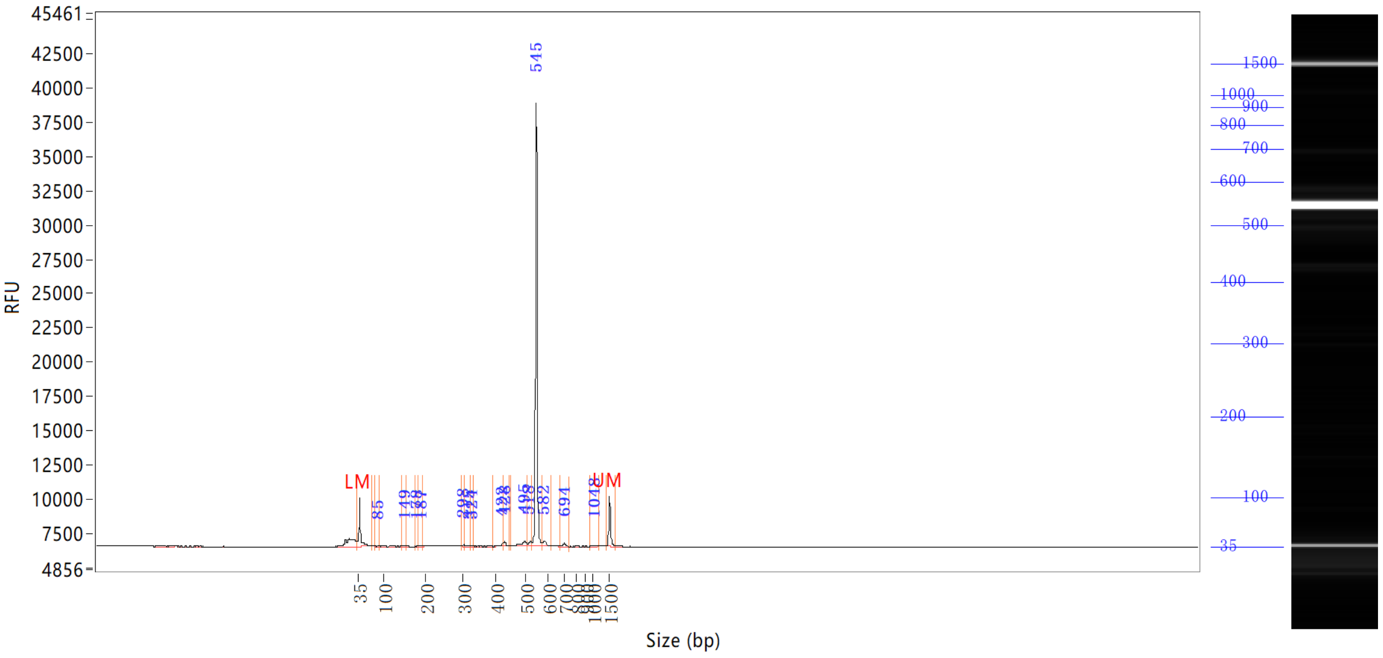

Peak	Size (bp)	Conc. (ng/uL)	From (bp)	To (bp)	Avg. Size (bp)	CV%	RFU	Corr. Peak Area
1	35 (LM)	0.8030	31	67	37	16.11	3542	23.675
2	85	0.1974	78	88	83	2.32	67	0.485
3	149	0.2385	142	153	148	1.57	97	0.586
4	178	0.1033	174	180	178	0.77	55	0.254
5	187	0.2051	180	190	185	1.15	74	0.504
6	298	0.2348	294	303	297	0.58	129	0.577
7	315	0.2072	303	320	313	1.22	87	0.509
8	324	0.1599	320	332	324	0.76	71	0.393
9	422	0.6722	390	424	418	1.62	273	1.652
10	428	0.7466	424	444	429	0.74	333	1.834
11	495	2.3925	450	506	487	2.37	412	5.878
12	518	1.3609	506	529	519	1.27	339	3.343
13	545	60.5865	529	573	545	0.86	32401	148.851
14	582	1.4603	573	621	584	1.30	398	3.588
15	694	0.6341	675	736	692	1.24	252	1.558
16	1048	0.2686	968	1214	1048	4.42	106	0.660
17	1500 (UM)	0.5000	1400	1678	1501	2.23	3642	14.741

TIC:	69.4678	ng/uL
TIM:	219.4167	nmole/L
Total Conc.:	70.8704	ng/uL

Sample Peak Width (sec): 5 Sample Min Peak Height: 50 Sample Baseline V to V?: Y Sample Baseline V to V pts: 3
Sample Filter: Binomial # of Pts for Filter: 3 Sample Start Region (min): 0 Sample End Region (min): 80
Marker Peak Width (sec): 5 Marker Min Peak Height: 500 Marker Baseline V to V?: Y Marker Baseline V to V pts: 3
Lower Marker Selection: First Peak > 500 RFU Upper Marker Selection: Last Peak > 500 RFU
Ladder Size (bp)35, 100, 200, 300, 400, 500, 600, 700, 800, 900, 1000, 1500
Quantification Using: Upper Marker Final Concentration (ng/uL): 0.5000 Dilution Factor: 12.0

 Copyright 2015 Advanced Analytical Technologies, Inc.

Sample: SampE3
Well Location: E3

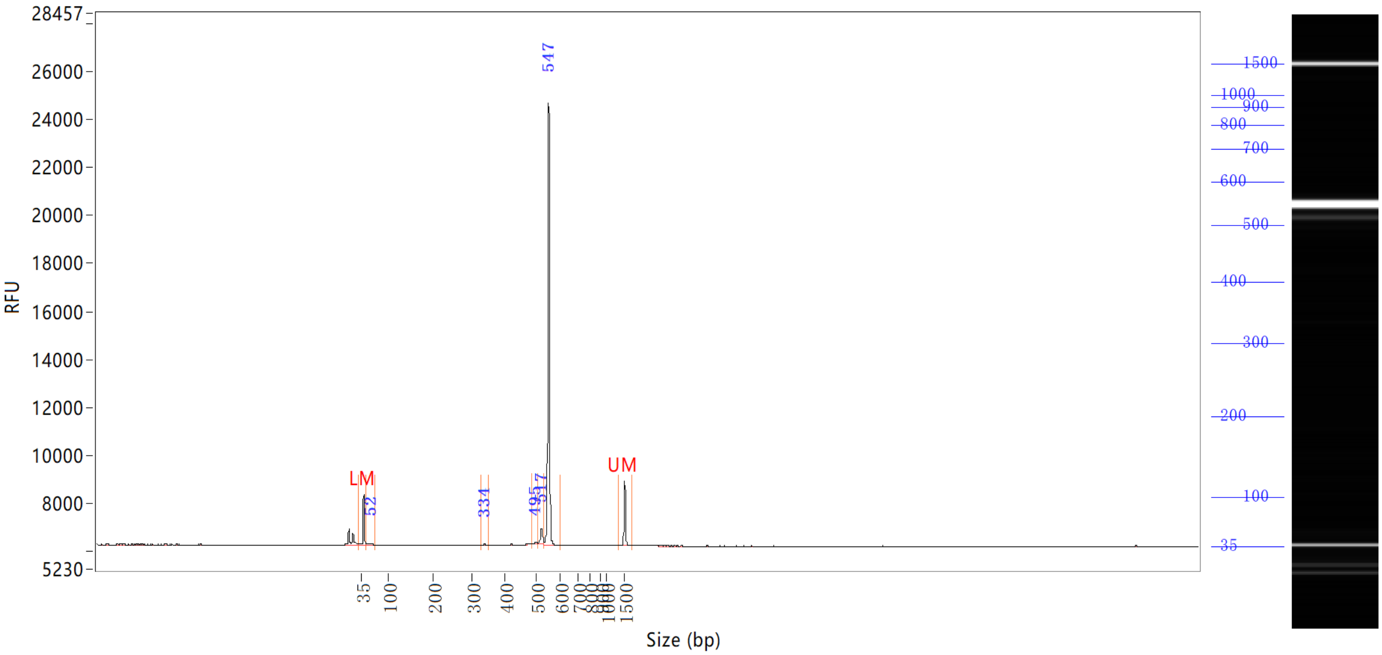

Peak	Size (bp)	Conc. (ng/uL)	From (bp)	To (bp)	Avg. Size (bp)	CV%	RFU	Corr. Peak Area
1	35 (LM)	0.5639	26	43	34	6.34	2088	10.572
2	52	0.8803	43	67	51	9.97	82	1.375
3	334	0.1499	324	346	334	1.12	53	0.234
4	495	0.3639	486	502	495	0.79	79	0.569
5	517	2.5970	502	529	516	1.09	633	4.057
6	547	49.8454	529	601	547	0.81	18493	77.878
7	1500 (UM)	0.5000	1327	1737	1497	2.29	2720	9.374

TIC:	53.8364	ng/uL
TIM:	188.4562	nmole/L
Total Conc.:	56.7353	ng/uL

Sample Peak Width (sec): 5   Sample Min Peak Height: 50   Sample Baseline V to V?: Y   Sample Baseline V to V pts: 3
Sample Filter: Binomial   # of Pts for Filter: 3   Sample Start Region (min): 0   Sample End Region (min): 80
Marker Peak Width (sec): 5   Marker Min Peak Height: 500   Marker Baseline V to V?: Y   Marker Baseline V to V pts: 3
Lower Marker Selection: First Peak > 500 RFU   Upper Marker Selection: Last Peak > 500 RFU
Ladder Size (bp)35, 100, 200, 300, 400, 500, 600, 700, 800, 900, 1000, 1500
Quantification Using: Upper Marker   Final Concentration (ng/uL): 0.5000   Dilution Factor: 12.0

 Copyright 2015 Advanced Analytical Technologies, Inc.

Sample: SampF3
Well Location: F3

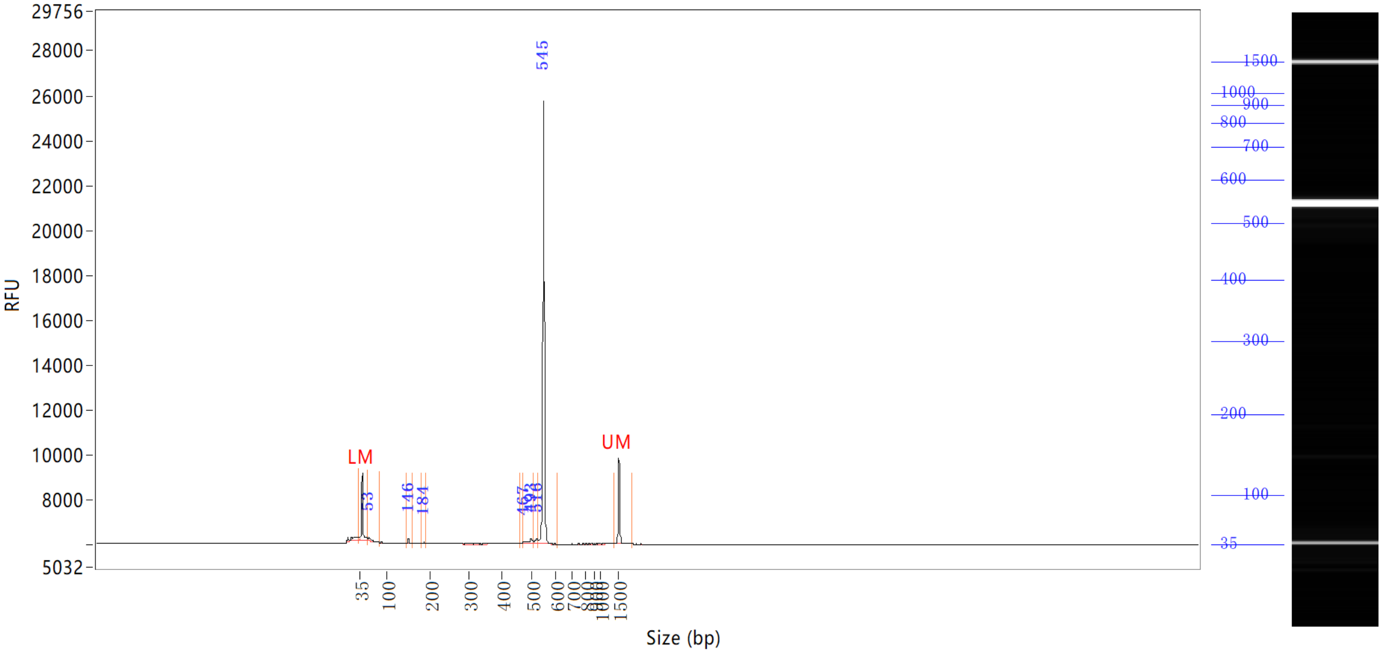

Peak	Size (bp)	Conc. (ng/uL)	From (bp)	To (bp)	Avg. Size (bp)	CV%	RFU	Corr. Peak Area
1	35 (LM)	0.6579	29	50	36	10.72	2966	17.116
2	53	0.8565	50	79	57	10.42	151	1.857
3	146	0.4800	143	159	147	1.86	192	1.041
4	184	0.2019	178	187	182	1.02	65	0.438
5	467	0.1258	457	468	465	0.52	68	0.273
6	493	1.2677	468	504	487	1.98	195	2.749
7	516	0.8431	504	526	516	1.22	189	1.828
8	545	38.8488	526	611	544	0.85	19731	84.229
9	1500 (UM)	0.5000	1354	1882	1498	2.75	3796	13.009

TIC:	42.6238	ng/uL
TIM:	156.7494	nmole/L
Total Conc.:	44.4543	ng/uL

Sample Peak Width (sec): 5 Sample Min Peak Height: 50 Sample Baseline V to V?: Y Sample Baseline V to V pts: 3
Sample Filter: Binomial # of Pts for Filter: 3 Sample Start Region (min): 0 Sample End Region (min): 80
Marker Peak Width (sec): 5 Marker Min Peak Height: 500 Marker Baseline V to V?: Y Marker Baseline V to V pts: 3
Lower Marker Selection: First Peak > 500 RFU Upper Marker Selection: Last Peak > 500 RFU
Ladder Size (bp)35, 100, 200, 300, 400, 500, 600, 700, 800, 900, 1000, 1500
Quantification Using: Upper Marker Final Concentration (ng/uL): 0.5000 Dilution Factor: 12.0

 Copyright 2015 Advanced Analytical Technologies, Inc.

Sample: SampG3
Well Location: G3

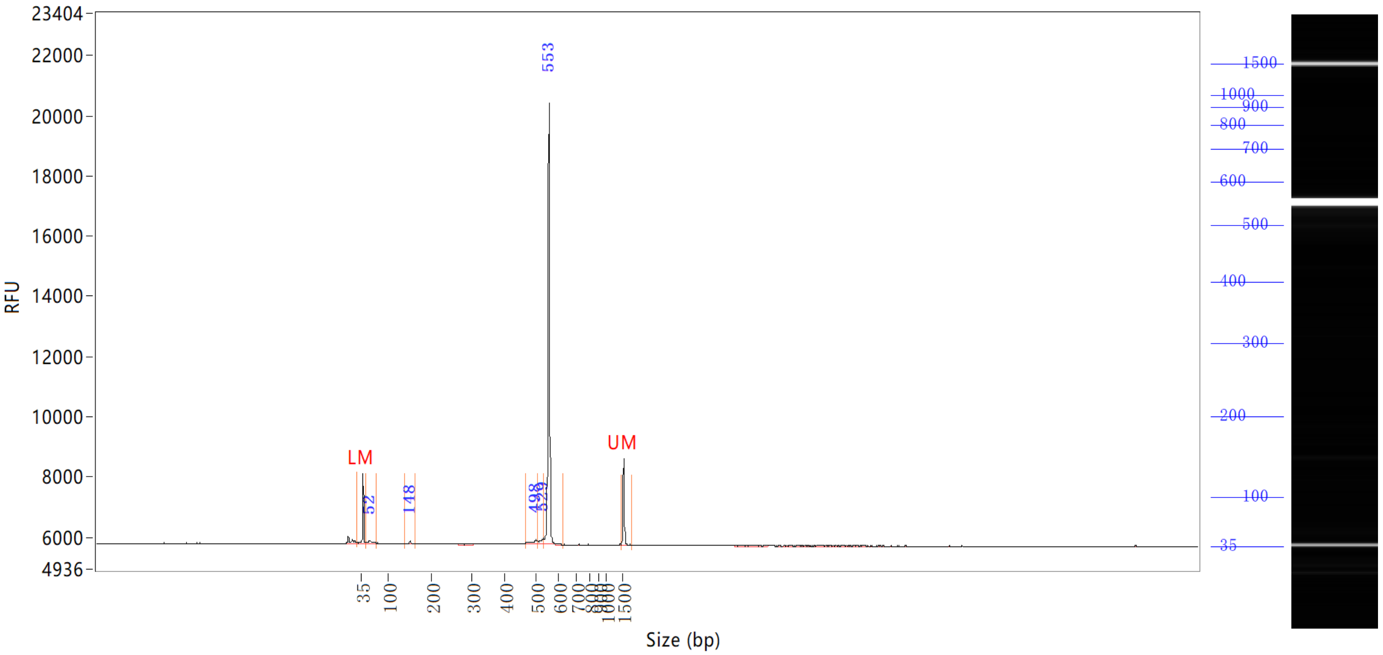

Peak	Size (bp)	Conc. (ng/uL)	From (bp)	To (bp)	Avg. Size (bp)	CV%	RFU	Corr. Peak Area
1	35 (LM)	0.5708	23	44	35	5.52	2325	11.144
2	52	0.9401	44	69	54	11.12	83	1.529
3	148	0.5206	135	161	146	2.77	114	0.847
4	498	1.2655	467	509	491	2.23	147	2.059
5	529	1.1233	509	533	523	1.34	203	1.827
6	553	39.7760	533	621	552	0.87	14688	64.710
7	1500 (UM)	0.5000	1433	1750	1498	2.11	2908	9.761

TIC:	43.6255	ng/uL
TIM:	160.7387	nmole/L
Total Conc.:	46.4817	ng/uL

```
Sample Peak Width (sec): 5    Sample Min Peak Height: 50    Sample Baseline V to V?: Y    Sample Baseline V to V pts: 3
Sample Filter: Binomial       # of Pts for Filter: 3        Sample Start Region (min): 0  Sample End Region (min): 80
Marker Peak Width (sec): 5    Marker Min Peak Height: 500   Marker Baseline V to V?: Y    Marker Baseline V to V pts: 3
Lower Marker Selection: First Peak > 500 RFU                Upper Marker Selection: Last Peak > 500 RFU
Ladder Size (bp)35, 100, 200, 300, 400, 500, 600, 700, 800, 900, 1000, 1500
Quantification Using: Upper Marker          Final Concentration (ng/uL): 0.5000          Dilution Factor: 12.0
```

 Copyright 2015 Advanced Analytical Technologies, Inc.

Sample: SampH3
Well Location: H3

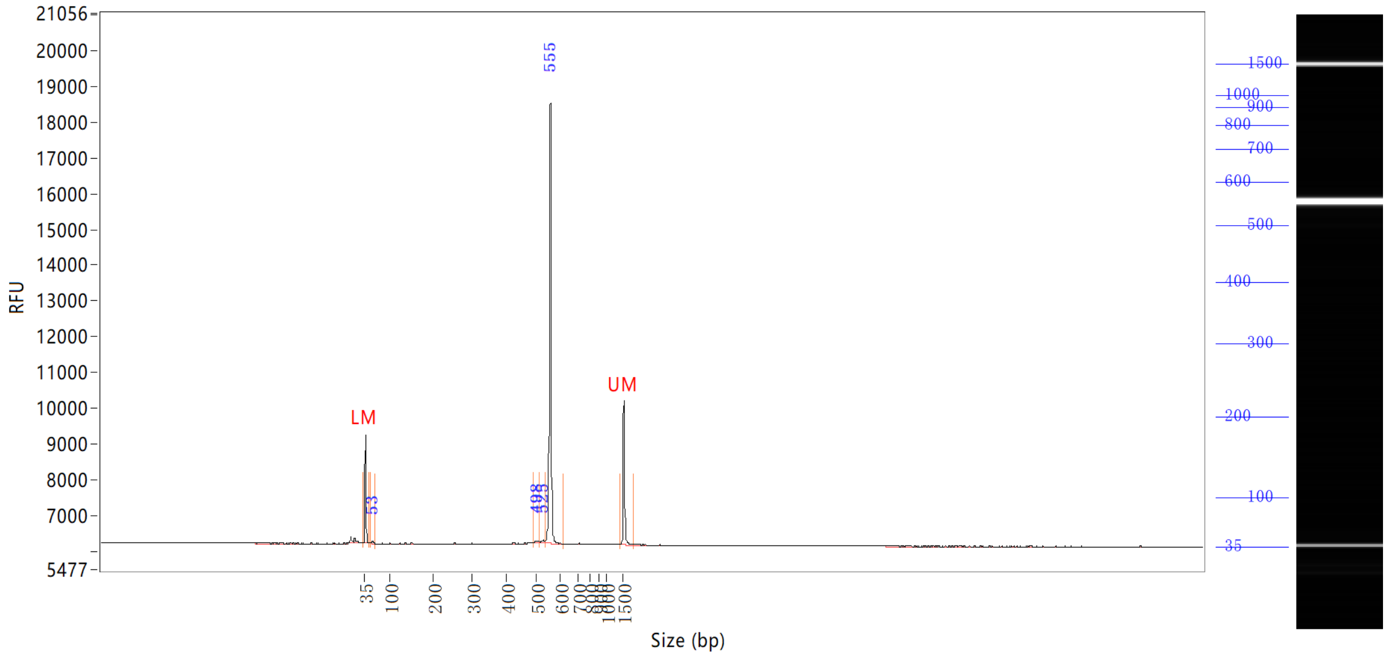

Peak	Size (bp)	Conc. (ng/uL)	From (bp)	To (bp)	Avg. Size (bp)	CV%	RFU	Corr. Peak Area
1	35 (LM)	0.5040	29	44	35	4.83	3007	14.163
2	53	0.1680	50	61	53	4.33	56	0.394
3	498	0.2734	489	511	499	1.11	72	0.640
4	525	0.3333	511	534	524	1.25	80	0.780
5	555	24.0180	534	620	554	0.91	12353	56.248
6	1500 (UM)	0.5000	1420	1776	1497	2.59	4036	14.051

TIC:	24.7926	ng/uL
TIM:	78.4848	nmole/L
Total Conc.:	26.9205	ng/uL

```
Sample Peak Width (sec): 5     Sample Min Peak Height: 50    Sample Baseline V to V?: Y     Sample Baseline V to V pts: 3
Sample Filter: Binomial        # of Pts for Filter: 3        Sample Start Region (min): 0   Sample End Region (min): 80
Marker Peak Width (sec): 5     Marker Min Peak Height: 500   Marker Baseline V to V?: Y     Marker Baseline V to V pts: 3
Lower Marker Selection: First Peak > 500 RFU                 Upper Marker Selection: Last Peak > 500 RFU
Ladder Size (bp)35, 100, 200, 300, 400, 500, 600, 700, 800, 900, 1000, 1500
Quantification Using: Upper Marker          Final Concentration (ng/uL): 0.5000          Dilution Factor: 12.0
```

 Copyright 2015 Advanced Analytical Technologies, Inc.

Sample: SampA4
Well Location: A4

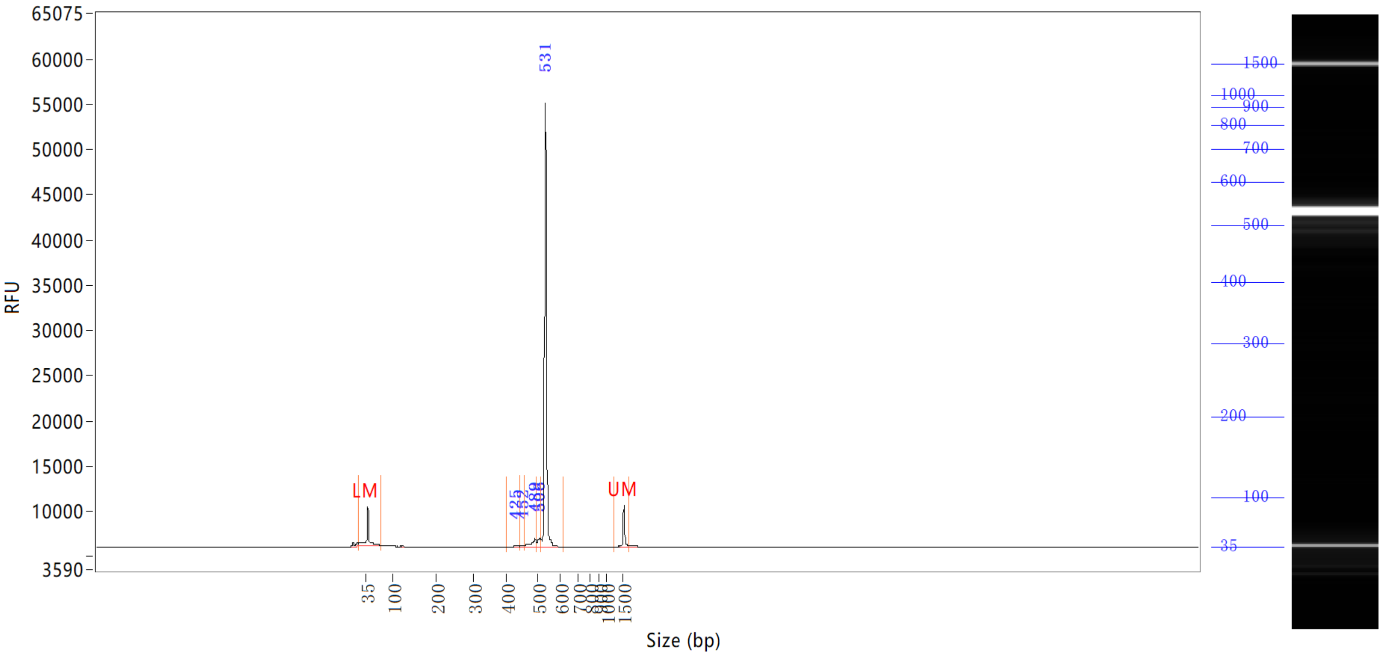

Peak	Size (bp)	Conc. (ng/uL)	From (bp)	To (bp)	Avg. Size (bp)	CV%	RFU	Corr. Peak Area
1	35 (LM)	1.1217	16	69	35	25.30	4290	39.109
2	425	0.2370	399	439	422	1.78	71	0.689
3	452	0.1883	439	455	450	0.84	80	0.547
4	489	3.8766	455	495	480	2.17	878	11.264
5	506	3.0524	495	516	505	1.20	909	8.869
6	531	90.9860	516	614	531	1.19	49124	264.368
7	1500 (UM)	0.5000	1227	1665	1498	2.88	4640	17.434

TIC:	98.3403	ng/uL
TIM:	306.8113	nmole/L
Total Conc.:	99.1389	ng/uL

```
Sample Peak Width (sec): 5     Sample Min Peak Height: 50    Sample Baseline V to V?: Y    Sample Baseline V to V pts: 3
Sample Filter: Binomial        # of Pts for Filter: 3        Sample Start Region (min): 0  Sample End Region (min): 80
Marker Peak Width (sec): 5     Marker Min Peak Height: 500   Marker Baseline V to V?: Y    Marker Baseline V to V pts: 3
Lower Marker Selection: First Peak > 500 RFU                 Upper Marker Selection: Last Peak > 500 RFU
Ladder Size (bp)35, 100, 200, 300, 400, 500, 600, 700, 800, 900, 1000, 1500
Quantification Using: Upper Marker          Final Concentration (ng/uL): 0.5000          Dilution Factor: 12.0
```

 Copyright 2015 Advanced Analytical Technologies, Inc.

Sample: SampB4
Well Location: B4

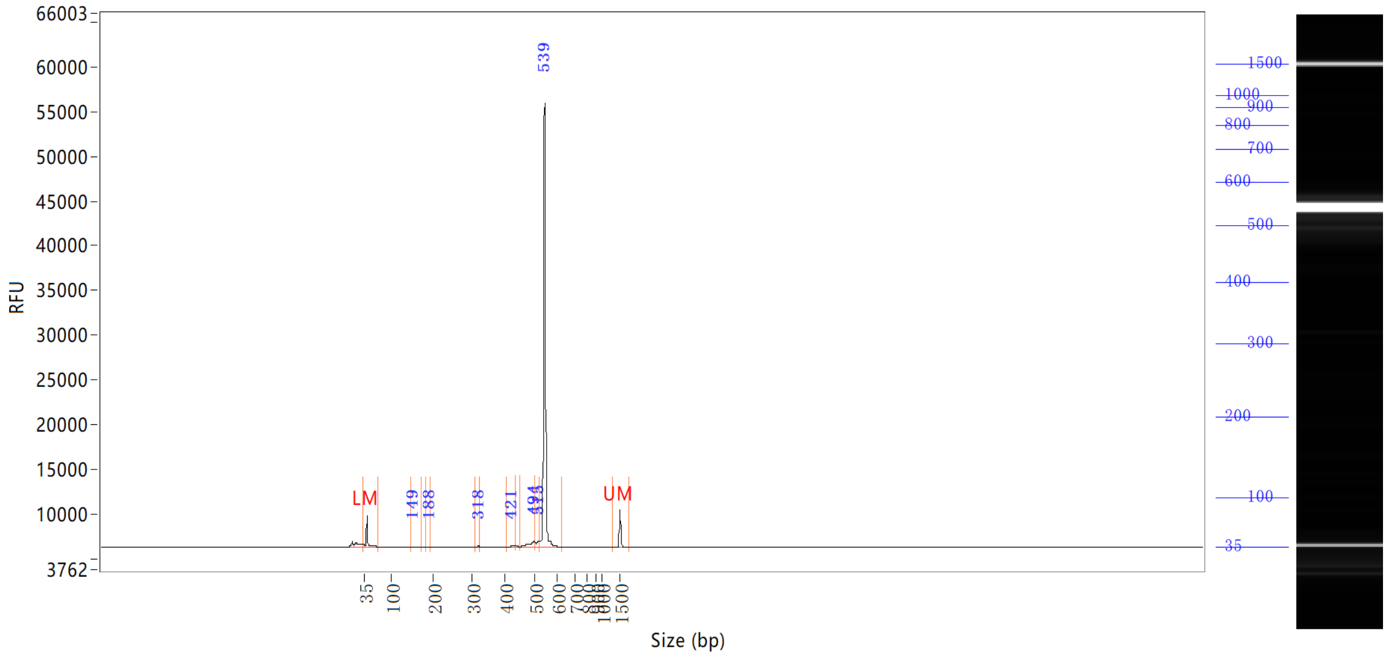

Peak	Size (bp)	Conc. (ng/uL)	From (bp)	To (bp)	Avg. Size (bp)	CV%	RFU	Corr. Peak Area
1	35 (LM)	0.6831	29	64	37	14.16	3454	21.318
2	149	0.1835	145	170	152	3.51	55	0.477
3	188	0.1361	181	192	187	1.04	64	0.354
4	318	0.2788	307	323	317	0.90	162	0.725
5	421	0.3085	404	435	423	1.43	81	0.802
6	494	3.6281	450	502	485	2.41	629	9.436
7	515	2.2351	502	524	514	1.21	592	5.813
8	539	98.0419	524	626	540	1.18	49717	254.981
9	1500 (UM)	0.5000	1307	1783	1501	2.89	4228	15.604

TIC:	104.8121	ng/uL
TIM:	324.0609	nmole/L
Total Conc.:	105.9936	ng/uL

Sample Peak Width (sec): 5   Sample Min Peak Height: 50   Sample Baseline V to V?: Y   Sample Baseline V to V pts: 3
Sample Filter: Binomial   # of Pts for Filter: 3   Sample Start Region (min): 0   Sample End Region (min): 80
Marker Peak Width (sec): 5   Marker Min Peak Height: 500   Marker Baseline V to V?: Y   Marker Baseline V to V pts: 3
Lower Marker Selection: First Peak > 500 RFU   Upper Marker Selection: Last Peak > 500 RFU
Ladder Size (bp)35, 100, 200, 300, 400, 500, 600, 700, 800, 900, 1000, 1500
Quantification Using: Upper Marker   Final Concentration (ng/uL): 0.5000   Dilution Factor: 12.0

 Copyright 2015 Advanced Analytical Technologies, Inc.

Sample: SampC4
Well Location: C4

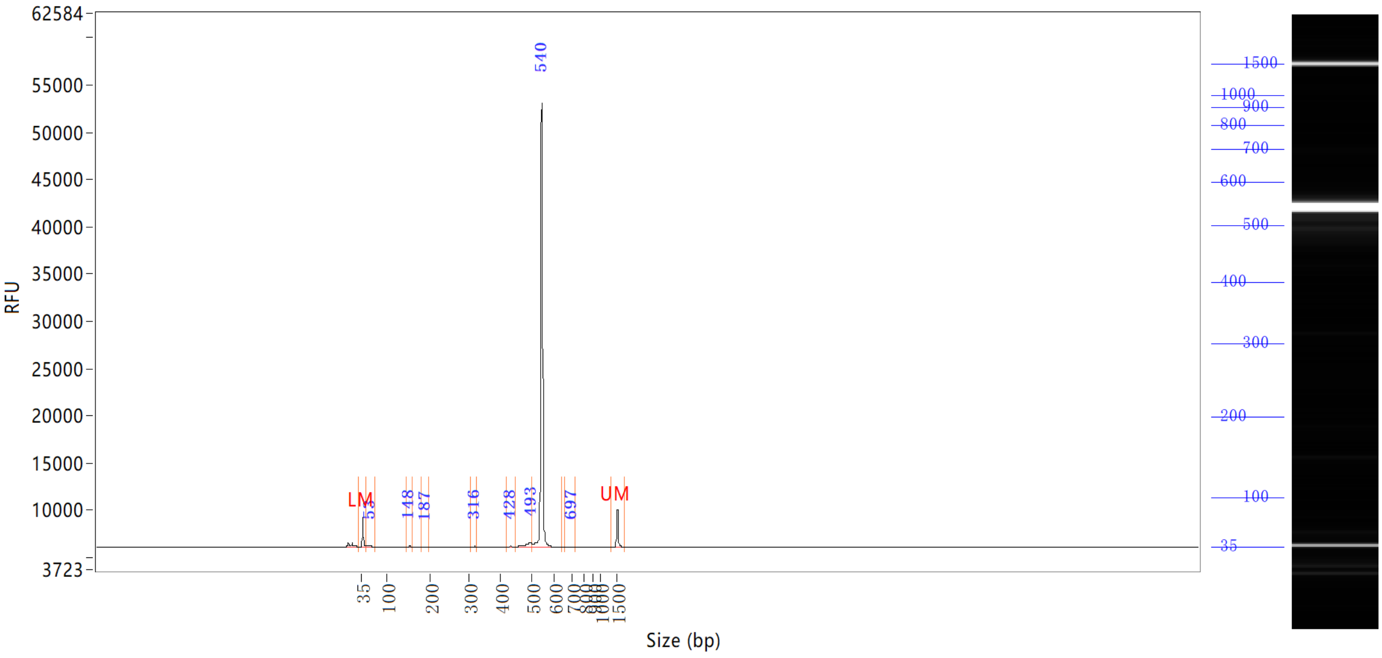

Peak	Size (bp)	Conc. (ng/uL)	From (bp)	To (bp)	Avg. Size (bp)	CV%	RFU	Corr. Peak Area
1	35 (LM)	0.5373	28	44	35	6.18	3088	15.591
2	53	1.0597	44	69	52	9.05	183	2.562
3	148	0.4376	144	157	148	1.51	168	1.058
4	187	0.3120	178	196	186	2.21	68	0.754
5	316	0.2686	303	323	314	1.24	126	0.650
6	428	0.3117	417	446	430	1.73	75	0.754
7	493	3.4949	446	502	483	2.68	508	8.450
8	540	100.1287	502	638	540	1.32	47055	242.100
9	697	0.2363	663	727	691	1.67	68	0.571
10	1500 (UM)	0.5000	1347	1724	1501	2.30	4007	14.507

TIC:	106.2494	ng/uL
TIM:	361.1968	nmole/L
Total Conc.:	107.8072	ng/uL

Sample Peak Width (sec): 5 Sample Min Peak Height: 50 Sample Baseline V to V?: Y Sample Baseline V to V pts: 3
Sample Filter: Binomial # of Pts for Filter: 3 Sample Start Region (min): 0 Sample End Region (min): 80
Marker Peak Width (sec): 5 Marker Min Peak Height: 500 Marker Baseline V to V?: Y Marker Baseline V to V pts: 3
Lower Marker Selection: First Peak > 500 RFU Upper Marker Selection: Last Peak > 500 RFU
Ladder Size (bp)35, 100, 200, 300, 400, 500, 600, 700, 800, 900, 1000, 1500
Quantification Using: Upper Marker Final Concentration (ng/uL): 0.5000 Dilution Factor: 12.0

 Copyright 2015 Advanced Analytical Technologies, Inc.

Sample: SampD4
Well Location: D4

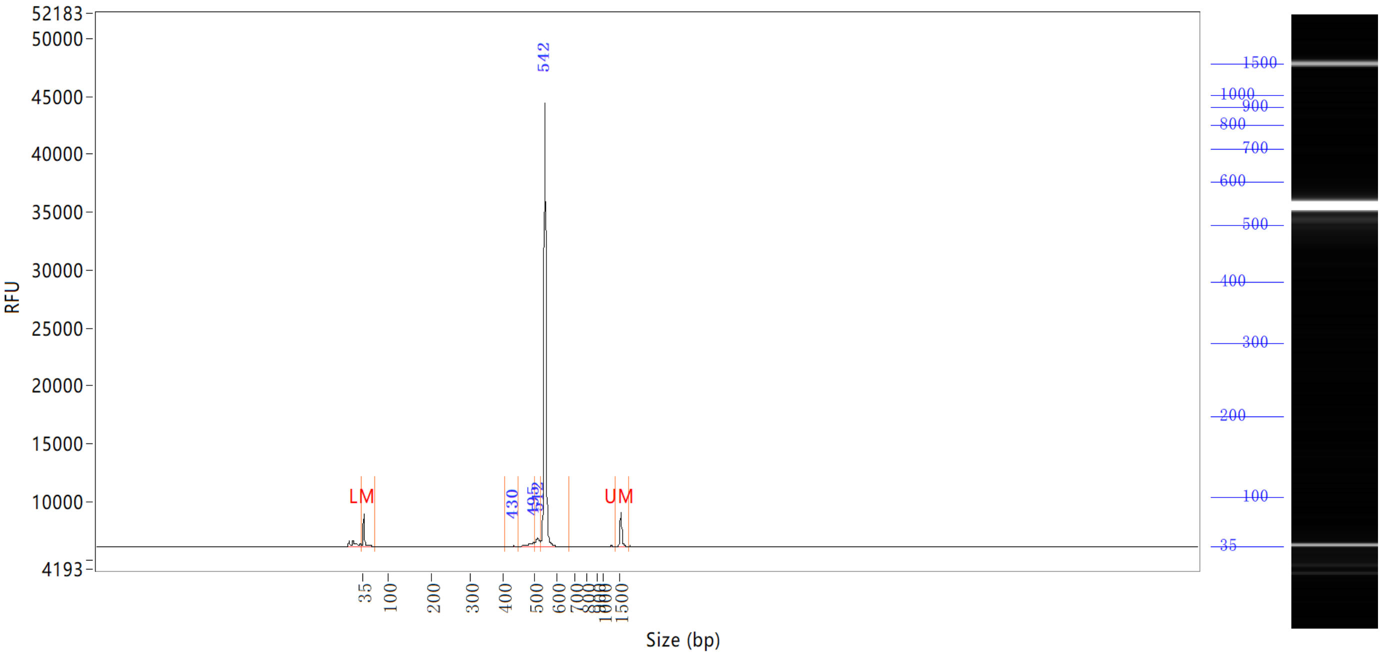

Peak	Size (bp)	Conc. (ng/uL)	From (bp)	To (bp)	Avg. Size (bp)	CV%	RFU	Corr. Peak Area
1	35 (LM)	0.6189	31	64	36	13.58	2877	16.360
2	430	0.4268	403	446	429	2.39	70	0.940
3	495	2.7816	446	499	481	2.68	358	6.128
4	512	3.4450	499	525	512	1.38	760	7.589
5	542	95.1670	525	663	543	1.30	38372	209.659
6	1500 (UM)	0.5000	1354	1737	1500	2.41	2982	13.218

TIC:	101.8204	ng/uL
TIM:	310.6301	nmole/L
Total Conc.:	103.7613	ng/uL

Sample Peak Width (sec): 5 Sample Min Peak Height: 50 Sample Baseline V to V?: Y Sample Baseline V to V pts: 3
Sample Filter: Binomial # of Pts for Filter: 3 Sample Start Region (min): 0 Sample End Region (min): 80
Marker Peak Width (sec): 5 Marker Min Peak Height: 500 Marker Baseline V to V?: Y Marker Baseline V to V pts: 3
Lower Marker Selection: First Peak > 500 RFU Upper Marker Selection: Last Peak > 500 RFU
Ladder Size (bp)35, 100, 200, 300, 400, 500, 600, 700, 800, 900, 1000, 1500
Quantification Using: Upper Marker Final Concentration (ng/uL): 0.5000 Dilution Factor: 12.0

 Copyright 2015 Advanced Analytical Technologies, Inc.

Sample: SampE4
Well Location: E4

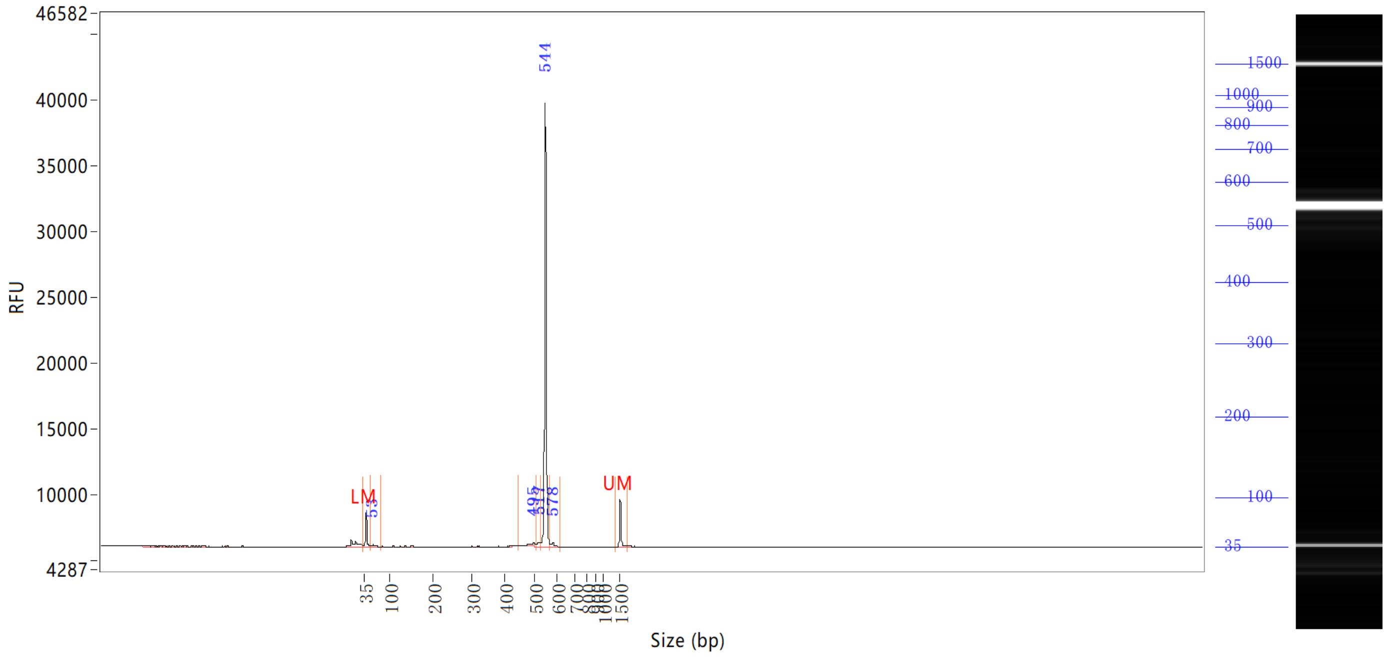

Peak	Size (bp)	Conc. (ng/uL)	From (bp)	To (bp)	Avg. Size (bp)	CV%	RFU	Corr. Peak Area
1	35 (LM)	0.5840	31	49	36	9.31	2619	14.500
2	53	0.6557	49	73	54	7.74	136	1.357
3	495	2.3869	447	504	485	2.62	295	4.939
4	517	1.5515	504	526	516	1.22	332	3.210
5	544	75.5092	526	569	544	0.83	33773	156.233
6	578	1.0838	569	618	578	1.29	304	2.242
7	1500 (UM)	0.5000	1374	1711	1499	2.35	3574	12.414

TIC:	81.1871	ng/uL
TIM:	264.4400	nmole/L
Total Conc.:	83.4169	ng/uL

Sample Peak Width (sec): 5   Sample Min Peak Height: 50   Sample Baseline V to V?: Y   Sample Baseline V to V pts: 3
Sample Filter: Binomial   # of Pts for Filter: 3   Sample Start Region (min): 0   Sample End Region (min): 80
Marker Peak Width (sec): 5   Marker Min Peak Height: 500   Marker Baseline V to V?: Y   Marker Baseline V to V pts: 3
Lower Marker Selection: First Peak > 500 RFU   Upper Marker Selection: Last Peak > 500 RFU
Ladder Size (bp)35, 100, 200, 300, 400, 500, 600, 700, 800, 900, 1000, 1500
Quantification Using: Upper Marker   Final Concentration (ng/uL): 0.5000   Dilution Factor: 12.0

 Copyright 2015 Advanced Analytical Technologies, Inc.

Sample: SampF4
Well Location: F4

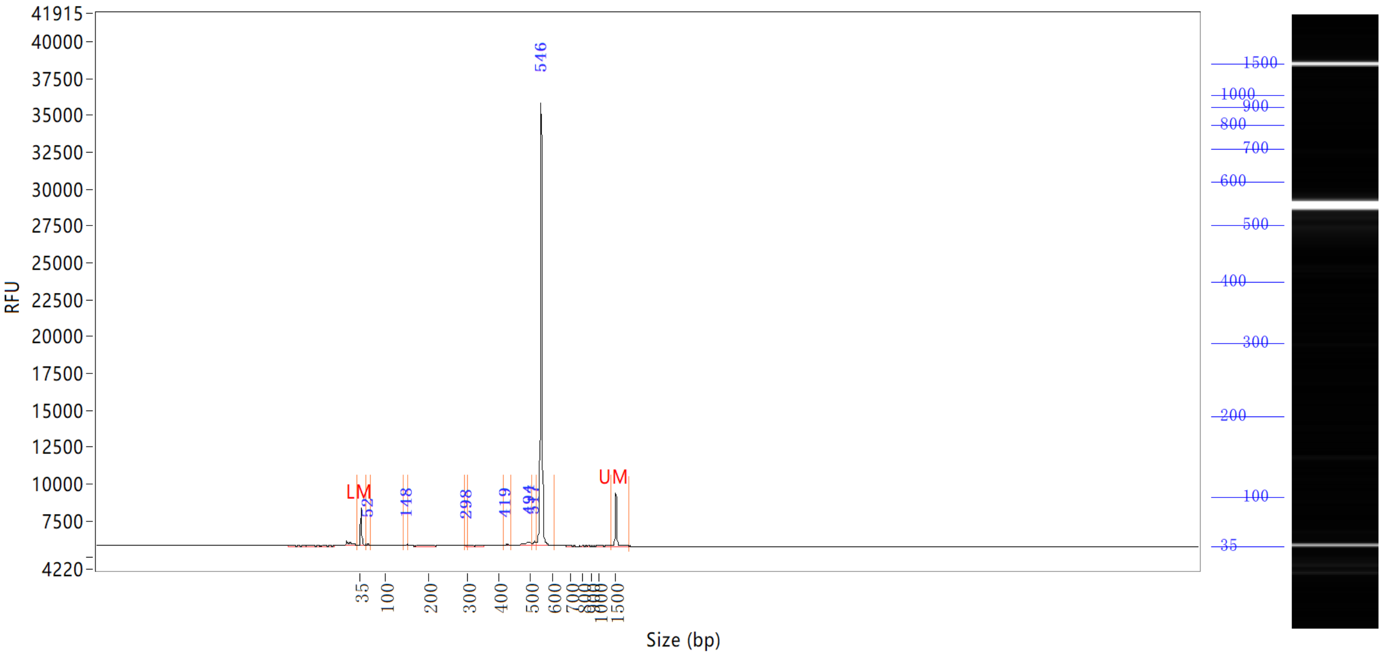

Peak	Size (bp)	Conc. (ng/uL)	From (bp)	To (bp)	Avg. Size (bp)	CV%	RFU	Corr. Peak Area
1	35 (LM)	0.5296	28	49	35	8.83	2578	13.696
2	52	0.4035	49	61	53	5.04	105	0.870
3	148	0.4607	142	152	147	1.28	162	0.993
4	298	0.1619	294	302	297	0.64	74	0.349
5	419	0.3474	413	434	422	1.04	99	0.749
6	494	2.3115	434	505	484	2.80	275	4.981
7	517	1.3253	505	527	517	1.24	288	2.856
8	546	64.8876	527	606	544	0.92	30079	139.831
9	1500 (UM)	0.5000	1374	1895	1506	3.88	3586	12.930

TIC:	69.8979	ng/uL
TIM:	228.2339	nmole/L
Total Conc.:	72.3351	ng/uL

Sample Peak Width (sec): 5 Sample Min Peak Height: 50 Sample Baseline V to V?: Y Sample Baseline V to V pts: 3
Sample Filter: Binomial # of Pts for Filter: 3 Sample Start Region (min): 0 Sample End Region (min): 80
Marker Peak Width (sec): 5 Marker Min Peak Height: 500 Marker Baseline V to V?: Y Marker Baseline V to V pts: 3
Lower Marker Selection: First Peak > 500 RFU Upper Marker Selection: Last Peak > 500 RFU
Ladder Size (bp)35, 100, 200, 300, 400, 500, 600, 700, 800, 900, 1000, 1500
Quantification Using: Upper Marker Final Concentration (ng/uL): 0.5000 Dilution Factor: 12.0

 Copyright 2015 Advanced Analytical Technologies, Inc.

Sample: SampG4
Well Location: G4

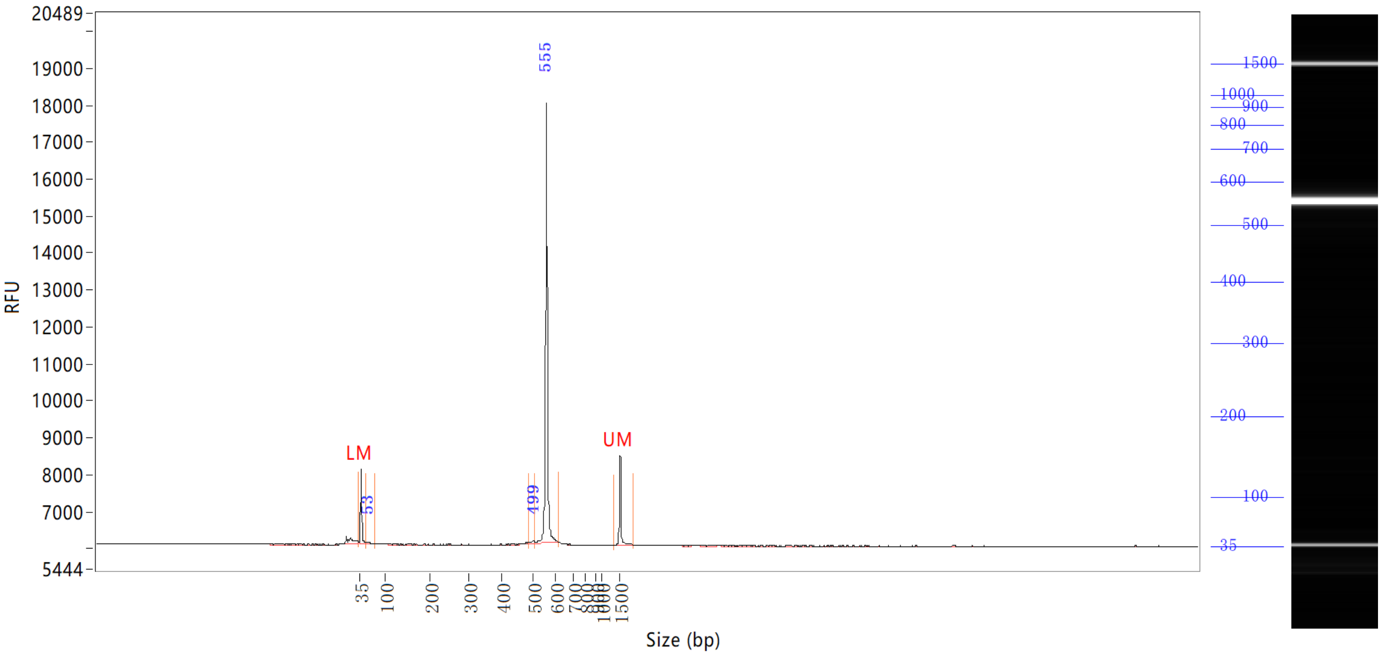

Peak	Size (bp)	Conc. (ng/uL)	From (bp)	To (bp)	Avg. Size (bp)	CV%	RFU	Corr. Peak Area
1	35 (LM)	0.5916	31	50	35	8.06	2010	10.660
2	53	0.3119	50	70	54	6.61	51	0.468
3	499	0.4953	487	509	497	1.13	78	0.744
4	555	39.5488	509	610	555	1.40	11921	59.381
5	1500 (UM)	0.5000	1354	1895	1508	3.56	2417	9.009

TIC:	40.3559	ng/uL
TIM:	128.3654	nmole/L
Total Conc.:	43.2456	ng/uL

Sample Peak Width (sec): 5   Sample Min Peak Height: 50   Sample Baseline V to V?: Y   Sample Baseline V to V pts: 3
Sample Filter: Binomial   # of Pts for Filter: 3   Sample Start Region (min): 0   Sample End Region (min): 80
Marker Peak Width (sec): 5   Marker Min Peak Height: 500   Marker Baseline V to V?: Y   Marker Baseline V to V pts: 3
Lower Marker Selection: First Peak > 500 RFU   Upper Marker Selection: Last Peak > 500 RFU
Ladder Size (bp)35, 100, 200, 300, 400, 500, 600, 700, 800, 900, 1000, 1500
Quantification Using: Upper Marker   Final Concentration (ng/uL): 0.5000   Dilution Factor: 12.0

 Copyright 2015 Advanced Analytical Technologies, Inc.

Sample: SampH4
Well Location: H4

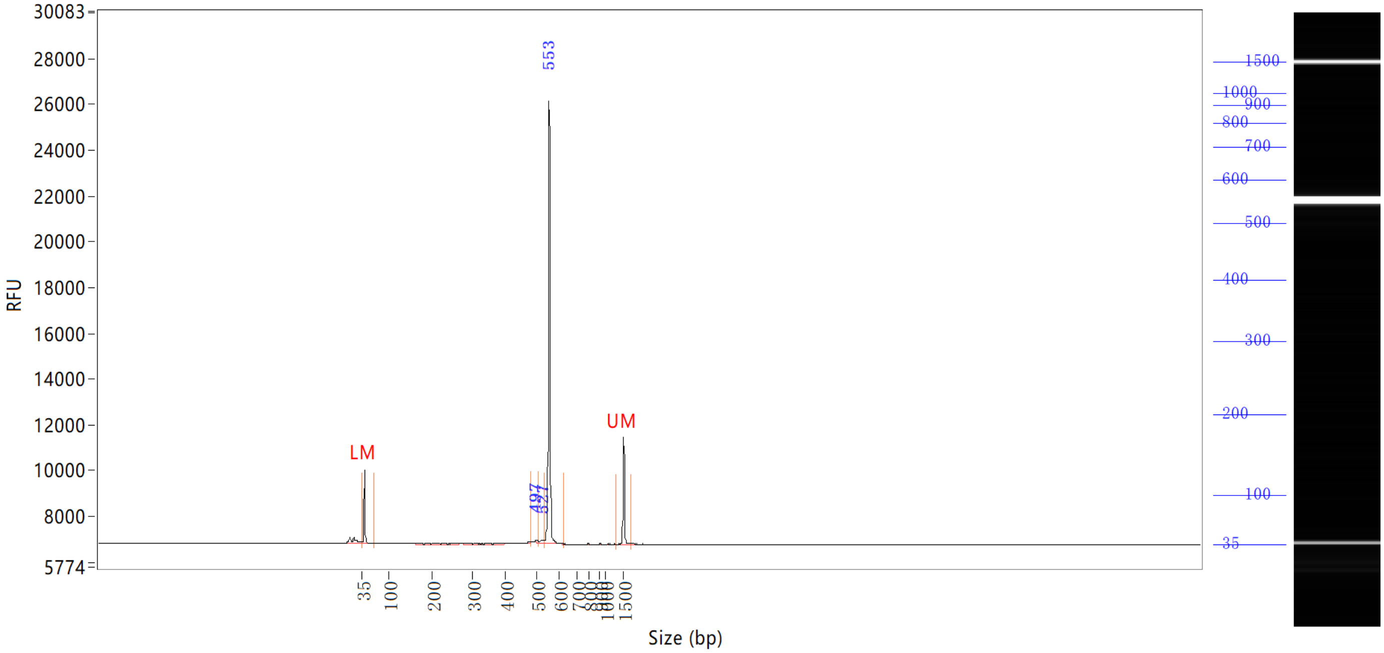

Peak	Size (bp)	Conc. (ng/uL)	From (bp)	To (bp)	Avg. Size (bp)	CV%	RFU	Corr. Peak Area
1	35 (LM)	0.5218	31	63	36	10.23	3227	16.545
2	497	0.3644	484	508	497	1.11	122	0.963
3	527	0.4489	508	532	522	1.28	115	1.186
4	553	31.6645	532	626	551	1.00	19372	83.671
5	1500 (UM)	0.5000	1287	1717	1496	2.16	4699	15.855

TIC:	32.4779	ng/uL
TIM:	97.1893	nmole/L
Total Conc.:	34.1686	ng/uL

Sample Peak Width (sec): 5 Sample Min Peak Height: 50 Sample Baseline V to V?: Y Sample Baseline V to V pts: 3
Sample Filter: Binomial # of Pts for Filter: 3 Sample Start Region (min): 0 Sample End Region (min): 80
Marker Peak Width (sec): 5 Marker Min Peak Height: 500 Marker Baseline V to V?: Y Marker Baseline V to V pts: 3
Lower Marker Selection: First Peak > 500 RFU Upper Marker Selection: Last Peak > 500 RFU
Ladder Size (bp)35, 100, 200, 300, 400, 500, 600, 700, 800, 900, 1000, 1500
Quantification Using: Upper Marker Final Concentration (ng/uL): 0.5000 Dilution Factor: 12.0

 Copyright 2015 Advanced Analytical Technologies, Inc.

Sample: SampA5
Well Location: A5

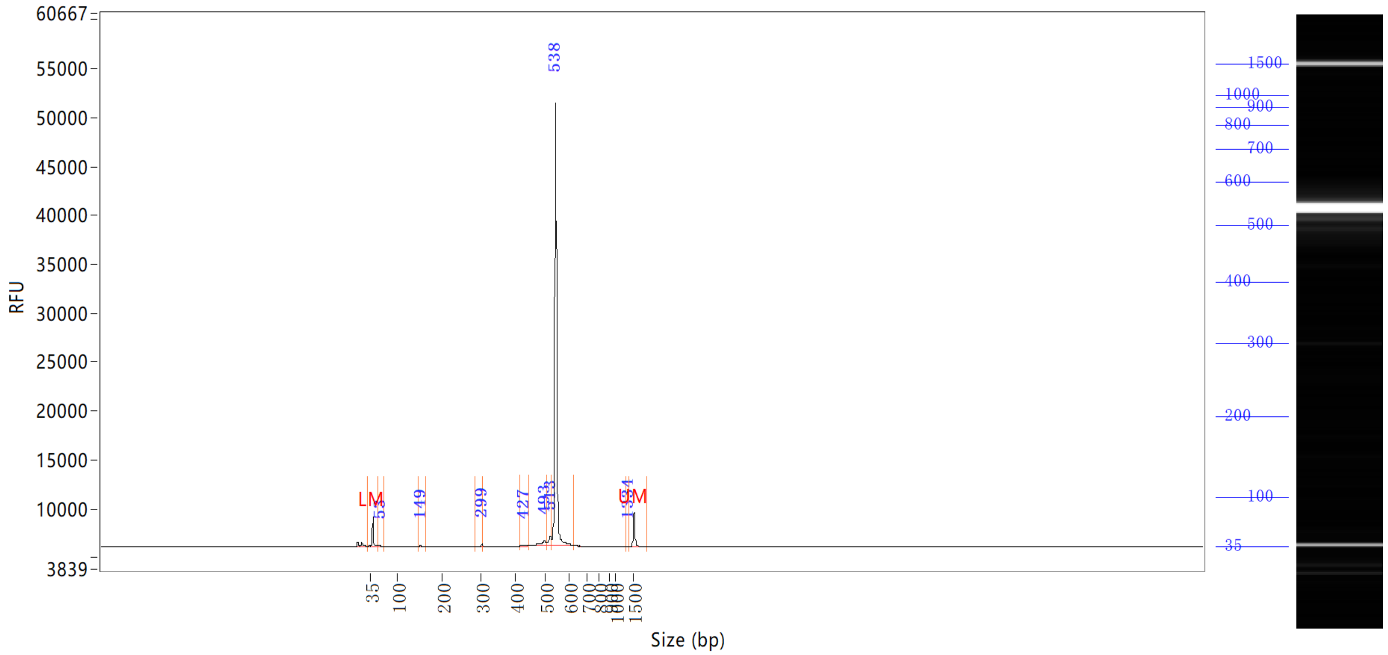

Peak	Size (bp)	Conc. (ng/uL)	From (bp)	To (bp)	Avg. Size (bp)	CV%	RFU	Corr. Peak Area
1	35 (LM)	0.5521	23	49	35	8.52	3030	15.503
2	53	0.3634	49	65	54	5.70	89	0.850
3	149	0.2424	145	162	150	2.17	81	0.567
4	299	0.4722	284	304	298	0.98	244	1.105
5	427	0.3081	417	444	427	1.12	111	0.721
6	493	3.5582	444	500	482	2.60	531	8.326
7	513	3.3495	500	523	513	1.15	985	7.838
8	538	103.7745	523	620	539	1.46	45333	242.841
9	1334	0.0907	1280	1380	1332	1.76	55	0.212
10	1500 (UM)	0.5000	1380	1888	1503	2.87	3529	14.041

TIC:	112.1590	ng/uL
TIM:	357.3033	nmole/L
Total Conc.:	114.0266	ng/uL

```
Sample Peak Width (sec): 5     Sample Min Peak Height: 50     Sample Baseline V to V?: Y     Sample Baseline V to V pts: 3
Sample Filter: Binomial        # of Pts for Filter: 3         Sample Start Region (min): 0   Sample End Region (min): 80
Marker Peak Width (sec): 5     Marker Min Peak Height: 500    Marker Baseline V to V?: Y     Marker Baseline V to V pts: 3
Lower Marker Selection: First Peak > 500 RFU                  Upper Marker Selection: Last Peak > 500 RFU
Ladder Size (bp)35, 100, 200, 300, 400, 500, 600, 700, 800, 900, 1000, 1500
Quantification Using: Upper Marker          Final Concentration (ng/uL): 0.5000          Dilution Factor: 12.0
```

 Copyright 2015 Advanced Analytical Technologies, Inc.

Sample: SampB5
Well Location: B5

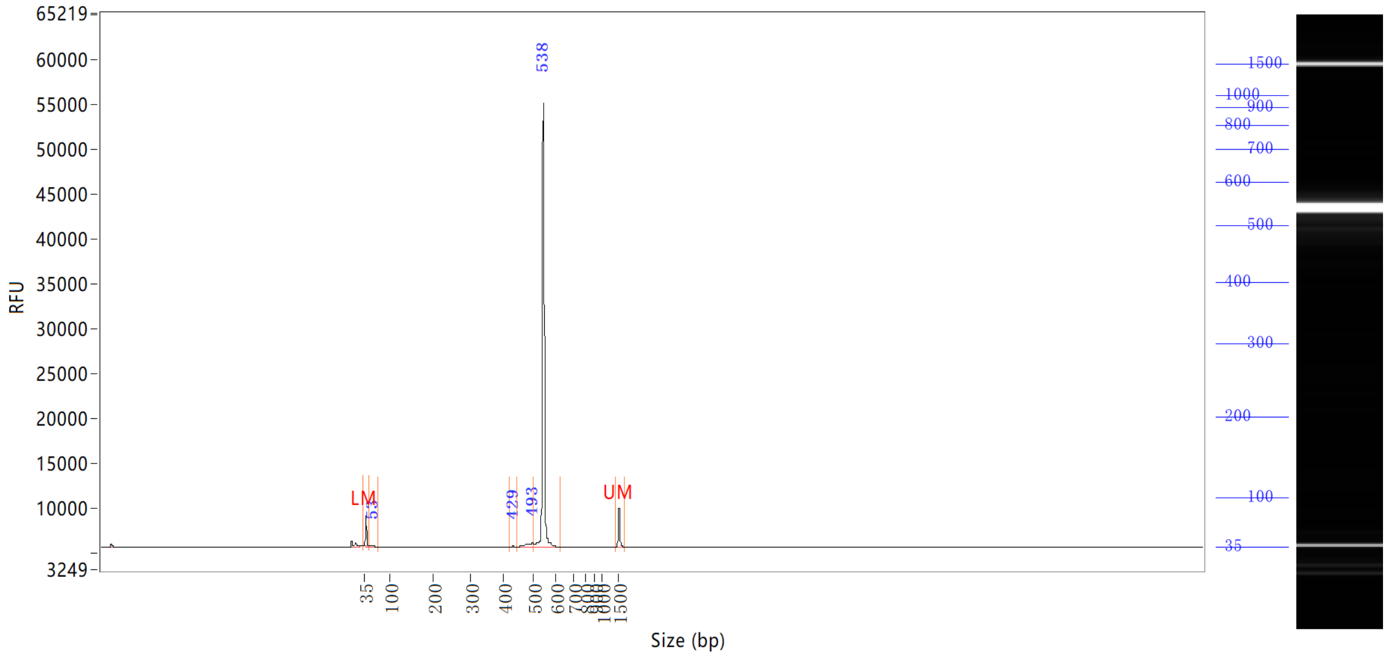

Peak	Size (bp)	Conc. (ng/uL)	From (bp)	To (bp)	Avg. Size (bp)	CV%	RFU	Corr. Peak Area
1	35 (LM)	0.5399	31	45	35	6.14	3410	17.062
2	53	0.7023	45	67	52	8.00	161	1.850
3	429	0.2678	418	444	430	1.47	67	0.705
4	493	3.2835	444	500	481	2.71	509	8.647
5	538	100.0656	500	618	538	1.40	49523	263.523
6	1500 (UM)	0.5000	1387	1671	1497	1.97	4354	15.801

TIC:	104.3193	ng/uL
TIM:	340.4513	nmole/L
Total Conc.:	105.6601	ng/uL

Sample Peak Width (sec): 5 Sample Min Peak Height: 50 Sample Baseline V to V?: Y Sample Baseline V to V pts: 3
Sample Filter: Binomial # of Pts for Filter: 3 Sample Start Region (min): 0 Sample End Region (min): 80
Marker Peak Width (sec): 5 Marker Min Peak Height: 500 Marker Baseline V to V?: Y Marker Baseline V to V pts: 3
Lower Marker Selection: First Peak > 500 RFU Upper Marker Selection: Last Peak > 500 RFU
Ladder Size (bp)35, 100, 200, 300, 400, 500, 600, 700, 800, 900, 1000, 1500
Quantification Using: Upper Marker Final Concentration (ng/uL): 0.5000 Dilution Factor: 12.0

 Copyright 2015 Advanced Analytical Technologies, Inc.

Sample: SampC5
Well Location: C5

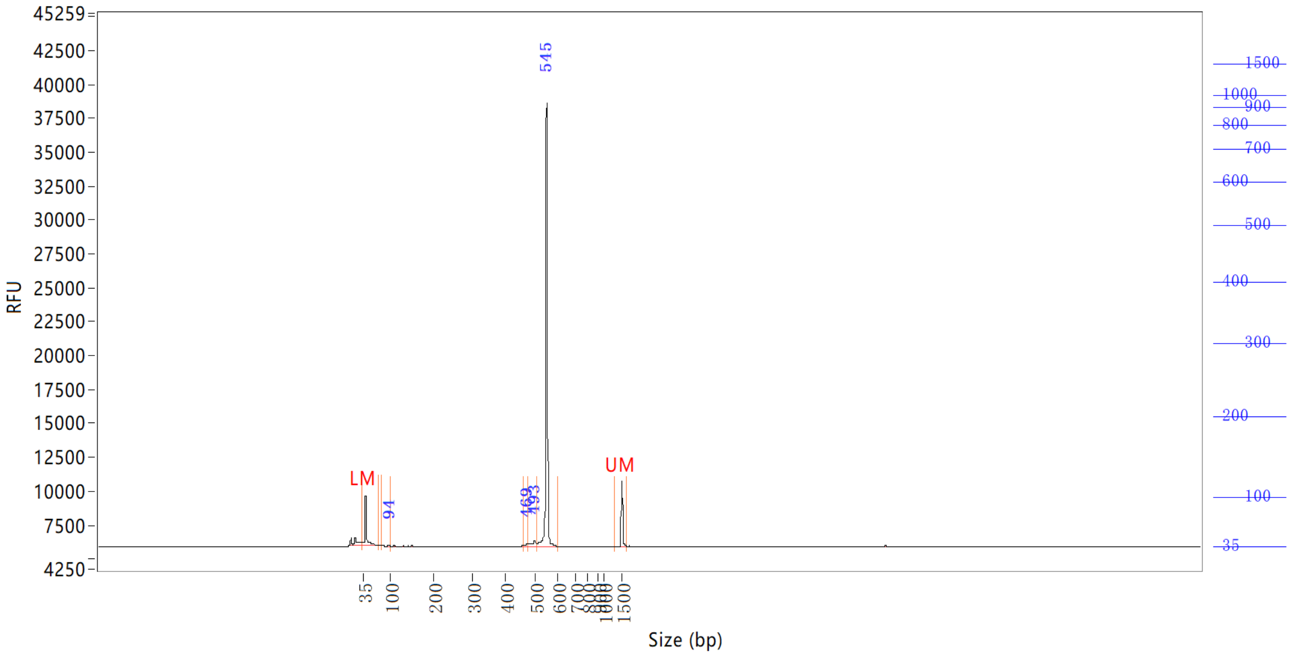

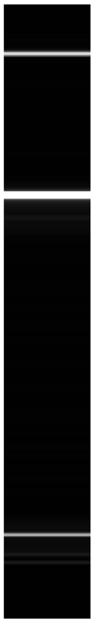

Peak	Size (bp)	Conc. (ng/uL)	From (bp)	To (bp)	Avg. Size (bp)	CV%	RFU	Corr. Peak Area
1	35 (LM)	0.7428	28	70	37	16.04	3612	24.365
2	94	0.1862	76	101	93	3.94	58	0.509
3	469	0.4255	459	473	467	0.79	155	1.163
4	493	1.7568	473	502	488	1.61	393	4.802
5	545	51.4238	502	599	543	1.23	32759	140.568
6	1500 (UM)	0.5000	1280	1658	1495	1.91	4792	16.401

TIC:	53.7923	ng/uL
TIM:	166.5508	nmole/L
Total Conc.:	55.5380	ng/uL

Sample Peak Width (sec): 5 Sample Min Peak Height: 50 Sample Baseline V to V?: Y Sample Baseline V to V pts: 3
Sample Filter: Binomial # of Pts for Filter: 3 Sample Start Region (min): 0 Sample End Region (min): 80
Marker Peak Width (sec): 5 Marker Min Peak Height: 500 Marker Baseline V to V?: Y Marker Baseline V to V pts: 3
Lower Marker Selection: First Peak > 500 RFU Upper Marker Selection: Last Peak > 500 RFU
Ladder Size (bp)35, 100, 200, 300, 400, 500, 600, 700, 800, 900, 1000, 1500
Quantification Using: Upper Marker Final Concentration (ng/uL): 0.5000 Dilution Factor: 12.0

 Copyright 2015 Advanced Analytical Technologies, Inc.

Sample: SampD5
Well Location: D5

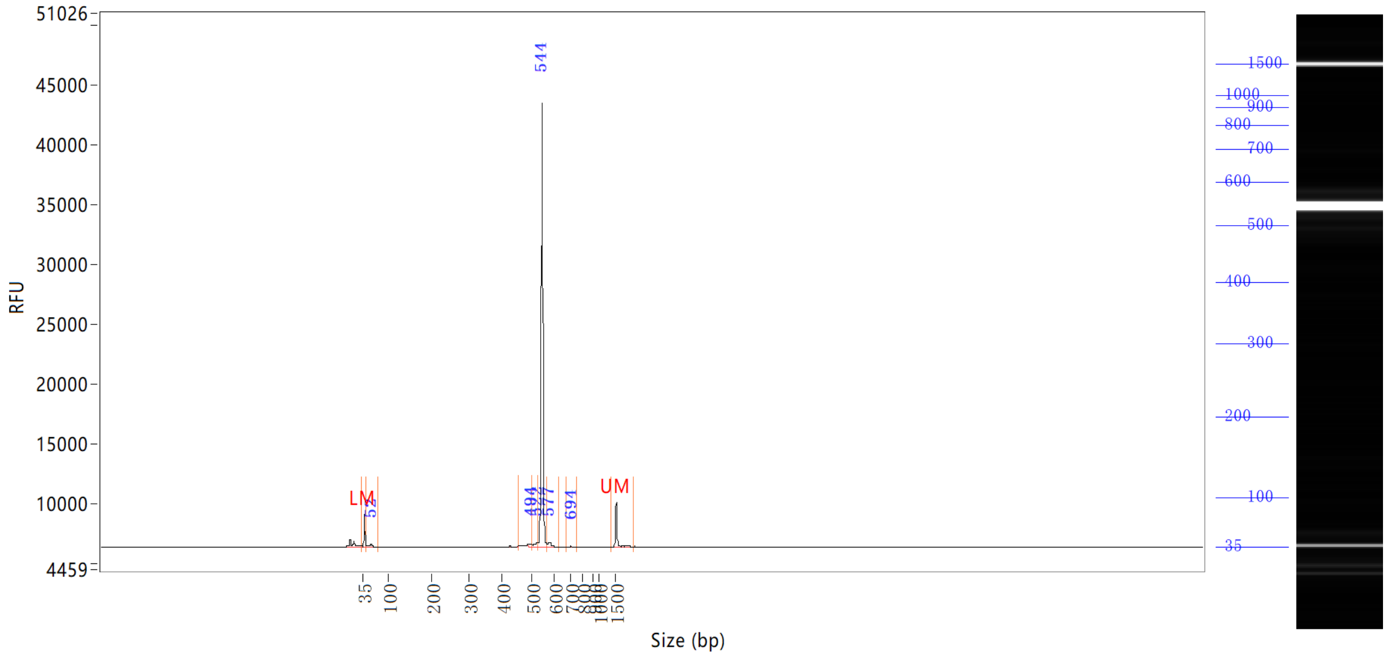

Peak	Size (bp)	Conc. (ng/uL)	From (bp)	To (bp)	Avg. Size (bp)	CV%	RFU	Corr. Peak Area
1	35 (LM)	0.4728	30	41	35	4.89	2697	13.437
2	52	1.4718	41	70	50	10.87	232	3.485
3	494	1.5059	457	504	487	2.19	245	3.566
4	522	1.0910	504	526	516	1.17	274	2.584
5	544	74.6184	526	571	543	0.88	37192	176.715
6	577	1.0970	571	624	580	1.29	350	2.598
7	694	0.2113	670	741	700	2.27	63	0.500
8	1500 (UM)	0.5000	1354	2020	1516	6.06	3757	14.209

TIC:	79.9954	ng/uL
TIM:	286.5196	nmole/L
Total Conc.:	81.8077	ng/uL

Sample Peak Width (sec): 5 Sample Min Peak Height: 50 Sample Baseline V to V?: Y Sample Baseline V to V pts: 3
Sample Filter: Binomial # of Pts for Filter: 3 Sample Start Region (min): 0 Sample End Region (min): 80
Marker Peak Width (sec): 5 Marker Min Peak Height: 500 Marker Baseline V to V?: Y Marker Baseline V to V pts: 3
Lower Marker Selection: First Peak > 500 RFU Upper Marker Selection: Last Peak > 500 RFU
Ladder Size (bp)35, 100, 200, 300, 400, 500, 600, 700, 800, 900, 1000, 1500
Quantification Using: Upper Marker Final Concentration (ng/uL): 0.5000 Dilution Factor: 12.0

 Copyright 2015 Advanced Analytical Technologies, Inc.

Sample: SampE5
Well Location: E5

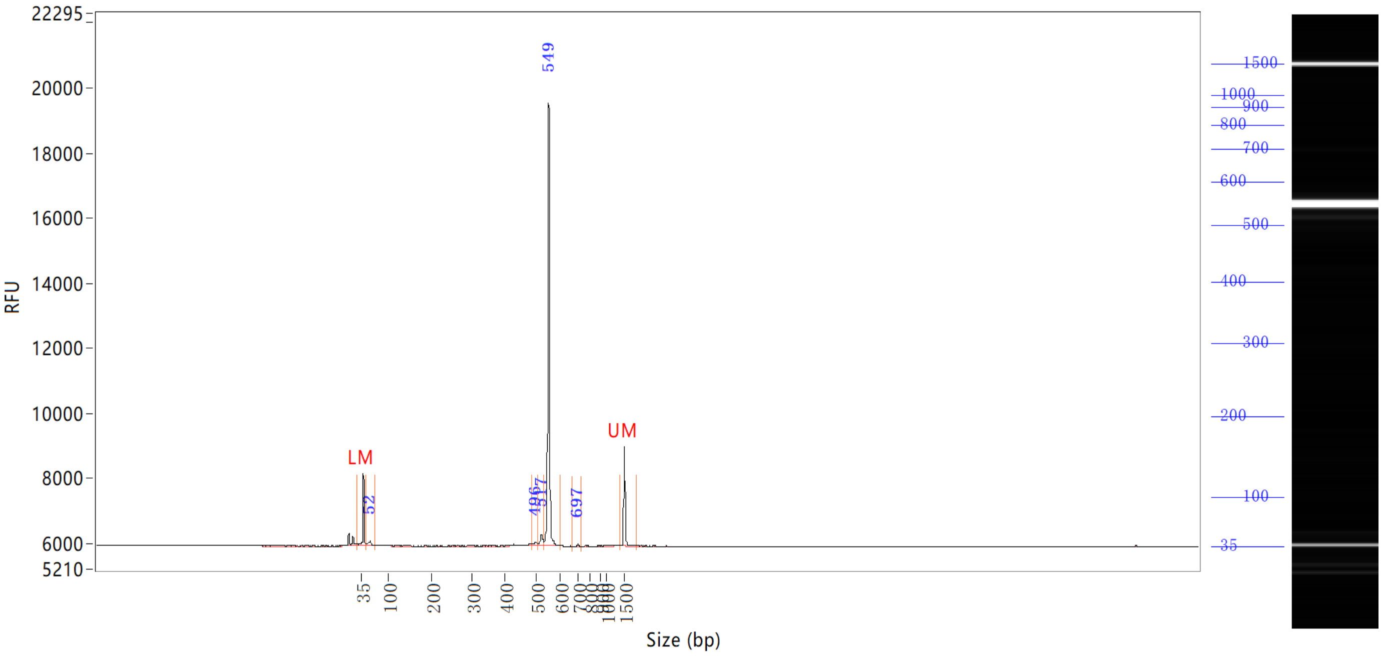

Peak	Size (bp)	Conc. (ng/uL)	From (bp)	To (bp)	Avg. Size (bp)	CV%	RFU	Corr. Peak Area
1	35 (LM)	0.5481	23	44	34	7.96	2235	11.762
2	52	0.9777	44	64	50	7.34	149	1.748
3	496	0.5683	483	505	494	1.18	95	1.016
4	517	1.4388	505	529	517	1.13	351	2.573
5	549	34.2324	529	605	548	0.98	13630	61.221
6	697	0.1948	670	721	695	0.99	71	0.348
7	1500 (UM)	0.5000	1367	1869	1498	3.00	3075	10.730

TIC:	37.4121	ng/uL
TIM:	141.7549	nmole/L
Total Conc.:	40.1204	ng/uL

Sample Peak Width (sec): 5   Sample Min Peak Height: 50   Sample Baseline V to V?: Y   Sample Baseline V to V pts: 3
Sample Filter: Binomial   # of Pts for Filter: 3   Sample Start Region (min): 0   Sample End Region (min): 80
Marker Peak Width (sec): 5   Marker Min Peak Height: 500   Marker Baseline V to V?: Y   Marker Baseline V to V pts: 3
Lower Marker Selection: First Peak > 500 RFU   Upper Marker Selection: Last Peak > 500 RFU
Ladder Size (bp)35, 100, 200, 300, 400, 500, 600, 700, 800, 900, 1000, 1500
Quantification Using: Upper Marker   Final Concentration (ng/uL): 0.5000   Dilution Factor: 12.0

 Copyright 2015 Advanced Analytical Technologies, Inc.

Sample: SampF5
Well Location: F5

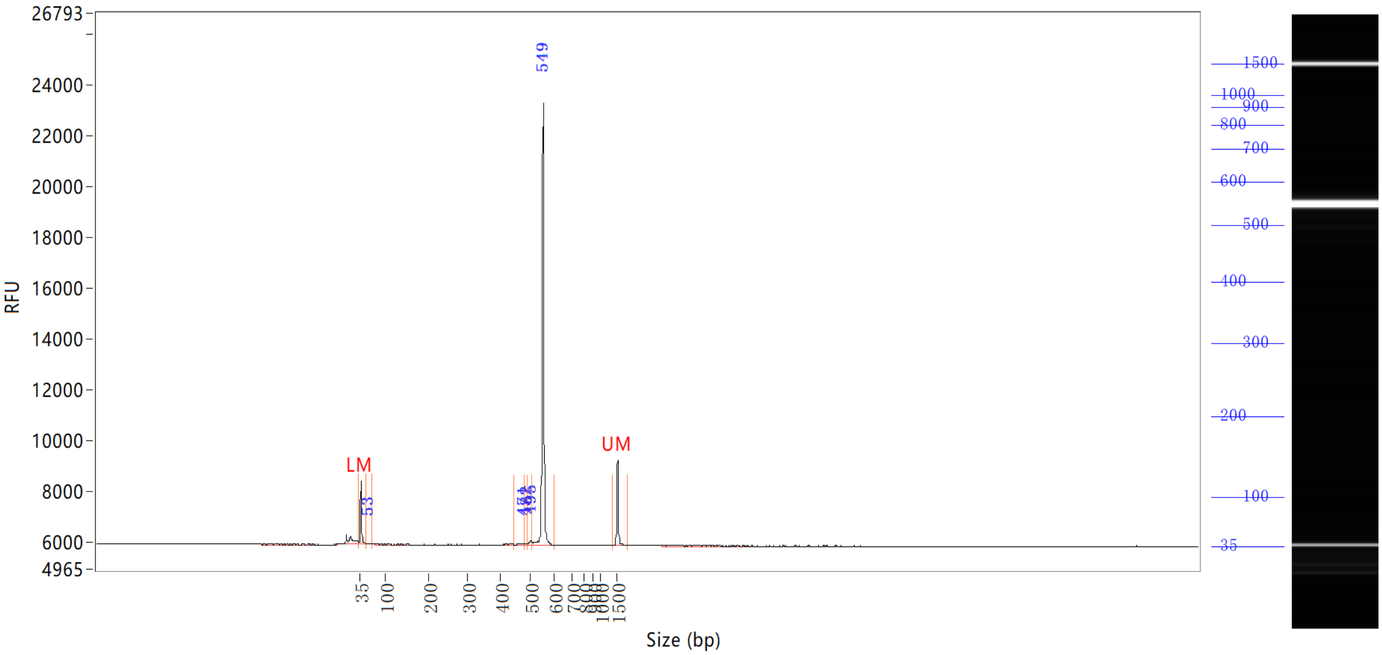

Peak	Size (bp)	Conc. (ng/uL)	From (bp)	To (bp)	Avg. Size (bp)	CV%	RFU	Corr. Peak Area
1	35 (LM)	0.5474	31	50	35	8.83	2471	13.199
2	53	0.2884	50	64	54	5.71	54	0.579
3	471	0.3441	443	476	466	1.61	65	0.691
4	482	0.2314	476	485	481	0.60	66	0.465
5	495	0.6409	485	506	495	1.08	133	1.288
6	549	39.8185	506	598	547	1.20	17383	80.006
7	1500 (UM)	0.5000	1360	1816	1502	3.07	3379	12.056

TIC:	41.3232	ng/uL
TIM:	132.6763	nmole/L
Total Conc.:	43.4826	ng/uL

Sample Peak Width (sec): 5   Sample Min Peak Height: 50   Sample Baseline V to V?: Y   Sample Baseline V to V pts: 3
Sample Filter: Binomial   # of Pts for Filter: 3   Sample Start Region (min): 0   Sample End Region (min): 80
Marker Peak Width (sec): 5   Marker Min Peak Height: 500   Marker Baseline V to V?: Y   Marker Baseline V to V pts: 3
Lower Marker Selection: First Peak > 500 RFU   Upper Marker Selection: Last Peak > 500 RFU
Ladder Size (bp)35, 100, 200, 300, 400, 500, 600, 700, 800, 900, 1000, 1500
Quantification Using: Upper Marker   Final Concentration (ng/uL): 0.5000   Dilution Factor: 12.0

Sample: SampG5
Well Location: G5

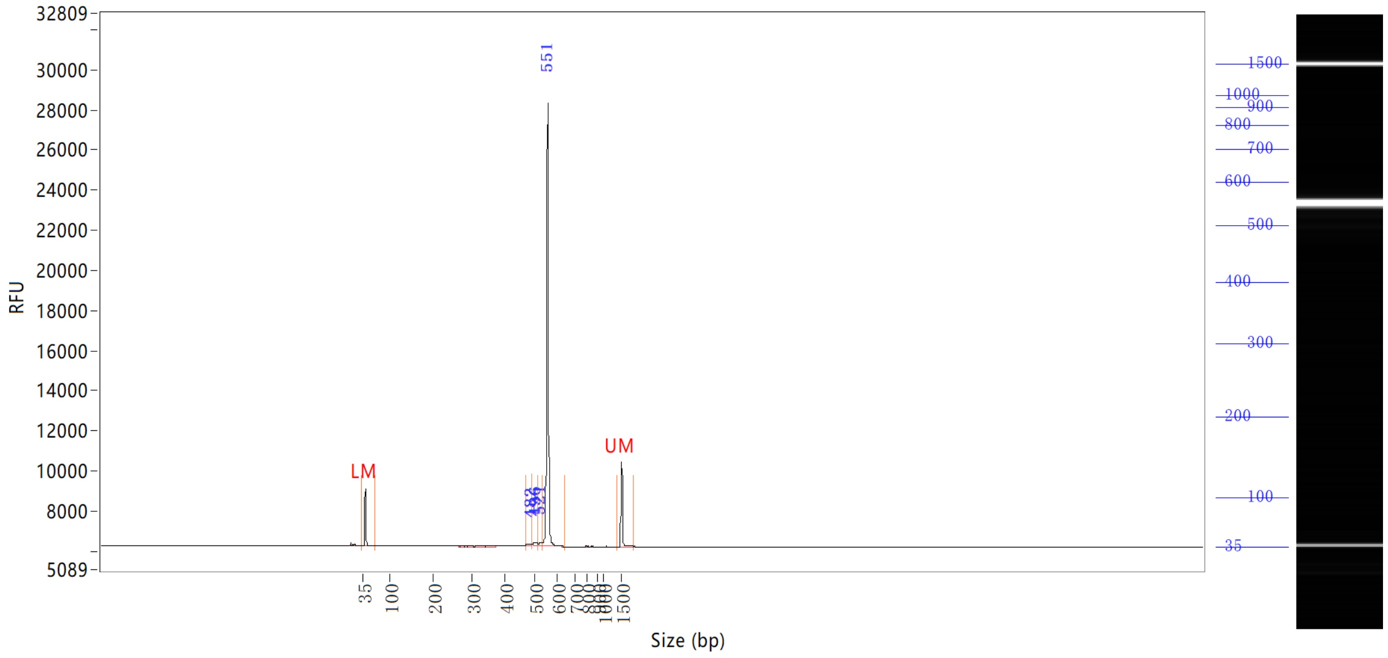

Peak	Size (bp)	Conc. (ng/uL)	From (bp)	To (bp)	Avg. Size (bp)	CV%	RFU	Corr. Peak Area
1	35 (LM)	0.4879	29	62	36	9.61	2843	13.962
2	482	0.2310	468	488	478	1.15	55	0.551
3	496	0.5293	488	512	498	1.23	139	1.262
4	521	0.5553	512	530	522	0.99	165	1.324
5	551	42.1350	530	637	550	0.97	22092	100.488
6	1500 (UM)	0.5000	1367	1862	1499	2.38	4221	14.309

TIC:	43.4506	ng/uL
TIM:	130.3612	nmole/L
Total Conc.:	45.1466	ng/uL

Sample Peak Width (sec): 5 Sample Min Peak Height: 50 Sample Baseline V to V?: Y Sample Baseline V to V pts: 3
Sample Filter: Binomial # of Pts for Filter: 3 Sample Start Region (min): 0 Sample End Region (min): 80
Marker Peak Width (sec): 5 Marker Min Peak Height: 500 Marker Baseline V to V?: Y Marker Baseline V to V pts: 3
Lower Marker Selection: First Peak > 500 RFU Upper Marker Selection: Last Peak > 500 RFU
Ladder Size (bp)35, 100, 200, 300, 400, 500, 600, 700, 800, 900, 1000, 1500
Quantification Using: Upper Marker Final Concentration (ng/uL): 0.5000 Dilution Factor: 12.0

 Copyright 2015 Advanced Analytical Technologies, Inc.

Sample: SampH5
Well Location: H5

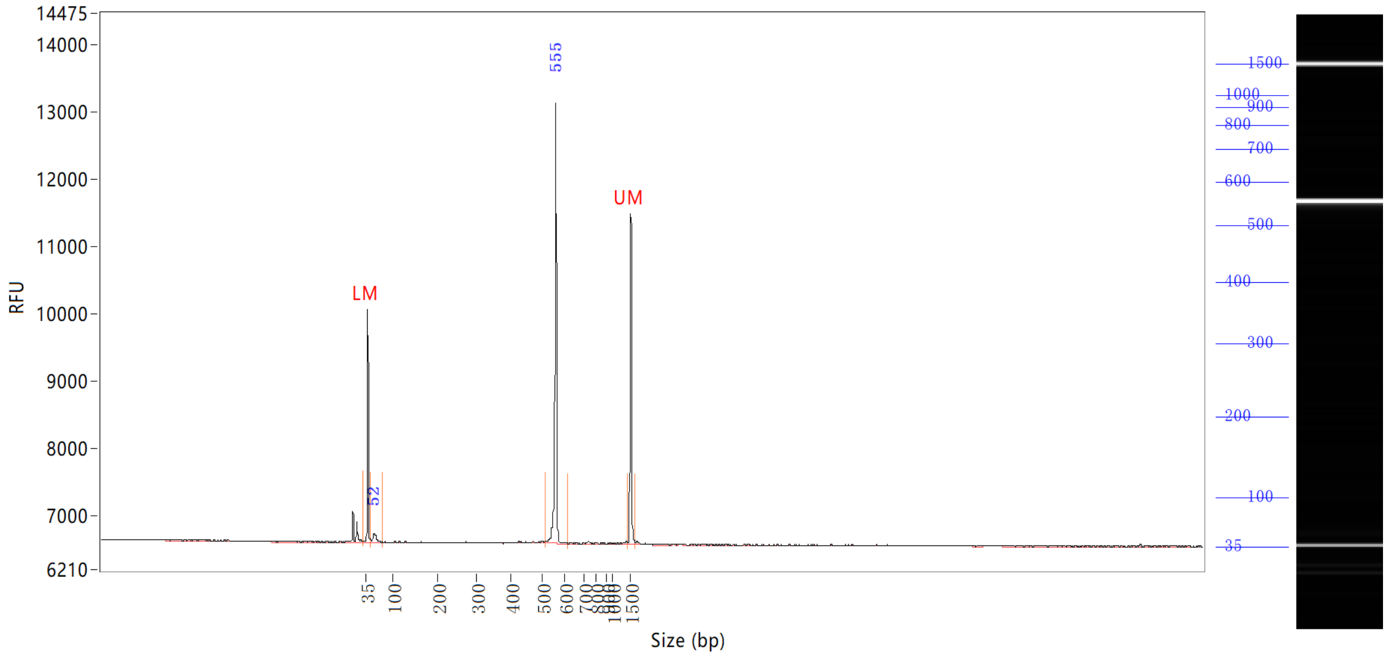

Peak	Size (bp)	Conc. (ng/uL)	From (bp)	To (bp)	Avg. Size (bp)	CV%	RFU	Corr. Peak Area
1	35 (LM)	0.5059	27	44	35	4.83	3451	16.294
2	52	0.7791	44	74	53	9.96	137	2.091
3	555	10.6313	510	614	552	1.26	6552	28.538
4	1500 (UM)	0.5000	1420	1638	1495	1.51	4909	16.106

TIC:	11.4104	ng/uL
TIM:	55.7773	nmole/L
Total Conc.:	13.2414	ng/uL

```
Sample Peak Width (sec): 5     Sample Min Peak Height: 50    Sample Baseline V to V?: Y    Sample Baseline V to V pts: 3
Sample Filter: Binomial        # of Pts for Filter: 3        Sample Start Region (min): 0  Sample End Region (min): 80
Marker Peak Width (sec): 5     Marker Min Peak Height: 500   Marker Baseline V to V?: Y    Marker Baseline V to V pts: 3
Lower Marker Selection: First Peak > 500 RFU                 Upper Marker Selection: Last Peak > 500 RFU
Ladder Size (bp)35, 100, 200, 300, 400, 500, 600, 700, 800, 900, 1000, 1500
Quantification Using: Upper Marker          Final Concentration (ng/uL): 0.5000          Dilution Factor: 12.0
```

 Copyright 2015 Advanced Analytical Technologies, Inc.

Sample: SampA6
Well Location: A6

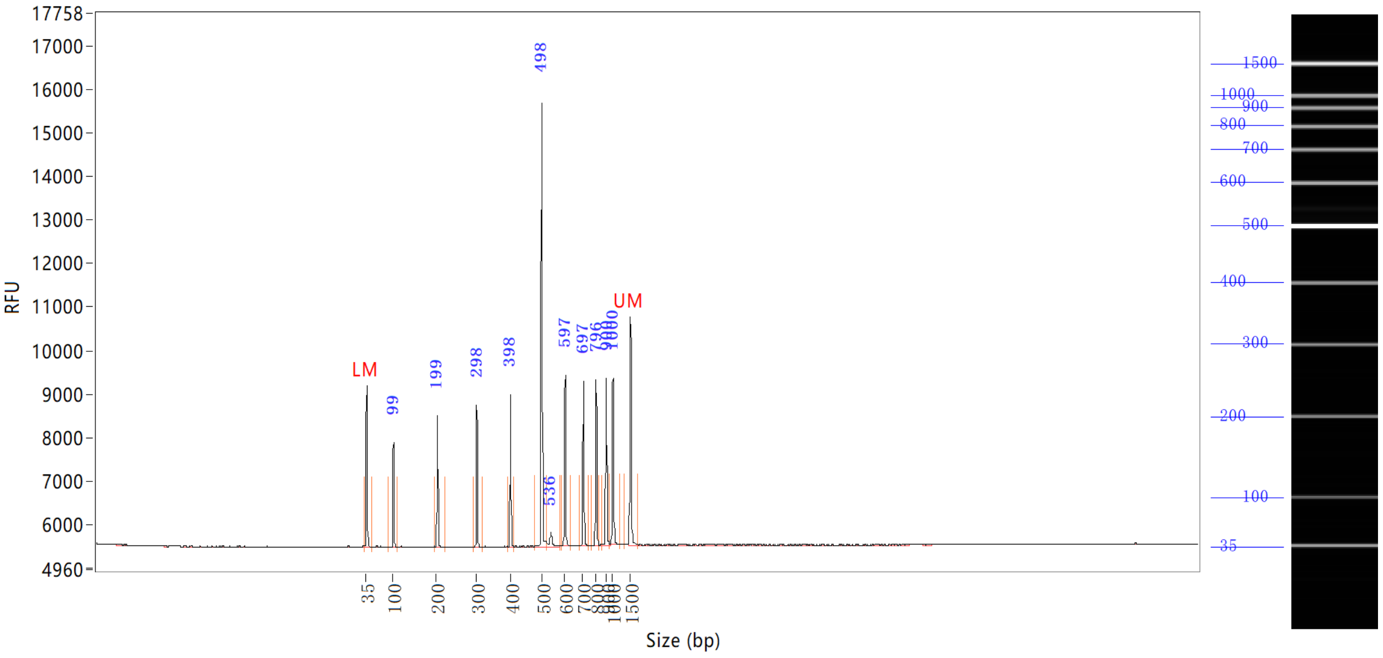

Peak	Size (bp)	Conc. (ng/uL)	From (bp)	To (bp)	Avg. Size (bp)	CV%	RFU	Corr. Peak Area
1	35 (LM)	0.4927	30	51	34	4.67	3708	17.287
2	99	3.2989	91	110	99	1.56	2409	9.645
3	199	3.6167	194	219	199	0.85	3012	10.574
4	298	3.7039	290	317	298	0.56	3274	10.829
5	398	3.7765	391	411	397	0.44	3511	11.041
6	498	11.4123	479	524	497	0.70	10220	33.366
7	536	0.7348	524	578	537	1.58	312	2.148
8	597	4.3425	586	633	596	0.62	3921	12.696
9	697	4.0421	677	740	696	0.61	3799	11.818
10	796	4.0407	763	834	795	0.65	3809	11.814
11	900	4.2043	855	958	897	0.94	3852	12.292
12	1000	4.3022	958	1187	998	2.06	3827	12.578
13	1500 (UM)	0.5000	1354	1730	1497	1.81	5241	17.542

TIC:	47.4750	ng/uL
TIM:	205.4549	nmole/L
Total Conc.:	48.4709	ng/uL

```
Sample Peak Width (sec): 5    Sample Min Peak Height: 50    Sample Baseline V to V?: Y    Sample Baseline V to V pts: 3
Sample Filter: Binomial       # of Pts for Filter: 3        Sample Start Region (min): 0  Sample End Region (min): 80
Marker Peak Width (sec): 5    Marker Min Peak Height: 500   Marker Baseline V to V?: Y    Marker Baseline V to V pts: 3
Lower Marker Selection: First Peak > 500 RFU                Upper Marker Selection: Last Peak > 500 RFU
Ladder Size (bp)35, 100, 200, 300, 400, 500, 600, 700, 800, 900, 1000, 1500
Quantification Using: Upper Marker          Final Concentration (ng/uL): 0.5000          Dilution Factor: 12.0
```

 Copyright 2015 Advanced Analytical Technologies, Inc.

Sample: SampH12
Well Location: H12
Created:
Fit Type: Point to Point

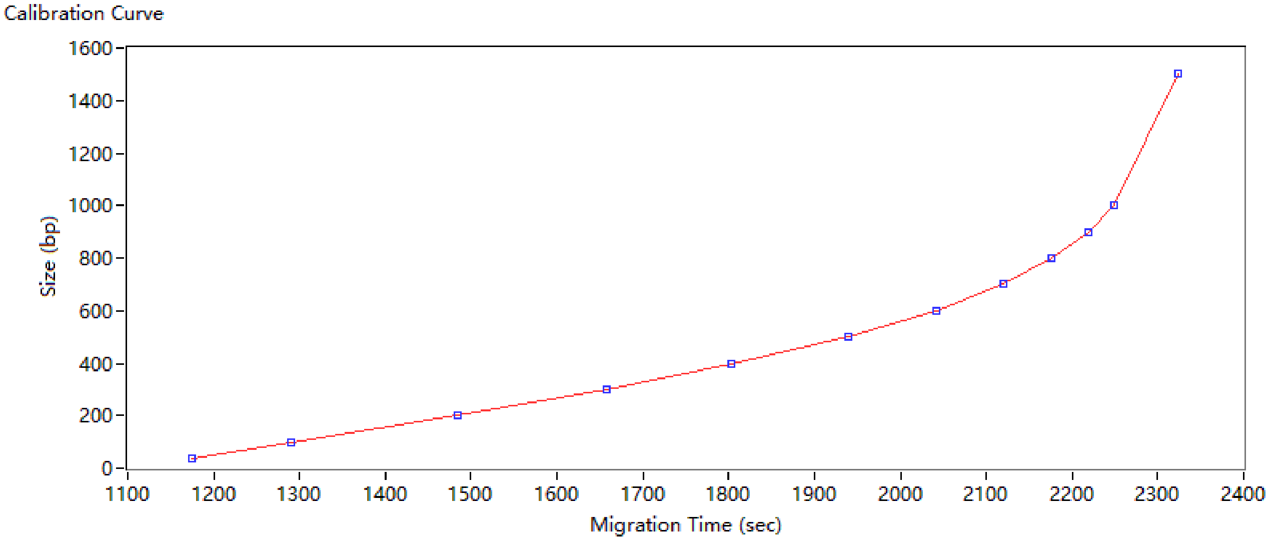

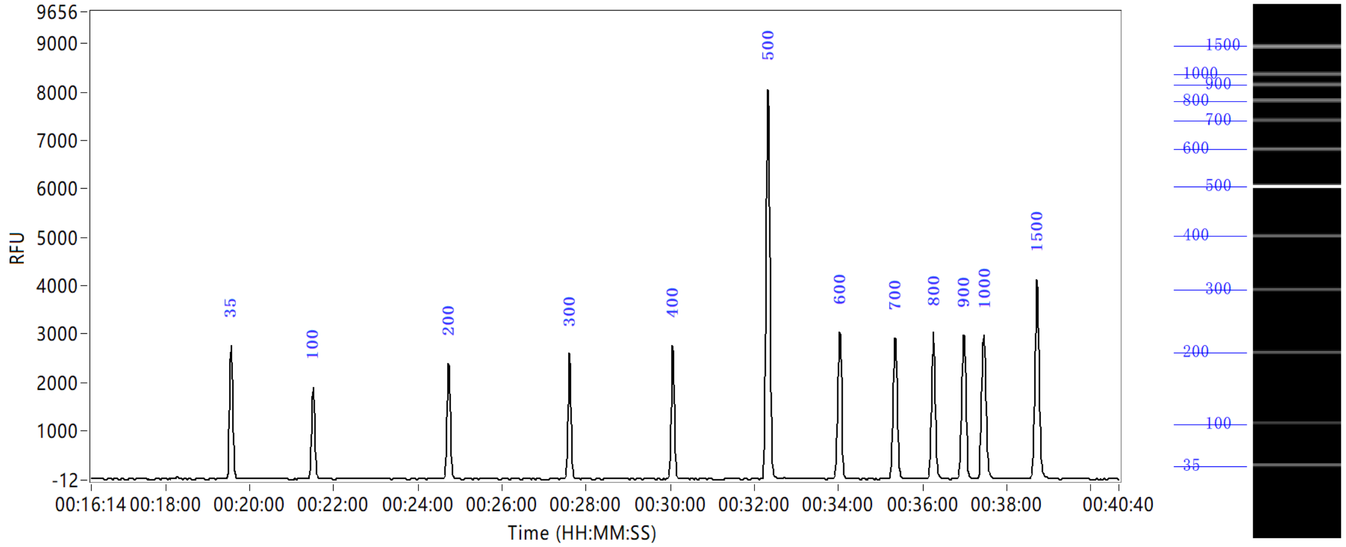

Copyright 2015 Advanced Analytical Technologies, Inc.
